# Clustering of 770,000 genomes reveals post-colonial population structure of North America

**DOI:** 10.1038/ncomms14238

**Published:** 2017-02-07

**Authors:** Eunjung Han, Peter Carbonetto, Ross E. Curtis, Yong Wang, Julie M. Granka, Jake Byrnes, Keith Noto, Amir R. Kermany, Natalie M. Myres, Mathew J. Barber, Kristin A. Rand, Shiya Song, Theodore Roman, Erin Battat, Eyal Elyashiv, Harendra Guturu, Eurie L. Hong, Kenneth G. Chahine, Catherine A. Ball

**Affiliations:** 1 AncestryDNA, San Francisco, California 94107, USA; 2 AncestryDNA, Lehi, Utah 84043, USA; 3 Department of Computational Medicine and Bioinformatics, University of Michigan, Ann Arbor, Michigan 48109, USA; 4 Department of Computational Biology, School of Computer Science, Carnegie Mellon University, Pittsburgh, Pennsylvania 15213, USA; 5 W.E.B. Du Bois Research Institute, Hutchins Center for African and African American Research, Harvard University, Cambridge, Massachusetts 02138, USA

## Abstract

Despite strides in characterizing human history from genetic polymorphism data, progress in identifying genetic signatures of recent demography has been limited. Here we identify very recent fine-scale population structure in North America from a network of over 500 million genetic (identity-by-descent, IBD) connections among 770,000 genotyped individuals of US origin. We detect densely connected clusters within the network and annotate these clusters using a database of over 20 million genealogical records. Recent population patterns captured by IBD clustering include immigrants such as Scandinavians and French Canadians; groups with continental admixture such as Puerto Ricans; settlers such as the Amish and Appalachians who experienced geographic or cultural isolation; and broad historical trends, including reduced north-south gene flow. Our results yield a detailed historical portrait of North America after European settlement and support substantial genetic heterogeneity in the United States beyond that uncovered by previous studies.

## Abstract (web summary)

Genetic data has led to great advances in our understanding of human evolution and dispersal, but information on more recent events is limited. Here, the authors analyse genotypes from 770,000 US individuals to map the fine-scale population structure of North America after European settlement.

Following the arrival of Columbus and his contemporaries, population expansion in the Americas has proceeded at an exceptionally rapid pace, with factors such as war, slavery, disease and climate shaping human demography. Recent genetic studies of the United States and North America have drawn insights into ancient human migrations^1^,^2^ and population diversity in relation to global population structure^3^,^4^,^5^,^6^,^7^,^8^,^9^,^10^,^11^. These insights have been primarily drawn from modelling variation in allele frequencies (for example, refs 11, 12, 13, 14, 15), which typically diverge slowly. This may in part explain why these studies have revealed little about population structure on the time-scale of post-European colonization (1500–2000 AD) that is not directly tied to pre-Columbian diversity within the Americas nor to ‘Old World' populations outside the United States.

In this study, we analyse genome-wide genotype data from over 777, 000 primarily US-born individuals. Among all pairs of individuals, we identify genetic connections defined by sharing a recent common ancestor; when these connections are aggregated into a network, our computational methods reveal densely connected clusters, in which the members of each cluster are subtly more related to each other. Using a unique collection of 20 million user-generated genealogical records, we annotate these densely connected clusters to identify the putative historical origins of such population substructure, and to infer temporal and geographic patterns of migration and settlement. With much greater granularity than previously possible, our analyses demonstrate the impact of subtle, complex demographic forces in shaping the patterns of genetic variation among contemporary North Americans.

## Results

### Identity-by-descent inference

To investigate recent, fine-scale population structure in the United States, we leveraged one of the largest human genetic data sets assembled to date: genome-wide genotypes of 774, 516 individuals born (96%) or currently residing (4%) in the United States (Supplementary Table 1; Supplementary Fig. 1). All individuals were genotyped at 709, 358 autosomal single-nucleotide polymorphisms (SNPs) using the Illumina Human OmniExpress platform as part of the AncestryDNA direct-to-consumer genetic test, and have consented to participate in research (Methods). In this sample, we analysed patterns of identity-by-descent (IBD)^16^, which have been shown to reveal signatures of recent demographic history^3^,^17^,^18^,^19^,^20^,^21^. If two individuals share an ancestor from the recent past, they will likely carry one or more long chromosomal segments inherited IBD from that ancestor. However, a practical difficulty is that since few pairs of individuals share large amounts of IBD due to shared ancestors in recent generations, such data are very sparse. For example, due to recombination and independent assortment, the probability that a particular position in the genome is shared IBD by two descendants sharing a single common ancestor four or more generations ago is <1%. Our large data set overcomes this limitation; even though only 0.2% of possible IBD pairs in our sample share >12 cM total detected IBD, in aggregate we estimated over 500 million such pairs, providing a rich data source for demographic inference.

### Hierarchical clustering and spectral analysis of IBD network

**Figure 1:**
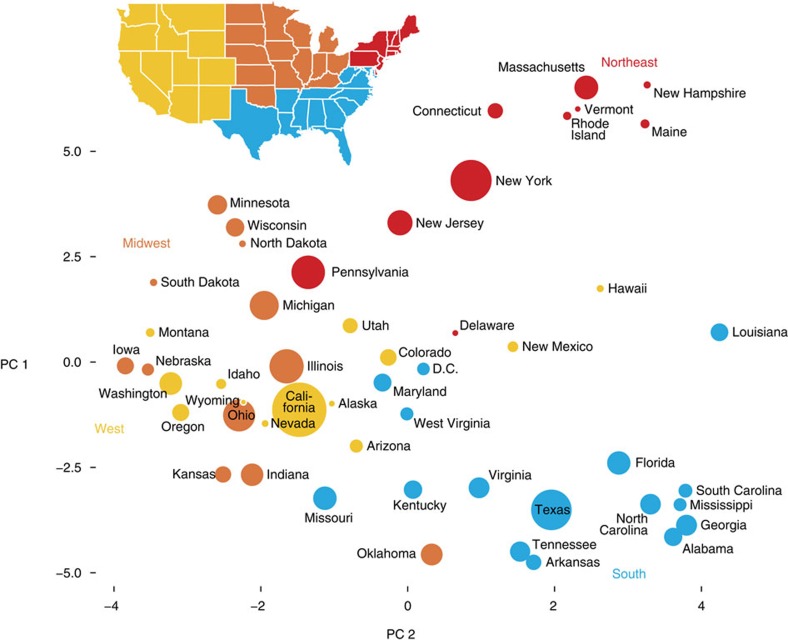
Two-dimensional projection of US states based on cross-state IBD. Principal components (PCs) are computed using kernel PCA, in which the kernel matrix is defined by total IBD between pairs of states, normalized to remove the effect of variation in within-state IBD. US states that share high levels of IBD on average are placed closer to each other in the projection onto the first two principal components. The area of each circle is scaled by number of self-reported birth locations in the state (Supplementary Fig. 1). US states are coloured by geographic region (Northeast, South, Midwest and West). Maps were generated with the maps R package using data from the Natural Earth Project (1:50 m world map, version 2.0). These data are made available in the public domain (Creative Commons CC0).

**Figure 2:**
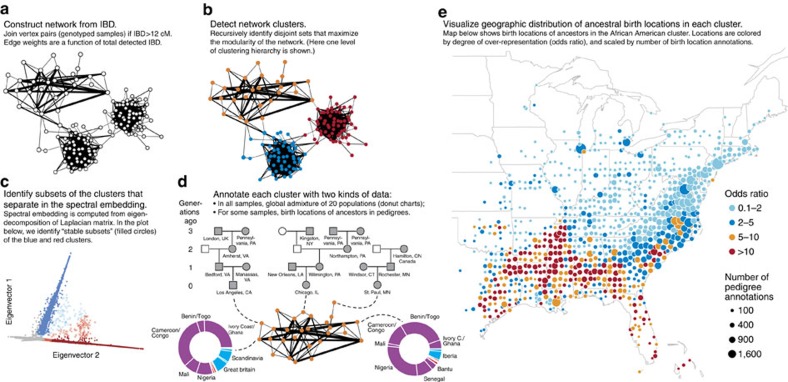
Schematic of workflow for identification and interpretation of clusters. Parts **a–c** summarize the identification of clusters: **a** constructing network from IBD, **b** detecting network clusters, and **c** identifying subsets of clusters that separate in the spectral embedding. Part **d** summarizes the interpretation of clusters by annotating clusters with admixture and genealogical data. Part **e** summarizes the genealogical data—birth location annotations in pedigrees (shaded symbols in **d**)—for the ‘African American' cluster. In **e**, each birth location in the pedigree (here, in generations 0–9, in which generation 0 is the genotyped individual) is converted to the nearest coordinate on a grid, with grid points every 0.5° of latitude and longitude. Point size is scaled by number of birth location annotations in the cluster at the given location, and coloured by odds ratio (OR): the proportion of ancestral birth locations linked to cluster members at that map location over the proportion linked to non-cluster members at the same location. Points on the map with higher odds ratios indicate geographic locations that are more associated with cluster membership. Maps were generated with the maps R package using data from the Natural Earth Project (1:50 m world map, version 2.0). These data are made available in the public domain (Creative Commons CC0).

Our first indication that demography could be inferred from genomic sharing among present-day Americans was the relationship we observed between US geography and the projection of state-level IBD summary statistics onto their first two principal components (PCs); PC 1 is correlated with north-south geography, and PC 2 is correlated with east-west (Fig. 1; Supplementary Data 1). Following this initial observation, we turned to using IBD to discover previously unidentified population structure. Similar in some respects to Gusev *et al*.^16^, and based on principles developed in machine learning and statistical physics, we used a model-free approach to learn structure in a network^22^,^23^,^24^. We hypothesized that structural features of this network could be related to population demography^16^,^25^,^26^,^27^, analogous to the common use of PC analysis (PCA) to infer structure from genetic polymorphism data without specifying a demographic process^11^,^14^. To define the IBD network as a graph (Fig. 2a), each individual is represented as a vertex, and IBD between each pair of individuals is represented as an edge. We applied a weight function to each edge, setting the edge weight >0 only if the estimated total IBD was >12 cM (Supplementary Fig. 2). This choice allowed us to focus on IBD corresponding to more recent demography while reducing spuriously identified IBD^28^. On the basis of this choice, 769,444 (99.3%) of the vertices (individuals) formed a completely connected network; the remaining 0.7% of samples could correspond to populations poorly represented in our sample, and they were not included in our subsequent analyses.

We took a simple, hierarchical clustering approach to infer structure in the IBD network by recursively identifying disjoint sets that maximized the modularity^22^,^23^,^29^. Informally, maximizing the modularity partitions the network so that a relatively large amount of IBD is contained within each of the partitions (Fig. 2b). In the top level of the hierarchical clustering, 99.9% of the IBD network (768,758 out of 769,444 vertices) was subdivided into only six clusters; of these, five each contained over 10,000 individuals. The rest of the network was assigned to many small clusters (at most 101 members). Since these small clusters were difficult to interpret and may correspond to subpopulations that have poor representation in our database, or to unusually over-represented families, we did not investigate them further.

To examine finer-scale population structure, we formed five sub-networks corresponding to the five largest clusters, then partitioned these sub-networks using the same clustering algorithm. We complemented this two-level clustering with a spectral dimensionality reduction technique for network data^24^,^26^,^30^. This yielded a low-dimension representation of the IBD network structure, analogous to PCA applied to genetic polymorphism data. We took a simple approach to infer population structure from the spectral dimensionality reduction by projecting all samples labelled by the hierarchical clustering onto this low-dimensional embedding, then using this data visualization to extract further clusters. These clusters, which we refer to as ‘stable subsets,' are subsets that project away from the origin in the spectral embedding, and represent unusually disconnected parts of the network (Fig. 2c; Supplementary Figs 3–5). While the hierarchical clustering identifies network structure underlying systematic patterns of variation in IBD, including continuous variation (for example, due to isolation-by-distance), visualizing this structure via the spectral analysis allows for isolation of the more discontinuous components of variation in IBD that putatively reflect genetic sharing originating from discrete populations (see Supplementary Fig. 6 for an illustration of these principles in a simulated data set). The results of this hierarchical clustering and the accompanying spectral analysis are summarized in Table 1 (see Supplementary Data 2 for a more detailed summary), and are the focus of our discussion as they most strongly demonstrate the connection between IBD and population structure. In examples below, we highlight the distinction between the hierarchical clustering and stable subsets. When the distinction is unimportant for the discussion, we refer to both generally as clusters.

### Historical annotation of clusters

**Figure 3:**
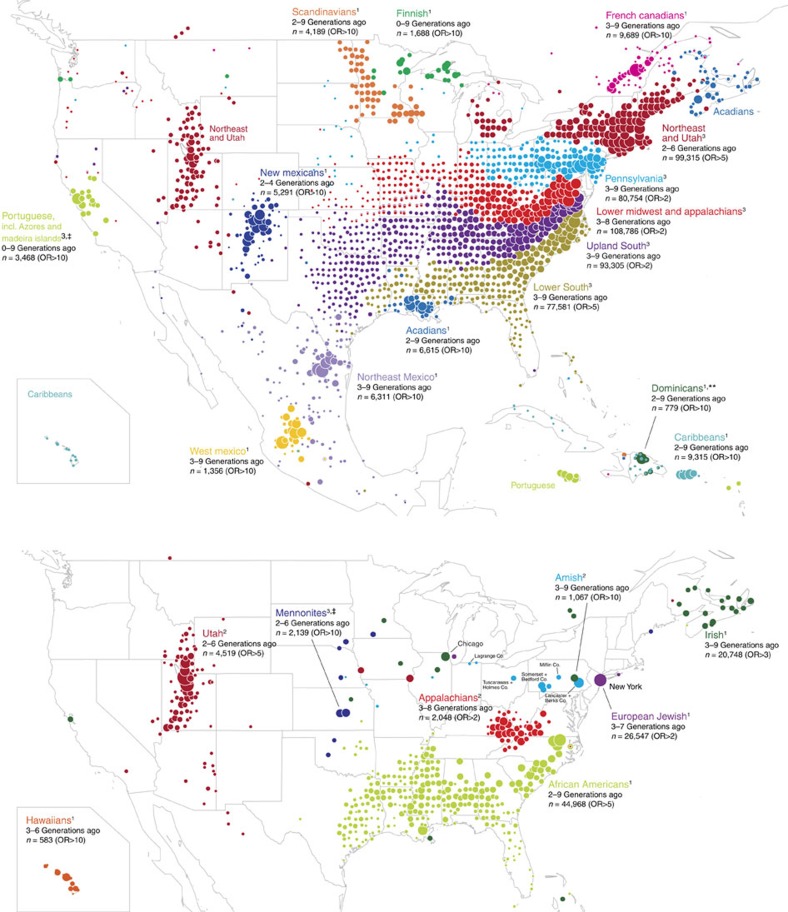
Distribution of ancestral birth locations in North America associated with IBD clusters. Points show pedigree birth locations that are disproportionately assigned to each cluster. Only birth locations with OR>*x* within indicated generations *y*–*z* are plotted, in which parameters *x*, *y*, *z* are chosen separately per cluster to better visualize the cluster's historical geographic concentration; full distributions of ancestral birth locations in the United States, Europe and worldwide are given in Supplementary Figs. 18–20. For each cluster, points are independently scaled by the number of pedigree annotations. See Fig. 2 and Table 1 for more details. Note that clusters are separated into two maps only for clarity. Also note that the concentration of Puerto Rican ancestors in Hawaii probably reflects their arrival there in the early 1900s (ref. 64). Maps were generated with the maps R package using data from the Natural Earth Project (1:50 m world map, version 2.0). These data are made available in the public domain (Creative Commons CC0).

Given the IBD network clustering identified by hierarchical clustering and spectral analysis, we used a combination of admixture estimates and genealogical data to annotate these clusters and connect them to recent, fine-scale demography in North America (Fig. 2d,e). First, we estimated admixture proportions in 20 global populations from the genotypes using ADMIXTURE^12^ (Supplementary Figs 7–11; Supplementary Table 2). Characterization of the clusters with respect to this estimated worldwide population structure is summarized in Table 1. Second, user-generated pedigree data linked to a large proportion of individuals in the data set provided birth dates and locations the ancestors of cluster members (Supplementary Figs 12–17). Such data allowed us to understand the ancestral history of individuals assigned to identified clusters, in particular suggesting birth locations that were over-represented at particular time periods in the pedigrees of individuals assigned to each cluster (Fig. 3; Supplementary Figs 18–21). The cluster names in Table 1 and Fig. 3 were drawn from statistical summaries of these admixture and genealogical data.

## Discussion

Taken together with the IBD network clustering results (Table 1), the visualizations of the genealogical data in North America (Fig. 3) highlight broad-scale demographic trends, as well as patterns specific to individual populations. While no two identified clusters have identical demographic histories, for convenience of discussion we divide the clusters into four broad categories, and present examples of each. The first grouping, which we label as intact immigrant clusters, are likely driven by population structure present before immigration that may have been maintained post immigration. These clusters often feature over-representation of a particular global admixture proportion, localized ancestral birth locations at both the source and destination of immigration, and genetic differentiation (*F*_ST_) comparable to those of the source populations. We label the second grouping as continental admixed groups, the majority of which represent Hispanic/Latino populations. While these clusters all display a characteristic genetic mixture from two or more continents, admixture proportions alone do not distinguish them. Instead, we find that ancestral birth locations, primarily from outside current US boundaries, reveal the groups' more recent origins. The vast majority of our samples are contained in the third set of clusters, which we label as assimilated immigrant groups. Although these clusters typically feature almost exclusively mixed European ancestry and very low genetic differentiation between one another, they can be distinguished primarily by geographic localization of their ancestral birth locations within the United States. Finally, the fourth set of clusters we label as post-migration isolated groups; these groups have historically resided in small or geographically isolated communities within the United States, and are distinguished by stable subsets within the IBD network, suggesting that reduced gene flow with neighbouring groups may have contributed to the identified structure.

The first grouping, intact immigrant clusters, can be attributed to population structure existing prior to immigration to the United States. Despite subsequent admixture following immigration, we found clusters corresponding to Finnish, Scandinavian, Jewish and Irish ancestries—all groups who immigrated to the United States in large numbers within the past 150–200 years—as well as African Americans and individuals with Polynesian ancestry (labelled Hawaiian). Although identification of these clusters was based solely on our IBD clustering approach, their relationship to these immigrant groups is demonstrated by their association with respective global ancestries estimated using a simple, frequency-based model^12^ (Table 1). (Note that, since the OmniExpress genotyping chip better captures European genetic variation, IBD detection may be less accurate for non-European populations, and therefore the association with global population structure could be partly driven by large population-level differences in allele frequencies). We further verify the identity of these clusters by examining ancestral birth locations within the pedigrees of cluster members. Births are generally over-represented within the expected geographic origin of the cluster; for example, ancestral birth locations associated with the Irish cluster are predominantly found in Ireland (Supplementary Fig. 20), and Scandinavian cluster birth locations are disproportionately found in Norway and, to a lesser extent, Sweden and Denmark (Supplementary Fig. 20). An exception is the African American cluster, which is not associated with West African birth locations; this is unsurprising given the lack of genealogical records tracing back to Africa. The majority of these groups also show evident geographic localization within the United States (Fig. 3), corresponding to known migration patterns; for example, the Scandinavian and Finnish clusters are concentrated in the Midwest^31^, while the African American cluster closely coincides with regions of high self-reported African ancestry^32^. Reinforcing the connection between IBD clustering and global population structure, we observe that the degree of disconnectedness in the IBD network often correlates strongly with amount of admixture (Supplementary Fig. 22; Jewish *r*^2^=0.97, Finnish *r*^2^=0.67). Also, F_*ST*_ (which measures differentiation in common genetic variation) between the Jewish, Irish, Scandinavian, Finnish, Hawaiian and African American clusters (F_*ST*_=0.001–0.084; Supplementary Table 4 and Supplementary Discussion) closely matches F_*ST*_ estimated from comparable worldwide populations sampled from the geographic locations representing these population's origins (for example, refs 5, 33, 34).

**Figure 4:**
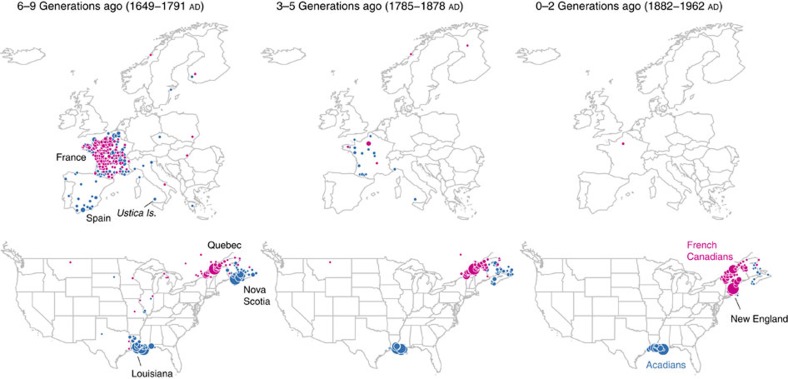
Genealogical data traces origins of Cajuns/Acadians in Atlantic Canada (blue) and migration of French Canadians (magenta) to the US. Map locations are plotted if OR>10 within the indicated range of pedigree generations (date ranges give the 5th and 95th percentiles of birth year annotations). Points are scaled by number of pedigree annotations, separately for each of the six maps. Note that not all current political borders are shown. See Fig. 2 for more details. Maps were generated with the maps R package using data from the Natural Earth Project (1:50 m world map, version 2.0). These data are made available in the public domain (Creative Commons CC0).

We highlight two additional immigrant clusters with clear geographic concentrations both within and outside the United States: Acadians and French Canadians. During the mid 18th century, Acadian residents (modern-day Atlantic Canada) were expelled by the British and took refuge in various colonies, eventually including Louisiana, then under Spanish control^35^. On the other hand, in the late 19th century, large numbers of French Canadians left rural Quebec in search of economic opportunities in New England and the northern United States^36^. We identified two clusters in the IBD network likely corresponding to these distinct descendant groups (Table 1). Pointing to their common origins, these two clusters overlap substantially in the spectral analysis, though they can each still be identified as stable subsets (Supplementary Fig. 4). The genealogical data allow us to corroborate both the shared and unique portions of these migratory histories with exceptional detail, highlighting the historical forces that may have led to the enrichment of IBD within these clusters (Fig. 4). As a final point, the low genetic differentiation (F_*ST*_=0.001) between these groups, and their nearly indistinguishable admixture proportions, illustrates that standard methods may have difficulty separating them as we do here.

Next, we identified continentally admixed clusters, including Colombians and groups in Central America and the Caribbean, labels which are primarily inferred from the ancestral birth locations of cluster members. While these groups all display a characteristic signature of ancestry from multiple continents, it is difficult to distinguish among them using only global admixture proportions. Several genetic studies have sought to identify genetic structure within the Caribbean and Central America^6^,^7^,^8^,^9^,^10^. However, none have been able to confidently distinguish these groups based on genetic data alone, likely due to their high shared levels of Native American, European and, in some cases, West African ancestry, with only subtle levels of population differentiation between them (F_*ST*_=0.001–0.011; Supplementary Table 4). This example again highlights the power of using IBD to identify recent population structure.

It should be noted that, in some cases, the clusters we identify using IBD could be more reflective of US immigration patterns than inherent structure within source locations^10^. For example, two of the Mexican clusters we identified are annotated with birth locations most concentrated in Jalisco and Monterrey, the predominant traditional sources of emigration to the United States^37^,^38^. The over-representation of West Mexican birth locations in southwestern United States and Northeast Mexican birth locations in Texas, particularly South Texas in recent generations (Supplementary Figs 20,23), confirms known patterns of migration from eastern versus western Mexico to the United States^37^. Such genetic structure, particularly as it relates to Mexican migration to the United States, has not yet been identified from genetic data.

**Figure 5:**
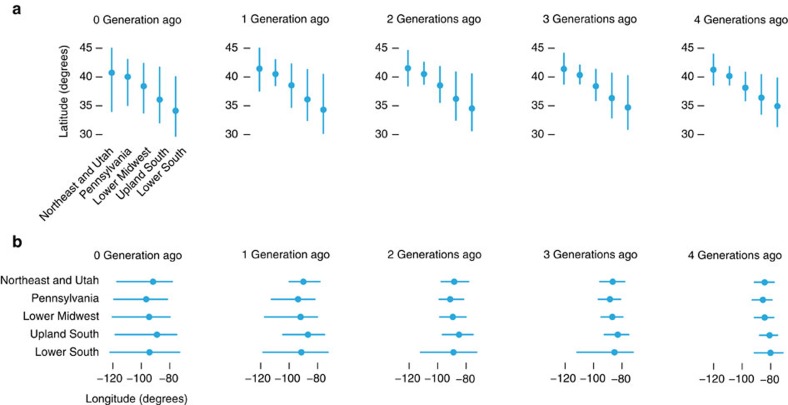
Five largest clusters predict North-South US geography across multiple generations. In **a**, each circle gives the mean latitude (in degrees) of all pedigree birth location annotations within a given generation linked to genotypes assigned to each of the five clusters; end points of vertical bars represent 10th and 90th empirical percentiles. These statistics are compiled from pedigree annotations with US birth locations only. Note that ‘0 generations ago' refers to genotyped individuals. For contrast, **b** shows the same statistics, but for longitude instead of latitude. Each degree of longitude or latitude is roughly equivalent to 100 km. Examples of US cities by latitude and longitude include Boston, MA (42.4, −71.1), New Orleans, LA (30.0, −90.1) and Sacramento, CA (38.6, −121.5).

The five largest clusters (third set of rows in Table 1), which we describe as assimilated immigrant clusters, account for a large portion (60%) of the IBD network and exhibit a markedly different profile. Lacking distinctive affiliations to non-US populations, they show almost no differentiation in allele frequencies (F_*ST*_ at most 0.001; Supplementary Table 5) and high levels of IBD to non-cluster members (Supplementary Data 2), suggestive of high gene flow between these clusters. Moreover, few members of these clusters could be assigned to a stable subset, indicating that this clustering is largely driven by continuous variation in IBD. Genealogical data reveal a north-to-south trend (Fig. 5), most consistently east of the Mississippi River (Fig. 3). These findings imply greater east-west than north-south gene flow, which is broadly consistent with recent westward expansion of European settlers in the United States, and possibly somewhat limited north-south migration due to cultural differences. While the precise numbers and boundaries of these clusters are not necessarily meaningful and may be partly driven by the assumption that inter-cluster connectivity follows a random graph model^39^,^40^, these findings demonstrate that isolation-by-distance, and specifically geography in the continental United States, can be captured from IBD alone.

Finally, we identified several clusters corresponding to post-migration isolated groups—historical groups who, despite possibly maintaining high levels of diversity and gene flow, likely experienced some geographic or cultural isolation during or following migration to the United States. One such cluster represents the Amish, a distinct ethno-religious minority that first arrived to the United States from Europe in the 18th century^41^; the genealogical data associated with the Amish cluster pinpoint individual counties in Midwestern states and Pennsylvania with present-day Amish communities (Fig. 3; Supplementary Fig. 20). The clustering of IBD in Utah is most likely attributed to population growth of descendants of Mormons, who settled in Utah in the mid-1800s (ref. 42; Supplementary Figs 20,24). In addition, we identified a cluster concentrated near the Cumberland Mountain range that is suggestive of residents of Appalachia, people who experienced delayed economic development and regional isolation up until the 20th century^43^,^44^. We emphasize that identification of IBD clusters coinciding with distinct historical groups does not imply they are ‘genetic isolates'; these groups still could have maintained high levels of diversity and gene flow. We also stress that these clusters are not necessarily representative of the entire population suggested by our labelling.

Our discussion thus far has centred on a simple two-level clustering of IBD and related stable subsets identified within the top 40 eigenvectors in the Laplacian matrix. An unresolved issue common to both hierarchical clustering and spectral analysis is that stopping criteria are not well established: when to stop subdividing clusters in hierarchical clustering and how many dimensions to analyse in spectral analysis. While stopping conditions have previously been proposed for both methods^22^,^24^, our experience with these data suggests these criteria either do not apply or do not work well in this context (Supplementary Materials for additional discussion of this topic). We expect that with more data, it will be possible to continue to improve the resolution of our clustering. Indeed, additional clusters informative of population structure emerged when we proceeded to the third level of the hierarchical clustering (Supplementary Figs 25–27). For example, additional clustering discriminated Italians, Scottish, Norwegians and Eastern Europeans, and yielded fine-scale geographic structure in Ireland (Supplementary Fig. 26), the southern United States (Supplementary Fig. 27), and on the Island of Puerto Rico (Supplementary Fig. 21). However, since the clustering of successively smaller sub-networks is complicated by several factors, including the rapid decline in IBD signal, interpretation of these clusters requires additional validation.

**Figure 6:**
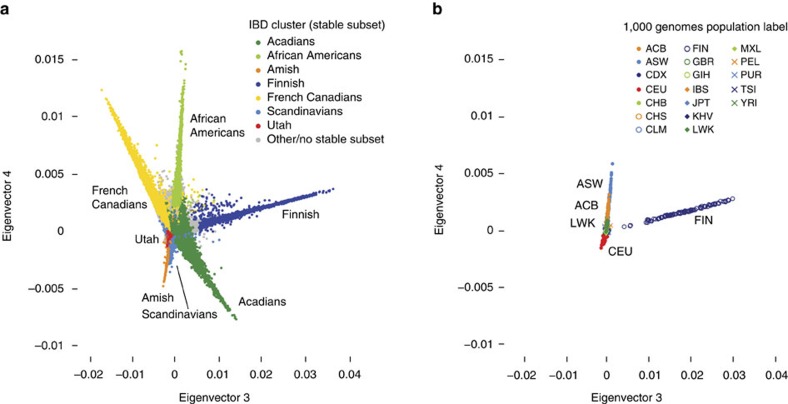
Illustration of spectral analysis in AncestryDNA and 1000 Genomes samples. (**a**) Projection of AncestryDNA genotype panel onto two dimensions of the IBD network spectral embedding. Inferred assignments to some stable subsets are shown. Note that some of the stable subsets shown here project away from the origin in other dimensions of spectral embedding, and are sometimes more distinguishable in those dimensions; see Supplementary Figs 4,5. (**b**) Projection of 1000 Genomes^33^ samples onto same two dimensions of spectral embedding. Projection is computed from IBD estimated between all pairs of AncestryDNA and 1000 Genomes samples. Samples in **b** are coloured according to the expert-provided population label. Population labels include ACB, African Caribbean in Barbados; ASW, people with African Ancestry in Southwest USA; CEU, Utah residents with Northern and Western European ancestry; FIN, Finnish in Finland; LWK, Luhya in Webuye, Kenya. See Supplementary Data 3 for interpretation of other population labels. Note that most samples are concentrated near the origin, which explains why most population labels are not visible in **b**.

Finally, for additional validation of our demographic inferences and methodology, we estimated IBD among individuals in a publicly available genetic data set—1,816 genotype samples from the 1000 Genomes Project^33^. We then compared the expert-provided population labels accompanying these samples against their projection onto the spectral embedding generated from the AncestryDNA genotype data (Fig. 6; Table 1; Supplementary Figs 4,5; Supplementary Table 3). We found that 1000 Genomes samples from Finland (FIN, Finnish in Finland) projected onto the same region as our identified ‘Finnish' cluster (Fig. 6b), and Puerto Rican (PUR) samples projected onto our identified ‘Caribbeans' cluster (Supplementary Fig. 4). As another example, many 1000 Genomes samples with Mexican ancestry from Los Angeles (MXL) projected onto the West Mexico cluster (Supplementary Fig. 4), supporting the possible connection between identified clusters and sources of Mexican immigration to the United States. Finally, European-descent samples collected in Utah (CEU, Utah residents with Northern and Western European ancestry) also projected onto the Utah cluster we identified (Supplementary Fig. 5). This analysis underscores that the population structure we identified from IBD data corresponds in several cases to structure that has been well characterized in other data sets.

We have demonstrated that patterns of IBD can be used to infer population structure indicative of very recent, documented historical patterns. Some of this population structure has been previously identified using genetic data, whereas the genetic separation of other groups of historical importance—such as regions of Mexico corresponding to different sources of US immigration, and the New Mexican cluster corresponding to the *Nuevomexicanos,* European colonial settlers from New Spain^45^—is a major contribution of this work.

While IBD is clearly a rich data source for detecting subtle genetic differentiation, it is also subject to inherent limitations requiring large, unbiased sample collections. For example, the inclusion of a small number of Jamaicans in the Portuguese cluster (Fig. 3) defies any documented historical explanation that we could identify and most likely arises from fitting clusters to a small number of genetic connections; this illustrates that robust clustering of IBD requires large samples. Also, although our results recapitulate much recent demography in the United States and North America, we do not identify known structure in the United States among some present-day immigrant and other groups that are poorly represented in our sample, such as Southeast Asians and Chinese. This suggests that additional structure will emerge with larger and more diverse samples.

Our interpretation of the IBD network clustering partially relied on genealogical data generated by individuals who have taken an AncestryDNA test. These genealogies are in part biased by the type and scope of historical records made available at Ancestry.com, as well as by user generation of these data. In the supplement, we briefly discuss some of the challenges and possible limitations in using genealogical data collections to interpret population structure. Several challenges include missing data (for example, Jewish genealogical records from many parts of Europe), and the fact that using measures of pedigree over-representation to characterize clusters may omit areas of relevance to a particular population if they are less distinctive (for example, Northern US cities do not feature prominently as over-represented in the African American cluster even though African Americans are historically well established in many of these cities). Despite such challenges, the scale and diversity of these data—322,683 pedigrees linked to genotyped samples in the United States alone and over 20 million total pedigree annotations—allow us to infer detailed historical portraits of the identified clusters, and would have been difficult to achieve by curation from professional genealogists. The geographic patterns of the clusters revealed by this data demonstrate that user annotation errors are overwhelmed by the abundance of high-quality information for annotation.

Our main methodological contribution was to demonstrate that existing network analysis techniques can be leveraged to uncover extensive, fine-scale population structure of historical relevance from IBD data. Similar ideas have been explored in previous research^16^,^25^,^26^,^27^. In our work, we investigated the benefits and limitations of two complementary approaches to analysing IBD network structure. Both approaches can be rapidly applied to large networks, provided that the networks are sparsely connected. The first approach was based on partitioning the network to approximately maximize the modularity measure^22^,^23^,^29^. This approach automatically detected interesting population structure, but did not provide control over the number of clusters. The second approach was based on spectral analysis techniques^24^,^26^,^30^. A key feature of this approach is that it provided a low-dimensional representation of network structure, potentially overcoming the unnatural assumption that each sample belongs to a single cluster. Our method for extracting discrete clusters, or ‘stable subsets,' from the spectral analysis was motivated by the need to implement a practical solution to summarizing the network structure, analogous to the use of PCA to identify clusters (for example, refs 15, 46).

Hierarchical clustering based on variation in haplotype frequencies, such as that implemented by fineSTRUCTURE^13^, has also recently yielded new findings into fine-scale population structure^47^,^48^. Since it is best suited to smaller genetic data sets (<10,000 samples), and scales poorly to large samples, fineSTRUCTURE should be viewed as complementary to our approach, which is expected to be most successful when applied to very large cohorts due to the sparseness of IBD data. Although our study focused on the pattern of genetic connections defined by long IBD segments suggestive of recent common inheritance, in principle these same techniques could be used to recover structure from connections representing more distant common ancestry provided that the shorter IBD segments are still estimated with high accuracy.

In summary, the discovery of fine-scale structure from IBD has highlighted detailed substructure of the United States, and a demographic portrait that is remarkably consistent with geography and post-European colonization of North America. In addition to demographic insights, the identification of clusters corresponding to genetically differentiated groups may aid in the development of targeted biomedical research cohorts. For example, we find clear examples in our data where disease-risk variants are present at higher frequencies in identified clusters (Supplementary Table 6); these include a risk allele for prostate cancer^49^ that has a frequency of 5.6% in the African American cluster but is very rare (0.1%) outside the cluster, and a protective allele for squamous cell lung carcinoma^50^ that is 10 times more common in the Finnish cluster. It is likely that a more comprehensive examination of disease-related genetic variation in some of the less well-characterized clusters that we identified may provide novel insights into fine-scale patterns of genetic disease risk in the US population.

## Methods

### Sample collection

All AncestryDNA samples included in this study were collected from AncestryDNA customers, who have agreed to the informed consent for the Independent Review Board approved Ancestry Human Diversity Project (Quorum Review #26168/1)—an AncestryDNA sponsored research project. Samples obtained from external sample collections were included only in the ADMIXTURE reference panel and were used in accordance with any applicable restrictions. Following sample quality-control steps described in Supplementary Methods, we obtain a final panel of 774,516 genotyped samples consented to participate in research. Consult Supplementary Methods for more details on sample collection procedures.

### Genealogical data

We compile statistics from pedigree data linked to genotyped individuals to better understand the historical and geographical context for the IBD clustering. After quality-control steps (Supplementary Methods), we obtain a final set of 432,611 genotyped samples linked to non-private pedigrees. We only include pedigree nodes corresponding to ancestors of the genotyped individuals. We use two types of information in our analyses of associated pedigree data: birth year and birth location (longitude and latitude). Among DNA samples linked to pedigrees, 322,683 (96% of reported birth locations) were born in the United States, 13,748 (4% of reported birth locations) were born outside the United States and 96,180 (22% of all DNA samples linked to pedigrees) have unreported birth locations. See Supplementary Methods for additional details.

### Genotyping

Customer saliva sample accessioning, DNA extraction, and genotyping were completed by the Illumina FastTrack Microarray Services labs. Customer genotype data for this study was generated using the Illumina Human OmniExpress platform. This genotyping array assays 730,525 SNPs (709,358 SNPs are on autosomal chromosomes) and small indels across the genome. The SNPs on this array were carefully selected to capture the majority of common genetic variation in European and other worldwide populations. (Note that genotype data on sex-linked chromosomes are not used in this study). Genotypes were called by Illumina technicians using the GenomeStudio platform.

### Genotype quality-control procedures

We perform extensive quality-control checks for each processed AncestryDNA customer sample. We have developed an almost entirely automatic workflow to signal problematic genotype samples. This process includes the following steps: (1) identifying and removing any duplicate samples (these samples are possibly identical twins); (2) identifying and removing samples with a per-sample call rate <98%, since low call rate can be indicative of either a poor-quality DNA sample or a technical failure in the genotyping array; (3) identifying discrepancies between gender inferred from the DNA sample and self-reported gender recorded by the customer during test activation; and (4) flagging an unusually high rate of genotype heterozygosity, which can indicate sample cross-contamination. We tolerate small numbers of samples failing checks 3 and 4. Additional tests are applied to each batch of 96 samples on a genotyping plate to identify and remove cases of large-scale laboratory mistakes such as incorrect plate rotation. If more than two samples on a single plate have mismatching genders or high heterozygosity, the entire plate of samples is withheld for manual examination. Samples that pass all quality-control tests proceed to the analysis pipeline; samples that fail one or more of the above tests must be collected again from customers, or manually cleared for analysis by lab technicians.

### SNP quality-control procedures

As these data were generated over a period of 3 years, the DNA product has gone through three revisions, mainly due to beadpool depletion and updates to array geometry. To minimize the impact of different versions of the array on any inferences made from the genotype data, we have developed a versioning quality-control procedure. Briefly, we re-genotype a collection of 180 reference samples for each new array version, and assess genotyping performance in terms of SNP presence/absence, genotype call rate and genotype discordance. If a SNP fails at least one of these assessments for a particular version of the array—for example, the SNP is absent due to probe dropout in re-manufacturing, its call rate is <95%, or its genotype discordance is >1%—the genotypes for that SNP are treated as missing for all samples processed on this version of the array. In the worst case, the genotypes of ∼2.6% SNPs were treated as missing. Any genotypes treated as missing were later imputed (see below).

### Genotyping phasing

Our method for phasing genotypes is similar to BEAGLE^51^, but with substantial improvements to achieve high phasing accuracy on a large scale. Our strategy is to first learn BEAGLE haplotype models from a reference panel of 217,722 genotype samples previously phased at 633,299 autosomal SNPs, which includes 558 phased samples from Phase 1 of the 1000 Genomes project^34^,^51^, then we use these models to quickly phase new samples. See Supplementary Methods for details on design of the phasing reference panel, modifications to BEAGLE to accommodate large data sets, and empirical evaluation of the phasing algorithm.

### Detecting IBD

For computational scalability, we have developed our own distributed-processing implementation of the GERMLINE algorithm^52^ to detect long chromosome segments suggestive of inheritance from a recent common ancestor (‘IBD segments'). We apply this method to the phased genotypes to detect pairs with total shared IBD >5 cM. Although other methods have been developed for accurate detection of IBD^53^, most of these methods scale quadratically with the number of samples, hence are not suitable for our data. GERMLINE has been shown to be particularly inaccurate for IBD segments <4 cM^28^, but this is less of a concern here since these short IBD segments contribute little or no weight to the IBD network.

Our algorithm produces the same output as GERMLINE, but offers two computational advantages that allow us to efficiently handle hundreds of thousands of phased genotypes: first, it distributes the computation over a Hadoop compute cluster; second, it stores the phased genotypes in a database so that new samples can be efficiently compared with previously processed samples. The inputs to our GERMLINE implementation are the phased genotypes, and the genetic distances (in cM) between consecutive pairs of SNPs. (We use interpolated HapMap genetic distances^54^). The algorithm output reproduces the same result as GERMLINE 1.5.1 with the following command-line arguments: germline -bits 96 -err_hom 0 -err_het 0 -min_m 5. In an effort to reduce computational cost and the rate of false positives in identifying IBD segments >5 cM among all pairs of >700,000 individuals, we set the ‘bits' parameter to a value slightly larger than some have recommended^55^, and we do not tolerate homozygous or heterozygous mismatches. The empirical distribution of IBD grows exponentially with decreasing IBD length, as expected (Supplementary Fig. 28).

### Visualizing geographic patterns of IBD

To get an initial suggestion that IBD in our large, un-curated sample might be informative of demography within the United States, in a preliminary phase of our study we compiled IBD statistics aggregated by US state, in which samples are assigned to states based on self-reported birth location. More precisely, we tabulate the total amount of IBD shared by individuals born in the same state (‘within-state IBD') and between states (‘cross-state IBD') from 322,683 genotypes with self-reported US birth locations. We only count pairs if total IBD is >12 cM. To summarize the state-level IBD data in a single figure (Fig. 1), we use kernel PCA^56^, implemented in *R* package *kernlab*^57^. We project US states onto the first two PCs, in which the kernel matrix is defined by total cross-state IBD, normalized to eliminate the effect of variation in total within-state IBD on the projection. More precisely, we define an *n* × *n* kernel matrix with entries *K*(*i*, *j*)=*t*(*i*, *j*)/{*d*(*i*) *d*(*j*)}^1/2^, where *n*=51 is the number of states including Washington, DC, *t*(*i*, *j*) is the cross-state IBD for *i*≠*j* and within-state IBD for *i*=*j*, (Supplementary Data 1), and *d*(*i*)=*t*(*i*, 1)+*t*(*i*, 2)+…+*t*(*i*, *n*) is the total IBD between state *i* and all other states, including within-state IBD *t*(*i*, *i*).

### Estimating admixture proportions from genotype data

Based on an individual's genotypes at 112,909 SNPs, we estimate the proportion of their genome that is attributed to different ancestral populations. We have subdivided the global human population into *K*=26 regions (Supplementary Fig. 7; Supplementary Table 2). We use the program ADMIXTURE^12^, together with a curated reference panel of 3,000 putatively single-origin (‘labelled') genotype samples, to jointly estimate admixture proportions in the unlabelled customer samples from their genotypes. This panel includes 855 samples from the Human Genome Diversity Project^58^,^59^, samples from a proprietary AncestryDNA reference collection, and putatively single-origin samples from consenting AncestryDNA customers.

To validate our admixture estimates against public collections where the genotype samples have been annotated by experts, we estimated admixture proportions in 1,043 Human Genome Diversity Project (HGDP) samples (of which 855 are also included in the reference panel) and 1,816 unrelated samples genotyped as part of the 1000 Genomes project^33^ using the Illumina OMNI 2.5M genotyping chip. (Note that none of these 1000 Genomes samples are included in the reference panel). These results are summarized in Supplementary Data 3. Overall, the admixture estimates align closely with the expert-provided population labels; that is, individuals are assigned higher proportions in the appropriate ancestral populations. For example, the Han HGDP samples are attributed 86–96% to the Asia East population. Many of these test samples are expected to be admixed (for example, ASW and MXL), and exhibit admixture in the expected proportions. Two of the defined ancestral populations, ‘Mali' and ‘European Jewish', could not be validated from these data because these ancestral populations contribute at most a small proportion to any of the test samples. We note that admixture proportions for populations that are less genetically differentiated, such as Great Britain and Europe West, are expected to exhibit less accurate admixture estimates using ADMIXTURE; these are typically combined in our calculations when reporting final admixture statistics for each cluster (Table 1; Supplementary Data 2). Finally, since few samples included in our IBD analyses have large contributions from individual West African ancestral populations, we collapse admixture proportions for all West African regions into a single statistic, for a total of 20 reported populations. Consult Supplementary Methods for more details on design of the reference panel, selection of SNPs for admixture calculations, and ADMIXTURE parameter settings.

### Constructing the IBD network

To define the IBD network, we apply a weight function, *w*[*e*(*i*, *j*)] € [0,1], to each edge *e*(*i*, *j*). We define *w*[e(*i*, *j*)] as the proportion of total IBD lengths observed in simulated genotypes that are due to relationships separated by at most eight reproductive events, or meioses (corresponding to common ancestors at most four generations back), although it may reflect more distant relationships for subpopulations that conform less closely to our simulations. This empirical distribution is fit to the Beta cumulative density function, and this fitted distribution (with scale parameters *α*=2, *β*=200) defines the weights for all edges in the network (see Supplementary Fig. 2 for more details). We remove all edges corresponding to pairs with total IBD <12 cM since they signal the target familial relationships <6% of the time, and therefore contribute little weight to the network. See Supplementary Methods and Supplementary Figs 29,30 for a description of the simulations, and a detailed rationale for this choice of edge weight function.

### Hierarchical clustering of IBD network

To identify network modules, we employ a simple and fast heuristic algorithm, the multi-level or Louvain method^29^, implemented in the igraph R package^60^, which heuristically maximizes the modularity by recursively merging subgraphs. (Note that the multi-level algorithm internally generates a hierarchy as it iteratively optimizes the modularity, but we do not use this internal hierarchy in our results). In an attempt to reduce clustering of ‘extended families,' before running the clustering algorithm we remove all edges in the network corresponding to total shared IBD >72 cM. Since this represents only 0.2% of all edges, removing these edges has little effect on our ability to detect larger modules. In addition to the multi-level method, we tested two other methods, both implemented in igraph, that have low computational complexity—*O*(*m*), where *m* is the number of edges—and so could feasibly be applied to our network: the Infomap method^61^, and the label propagation method of Raghavan *et al*.^62^. Although all three provided similar higher-level clustering, only the multi-level method was able to identify substructure within the portion of the network that represents the vast majority of the sample—samples primarily of European or African descent (African Americans). Even though the multi-level method partitions this sub-network of 687,470 genotyped individuals into only two clusters, no other tested algorithms were able to identify non-trivial structure within this sub-network. This constitutes the main motivation for using the multi-level community detection algorithm. See Supplementary Methods for more details, including the procedure for recursively subdividing the IBD network using the multi-level algorithm.

### Spectral analysis of IBD network

The spectral analysis is based on the Laplacian eigenmaps method^30^, which has close connections to spectral clustering^24^. Here we briefly describe computation of the spectral embedding; our procedure for identifying ‘stable subsets' from the spectral embedding is described in the Supplementary Methods. The Laplacian eigenmaps method is derived from a spectral decomposition of the (normalized) Laplacian matrix, L=D^−1/2^ WD^−1/2^, where W is the *n* × *n* weighted adjacency matrix with entries *W*(*i*, *j*)=*w*[*e*(*i*, *j*)], and **D** is the *n* × *n* diagonal matrix in which diagonal entry *D*(*i*, *i*) is equal to the *degree* of node *i*, or the sum of the edge weights *w*[*e*(*i*, *j*)] for individual *i*. Here we define *W*(*i*, *i*)=1 for all *i*, so that there is always a nonzero probability of remaining at the same occupancy state in a random walk of the graph^24^. We define the *spectral embedding* as the first *m* eigenvectors of the normalized Laplacian. Here we limit each spectral embedding to the top *m*=40 eigenvectors, primarily for manageability of the analysis procedure. We cannot use the ‘eigengap' heuristic^26^ to choose *m* because it only appropriate to use if the network contains well-pronounced modules^24^, which is not the case here.

Once we have completed the spectral analysis of the completely connected graph with 769,444 vertices, we compute a second spectral embedding from a subgraph with 586,147 vertices that is obtained by first removing the small sets of individuals and the clusters that project away from the origin in the initial spectral embedding. This step is taken because the spectral decomposition captures the most dominant modular structure in the network, and possibly obscures other, more subtly disconnected subsets. Briefly, to define stable subsets, we visualize the spectral embedding, labelled by the hierarchical clustering, and extract subsets with the same label that project away from the origin. Using this method (described in detail in the supplement and in Supplementary Fig. 31), we identify 18 stable subsets from the spectral embedding (Supplementary Data 2): 10 in the initial spectral embedding, and 8 more in the subgraph embedding. Projecting new samples not included in the IBD network onto the spectral embedding—in particular, the 1000 Genomes samples used as a validation—is described in the supplement.

### Historical and geographic interpretation of clusters

Once we have completed hierarchical clustering and spectral analysis of the IBD network, we use the available annotations to investigate how the clusters relate to demography. To accomplish this, we identify features that distinguish members of the cluster, then we deduce a likely demographic scenario from these distinguishing features. For this analysis, we rely on two sets of features: (1) admixture proportions in 20 global populations estimated from the genotypes; and (2) ancestral birth dates and locations from pedigrees associated with some genotyped individuals. To simplify the presentation of admixture summary statistics, some population labels used to define the summary statistics are taken as combinations of ancestral populations; for example, we define ‘Europe West' as Ireland, Great Britain, Scandinavia, and the region containing Germany and France.

We generate birth location maps by converting each birth location, within a specified range of generations, to the nearest coordinate on a two-dimensional grid, with grid points every 0.5° of latitude and longitude. Then, we count the number of birth locations at each grid point. The location of each grid point plotted on the map is the mean latitude and longitude over all the annotations assigned to that grid point. By scaling the area of each grid point by the number of birth annotations at that location, the maps yield population density estimates, and highlight large urban areas, at different time periods in the United States and Europe. All maps are produced in the same way, differing only in the granularity of the grid and the scale of the plotted points. The distribution of ancestral birth locations by generation recapitulate broad population trends in the United States and in Europe, such as increasing concentration in urban areas over time, increases in population density west of the Mississippi River reflecting westward expansion of European settlement, and migration trends from Europe to the United States (Supplementary Figs 16,17).

To discover geographic features characteristic of a given cluster, we compile statistics from genealogical data specific to each cluster. Specifically, we compute, for each grid point, the odds ratio (OR) for a given cluster—the odds that the grid point is associated with a cluster member over the odds that the grid point is associated with a non-member—then we visualize the distribution of map locations with the largest odds ratios. One rationale for using the OR statistic is that it is informative of cluster prediction accuracy; if we label all map locations with OR>*x* as ‘ground-truth cluster locations', then cluster assignments will yield a higher rate of true positives (recovered ground-truth cluster locations) for larger *x* (assuming the map location frequency remains the same). To highlight the geographic concentration of individual clusters in Fig. 4, we plot only locations satisfying OR>*x*, with *x* chosen separately for each cluster. All plotted map locations require a minimum of 10 birth locations associated with cluster members. In some cases, the geographic concentration of birth locations becomes more apparent when the OR calculations are restricted to certain pedigree generations; for example, the birth locations of ancestors 0–5 generations ago associated with the Utah cluster are more highly concentrated in Utah, and other ancestral generations are more dispersed across the eastern United States (Supplementary Fig. 24). Although this strategy is useful for characterizing most clusters, in the Supplementary Discussion we point out some limitations in using the genealogical data to interpret the clusters. For example, our ability to accurately infer demographic trends depends on the composition of the AncestryDNA database, and the availability of genealogical records. Also, note that ORs are more relevant than evidence for enrichment (for example, *P* value); for example, consider that we would often expect strong evidence for enrichment in large US cities even when the OR is only slightly >1 (for example, association of Chicago with African American cluster), but these locations would provide relatively little information for interpreting the cluster given that many other groups have typically settled in large US cities.

### Genetic differentiation between clusters

We calculate pairwise F_*ST*_ to assess genetic differentiation in common variation between clusters. We include 611,560 SNPs on autosomal chromosomes in the F_*ST*_ calculations, a subset of the 633,299 SNPs used in phasing that have genotype call rate >95% in the sample. We use the ratio-of-averages formula that also adjusts for differences in sample size^63^.

### Data availability

The HGDP^58^,^59^ genotype samples included in the ancestry reference panel were obtained from the HGDP website ( http://www.hagsc.org/hgdp). 1000 Genomes Project Phase 3 (ref. 3333) genotype samples that were used for validation of admixture estimates and the spectral analysis were downloaded from the NCBI FTP site ( ftp://ftp-trace.ncbi.nih.gov/1000genomes). For the purpose of ensuring reproducibility, we will share the IBD network topology, edge weights, and cluster labels on request and subject to relevant data use policies. Although we cannot make the genealogical and genotype data widely available to the academic community in light of our commitment to our customers, we are interested to pursue research collaboration opportunities. Please contact C.A.B. ( cball@ancestry.com) for guidelines on submitting a research proposal.

## Additional information

**How to cite this article:** Han, E. *et al*. Clustering of 770,000 genomes reveals post-colonial population structure of North america. *Nat. Commun.* **8,** 14238 doi: 10.1038/ncomms14238 (2017).

**Publisher's note:** Springer Nature remains neutral with regard to jurisdictional claims in published maps and institutional affiliations.

## Supplementary Material

Supplementary Information Supplementary Figures, Supplementary Tables, Supplementary Discussion, Supplementary Methods, and Supplementary References

Supplementary Dataset 1 Detailed summary of clusters and stable subsets identified from community detection and spectral analysis. Table includes all network and sub-network clusters with at least 2,000 members, and all stable subsets identified in the spectral analysis (aside from small subsets with much fewer than 100 members). The table columns are divided into three sets: (1) top-level clusters detected in the full IBD network; (2) level-two clusters identified in the sub-networks; and (3) stable subsets of clusters identified in the spectral analysis. Individual columns are as follows: "cluster label" is the name assigned to the cluster based on statistical summaries of the admixture and genealogical data; "DNA samples" is the number of genotyped individuals assigned to the cluster; "pedigree nodes" is the total number of pedigree nodes with birth location annotations linked to cluster members; "average size of pedigree" gives the average number of ancestral birth location annotations per cluster member; Win and Wout summarize within-cluster (Win) and cross-cluster (Wout) network "density", defined as (σew[e])/(σe1), in which the sums are over all edges e(i, j) between cluster members (Win), and between cluster members and non-members (Wout); in the "admixture proportions" column, admixture statistics are expressed as d > x, q% (p%), meaning that q% individuals assigned to the cluster and p% outside the cluster have estimated admixture proportions for ancestral population d greater than x (an NA indicates that no simple summary statistics usefully capture admixture of the cluster members). The final set of columns give additional details about the stable subsets identified in the spectral analysis: "eigenvectors" is the pair of eigenvectors of the Laplacian or subgraph Laplacian (†) used to determine the stable subset; "cluster level"—the cluster assignment that is most correlated with the stable subset assignment is found at this level; "false negatives" gives the number of samples assigned to the cluster based on community detection that are not selected based on the linear decision rule; "false positives" gives the number of samples that are selected based on the linear decision rule, but are not assigned to the same cluster in the community detection (note that these false positives are not included in the stable subset, and are only used to assess the quality of the decision rule); "FP rate" gives the proportion of selected samples that are not assigned to the same cluster; "decision rule" defines the selection criterion for the stable subset, in which variables Y1 and Y2 are the projection of the sample onto the 2-d embedding specified by the selected eigenvectors, after applying the specified rotation (positive rotations are counterclockwise). To compile the admixture statistics, we define the population labels (d) from the 20 ancestral populations (Supplementary Table 4) as follows: "Europe South" combines both the Iberian Peninsula and the region containing Italy and Greece; "Europe West" is the population that is attributed to the majority of genotyped individuals, and combines Ireland, Great Britain, Scandinavia, and the region that contains Germany and France; all other population labels are the same as those defined for the global ancestry reference panel (Supplementary Table 2). *Stable subset of toplevel etected in IBD network. *Dominicans stable subset is contained within Caribbeans stable subset. **Cluster is not subdivided further. ‡Interpretation of cluster is uncertain; label represents best guess. Native Am. = Native American.

Supplementary Dataset 2 Detailed summary of clusters and stable subsets identified from

Supplementary Dataset 3 Validation of admixture estimates in HGDP and 1000 Genomes samples. The 26 columns labeled "Africa Southern Bantu" through "Finland and Northwest Russia" summarize estimated admixture proportions for each of the 26 defined ancestral populations based on the global ancestry reference panel; specifically, each entry gives the median admixture proportion, with 5th and 95th percentiles in square brackets, among all genotype samples assigned a given population label. Entries are highlighted in bold whenever the median admixture proportion is greater than 10%. The "number of samples" column gives the number of genotype samples assigned a given population label. This table includes admixture estimates for 1,043 HGDP genotype samples2 (of which 855 are also included in the global ancestry reference panel) and 1,816 "unrelated" samples genotyped on the Illumina OMNI 2.5M genotyping chip platform as part of Phase 3 of the 1000 Genomes Project3. (By "unrelated," we mean one sample is selected from each duo, and both parents are selected from trios.) To estimate admixture proportions in the 855 samples that are also included in the ancestry reference panel, we run 20-fold cross-validation; that is, we estimate admixture proportions by running ADMIXTURE separately on 20 different data sets, in which approximately 5% of the HGDP and 1000 Genomes samples are included in each of the 20 in this analysis have substantial contributions (>25%) from the Polynesia, Mali and European. The 1000 Genomes labels are abbreviations for the following populations: ACB = African Caribbean in Barbados; ASW = People with African Ancestry in Southwest USA; CDX = Chinese Dai in Xishuangbanna, China; CEU = Utah residents (CEPH) with Northern and Western European ancestry; CHB = Han Chinese in Beijing, China; CHD = Han Chinese from Denver, Colorado; CHS = Southern Han Chinese; CLM = Colombians in Medellin, Colombia; FIN = Finnish in Finland; GIH = Gujarati Indians in Houston, TX, USA; GBR = British in England and Scotland; IBS = Iberian Populations in Spain; JPT = Japanese in Tokyo, Japan; KHV = Kinh in Ho Chi Minh City, Vietnam; LWK = Luhya in Webuye, Kenya; MXL = People with Mexican Ancestry in Los Angeles, CA, USA; PEL = Peruvians in Lima, Peru; PUR = Puerto Ricans in Puerto Rico; TSI = Toscani in Italia; YRI = Yoruba in Ibadan, Nigeria.

## Figures and Tables

**Table 1:** Summary of IBD network clustering.

**Cluster**	**Samples**	**Birth location**	**1000**	**Global admixture proportions**		
		**Annotations**	**Genomes**	**Admixture Statistic**	**In (%)**	**Out (%)**
African Americans*	44,966	172,087	ASW, ACB	West Africa>0.5	95	1.2
European Jewish*	26,547	261,655	—	Jewish>0.1	99	0.4
Irish*	20,747	222,198	—	Celtic>0.25	93	21
French Canadians*	9,689	363,916	—	West+South Europe>0.75	88	72
Acadians*	6,615	204,131	—	West+South Europe>0.75	83	72
Scandinavians*	4,189	97,496	—	Scandinavia>0.2	96	14
Portuguese, including Azores and Madeira Is.†‡	3,468	32,703	—	Iberian>0.1	66	12
Finnish*	1,687	29,850	FIN	Finland and NW Russia>0.2	97	0.2
Hawaiians*	583	4,715	—	Polynesia>0.2, Asia East>0.2	94, 35	0.1, 0.7
Northeast Mexico*	6,311	61,391	—	Iberian>0.1 and Nat. Am.>0.1	81	3
New Mexicans*	5,291	65,236	—	Iberian>0.1 and Nat. Am.>0.1	79	4
West Mexico*	1,356	5,924	MXL	Iberian>0.1 and Nat. Am.>0.1	90	4
Caribbeans*	9,315	73,274	PUR	Iberian>0.1	84	11
Dominicans*§	779	1,698	—	Iberian>0.1 and W. Africa>0.1	80	1
Central Americans*‖	1,407	6,971	—	Iberian>0.1 and Nat. Am.>0.1	80	4
Colombians*‖	710	3,261	CLM	Iberian>0.1 and Nat. Am.>0.1	88	4
Lower Midwest and Appalachians†	108,786	4,131,104	—	West Europe>0.75	87	56
Northeast and Utah†	99,315	4,088,040	—	West Europe>0.75	80	57
Upland South†	93,305	3,341,813	—	West Europe>0.75	88	56
Pennsylvania†	80,754	2,370,273	—	West Europe>0.75	71	59
Lower South†	77,581	2,608,314	—	West Europe>0.75	88	57
Utah¶	4,519	283,911	CEU	West Europe>0.75	96	61
Mennonites†‡	2,139	52,216	—	West Europe>0.75	70	60
Appalachians¶	2,048	87,725	—	West Europe>0.75	99	61
Amish¶	1,067	42,903	—	West Europe>0.5	94	74

ACB, African Caribbean in Barbados; ASW, people with African Ancestry in Southwest USA; CEU, Utah residents with Northern and Western European ancestry; CLM, Colombians from Medellin, Colombia; FIN, Finnish in Finland; MXL, Mexican ancestry from Los Angeles; Nat. Am., Native American; PUR, Puerto Rican.

Rows are grouped to coincide with the discussion. Admixture statistics are expressed as *d*>*x*, *P*_in_%, *P*_out_*%*, meaning that *P*_in_% individuals assigned to the cluster and *P*_out_% outside the cluster have estimated admixture proportions for ancestral population *d*>*x*. The ‘1000 Genomes' column summarizes population labels of any 1000 Genomes samples that project onto the same stable subsets defined in spectral embedding (Fig. 6; Supplementary Figs 4,5). See Supplementary Data 2 for a more detailed summary of the clustering results, including definition of the population labels (*d*).

^*^A stable subset including more than 5% of a hierarchical cluster.

^†^A hierarchical cluster in which no detected stable subset includes more than 5% of the cluster.

^‡^Interpretation of cluster is uncertain; label represents our best estimate.

^§^Dominicans cluster is contained within Caribbean cluster.

^‖^Not shown in Fig. 3.

^¶^A stable subset accounting for less than 5% of a hierarchical cluster.
