# Supplementary material for: Clustering of 770,000 genomes reveals post-colonial population structure of North America
Source: Nat Commun. 2017 Feb 7;8:14238. doi: 10.1038/ncomms14238 (PMC5309710; doi:10.1038/ncomms14238)
Supplement: Supplementary Information — Supplementary Figures, Supplementary Tables, Supplementary Discussion, Supplementary Methods, and Supplementary References [file ncomms14238-s1.pdf]

Supplementary Discussion

Projection of US states based on IBD

Here we discuss results of an analysis of IBD aggregated across US states (see Supplementary Methods). US states sharing high levels of IBD on average are positioned close to each other in a projection onto the first two principal components (PCs), evoking a distinct relationship to US geography arising from isolation-by-distance (Fig. 1): PC 1 correlates with a North-South geographic axis, and PC 2 correlates with an East-West geographic axis. The North-South separation is stronger for eastern states, as one might expect given recent mass European settlement[7, 8]. An exception is Louisiana, positioned roughly equidistant between Northern and Southern states in Fig. 1**;** this likely reflects the history of 17th century French colonists in Acadia (current day Atlantic Canada and Maine), who were expelled during the French and Indian War, and some of whom later resettled in the Spanish colony of *Luisiana*, now Louisiana[9]. Results from IBD network clustering further support this finding (see the main text). This observation suggested that the signature of a recent historical migration within North America could in fact be identified by examining patterns of IBD among present-day Americans.

Genetic differentiation between clusters

To assess the connection between IBD clustering and global populations, we measured differentiation in common genetic variation (F_{ST}) between the 6 largest clusters detected in the IBD network (Supplementary Table 8), clusters detected in the sub-networks (Supplementary Table 5), and the clusters ("stable subsets") identified in the spectral analysis (Supplementary Table 4). F_{ST} between Jewish, Irish, Scandinavian, Finnish, Hawaiian and African American stable subsets (Supplementary Table 4) closely matches F_{ST} estimated from comparable worldwide populations. For example, compare F_{ST} = 0.078–0.081 between African American and European-origin stable subsets (Irish, Scandinavian, Finnish) to European–African F_{ST} of 0.096[10]. Also, F_{ST} = 0.005–0.007 between Jewish and European-origin stable subsets are similar to F_{ST} estimates of 0.008–0.011 between Italian/Ashkenazi Jews and French/North Italians[11]. Our F_{ST} estimates are typically slightly lower than others'; this is expected because our samples were not recruited to specifically capture genetic variation of ancestral populations, as in 1000 Genomes[13] or People of the British Isles[14], and the clusters likely contain more admixed individuals than the comparable cohorts. Here, we use F_{ST} calculated from the stable subsets because they appear to be more representative of global populations than the hierarchical clustering; Jewish and African Americans are striking examples of this (Supplementary Data 2). This illustrates that the hierarchical clustering, as opposed to the stable subsets, contain individuals who may not neatly "fit" within a single subgroup, and are potentially more admixed than the individuals within the stable subsets.

Relationship between spectral analysis of IBD and frequency-based admixture

In addition to F_{ST}, comparing distances in the spectral embedding to admixture proportions estimated from genotype data demonstrates a relationship between IBD and global population

structure (Supplementary Fig. 22). Since most samples lie near the origin in any given dimension of the spectral embedding, and since Euclidean distance in the embedding is proportional to the diffusion distance[15], it follows that samples further away from the origin are the most disconnected, or "isolated", in the network. Interestingly, this degree of isolation in the network is often strongly correlated with the amount of admixture. For example, we observe a very strong correlation (r^2 = 0.97) between European Jewish admixture proportions and spectral embedding distance in the Jewish stable subset; in particular, single-origin European Jewish tend to be the most disconnected nodes in the network. Similarly, the Hawaiian cluster demonstrates a strong relationship between the degree of network isolation and the amount of Polynesian ancestry (r^2 = 0.80). Although the correlation is not nearly as strong for Finnish (r^2 = 0.67) and African Americans (r^2 = 0.26), it is nonetheless interesting that the most isolated cluster members tend to be the least admixed individuals.

Discussion on interpretation of clusters using genealogical data

Although the approach we have described is useful for characterizing most clusters, here we discuss some of the limitations we encountered in using the genealogical data to interpret the clusters. Perhaps most obviously, groups that do not show a particularly unusual concentration of ancestral birth locations in any regions will be difficult to interpret from these data alone. The most conspicuous example of this is the Jewish cluster; most areas of the US and Europe featuring large past or present Jewish communities are also ancestral locations of other large ethnic groups that immigrated to the US. In this case, the estimated admixture proportions allow for a clear interpretation of the Jewish cluster, but these data did not allow for an unambiguous interpretation of the three Jewish sub-network clusters, labeled "European Jewish A, B and C" (Supplementary Data 2). Several other groups do not exhibit a particularly unusual geographic concentration in the US, including Irish, Colombians and Caribbeans, because they have primarily migrated to areas inhabited by many other groups. For similar reasons, large cities such as Chicago, Detroit and New York are important ancestral birth locations for several groups (e.g., African Americans), but do not feature prominently in the maps because these cities are also attributed to other clusters.

Another consideration is that the extent to which these data reflect US-wide trends hinges on the composition of the AncestryDNA database, and the availability of genealogical records. Clearly, this database captures much of the genetic diversity of the US, but in Supplementary Fig. 17, for example, we might expect a greater density of ancestors from Poland given that it is one of the largest ethnic groups in the US. This discrepancy could be partly attributed to lower availability of Polish genealogical records, and a predisposition in the AncestryDNA database toward individuals with Western European origins.

Additional historical details on selected clusters, including demographic trends recapitulated by genealogical data

In this section, we provide additional details about clusters described in the main text, as well as clusters mentioned in the results but not discussed in any detail in the main text. Here, we also

provide descriptions of several additional clusters identified in the third level of the hierarchical clustering. We do not comprehensively discuss all of these clusters due to their more speculative nature (see main text). Below, we specify the level of hierarchical clustering in which the cluster was identified (first, second, or third), and whether the cluster was identified from the spectral analysis ("stable subset"). To be consistent with the main text, we divide our discussion of these clusters into the same four main categories used in the main text—intact immigrant clusters, continental admixed groups, assimilated immigrant groups, and post-migration isolated groups—with the caveat that these distinctions are somewhat arbitrary and meant only to aid in understanding the clusters identified.

Intact immigrant clusters

African Americans. Our interpretation of this cluster, identified in both the clustering of the sub-networks (second level of hierarchical clustering) and the spectral analysis, is strongly supported by the distribution of global admixture proportions (Supplementary Figs. 10, 11). The distribution of ancestral birth locations in this cluster closely traces the westward expansion of cotton cultivation and slavery, originating in the rich coastal plains of North and South Carolina, then progressing west until it reaches eastern Texas (Supplementary Figs. 19, 20). It also closely coincides with regions of high self-reported African ancestry[16] and regions that historically practiced slavery, suggesting some amount of continued isolation. We note that this cluster does not specifically exclude African Americans in the North; the apparent absence of northern US cities such as Detroit and Milwaukee from Fig. 3 is attributed to the fact that these cities also include significant contributions from other clusters as well ($OR < 5$ for these cities). In the third level of the hierarchical clustering, we find additional substructure in the African American cluster corresponding to geography in the southern US; however, we do not detail these results here.

European Jewish. One of the largest clusters initially identified in the IBD network (first level of hierarchical clustering) is the European Jewish cluster, which was also identified as a cluster in the spectral analysis. In the ancestral birth location maps for this cluster (Supplementary Figs. 19, 20), we do not find a large over-representation of ancestral birth locations at specific locations within the US. The strongest over-representation of Jewish birth locations is in or near New York and Chicago ($OR > 2$); these cities received large numbers of Jewish immigrants during the late 1800’s and early 1900’s. We note that this cluster is subdivided into 3 clusters in the second level of the hierarchical clustering, and the historical significance of these 3 clusters is unclear ("European Jewish A, B and C" in Supplementary Data 2). We are unable to clearly distinguish between these 3 groups based on the genealogical data, perhaps owing to the fact that the ancestral Jewish communities in Europe are co-located with other European ethnic groups, and therefore locations with high odds ratios do not consistently pinpoint the locations in Europe most relevant to these clusters.

*Portuguese***.** In the second-level of the hierarchical clustering, we identify a cluster corresponding to the Portuguese (Table 1, Supplementary Data 2, Supplementary Fig. 19). The Portuguese began to immigrate to the US in large numbers in the late 19th century; immigrants

were primarily men from the Azores and Madeira Islands, recruited to work on whaling ships[17]. These men emigrated to the eastern US, establishing communities in various New England coastal cities (major regions include Providence, Bristol, Pawtucket in Rhode Island, and New Bedford, Taunton, Fall River in Southeastern Massachusetts); we do not find strong enrichment of ancestral birth locations of Portuguese cluster members in these regions, likely due to the influx of other groups to these regions. The Portuguese also immigrated to various cities in California, including the San Francisco-Oakland Bay Area, Santa Cruz, the Central Valley, and San Diego, and this is reflected in Fig. 3. In the mid-to-late 20th century, there was another documented surge of Portuguese immigration in America, mainly in the Northeast (New Jersey, New York, Connecticut, Rhode Island, Massachusetts). We have no good explanation for the inclusion of individuals with Jamaican ancestral birth locations in this cluster. This finding is probably a result of having a small number of samples of Jamaican origin that were arbitrarily grouped with Portuguese to form a slightly larger cluster, and illustrates the lack of robustness of IBD clustering with small sample sizes.

Eastern Europeans. In the second level of the hierarchical clustering, we identify a cluster we call “Midwest immigrants” (Supplementary Data 2). Although we do not specifically discuss this cluster in the main text, we do discuss two of its stable subsets, Scandinavians and Finnish. This cluster is subdivided into additional large clusters in the third level of the hierarchical clustering (Supplementary Figs. 25, 26). Some of these clusters clearly relate to geographic structure and immigration patterns from Eastern Europe. For example, the ancestral birth location patterns of the "Eastern Europeans and Italians in Pennsylvania and Midwest" cluster (Supplementary Fig. 26) might correspond to the migration of nearly 24 million southern and eastern Europeans to the US between 1880 and 1920, before the restriction of immigration in 1924[18]. Immigrants predominantly came from Hungary, Poland, Austria, Slovakia, Czech Republic and Italy between 1840 and 1870, with New York state and Pennsylvania the primary destinations for these immigrants. In 1924, the US Congress passed the Immigration Act, effectively cutting off immigration from southern and eastern Europe and giving preference to European countries in the north and west[18]. Additionally, we identify a cluster corresponding to “German, Dutch and Eastern Europeans in Upper Midwest” (Supplementary Fig. 26), in which European ancestral birth locations are located further north, and found in the US primarily in the Midwest. Finally, we identify another cluster corresponding to “Croats, Albanians, Greeks and Turkish” (Supplementary Fig. 26). As we discuss in the main text, the identification of these clusters likely corresponds to pre-migration population structure; however, localized immigration of these ethnic groups to the US, particularly to certain regions of the Midwest, seems to have also contributed to the structure we identify in the IBD network.

Northern Europe (Finnish, Swedish, Danish, Norwegians and Scandinavians). Additional third-level clustering of the "Midwest immigrants" cluster relates to regions in Northern Europe. A Finnish cluster was identified in the spectral analysis (Table 1, Supplementary Figs. 11, 19), and in the third level of hierarchical clustering (Supplementary Figs. 25, 26). Ancestral birth locations in the Finnish cluster and stable subset coincide with their historical record of migration to the US, and in particular to the Michigan Upper Peninsula[19]. We also identify a stable subset in the Midwest immigrants cluster which we label as Scandinavians (Table 1, Fig. 3). Ancestral birth

locations in this cluster closely correspond to the settlement pattern of Norwegians in rural Minnesota, North Dakota and Wisconsin, with large numbers moving to Minneapolis and Chicago[19, 20] (Supplementary Fig. 20). The subdivision of the Midwest immigrants cluster also delineates two clusters, one corresponding to Swedish and Danish, and the other to Norwegians (Supplementary Fig. 26).

Irish. In the spectral analysis, we identify a cluster which likely corresponds to descendants of Irish immigrants. Six million Irish settled in the US in the 19th century, with immigration peaking in 1852 during the Irish famine[21]. Irish migration was historically characterized by a highly localized pattern of chain migration, as migrants followed family members and neighbors to the same towns and cities in the U.S.[21]. In fact, almost half (46%) of Irish immigrants migrated to just 10 U.S. counties[22]. Once in the US, the Irish may have tended to marry within their ethnic group since their migration was relatively gender-balanced compared to other European groups, which were male-dominated. Many young Irish women emigrated to the U.S. in order to find a spouse, have families, and achieve economic independence, as the famine reduced and delayed opportunities for marriage in Ireland for decades[23]. Interestingly, in the third level of the hierarchical clustering, we identify 3 clusters corresponding to North, South and West geography within Ireland (Supplementary Fig. 26).

Other European immigrant groups. In addition to the results already discussed, we identify substructure corresponding to other European immigrant groups when we subdivide the "Italians, Irish and Scottish" cluster (Supplementary Data 2, Fig. 26). For example, we identify a cluster whose ancestral birth locations are disproportionately concentrated in Scotland, Atlantic Canada and Ontario (Supplementary Fig. 26), corresponding to migration of large numbers of Scottish to Canada. Furthermore, we also find an Italian cluster (Supplementary Fig. 26). The ancestral birth locations for the Italian cluster are particularly concentrated in southern Italy, reflecting the predominant source of Italian immigration to the US.

Polynesians, East Asians and Hawaiians. In the first level of hierarchical clustering, we identify a cluster corresponding to Polynesians and East Asians (Supplementary Data 2) with only a small number of individuals, and thus we do not subdivided it further. The Hawaiian stable subset identified in this cluster is representative of the Polynesian population with some East Asian admixture. Reflecting this finding, Hawaiians have likely remained genetically isolated due to the large distance from the continental US, while Hawaii has a complicated history with recent, abrupt population changes and rapid growth[24].

Continental admixed groups

Mexico clusters. In the main text, we discuss the connection between the Northeast and West Mexico clusters and Mexico-US migration patterns. We further note that areas that have not traditionally seen a large influx of migrants to the US, such as southern Mexican states, are poorly represented in the Mexican clusters (Supplementary Figs. 17, 18), reflecting our US-biased sample.

New Mexicans. We identify a cluster corresponding to New Mexicans in both the clustering of sub-networks as well as in the spectral analysis (Fig. 3, Table 1, Supplementary Data 2). This cluster most likely represents descendants of the *Nuevomexicanos*, some of the earliest European colonial settlers that migrated northward from New Spain along the *El Camino Real de Tierra Adentro* trail[25]. Supporting this hypothesis, ancestral birth locations are disproportionately found in parts of Mexico and New Mexico near this trail (Supplementary Fig. 20).

Puerto Ricans. In our discussion in the main text, although we do not single out Puerto Ricans from other Caribbean Islands peoples, Puerto Ricans are by far the predominant Caribbean group in our sample. Puerto Ricans typically have a mixture of European and African ancestry, with smaller amounts of Native American admixture[26], and this is reflected in our data (Supplementary Figs. 9–11, Supplementary Data 2). In the second level of the hierarchical clustering, we identify fine-scale structure on the island of Puerto Rico in the 3 largest clusters of the Caribbean sub-network (Supplementary Data 2). These 3 clusters are clearly correlated with geography of the island of Puerto Rico, as they roughly subdivide the island into 3 regions—northwest, southwest and east (Supplementary Fig. 21). These 3 Puerto Rican clusters show small differences in Native American, West African and European admixture proportions (Supplementary Fig. 11) that only partially reproduce the findings of genetic variation across the island of Puerto Rico[26], perhaps due to differences in the composition of our database and their sample. Interestingly, the Puerto Rican cluster has an enrichment of ancestral birth locations on the island of Hawaii. This likely reflects the arrival of sugar cane plantation laborers to Hawaii in the early 1900's; Puerto Ricans have been documented as a separate ethnic group in Hawaii as early as 1910, and they have constituted over 2% of the population up until 1950[27].

Assimilated immigrant groups

Pennsylvania. In the second-level hierarchical clustering, we identify a cluster with birth locations concentrated in Pennsylvania (Table 1, Fig. 3, Supplementary Fig. 19). Roughly 80,000 Germans immigrated to the colonies in North America between 1717 and 1775, with the majority settling in Philadelphia, southeast Pennsylvania, and New Jersey. By 1760, 50,000 Germans had settled in southeast Pennsylvania alone. At the time, Germans constituted the largest ethnic group in the colony with a distinct language, religious culture, and identity[28]. German immigrants tended to marry within their ethnic group and remained geographically stable for many generations[28]. While this demography may not encompass the entire history of the Pennsylvania cluster, it provides some background for the genealogical data associated with individuals assigned to this cluster.

Southern US: Alabama, and North and South Carolina. Here, we discuss further substructure identified in third-level hierarchical clustering of the "Lower South" cluster (Supplementary Figs. 25, 27). The predominant migration pattern from North and South Carolina to Alabama observed in the genealogical data of the "Alabama, and North and South Carolina" cluster (Supplementary Fig. 27) may be explained by the westward expansion of the cotton industry between 1820 and 1860. "Alabama fever" gripped South Carolinians after the opening of the

territory to European settlement following the expulsion of the Creek people in 1814[29]. Since soil quality had declined in both South Carolina and Georgia by 1820[30], migrants passed through Georgia and moved directly into Alabama, where the nutrient rich soil yielded 3 times more cotton per acre[31]. By 1850, there were an estimated 45,000 migrants from South Carolina in Alabama[31], accounting for approximately 30% of all incoming migrants[30]. South Carolina migration to Alabama began to decrease in 1860 due to the opening of new migration routes further west into Texas, Arkansas, and Florida[30].

Southern US: Florida, Georgia, and South Carolina. Another cluster identified from third-level hierarchical clustering of the "Lower South" cluster has enriched ancestral birth locations in Florida, Georgia, and South Carolina (Supplementary Figs. 25, 27). The southward movement of people in Georgia and South Carolina (Supplementary Fig. 27) is also consistent with documented historical migration patterns. Settlers moved into the rich coastal plains of Georgia and South Carolina—the best agricultural land in the South—between 1780 and 1810[32]. Then, between 1825 and 1840, migrants poured into “middle Florida” (near present-day Tallahassee) prompted by the acquisition of the Florida territory from Spain in 1821, and the removal of native Seminole, Miccosukee and Red Stick Creek people in 1824[33]. The end of the in-migration to Florida coincided with the collapse of cotton prices in 1840[33].

Southern US: additional substructure. In the third-level hierarchical clustering of the Lower South and Upland South clusters (Supplementary Figs. 25, 27), we identify further fine-scale structure that corresponds in part to geography in the Southern US, and possibly other historical migration patterns. Again, we emphasize that the demographic interpretation of many third-level clusters is more speculative.

Post-migration isolated groups

Appalachians. In the main text, we discuss the Appalachians stable subset. The Appalachians cluster is particularly concentrated in southeastern Kentucky near the Cumberland Mountains, which was more geographically and economically isolated relative to other parts of Appalachia. Moore[34] claims that the problematic stereotype of Appalachia as remote and homogenous is based on this particular sub-region. The coal industry developed first in northern and western Kentucky, then only gradually moved into the southeastern part of the state. Railroads did not reach eastern Kentucky until the 1880s, and only came to Harlan County, where a dense number of ancestral birth locations are concentrated (Supplementary Fig. 20), during World War I[34, 35]. This history, as discussed in the main text, provides an explanation for the identification of this cluster in the IBD network.

Mennonites. We identify what we hypothesize to be a sub-network cluster corresponding to Mennonites, although this label is less clear than the others. Mennonite families homesteaded in different parts of the Great Plains; districts with concentrated Mennonite settlement in the include Marion, McPherson, Harvey, Butler and Reno counties in Kansas[36], and near Korn (later Corn), Fairview, North Enid, and in the Meno-Ringwood-Goltry in Oklahoma[37]. In

Supplementary Fig. 19, we observe that many enriched ancestral birth locations of this cluster occur at or near these settlements.

Utah. Here we include some additional details about the Utah stable subset that we did not have room to discuss in the main text. Using genealogical annotations, we are able to trace the migration patterns of this cluster with remarkable detail (Supplementary Fig. 24). Large numbers of Scandinavians migrated to the Northeast in the 1700's, descendants of whom later moved to Utah; this is well captured by the large concentration of ancestral birth locations for the Utah cluster in Scandinavia, especially Denmark. Areas in the west outside Utah (Mexico, Arizona and British Columbia) also appear as over-represented ancestral birth locations in this cluster, and correspond to known Mormon settlements. Over-represented ancestral birth locations in and near Iowa correspond to important settlements along the Mormon trail (Nauvoo, Illinois and Omaha, Nebraska).

Discussion of IBD network analysis

We sometimes find that the clusters identified by recursively maximizing the modularity closely overlap with the stable subsets (examples include European Jewish and New Mexicans; see Supplementary Data 2). In such cases, the spectral analysis provides additional support for the clusters, and narrows the range of likely demographic hypotheses underlying the hierarchical clustering. Since the spectral analysis only delineates the most disconnected subgraphs, an additional benefit is that it filters out "admixed" individuals that might be arbitrarily assigned to clusters—e.g., a child of parents from two genetically isolated populations. This explains why stable subsets are more representative of the global ancestral populations than their corresponding clusters (compare, in Supplementary Data 2 and Supplementary Figs. 9, 10, the admixture proportions in the clusters and stable subsets corresponding to African Americans and European Jewish).

In other cases, the stable subset contains only a small fraction of the cluster, or no stable subset is identified in the cluster (Table 1, Supplementary Data 2). Such clusters are typically much less modular than the clusters that closely overlap stable subsets; this can be seen by comparing the internal edge density of the cluster (W_{in}) to the density of edges to non-cluster members (W_{out}). Although any population structure underlying this clustering is therefore subtler, we find that the clustering in several cases corresponds to unambiguous demographic patterns. In this circumstance, the likely interpretation of the hierarchical clustering is that it is the discretization of some unknown, continuous feature of IBD variation (*e.g.,* geographic distance). That being said, we often find that this network structure is often more difficult to interpret, and the exact boundaries of the clusters may be partly influenced by synthetic factors, such as the frequent failure of modularity-maximizing methods to partition small modules[38–40].

Laplacian eigenmaps and related spectral clustering methods have been previously proposed for inferring population structure from genetic data, and there are several published works on this topic[41–43]. The most closely related work is by Lee *et al.*[41,42]; they use spectral dimensionality reduction methods, as we do here, to uncover population structure in the

POPRES data set. The key differences are: (1) we define similarity between data points using pairwise IBD estimates, whereas they take a dot product of the genotypes; (2) we uncover population structure at much finer scale; and (3) we apply spectral methods to data on a much larger scale.

Also, we are not the first to combine hierarchical clustering with spectral methods, and it is possible that other algorithms could provide a more systematic implementation of our approach to identifying modular structure informative of population demography in the IBD network; see, for example, the *HQcut* method developed by Ruan and Zhang[44]. However, an important consideration is the massive scale of our network, which prohibits the application of this method and other more computationally complex algorithms developed for community detection. Additionally, visualizing the individual dimensions of the spectral embedding generated from the IBD network yields additional information about structure in the population (Supplementary Fig. 22).

An unresolved issue common to both the hierarchical clustering and spectral analysis is that the stopping criterion is unclear: in the hierarchical clustering, there is the question of when to stop subdividing clusters; in the spectral analysis, there is the question how many dimensions of the spectral embedding to use to delineate stable subsets. Although researchers have proposed stopping criteria for these methods (e.g., [39, 45]), our experience as well as the unique properties of the IBD network suggest that these criteria either do not apply or do not work well in our setting. In particular, the question of assessing statistical significance in detected modules has received considerable attention in the literature; see Berry *et al.*[38] and Fortunato[39] for some recent reviews of the topic. However, this unresolved question is even further complicated by the difficulty of determining an appropriate "null model" for a network reflecting IBD in a modern-day human populations. As a result, we have not attempted to make any claims about statistical significance of clusters detected in the IBD network. For the hierarchical clustering, we have used an *ad hoc* stopping criterion based on the size of the cluster (see above), and this is an aspect we hope to address more systematically in future work.

An open question in the spectral analysis is whether the order in which stable subsets are discovered in the spectral embedding yields an approximate ranking of how closely the clusters resemble disconnected components, and therefore provides a measure of genetic isolation. Anecdotally, there is evidence for this interpretation—Jewish and Hispanic/Latino groups cluster in the first dimensions of the spectral embedding, whereas the last stable subsets we identify correspond to Utah settlers and Irish immigrants which show little genetic differentiation from most European-origin individuals in the US. However, except in idealized settings[45,46], there is no theory supporting the ordering of the eigenvectors of the Laplacian to rank the clusters according to the "isolation" in the network.

Finally, we consider a general limitation to the proposed approach: the structure of the IBD network hinges on parameter choices and assumptions made. For example, while the edge weights are defined from simulations intended to reflect real biological relationships (as described), they do not account for factors such as population-specific excess IBD sharing (e.g.,

due to founder events or rapid population growth). As a result, network structure corresponding to subpopulations exhibiting sharp deviations from random mating may reflect more distant demography compared to subpopulations that are more closely modeled by our simulations. A model-based approach that adjusts the demographic model parameters to fit the IBD data could potentially address this limitation. Recent work has demonstrated the power of coalescent model-based methods to infer population demographic parameters from IBD[47–49]. The distribution of detected IBD in each of the clusters could be used to reconstruct more detailed histories of the underlying historical groups. However, for *discovering* population structure, an important yet unresolved question is how to develop a model-based method that can jointly learn *both* the population parameters and population assignments. In our model-free approach, we take the pragmatic view that sensitivity of the inferred network structure to assumptions could be a feature of our method—that is, in future work, alternative choices could reveal population structure arising from different time-scales.

Supplementary Methods

Sample collection

All DNA samples included in this study were collected from AncestryDNA customers (except for samples obtained from external sample collections that are included only in the admixture reference panel—see below). The typical sample collection process for an AncestryDNA customer is as follows: a customer orders an AncestryDNA kit through the AncestryDNA website; the customer collects saliva at home, and returns the saliva sample in stabilizing solution; DNA from the saliva is extracted; finally, genotypes are called for a dense panel of single nucleotide polymorphisms (SNPs) across the genome.

A DNA sample is only processed at the laboratory once the customer has activated the kit through the website. As part of the activation step, the customer provides basic personal information, including age and/or year of birth, first and last name, and gender. (Some of this information, such as gender, is used for subsequent quality control steps.) During or after the activation, the customer is able to associate ("link") his or her DNA sample with a node in a pedigree. These pedigrees are accessed through the user's online account, and they are generated either by the customer or by other users (more details are given below).

During activation, each customer is given the option of consenting to the AncestryDNA Human Genetic Diversity research project. Following sample quality control steps described in below, we obtain a final panel of 774,516 genotyped samples consented to participate in research. This is the number of samples available for our subsequent analyses.

Genealogical data

Many customers have provided detailed information about their family history. Online pedigrees are created by individual users or, occasionally, by professional genealogists. An individual can

associate a DNA sample to a node in any pedigree that is accessible through their Ancestry user account. Pedigrees are viewable by other users unless a user has marked a pedigree as “private”. This hides the information in the pedigree from public view. For DNA samples linked to pedigrees, we use the pedigree data in aggregate to better understand the historical and geographical significance of the clusters that are identified from the IBD data.

We take a few basic steps to remove associations between DNA samples and pedigrees that are unlikely to be correct. We exclude all pedigrees linked to DNA samples that do not satisfy all of the following criteria: (1) recorded death date for the linked pedigree node (when this death date is available) occurs after AncestryDNA began; (2) the gender is the same as the gender recorded during DNA activation; and (3) the birth date is within 3 years of the birth date recorded during DNA activation. (DNA samples that do not satisfy these criteria are still included in our analyses, but the associated pedigree data is not used.) We note that while more stringent tests for reliability could be used, they are of unclear value given the size and complexity of these data. Finally, we exclude all pedigrees that users marked as “private". After taking these filtering steps, we obtain a final set of 432,611 DNA samples linked to non-private pedigrees.

In all subsequent analyses of the genealogical data, we only include pedigree nodes corresponding to ancestors of the tested individuals; that is, we only retain pedigree nodes x such that the DNA sample is a descendant of x. (These are the only pedigree members that could have passed down genetic material to the associated user.) We exclude all ancestors more than 9 generations back in users’ pedigrees since we have found that the reliability of the pedigree information diminishes considerably after 9 generations.

We include two types of information in our analyses of associated pedigree data: birth year and birth location (map coordinates in longitude and latitude). Other available pedigree information, such as place names, surnames, death dates, and evidence in the form of documents attached to pedigree nodes, are not used in this study. We only use birth locations that include state or province information, and we only use US birth locations that include county or city information.

Based on these pedigree data, the vast majority of the DNA samples are from individuals born in the US; out of the DNA samples linked to pedigrees, 322,683 (96% of reported birth locations) were born in the US, 13,748 (4% of reported birth locations) were born outside the US, and an additional 96,180 (22% of all DNA samples linked to pedigrees) have unreported birth locations. Based on these reported birth locations, we have a reasonably good representation of DNA from individuals born in all US states, with the largest proportion from California (Supplementary Table 1, Supplementary Fig. 1).

The user-generated pedigrees associated with DNA samples exhibit wide variation in size (number of pedigree nodes), completeness (proportion of an individual's ancestors added to the pedigree), and depth (number of generations represented in pedigree); see Supplementary Figs. 12, 13. The average size of the pedigrees also appears to vary somewhat by US state

(Supplementary Fig. 14, Supplementary Table 1); for example, pedigrees for individuals born in Maine and Utah have, on average, the largest pedigrees.

About 76% of all pedigree nodes include birth location, birth year and surname. Reassuringly, the proportion of pedigree nodes with these three annotations does not appear to vary by generation (Supplementary Fig. 15). In other words, if an ancestral node is present in a pedigree, how well that node is annotated does not appear to be strongly affected by the depth of that ancestor in the pedigree. Annotation completeness varies only slightly by US state (Supplementary Table 1). Variability in tree and annotation completeness could be partly a result of access to historical records, either in the US, or for different ethnic groups.

We caution that pedigree information from different users is not always independent. For example, multiple DNA samples may be linked to similar or identical pedigrees. This may even be the case for individuals that are more distantly related to each other. (With Ancestry's "hinting" system, new relatives can be suggested for an individual's pedigree based on similarity with other online pedigrees. Although this system has been successful for helping users expand their family trees, it can also perpetuate errors in pedigrees.) Adoption is another source of error, although this is expected to have a limited impact. (Users can mark some lines as biological or adoptive, but many users are unaware of this feature.) For the purposes of this study, we assume that inaccurate pedigree data has a negligible impact on the accuracy of the summary statistics when they are compiled from thousands of pedigrees.

Phasing reference panel

The reference panel is based on 217,722 genotype samples that were available in the AncestryDNA database at the time when the panel was constructed. We use a subset of 633,299 autosomal SNPs for all steps of the phasing analysis. Before phasing these samples, we first impute the small proportion of missing genotypes using Beagle. This is accomplished in batches of 200–1,000 samples. Each of these batches also includes 558 phased samples that were downloaded from the Beagle website[50], and originally collected as part of Phase 1 of the 1,000 Genomes Project[13, 51].

Next, we combine these imputed samples into larger batches of approximately 50,000 samples each. Each of these batches are phased separately using HAPI-UR version 1.01[6]. Once we have phased all the reference samples using HAPI-UR, we learn and store the haplotype models using our Beagle-like algorithm. Phasing new genotype samples using this reference panel takes only a few seconds to complete for each sample.

Genotype phasing algorithm

Beagle defines a probability distribution over haplotypes across each chromosome region, or "window", using a Markov model[55]. The accuracy of the haplotype models increase with sample size[6]. However, Beagle was not designed to scale to hundreds of thousands of samples. An alternative method is HAPI-UR[6], which is able to simultaneously phase tens of thousands of

samples. As of this writing, HAPI-UR can handle larger numbers of samples than Beagle (we are using HAPI-UR version 1.01 and Beagle version 3.3.2). Still, it can take several days for HAPI-UR to complete its computation for the large samples used in this study.

Our algorithm extends Beagle in two ways to accommodate the size of our data set. First, when estimating the transition likelihoods in the haploid models, we add a "pseudocount" (10^{-4}) to each haplotype count. This allows for the possibility that a new genotype sample contains a haplotype that was never previously encountered. Without this modification, the probability of a new haplotype is zero.

Second, we modify the criterion for deciding whether two haplotype clusters (*i.e.* nodes of the haploid Markov model) should be merged during model learning. Since the standard method is overly confident for frequencies that are close to 0 or 1, we regularize the estimates using a symmetric beta distribution as a prior. Specifically, haplotype clusters *x* and *y* are not merged unless the following condition is satisfied for some haplotype *h*:

$$\frac{(\tilde{p}_x^{(h)} - \tilde{p}_y^{(h)})^2}{\frac{\tilde{p}_x^{(h)}(1-\tilde{p}_x^{(h)})}{n_x} + \frac{\tilde{p}_y^{(h)}(1-\tilde{p}_y^{(h)})}{n_y}} \geq C$$

where n_x and n_y are the sizes of clusters *x* and *y*. The posterior allele frequency estimates in this formula are

$$\begin{aligned} \tilde{p}_x^{(h)} &= \frac{n_x(h) + \alpha}{n_x + \alpha + \beta} \\ \tilde{p}_y^{(h)} &= \frac{n_y(h) + \alpha}{n_y + \alpha + \beta} \end{aligned}$$

where $n_x(h)$ and $n_y(h)$ are the numbers of haplotypes that begin with haplotype *h*. We set the parameters of the Beta prior (the prior counts), α and β, to 0.5. Compare this criterion to[55], which merges two clusters unless the following relation holds for some *h*:

$$\left| \hat{p}_x^{(h)} - \hat{p}_y^{(h)} \right| \geq \sqrt{n_x^{-1} + n_y^{-1}}$$

where $\hat{p}_x^{(h)}$is the proportion of haplotypes in cluster *x* with that begin with haplotype *h*, and $\hat{p}_y^{(h)}$is the proportion of haplotypes in cluster *y* that begin with *h*. We evaluated the phasing accuracy of the algorithm using a few different values for constant *C* and settled on *C* = 20.

Evaluation of genotype phasing method

Supplementary Table 7 compares the phasing accuracy of BEAGLE applied to datasets of different size against our phasing method. We evaluated phasing accuracy on a test set of 1,188 unrelated individuals from our database that have been trio-phased. This experiment shows that our implementation infers the phase of new genotype samples more accurately than BEAGLE—and with much lower computational cost—provided we are able to make use of a very large panel of phased genotypes.

Constructing the global ancestry reference panel

Predicting the ethnic origins of an individual's ancestors from their DNA is a central feature of the AncestryDNA product. We use these admixture estimates here to interpret the clusters in relation to worldwide regions (regions in Europe, Africa, and so on). Based on an individual's genotypes, we estimate the proportion of their genome that is attributed to different ancestral populations. For additional details on these methods, refer to the AncestryDNA Ethnicity Estimate White Paper[11].

For estimating admixture proportions, we curate a reference panel of 3,000 "labeled" genotype samples for which we can reasonably trace their origins to one of the 26 ancestral populations. We begin with an initial set of 4,657 labeled samples compiled from multiple sources: 855 samples from 52 worldwide populations collected as part of the Human Genome Diversity Project[2,56]; over 1,200 samples from a proprietary AncestryDNA reference collection; and over 1,600 putatively single-origin samples from AncestryDNA customers that consented to participate in research. To identify AncestryDNA candidates for inclusion in the reference panel, we consult user-generated pedigrees, and we select a customer sample if all lineages trace back to the same geographic region. (More precisely, we check the birth locations of all available grandparents and great-grandparents in the customer's pedigree.) For samples from the proprietary reference collection, we also check birth locations of the most distant ancestors that were provided in the pedigree. We take an additional step to confirm single-origin ancestry of the candidate reference samples in the admixture analysis, detailed below.

The samples from the proprietary collection are genotyped using the same Illumina OmniExpress array (described above). To ensure high-quality genotype data, we follow identical quality control steps to the AncestryDNA samples. The HGDP samples are genotyped on the Illumina 650K platform[2,56]. Therefore, for the admixture analysis we use only the ~300,000 SNPs common to both OmniExpress and 650K arrays.

Population structure analysis can be sensitive to inclusion of genetically related samples. Therefore, we take steps to discard samples so that no two individuals in the reference panel have an unusually high amount of DNA sharing. To quantify DNA sharing, we estimate the probability that 1 and 2 alleles are identical-by-descent (IBD). We use PLINK[57] to compute these probabilities for each pair of individuals. When assessing outlying IBD proportions, we compare against members assigned to the same region only, since different populations can exhibit markedly different IBD distributions (for example, due to historical bottlenecks or rapid

population expansion). This filtering step removes about 100 samples from consideration for the reference panel.

Next, we take an iterative process to gradually refine the reference panel, determine the final populations for admixture estimation, and eliminate samples in which the genetic estimates disagree with the provided labels. This process involves iterating two separate analyses:

(1) We visually inspect the labeled genotype samples projected onto 2 two principal components (PCs). We use this projection to suggest groupings and identify outliers; groups that do not separate as well as others based on this projection are merged. We also use this projection to identify "outliers" that are far from the majority of the samples assigned to the same group. Because the top 2 PCs correspond to different axes of variation depending on the samples included, we repeat this analysis at different "scales", typically by geography; e.g., global samples, then only samples from Europe, then samples from Scandinavia, then samples from Norway only.

(2) We run additional analyses using ADMIXTURE to further identify outliers and groups that are not well delineated genetically. Specifically, we perform a leave-one-out validation, in which we remove the label of one candidate sample from the reference panel, and attempt to estimate the admixture proportions of this sample using the remaining (labeled) reference samples. We repeat this procedure for each sample. Samples that are assigned high admixture proportions to the wrong populations are considered outliers, and removed from the panel. This leave-one-out validation step is also useful for informing population boundaries; groups that are commonly confounded by ADMIXTURE are combined into one population.

After completing this process, the final reference panel consists of 3,000 samples partitioned into K = 26 ancestral populations (Supplementary Fig. 7, Supplementary Table 2). This reference panel is used to estimate admixture proportions in all unlabeled (customer) samples.

Estimating ancestral admixture proportions in unlabeled genotype samples using ADMIXTURE

We use the program ADMIXTURE[58,59] to jointly estimate admixture proportions in the labeled reference panel and unlabeled customer samples from their genotypes. One reason to use ADMIXTURE over other software (e.g., STRUCTURE[60]) is that it can easily incorporate information from labeled samples. Another important reason is that the computation scales well to large data sets, so we can deliver accurate admixture estimates for hundreds or thousands of individuals in a reasonable amount of time.

We iteratively run ADMIXTURE jointly on the 3,000 labeled samples and small batches of unlabeled samples. The size of the batch, as well as the composition of samples included in the batch, can impact the final admixture prediction for a given individual since the population allele frequencies are also adjusted to reflect the unlabeled samples. However, in practice we find that variation in the predictions for different batches is small so long as the number of unlabeled

samples included in a single batch is small relative to the size of the reference panel. Also, since admixture estimates can be sensitive to closely related individuals, we take steps to ensure that no closely related samples (customer samples or reference samples) are included in the same batch.

We set K = 26, and run ADMIXTURE in "supervised" mode (more accurately, it is semi-supervised). We provide population labels (in a `.pop` file) for the 3,000 reference samples only. Since the model assumes independent markers, we use PLINK[57] to prune SNPs that are in high linkage disequilibrium. Because a few ancestral populations represent a large proportion of our reference panel, and therefore dominate the correlation observed in the panel, we calculate the correlation coefficient (r^2) between the same pair of SNPs in all 26 populations separately, then we define a new correlation coefficient by taking the average of the 26 values. We repeat this calculation for all pairs of SNPs within each 50-SNP window. We then prune SNPs until no pair of SNPs within a window has an "averaged" r^2 greater than 0.2. We repeat this process for each window, starting at every 5th marker on a chromosome. After this pruning procedure, we arrive at a set of 112,909 SNPs—these are the SNPs used to estimate the admixture proportions for all unlabeled samples.

Overview of IBD network analysis

Rather than infer population characteristics from IBD patterns in known, or assumed, populations, here we aim to *discover* underlying population structure from IBD. We use a model-free approach, turning to the well-studied problem in machine learning and statistical physics of learning structure in a network. Intuitively, our approach is analogous to the way that principal components analysis (PCA) has been widely used to infer structure from genetic polymorphism data without specifying a demographic process[60]. (Although we note that recent work has formally related PCA to underlying demographic processes; e.g., [61].) The key idea is to transform the problem of inferring population structure from IBD to the well-studied problem of learning structure in a network—that is, an undirected graph with weighted edges[39,45,62]. This is similar in some respects to the approach described by Gusev *et al.*[63]. Our hypothesis is that some of the structural features we identify in the IBD-based network can be related to population demography. (Note this approach is unrelated to reconstruction of haplotype networks[64].)

Our method involves three key steps. First, we estimate the total length of IBD shared between each pair of samples in our database (this step is described above). Second, using the estimated IBD, we construct an *IBD network*—a graph in which vertices correspond to genotyped individuals, and edges are a function of the estimated IBD between each pair (details are given in next section). Third, we build on methods developed in machine learning and statistical physics to study the structural properties of the IBD network.

We take a simple approach to inferring structure in the IBD network by identifying subgraphs with a relatively high density of internal edges—these are commonly called either *modules* or *communities*. (In our paper, we have deliberately avoided using the term "community" in this

way because it can be confused with its usage in population studies, although we do refer to the method as "community detection".) This is the most widely used strategy for inferring network structure, and many algorithms have been developed that can quickly and accurately approximate the modular structure of a network[39,62].

In contrast to PCA applied to genetic polymorphism data, here we do not have the benefit of previous work supporting the interpretation of modular network structure as an underlying demographic process. Another underlying concern is that we cannot guarantee that standard theory and practice of inferring modular network structure is applicable because the IBD network has an unusual combination of properties that distinguish it from other types of networks commonly studied in the literature:

(1) The network is very sparse; for example, if we assign nonzero edges to all pairs with total IBD > 12 cM in our sample (see next section), only 0.2% of pairs are connected in the network.

(2) The edge weights are noisy; IBD as a predictor of familial relationships has high variance, even when detection of IBD is very accurate.

(3) Most community detection methods assign each vertex to a module, but here we expect that many, if not most, individuals do not neatly "fit" within any single module—consider an individual with grandparents from different populations.

(4) In addition, some individuals may not belong to any module; for example, individuals from groups that are poorly represented in our sample.

With these points in mind, we have developed a network analysis based on two complementary methods for inferring modular network structure: (1) a hierarchical clustering method that recursively maximizes the modularity of the network; (2) a spectral analysis method that generates a low-dimension representation of the network structure, which we use to extract "unusually disconnected" subsets of the network. In the remainder of the Supplementary Methods, we use "cluster" to refer exclusively to a subgraph identified by recursive modularity maximization, and a "stable subset" to mean a subgraph identified via the spectral analysis. (Elsewhere in the description of the results, for brevity we also use "cluster" to refer to stable subsets when the distinction between cluster and stable subset is not relevant to the result.) We use the term "stable subset" to contrast with degenerate network clusterings[40] that may underlie continuous variation in IBD due to, for example, isolation-by-distance. These stable subsets tend to isolate the more discontinuous portion of variation in IBD that putatively reflects IBD patterns from distinct subgroups—see Supplementary Fig. 6 for an illustration of this using simulated data. Unlike the hierarchical clustering, in the spectral analysis we do not attempt to assign every individual to a cluster—we only use it to identify subsets that are unusually disconnected from the rest of the network. Since we have found that this type of population structure is typically more straightforward to interpret, much of our presentation focuses on annotating and interpreting the network structure captured by the spectral analysis. For

additional discussion of the relationship between the hierarchical clustering and the spectral analysis, see "Discussion of network analysis" in the Supplementary Text.

Constructing the IBD network

There are two key considerations that constrain our choice of edge weight function $w[e(i, j)]$. First, only a very small proportion of estimated IBD segment lengths are suggestive of close relationships. Therefore, if we place most of the weight on close relationships, the graph will be extremely sparse and disconnected, and there will be little population demographic structure that can be inferred from the graph. A second consideration is that while GERMLINE can infer longer IBD segments with high accuracy, it cannot reliably distinguish between shorter tracts that are truly inherited from a common ancestor and false positive IBD[65]. Therefore, if we place substantial weight on shorter shared IBD arising from more distant familial relationships (e.g., IBD less than 4 cM in length), there is a good chance that the "noise" in the network structure will overwhelm the pattern of genetic connections that we are ultimately interested in investigating.

Within these constraints, we still have considerable flexibility for defining edge weights from IBD. Our strategy is as follows. First, we choose a target range of ancestral generations. Second, we empirically assess the distribution of IBD lengths via simulation. Third, we place most weight on IBD lengths arising from familial relationships corresponding to the target generations. The defined edge weights necessarily hinge on the assumptions made in the simulations. Therefore, we make these assumptions, and their rationale, clear.

We compile estimated IBD from a variety of familial relationships by simulating reproductive events from a subset of the sample genotypes. This simulation is intended to capture the correspondence between familial relationship (specifically, number of separating meioses) and IBD segment length for an "idealized" (random mating) population with a population distribution that reflects our customer database. We begin with a subset of 24,362 samples selected so that no pair of samples share a 20-cM IBD segment (as detected using the procedures described above). We draw samples at random without replacement to simulate familial relationships as close as parent-child and as distant as 10th cousins. Supplementary Fig. 29 gives an example simulation of two individuals (labeled *S3* and *S4*) with a first-cousin relationship. All other familial relationships are simulated in a similar way. Note that we do not simulate relationships, such as half-sibs, that do not follow this pedigree pattern. Recombination events during meiosis are simulated according to interpolated HapMap genetic distances[66]. The final product of the simulations is 4,412 genomes with known familial relationships.

As discussed above, we note that this simulation does not capture population-specific excess IBD sharing due to founder events, non-random mating, rapid population expansion, or both. This means that for some subpopulations, most of the edge weights will be more correctly concentrated on IBD due to ancestors a specified number of generations back, whereas for other subpopulations with sharp deviations from our simulation assumptions (e.g., European Jewish), the IBD corresponds in expectation to slightly more distant common ancestors. As a

result, clustering of the IBD network could reveal population structure that is further back from the target generations.

The distribution of total IBD from this simulation experiment is summarized in Supplementary Fig. 30. Note that the proportions for a given amount of total IBD depend on the relative number of relationships simulated of a given type. We ensured that the number of relationships doubles for every increase of 2 to the number of separating meioses (e.g., there are twice as many pairs separated by 6 meioses as there are separated by 4 meioses).

Finally, we define the edge weights in the network as the observed proportion of total IBD lengths that are due to relationships separated by at most 8 meioses (corresponding to common ancestors at most 4 generations back). This empirical distribution is fit to the Beta cumulative density function, and this fitted distribution (with scale parameters $\alpha = 2$, $\beta = 200$) defines the weights for all edges in the network (refer to Supplementary Fig. 2 for more details). Furthermore, we remove all edges corresponding to pairs with total IBD less than 12 cM since they signal the target familial relationships less than 6% of the time, and therefore contribute little weight to the network. Intuitively, pruning edges representing small amounts of genomic sharing allows us to focus on IBD that corresponds to more recent demography, while also removing significant amounts of spuriously identified IBD[65]. This reduces the chance of having edges corresponding to false-positive IBD, while allowing for a large number of edges in the graph.

Hierarchical clustering of IBD network

In this phase of our analysis, we employ a simple and fast heuristic algorithm, the *multi-level* or *Louvain* method[67], to identify network modules. After running this multi-level community detection algorithm, 99.9% of the IBD network (768,758 out of 769,444 vertices) is subdivided into 6 clusters (Supplementary Data 2). The rest of the network (0.1% of the vertices) is assigned to many extremely small clusters with at most 101 members. Many of these small clusters likely correspond to subpopulations that have poor representation in our database, or to unusually large, tight-knit families. They are difficult to interpret based on the available genealogical data, so we do not examine them further.

To investigate more fine-scale clustering, and potentially fine-scale population structure, we split the original network into 5 sub-networks corresponding to the largest 5 clusters, then we partition these sub-networks into additional clusters using the same multi-level community detection algorithm. Although the smallest clusters are more likely to contain additional modular structure than larger clusters[68], to safeguard the clustering against over-fitting due to noise in the observed edges and the sparse number of pairs with IBD > 12 cM, we only run this second round of community detection on the 5 clusters with at least 10,000 members. (Of the 6 initial clusters, the smallest contains only 3,845 samples; see Supplementary Data 2.)

Applying the multi-level algorithm to the 5 sub-networks partitions the network into a total of 112 clusters, the majority of which are very small; 71 out of the 112 have less than 100 members.

Again, since small clusters are more difficult to reliably interpret with the available genealogical data, in the results we restrict in our attention to clusters with at least 2,000 members. A total of 22 second-level clusters have at least 2,000 members (Supplementary Data 2). These 22 clusters, and the 6th-largest top-level cluster that was not subdivided further, account for 98.8% of the network (759,925 out of 769,444 vertices). We observe wide variation in size among the 22 largest second-level clusters; the largest has 108,786 members, while others have just over 2,000 members.

To generate the third level of the cluster hierarchy, we apply the multi-level method to the 12 second-level (sub-network) clusters with more than 10,000 members. Many of these clusters are likely informative of additional, more subtle trends in population structure, but are often difficult to interpret unambiguously from our data. In total, we identify 164 clusters within the 12 second-level clusters. As before, many of these clusters are small; 64 have less than 100 members. Still, we identify a total of 55 clusters with more than 2,000 members, the largest of which includes 65,551 samples (this is a subset of the "Lower Midwest and Appalachians" cluster; see Supplementary Data 2).

Subsequent inspection of the genealogical data in the third-level clustering strongly indicates that many of these clusters are highly informative of fine-scale population structure—some of the more unmistakable examples are clusters corresponding to Italians, Irish, Scottish, Finnish, Norwegians and Puerto Ricans (see Supplementary Figs. 25–27 and Supplementary Discussion). However, out of concern for reporting network structure attributed to synthetic factors (either false positive clusters, or clustering that can be strongly biased by properties of the modularity-maximization approach), as well as the difficulty of characterizing the subtle population structure underlying many of these clusters, we focus on the first and second levels of the hierarchical clustering in the main presentation of the results. Although the third-level clustering is not the focus of our main presentation, we do treat these clusters as candidates for the spectral analysis (see below). Some of these clusters are indeed supported by the spectral analysis; that is, some of the clusters detected in the largest second-level clusters closely coincide with “stable subsets” identified in the spectral analysis (Supplementary Data 2).

Spectral analysis of IBD network

We complement the hierarchical clustering using a spectral dimensionality reduction technique for network data. Our spectral analysis approach is based on the Laplacian eigenmaps method[69], which has close connections to spectral clustering[45,46]. Spectral methods have been previously used to infer population structure from genetic data[42,43].

The Laplacian eigenmaps method[69] is derived from a spectral decomposition of the (normalized) Laplacian matrix. The intuition behind this approach is that the eigenvectors associated with the largest eigenvalues of the Laplacian matrix (outside the largest eigenvalue, which is always 1, or nearly 1) separate disconnected components, or weakly connected components, of the graph.

We define the *spectral embedding* as the first m eigenvectors of the normalized Laplacian. (To the best of our knowledge, there are no theoretical results guiding the choice of m in this setting, and this is a point we return to below.) We efficiently solve for the largest m eigenvectors of the sparse, symmetric $n \times n$ Laplacian matrix using the Lanczos iterative algorithm[70], implemented in ARPACK[71], and interfaced to *R* through the *rARPACK* library. To justify the interpretation of the spectral embedding as the projection of samples onto a Euclidean space, we note that the first m eigenvectors and eigenvalues can be used to formally define a projection operator[72].

A key feature of the spectral analysis is that provides a continuous representation of network structure, potentially overcoming the unnatural assumption that each sample belongs to a single cluster, or population. However, it is currently unknown how to generalize interpretation of this representation beyond the few examples we have seen where some dimensions of the spectral embedding correlate strongly with admixture proportions estimated using ADMIXTURE (Supplementary Fig. 22). Therefore, we take a simple approach to infer population structure from the spectral embedding by projecting the hierarchical clustering onto this embedding, then using this projection to extract clusters. These clusters, which we refer to as "stable subsets," represent unusually disconnected portions of the network.

Before describing our procedures for identifying stable subsets more formally, we first give an example to illustrate how the spectral embedding can be used to identify these highly disconnected subsets. Supplementary Fig. 31 shows the projection of all genotyped individuals (*i.e.*, vertices in the IBD network) onto the first two dimensions of the spectral embedding. The vast majority of samples are concentrated near the origin. Labeling the samples according to their membership to the first-level IBD network clusters, we find that many of the samples projecting away from the origin along the first dimension are assigned to the cluster labeled as "Caribbeans"; these are drawn as blue circles and crosses in the figure. Many of the samples projected away from the origin along the second dimension belong to the cluster labeled as "European Jewish"; these are drawn as red circles and crosses in the figure. (Later, we explain how we interpret these clusters as European Jewish and Caribbean Islanders.)

To define a stable subset for the Caribbeans cluster, we specify a classification rule of the form $y_{i,1} > b_1 \wedge y_{i,2} > b_2 \Rightarrow$ "*i* is a member of Caribbeans stable subset". Here, $y_{i,1}$ and $y_{i,2}$ are the projection of sample i onto dimensions 1 and 2 of the spectral embedding, rotated by r degrees. The choice of this rule—that is, the choice of parameters b_1, b_2 and r—is subject to the following condition: most of the samples satisfying this classification rule must also be assigned to the Caribbeans cluster (again, as identified by hierarchical clustering). In other words, the rate of "false positives" must be low, in which we define "true positives" and "false positives" using hierarchical clustering membership as the “ground-truth.” Setting $b_1 = 0.001$, $b_2 = -0.0005$ and $r = 8$ degrees counter-clockwise, 9,315 out of 11,807 Caribbean cluster members are assigned to the stable subset (79% recall). These are the blue circles in Supplementary Fig. 31. The 2,492 cluster members that are not assigned to the stable subset are shown as blue crosses. There are an additional 60 samples that satisfy this classification rule, but are not members of the Caribbean cluster (and so are not represented in the figure). Therefore, we obtain a false positive rate of only 0.6%. The final stable subset we report is the set of 9,315 samples that

satisfy this classification rule and are assigned to the Caribbeans cluster in the hierarchical clustering (see Supplementary Data 2).

Following the same procedure, we define the stable subset for the European Jewish cluster. In this case, we specify the classification rule as $y_{i,1} < 0.001 \wedge y_{i,2} < -0.001 \Rightarrow$ "i is a member of European Jewish stable subset". Applying this classifier, 26,547 out of 32,708 cluster members are included in the stable subset (81% recall). The selected samples are the red circles in the figure, and the remaining cluster members are shown as red crosses. In this case, only 13 individuals that are not members of the European Jewish cluster satisfy this classification rule, for a very low false positive rate of 0.05%. The final stable subset we report is the set of 26,547 samples that satisfy this classification rule and are assigned to the cluster. Below, we describe the desired thresholds for recall and false positive rate in the identification of stable subsets, as well as our rationale for using the hierarchical clustering as validation.

Note that many of the members of the two clusters that are not included in their respective stable subsets—the blue and red crosses—lie somewhere in between the two stable subsets. Our conjecture is that this is an example of how the community detection method arbitrarily assigns "admixed" individuals to a single cluster—these are putatively individuals of both Jewish and Caribbean descent—whereas the mapping onto the spectral embedding allows us to distinguish such individuals.

Once the eigenvectors corresponding to the largest *m* eigenvalues have been computed, a common strategy is to use a simple algorithm such as *k*-means to estimate clusters in the spectral embedding (e.g., [46]). Although this approach has been successful in many domains, the "local scaling problem" in spectral clustering, which has been studied in other types of data[73], and here reflects the relative density of the IBD connections, complicates this enormously. This is readily appreciated—in two dimensions, at least—by observing the wide variation in dispersal of the clusters identified in the spectral embedding (Supplementary Figs. 4, 5). To circumvent the local scaling problem, we use the projection of the hierarchical clustering onto the spectral embedding to generate a set of "candidate clusters" for the spectral analysis.

Our formal procedure for identifying stable subsets is as follows. The first step is to identify a cluster that projects away from the origin in the spectral embedding. This is accomplished simply by visually inspecting the projection of the candidate clusters in the spectral embedding, in which we label the clusters with different colors and symbol shapes (see Supplementary Figs. 4, 5 for examples of this). Obviously, this can be only realistically done in two dimensions at a time. Further, to make this process more tractable, we only inspect pairs of consecutive eigenvectors; that is, $j = i + 1$. It is conceivable that some clusters are better delineated by non-consecutive pairs of eigenvectors, or even more than two eigenvectors[74], but we did not investigate this. Thus, this procedure does not guarantee identification of all stable subsets in the spectral embedding.

Once we have selected a cluster that projects away from the origin, we attempt to identify a subset satisfying the following definition: a subset of a hierarchical cluster is defined as a *stable*

subset if it is possible to specify a simple linear classification rule (detailed above) using eigenvectors *i* and *j* to classify some of the individuals to the same cluster with a low rate of false positives (again, a "false positive" is a sample that is incorrectly classified according to the cluster assignment from the hierarchical clustering). This definition does not uniquely determine the stable sets, as there is still considerable flexibility in how these stable subsets can be chosen from the spectral embedding. Our approach is to specify the linear decision rule that recovers the largest number of cluster members (that is, high recall), while keeping the false positive rate acceptably low (as a guideline, we use 10%). All stable subsets we detect in the spectral embedding have low false positive rates; the largest false positive rate, in the Central American cluster, is 12% (Supplementary Data 2). Finally, to verify that the selected cluster is the most appropriate one for defining the stable subset, we check that the correlation between the linear classifier and cluster assignment is highest for the selected hierarchical cluster. Linear decision rules, false positive rates and other details for all identified stable subsets are given in Supplementary Data 2.

Beyond the local scaling problem, another challenge with identifying clusters in the spectral embedding is that in most dimensions of the spectral embedding, only a small number of samples project away from the origin—typically far less than 100. These correspond to very small subgraphs of the IBD network that are the least connected with the rest of the network. (Note that these highly disconnected subgraphs do not necessarily correspond to the very small clusters identified in the first, second and third rounds of the hierarchical clustering.) Furthermore, many of the same clusters appear in multiple dimensions of the spectral embedding. In short, the spectral embedding captures the clusters and small numbers of samples that exhibit the most dominant modular structure in the network, possibly obscuring other, more subtly disconnected subsets. (In some respects, this is the opposite of the "resolution limit" problem in modularity-maximizing methods[68], in which strong modular structure in small subgraphs can be obscured by more subtle modular structure in large portions of the network.)

We address this issue by isolating the subgraph for which we have not identified any substructure, analogous to the approach of recursively subdividing clusters in the hierarchical clustering. Initially, we compute the spectral embedding from the completely connected graph with 769,444 vertices. Once we have completed the spectral analysis of this graph, we compute the spectral embedding from a subgraph with 586,147 vertices that is obtained by first removing the small sets of individuals and the clusters that project away from the origin in the initial spectral embedding (*i.e.*, the clusters for which we are able to identify stable subsets in the first phase). Therefore, we use two sets of eigenvectors in the spectral analysis: eigenvectors of the Laplacian defined by the completely connected graph with 769,444 vertices; and eigenvectors of the Laplacian defined by the subgraph on 586,147 vertices.

Finally, an important aspect to this procedure that remains unresolved is the number of eigenvectors that are used to define the spectral embedding. One commonly proposed criterion is the "eigengap" heuristic[42], which is based on theory showing that a relatively large difference in consecutive eigenvalues of the Laplacian matrix is suggestive of the number of disconnected

components of the graph. However, this heuristic only works well if the network contains very well-pronounced modules[45], which is not the case here. Here, we limit the spectral embedding to the top m = 40 eigenvectors, primarily for manageability of the analysis procedure. It is possible that inspecting more eigenvectors (with smaller eigenvalues) could provide support for additional substructure in the IBD network.

Projecting 1000 Genomes samples onto the spectral embedding

Since the spectral embedding defines a projection operator[63], we can map new genotype samples onto the manifold defined by this embedding. This allows us to validate against data not used to construct the embedding. Here, we use the 1000 Genomes data[31]. The SNPs genotyped in the AncestryDNA samples (using the OmniExpress chip) were also genotyped in 1,816 unrelated 1000 Genomes samples (using the Illumina OMNI 2.5M chip), so we can follow the steps above to estimate IBD between all pairs (i, j), in which i is a 1000 Genomes sample and j is an AncestryDNA sample. (Note that we do not need to estimate IBD shared by 1000 Genomes samples as it is not needed to compute the projection, below.) These IBD detection results are summarized in Supplementary Table 3. Since only 1,219 out of 1,816 samples share greater than 12 cM IBD with at least one AncestryDNA sample (and therefore has at least one network edge with a positive weight), only these 1,219 samples are retained for the validation. These IBD data form an $n^* \times n$ matrix $\mathbf{W^*}$ with entries $w[e(i, j)]$, where n^* = 1,219 and n = 774,516. Next, representing the spectral decomposition of the Laplacian as an $n \times m$ matrix of eigenvectors, $\mathbf{R}$, and $m \times m$ diagonal matrix of eigenvalues, $\mathbf{A}$, the projection is defined as $\mathbf{R^*} = (\mathbf{D^*})^{-1/2}\mathbf{W^*D}^{-1/2}\mathbf{RA}^{-1}$, where $\mathbf{D^*}$ is an $n^* \times n^*$ diagonal matrix with diagonal entries $D^*(i, i)$ each equal to the sum of the edge weights $w[e(i, j)]$.)]. The rows of $\mathbf{R^*}$ define how the samples are projected onto the spectral embedding, and can be used to create visualizations of the 1000 Genomes data (e.g., Fig. 6).

Supplementary References

1. Ball, C. A. *et al.* Ethnicity Estimate White Paper. http://dna.ancestry.com/resource/whitePaper/AncestryDNA-Ethnicity-White-Paper (2013).

2. Cann, H. M. *et al.* A human genome diversity cell line panel. *Science* **296**, 261–262 (2002).

3. Auton, A. *et al.* A global reference for human genetic variation. *Nature* **526**, 68–74 (2015).

4. Rosenberg, N. A. Standardized subsets of the HGDP-CEPH human genome diversity cell line panel, accounting for atypical and duplicated samples and pairs of close relatives. *Ann. Hum. Genet.* **70**, 841–847 (2006). See also http://rosenberglab.stanford.edu/data/rosenberg2006ahg/SampleInformation.txt.

5. Browning, S. R. & Browning, B. L. Haplotype phasing: existing methods and new developments. *Nat. Rev. Genet.* **12**, 703–714 (2011).

6. Williams, A. L., Patterson, N., Glessner, J., Hakonarson, H. & Reich, D. Phasing of many thousands of genotyped samples. *Am. J. Hum. Genet.* **91**, 283–251 (2012).

7. Billington, R. A. & Ridge, M. *Westward expansion: a history of the American frontier* (University of New Mexico Press, Albuquerque, NM, 6th ed. abridged, 2001).

8. White, R. *Railroaded: the Transcontinentals and the making of modern America* (Norton & Company, 2012).

9. Arsenault, B. *Histoire des acadiens* (Éditions Fides, 2004).

10. Bryc, K. *et al.* Genome-wide patterns of population structure and admixture in West Africans and African Americans. *P. Natl. Acad. Sci. USA* **107**, 786–791 (2010).

11. Atzmon, G. *et al.*, Abraham's children in the genome era: major Jewish diaspora populations comprise distinct genetic clusters with shared Middle Eastern ancestry. *Am. J. Hum. Genet.* **86**, 850–859 (2010).

13. 1000 Genomes Project Consortium, An integrated map of genetic variation from 1,092 human genomes. *Nature* **491**, 56–65 (2012).

14. Leslie, S. *et al.* The fine-scale genetic structure of the British population. *Nature* **519**, 309–314 (2015).

15. Nadler, B., Lafon, S., Coifman, R. R. & Kevrekidis, I. G. Diffusion maps, spectral clustering and eigenfunctions of Fokker-Planck operators. *Appl. Comput. Harmon. A.* **21**, 5–30 (2005).

16. Rastogi, S., Johnson, T. D., Hoeffel, E. M. & Drewery, M. P. *The Black Population: 2010.* US Census Bureau. http://www.census.gov/prod/cen2010/briefs/c2010br-06.pdf (2011).

17. Williams, J. R. *In pursuit of their dreams: a history of Azorean immigration to the United States* (Dartmouth Center for Portuguese Studies and Culture, University of Massachusetts, Dartmouth, 2005).

18. Timeline: key dates and Landmarks in United States immigration history. Aspiration, Acculturation, and Impact: Immigration to the United States, 1789-1930. Harvard University Open Collections Program, http://ocp.hul.harvard.edu/immigration/timeline.html (accessed May 31, 2015).

19. Scandinavian Immigration, Aspiration, Acculturation, and Impact: Immigration to the United States, 1789-1930. Harvard University Open Collections Program. http://ocp.hul.harvard.edu/immigration/scandinavian.html (accessed June 1, 2015).

20. Immigration Explorer, New York Times. http://www.nytimes.com/interactive/2009/03/10/us/20090310-immigration-explorer.html (March 10, 2009).

21. Fitzgerald, P. & Lambkin, B. *Migration in Irish history 1607–2007* (Palgrave Macmillan, New York, 2008).

22. Foley, M. C. & Guinnane, T. W. Did Irish marriage patterns survive the emigrant voyage? Irish-American nuptiality, 1880–1920. *J. Econ. Soc. Hist.* **26**, 15–24 (1999).

23. Nolan, J. A. *Ourselves alone: Women's emigration from Ireland, 1885–1920* (University Press of Kentucky, Lexington, KY, 1989).

24. Kim, S. K. *et al.* Population genetic structure and origins of native Hawaiians in the Multiethnic Cohort Study. *PLoS ONE* **7**, 1–10 (2012).

25. Simmons, M. *Spanish pathways: readings in the history of Hispanic New Mexico* (University of New Mexico Press, 2001).

26. Via, M. *et al.* History shaped the geographic distribution of genomic admixture on the island of Puerto Rico. *PLoS ONE* **6**, e16513 (2011).

27. Pobutsky, A. & Krupitsky, D. A demographic profile of Hispanics in Hawaii—implications for population health? *Hawai'i J. Public Heal.* **2**, 67–73 (2009).

28. Fogleman, A. S. *Hopeful journeys: German immigration, settlement, and political culture in colonial America, 1717–1775* (University of Pennsylvania Press, Philadelphia, PA, 1996).

29. Keith, L. *Alabama Fever*. Encyclopedia of Alabama. http://www.encyclopediaofalabama.org/ article/h-3155 (published October 14, 2011; accessed May 27, 2015).

30. Rogers, T. W. The great population exodus from South Carolina, 1850–1860. *South Carolina Historical Magazine* **68**, 14-21 (1967).

31. Edgar, W. B. *South Carolina: a history* (University of South Carolina Press, Columbia, SC, 1998).

32. Hudson, J. C. *Across this land: a regional geography of the United States and Canada*. (Johns Hopkins University Press, Baltimore, MD, 2002).

33. Hoffman, P. E. *Florida's frontiers* (Indiana University Press, Bloomington, IN, 2002).

34. Moore, T. G. Eastern Kentucky as a model for Appalachia: the role of literary images. *Southeastern Geographer* **31**, 75–89 (1991).

35. Lewis, R. L. Beyond isolation and homogeneity: diversity and the history of Appalachia. In *Back talk From Appalachia: confronting stereotypes* (D. B. Billings, G. Norman, K. Ledford, eds., University Press of Kentucky, Lexington, KY, 2013).

36. Keel, W. D. From the Netherlands to Kansas: Mennonite Low German. *Heritage of the Great Plains*, vol. 27, Summer 1994, 47.

37. Kroeker, M. E. *Mennonites*. Encyclopedia of Oklahoma History and Culture. http://www.okhistory.org (accessed October 26, 2015).

38. Berry, J. W., Hendrickson, B., LaViolette, R. A. & Phillips, C. A. Tolerating the community detection resolution limit with edge weighting. *Phys. Rev. E* **83**, 056119 (2011).

39. Fortunato, S. Community detection in graphs. *Phys. Rep.* **486**, 75–174 (2010).

40. Good, B. H., de Montjoye, Y. A. & Clauset, A. Performance of modularity maximization in practical contexts. *Phys. Rev. E* **81**, 1–19 (2010).

41. Lee, A. B., Luca, D., Klei, L., Devlin, B. & Roeder, K. Discovering genetic ancestry using spectral graph theory. *Genet. Epidemiol.* **34**, 51–59 (2010).

42. Lee, A. B., Luca, D. & Roeder, K. A spectral graph approach to discovering genetic ancestry. *Ann. Appl. Stat.* **6**, 179–202 (2012).

43. Zhang, J. Ancestral informative marker selection and population structure visualization using sparse Laplacian eigenfunctions. *PLoS ONE* **5**, e13734 (2010).

44. Ruan, J. & Zhang, W. Identifying network communities with a high resolution. *Phys. Rev. E*, **77**, 016104 (2008).

45. Von Luxburg, U. A tutorial on spectral clustering. *Stat. Comput.* **17**, 395–416 (2007).

46. Ng, A. Y., Jordan, M. I. & Weiss, Y. On spectral clustering: analysis and an algorithm. *Adv. Neur. In.* **14**, 849–856 (2001).

47. Carmi, S., Palamara, P. F., Vacic, V., Lencz, T., Darvasi, A., & Pe'er, I. The variance of identity-by-descent sharing in the Wright-Fisher model. *Genetics* **193**, 911–928 (2013).

48. Palamara, P. F., Lencz, T., Darvasi, A. & Pe'er, I. Length distributions of identity by descent reveal fine-scale demographic history. *Am. J. Hum. Genet.* **91**, 809–822 (2012).

49. Palamara, P. F. & Pe'er, I. Inference of historical migration rates via haplotype sharing. *Bioinformatics* **29**, 180–188 (2013).

50. http://bochet.gcc.biostat.washington.edu/beagle/1000_Genomes.phase1_release_v3

51. Browning, B. L. & Browning, S. R. Rapid and accurate haplotype phasing and missing-data inference for whole-genome association studies by use of localized haplotype clustering. *Am. J. Hum. Genet.* **81**, 1084–1096 (2007).

55. Browning, S. R. Multilocus association mapping using variable-length Markov chains. *Am. J. Hum. Genet.* **78**, 903–913 (2006).

56. Cavalli-Sforza, L. L. The Human Genome Diversity Project: past, present and future. *Nat. Rev. Genet.* **6**, 333–340 (2005).

57. Purcell, S. *et al.* PLINK: a tool set for whole-genome association and population-based linkage analyses. *Am. J. Hum. Genet.* ***81***, 559–575 (2007).

58. Alexander, D. H., Novembre, J. & Lange, K. Fast model-based estimation of ancestry in unrelated individuals. *Genome Res.* **19**, 1655–1664 (2009).

59. Alexander, D. H. & Lange, K. Enhancements to the ADMIXTURE algorithm for individual ancestry estimation. *BMC Bioinformatics* **12**, 246 (2011).

60. Novembre, J. & Stephens, M. Interpreting principal component analyses of spatial population genetic variation. *Nat. Genet.* **40**, 646–649 (2008).

61. McVean, G. A genealogical interpretation of principal components analysis. *PLoS Genet.* **5**, e1000686 (2009).

62. Newman, M. E. J. Communities, modules and large-scale structure in networks. *Nat. Phys.* **8**, 25–31 (2011).

63. Gusev, A. *et al.* The architecture of long-range haplotypes shared within and across populations. *Mol. Biol. Evol.* **29**, 473–486 (2012).

64. Tishkoff, S. A. *et al.*, Convergent adaptation of human lactase persistence in Africa and Europe. *Nat. Genet.* **39**, 31–40 (2007).

65. Durand, E. Y., Eriksson, N. & McLean, C. Y. Reducing pervasive false-positive identical-by-descent segments detected by large-scale pedigree analysis. *Mol. Biol. Evol.* **31**, 2212–2222 (2014).

66. The International HapMap Consortium. A second generation human haplotype map of over 3.1 million SNPs. *Nature* **449**, 851–861 (2007).

67. Blondel, V. D., Guillaume, J., Lambiotte, R. & Lefebvre, E. Fast unfolding of communities in large networks. *J. Stat. Mech. Theory E*, P10008 (2008).

68. Fortunato, S. & Barthélemy, M. Resolution limit in community detection. *P. Natl. Acad. Sci. USA* **104**, 36–41 (2007).

69. Belkin, M. &. P. Niyogi. Laplacian Eigenmaps for dimensionality reduction and data representation. *Neural Comput.* **15**, 1373–1396 (2003).

70. Trefethen, L. N. & Bau, D. Numerical linear algebra (SIAM, 1997).

71. Lehoucq, R. B., Sorensen, D. C. & Yang, C. *ARPACK users' guide: solution of large-scale eigenvalue problems with implicitly restarted Arnoldi methods* (SIAM; 1998).

72. Bengio, Y., Paiement, J., Delalleau, P. V. O., Le Roux, N. & Ouimet, M. Out-of-sample extensions for LLE, Isomap, MDS, Eigenmaps and spectral clustering. *Adv. Neur. In.* **16**, 177–184 (2004).

73. Zelnik-Manor, L., Perona, P. Self-tuning spectral clustering. *Adv. Neur. In.* **17**, 1601–1608 (2004).

74. Zhao, F., Jiao, L., Liu, H., Gao, X. & Gong, M. Spectral clustering with eigenvector selection based on entropy ranking. *Neurocomputing* **73**, 1704–1717 (2010).

Supplementary figures

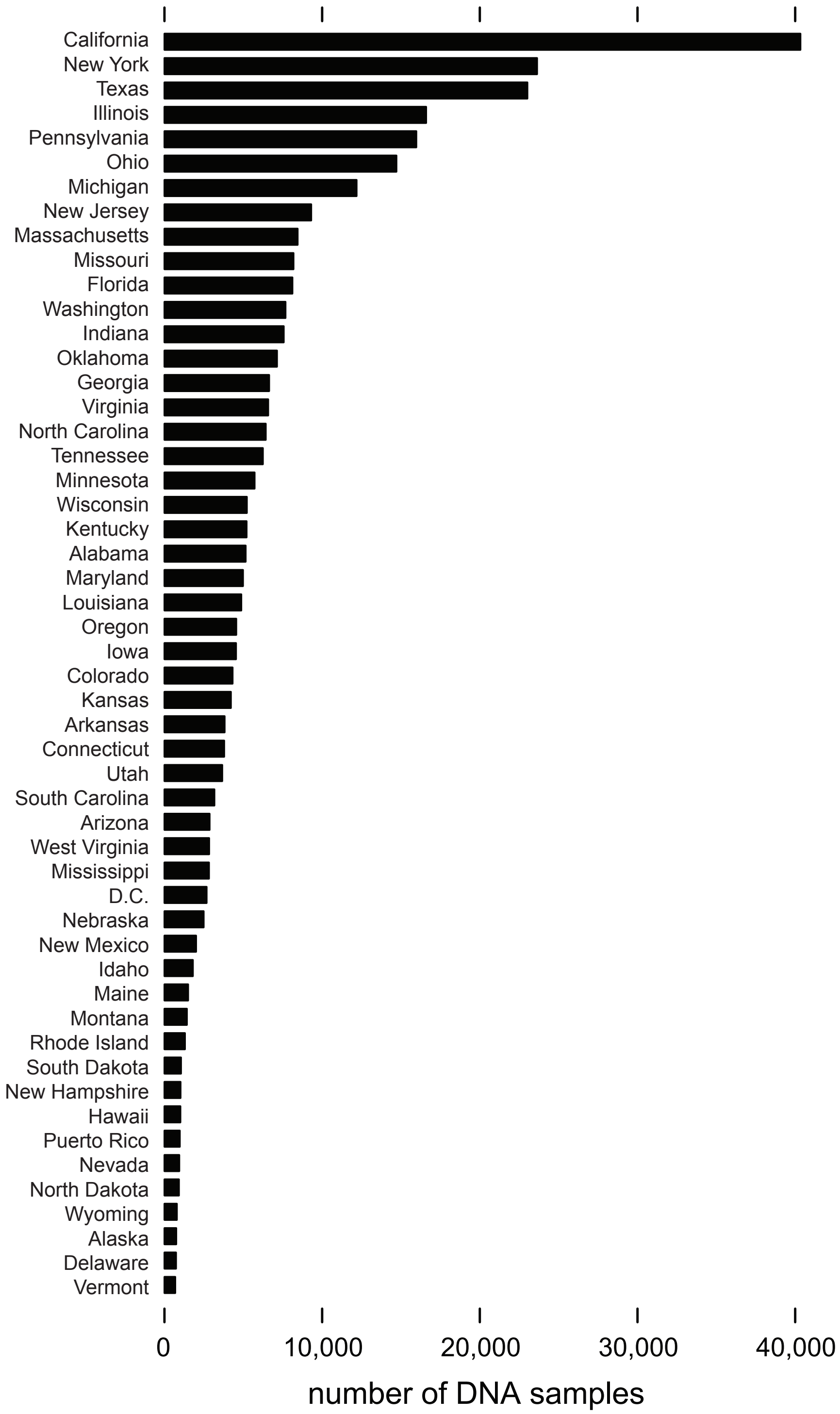

Supplementary Figure 1 | Number of DNA samples with reported birth locations in US states. These numbers are obtained from pedigree nodes linked to DNA samples. Note that the counts by US state don't add up to the total number of US born samples because "US born" also includes Puerto Rico, Guam and other US territories.

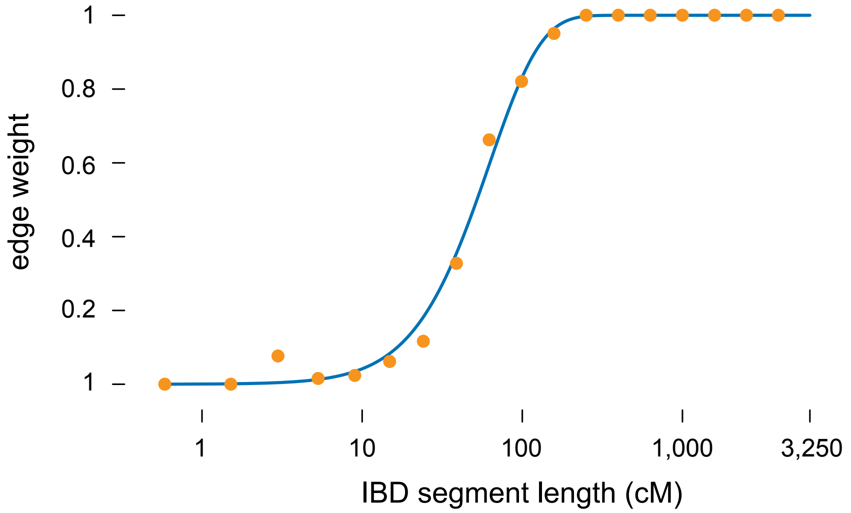

Supplementary Figure 2 | Mapping from total IBD length (in cM) to edge weight. Blue curve gives the mapping from IBD segment length to edge weight. The mapping is defined by the beta distribution $Pr(X \leq x)$, where x is the estimated proportion of the genome that is IBD (or, equivalently, the kinship coefficient), fit to the empirical distribution of $a/(a + b)$, in which a is proportion of relationships that are within 8 meioses or closer, and b is the proportion of relationships that are separated by 9 meioses or more. In other words, the mapping is defined by simple beta approximation to the conditional probability distribution *Pr*(number of separating meioses | total detected IBD). The proportions a and b are calculated from 4,412 genomes with known (simulated) familial relationships; see Supplementary Fig. 30 and Supplementary Methods for more details**.** The fitted beta distribution (the blue curve) has scale parameters α = 2, β = 200.

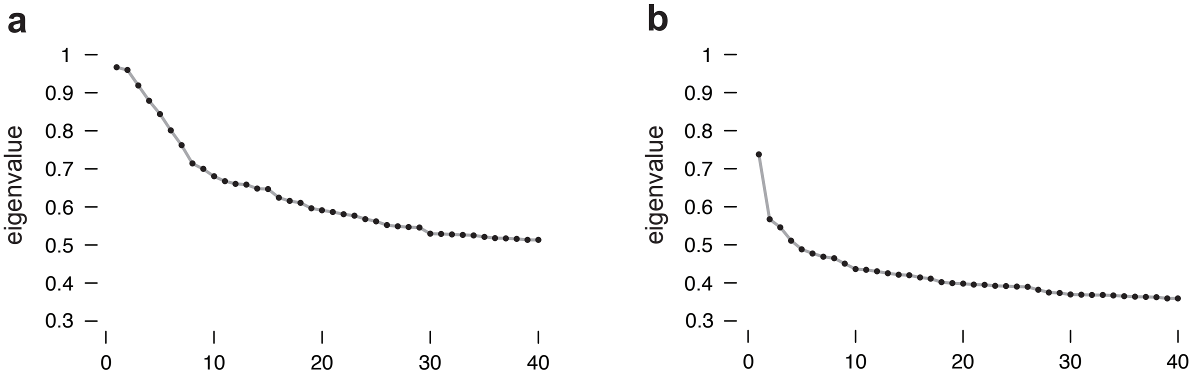

Supplementary Figure 3 | Top 40 eigenvalues of Laplacian matrices. *Panel a:* eigenvalues of the Laplacian matrix computed from the completely connected network (with 769,444 vertices). *Panel b:* eigenvalues of the subgraph Laplacian (with 586,147 vertices) after discarding samples assigned to clusters containing stable subsets identified from the initial Laplacian matrix. The first eigenvalue of 1, or near 1, is omitted from each plot.

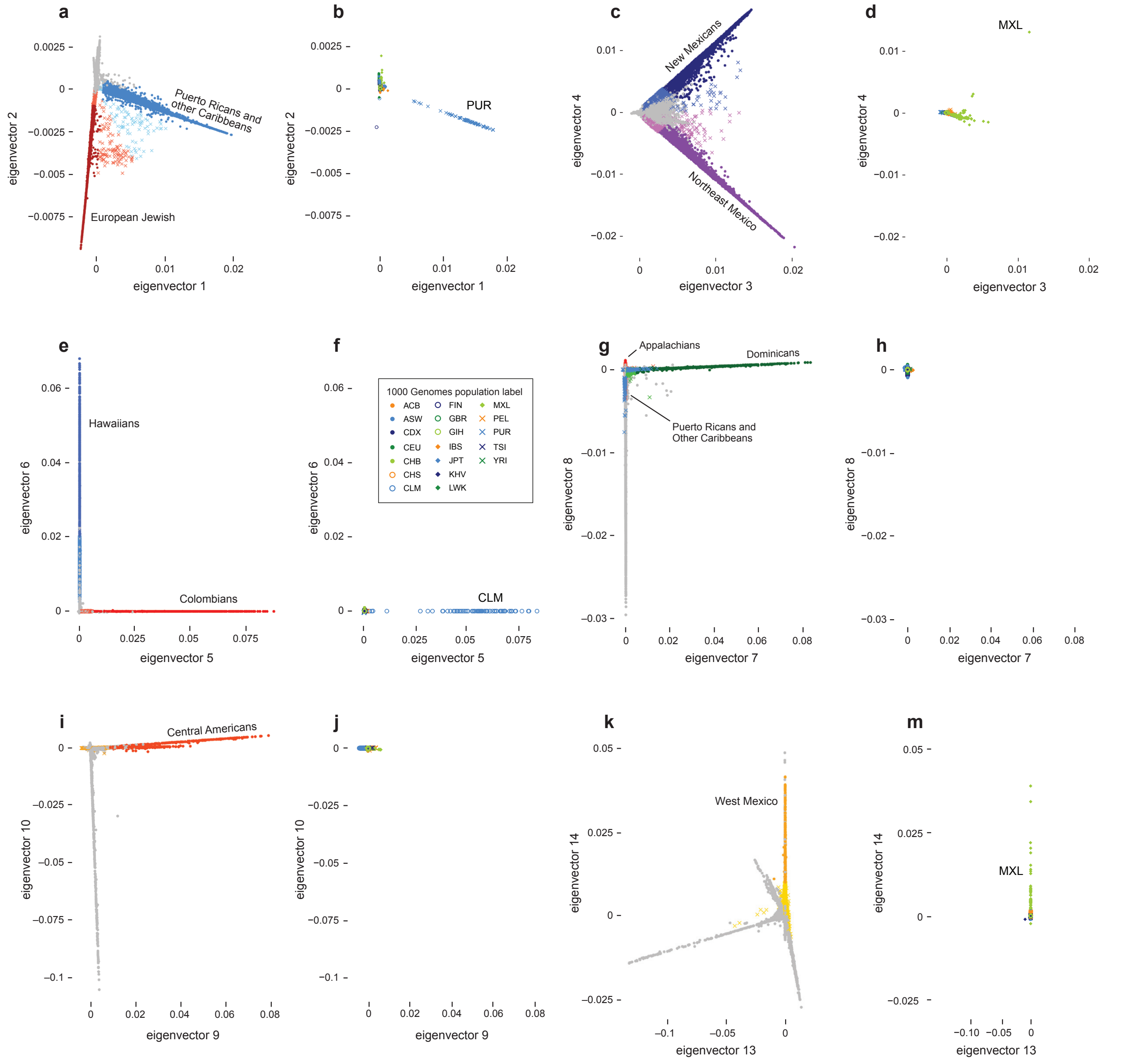

Supplementary Figure 4 | Clusters projected onto spectral embedding. Plots a, c, e, g, i, k show all 769,444 vertices in the completely connected network projected onto different dimensions of the spectral embedding. Samples are colored by membership to selected clusters, and by their assignment to a stable subset (Supplementary Data 2). Samples assigned to the same cluster and stable subset are shown as darker colored circles; samples assigned to the same cluster but not assigned to the stable subset are shown as lighter colored crosses; all other samples are shown as light gray circles. Some dimensions appear to suggest additional stable subsets, but are not highlighted in the plots for one of the following three reasons: (1) the samples are not assigned consistently to a single cluster in the hierarchical clustering; (2) the stable subset includes only a very small number of samples, so it is difficult to interpret; or (3) the samples are assigned to a stable subset based on the projection onto other dimensions. Note that the separation of the Appalachians cluster is less visually apparent because the

samples are projected onto a small region. For validation, plots b, d, f h, j, m show the projection of 1000 Genomes[62] samples onto the same dimensions of spectral embedding. The projection is computed from IBD estimated between all pairs of AncestryDNA and 1000 Genomes samples. These samples in are colored according to the provided population label. See Supplementary Data 3 for an explanation of the abbreviations used for the population labels.

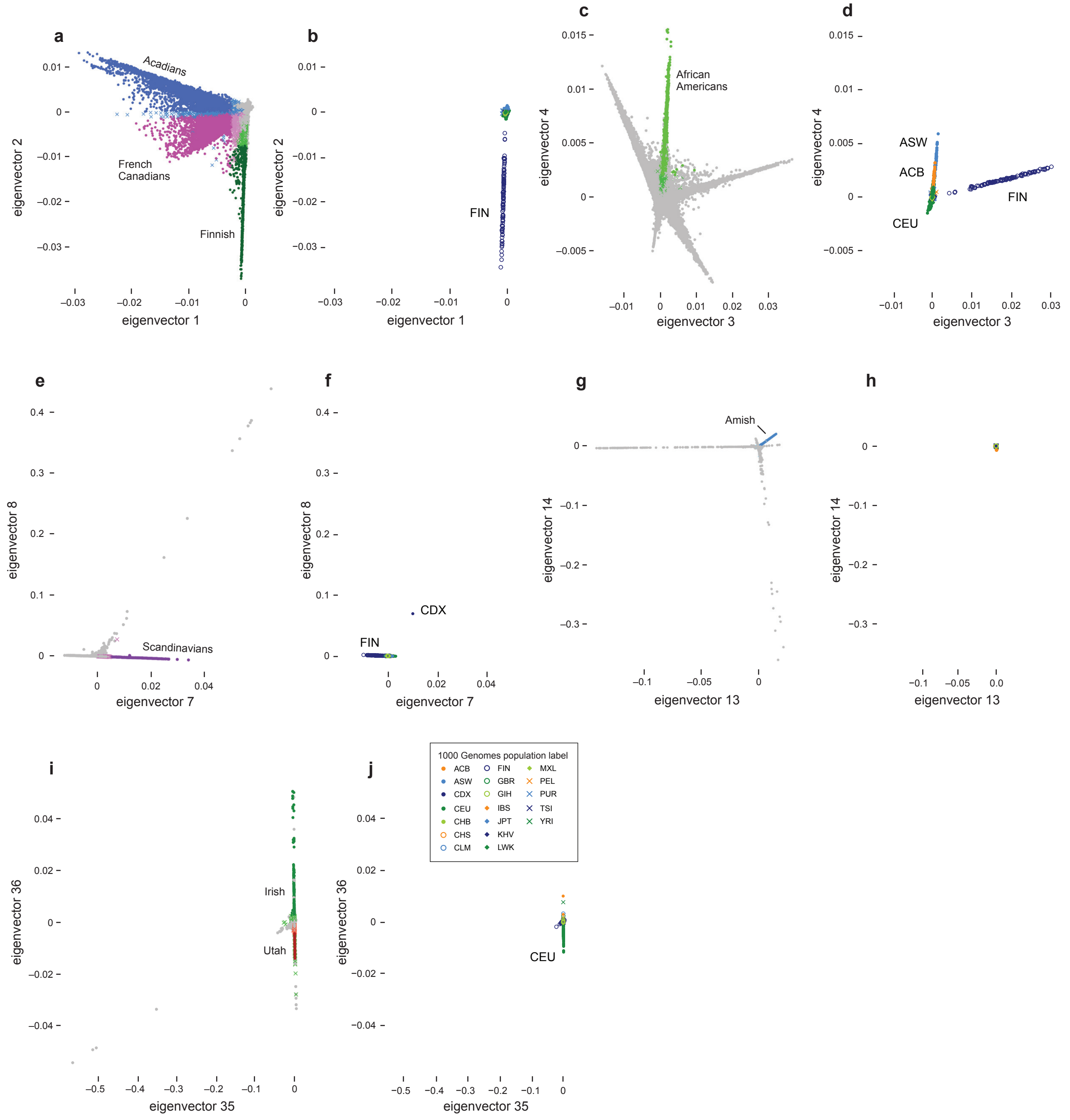

Supplementary Figure 5 | Clusters projected onto subgraph spectral embedding. The subgraph is obtained by discarding vertices assigned to clusters containing stable subsets identified in the initial spectral embedding. Plots a, c, e, g, i show all 586,147 vertices in the subgraph projected onto different dimensions of the spectral embedding. Samples are colored by membership to selected clusters, and by their assignment to a stable subset (Supplementary Data 2). For validation of the spectral analysis, plots b, d, f, h, j, m show the projection of 1000 Genomes[62] samples onto the same dimensions of spectral embedding. See description of Supplementary Figure 4 for additional details.

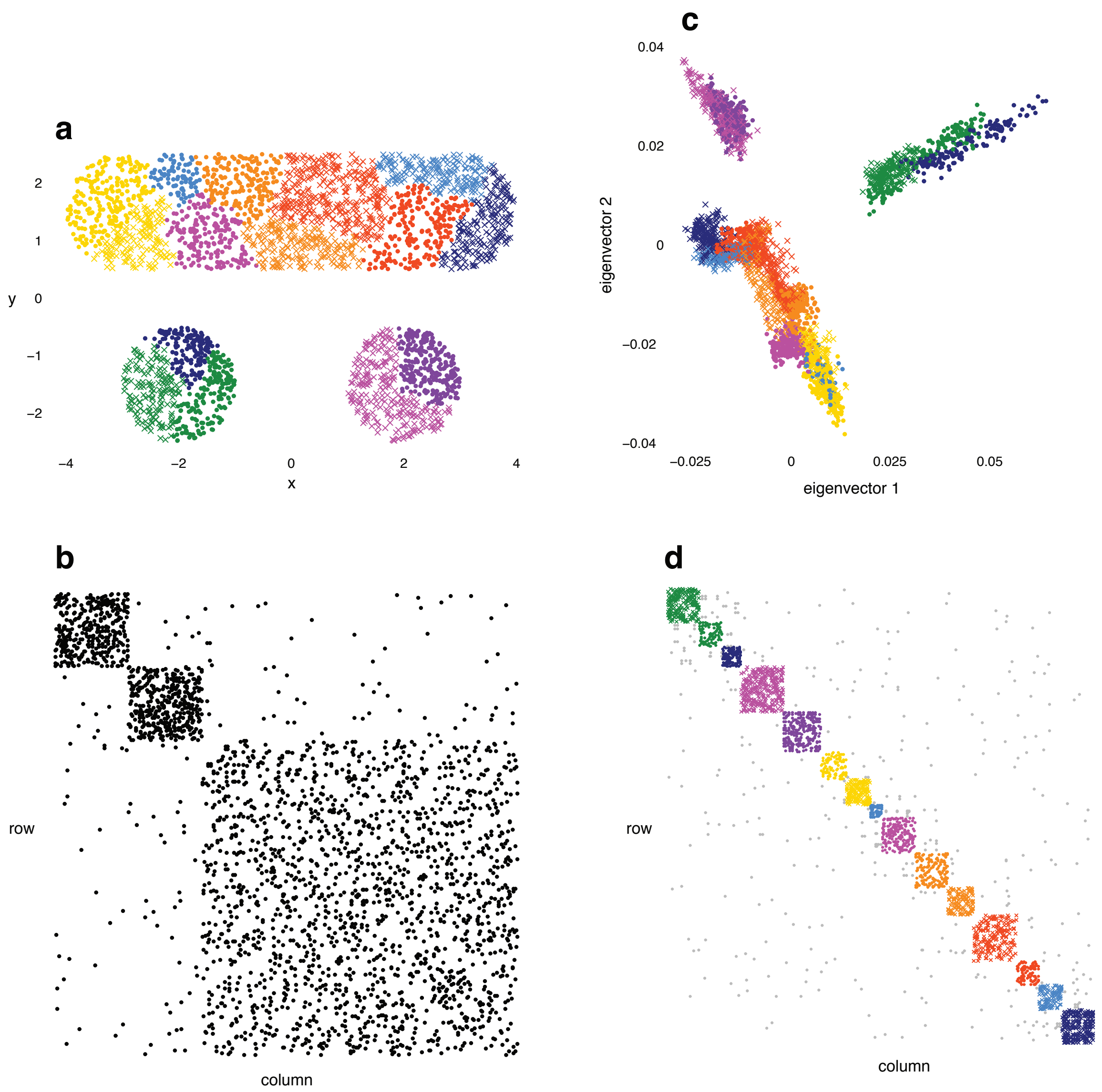

Supplementary Figure 6 | Illustration of community detection and spectral analysis in a simulated data set. Panels a–d summarize the results of running the community detection (multi-level) algorithm—the algorithm used to generate the hierarchical clustering—and spectral analysis (Laplacian eigenmaps method) on a small, simulated data set. The data set is generated by first drawing co-ordinates (x_i, y_i) uniformly at random from 3 geographic regions, representing 3 discrete populations with low connectivity (e.g., genetic relatedness) between them: the unit circle centered at (–2, –1.5); the unit circle centered at (2, –1.5); and a box [-4, 4] × [0.5, 2.5] with rounded corners. In total, n = 3,000 points are drawn, in which 500 are from population 1, 500 are from population 2, and 2,000 are from population 3. These 3,000 points are depicted in Panel a. The network, or undirected graph, is defined from these data in the following way: (i, j) is an edge in the graph if and only if i is among the 25 nearest neighbors of j, or if j is among the 25 nearest neighbors of i, in which "nearest neighbor" is determined by Euclidean distance. All edges are assumed to have a weight of 1. In this way, points nearest to each other in Panel a are connected to each other in the network. Additionally, 0.1% of pairs are

connected uniformly at random if they aren't already connected by an edge. Panel b shows the (symmetric) adjacency matrix of the undirected graph for 500 randomly chosen vertices of this graph. (Note that the diagonal of this adjacency matrix is set to 1 for the Laplacian eigenmaps method.) After generating these data, we apply the community detection (Panel d) and spectral analysis methods (Panel c) to these data. The community detection algorithm subdivides the network into 15 communities, or clusters; the assignment of samples to these 15 clusters is depicted by different colors and shapes in Panel a, c and d. These 15 clusters accurately capture the 3 populations because there are few connections between these populations, but it also subdivides each of the 3 populations in such a way that samples nearby each other are usually included in the same cluster. Panel d shows the sample adjacency matrix, again for a random subset of 500 vertices, in which the vertices are arranged by assignment to the 15 clusters. Despite the apparent arbitrariness of this clustering, it is consistent with the aim of maximizing the modularity of the network, as the density of connections between the detected clusters is relatively small. In Panel c, the dominant structure captured by the spectral analysis—specifically, the first 2 eigenvectors of the Laplacian—is the separation of the points into the 3 populations. Although the spectral embedding also captures structure within population 3, specifically the location in population 3 along the horizontal (x) axis, this is a less dominant feature in the embedding. In summary, this example illustrates that the community detection method identifies clusters that capture both discrete and continuous population structure (e.g., isolation-by-distance), whereas the spectral analysis can be used to isolate discrete population structure.

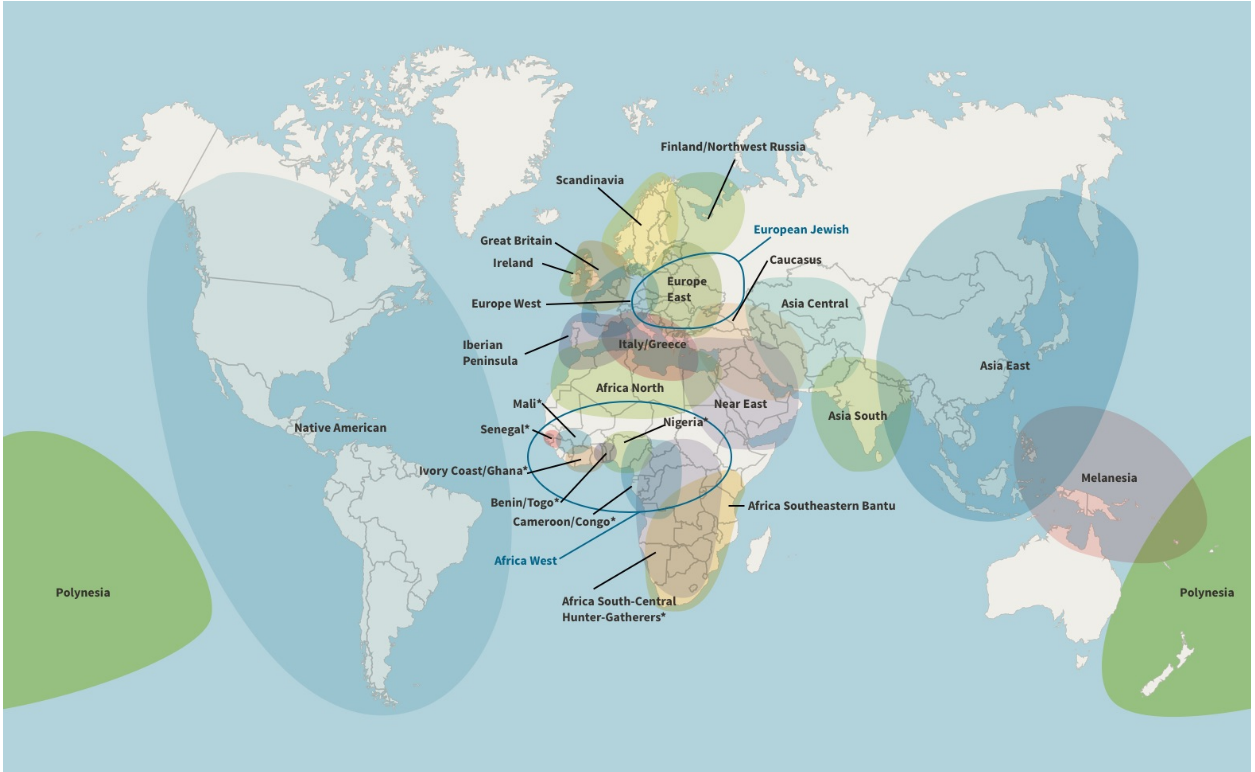

Supplementary Figure 7 | Geographic regions corresponding to ancestral populations in global ancestry reference panel. See Supplementary Table 2 for composition the panel. *Admixture proportions for these regions are collapsed into a single admixture proportion representing West Africa. Map and figure designed by AncestryDNA.

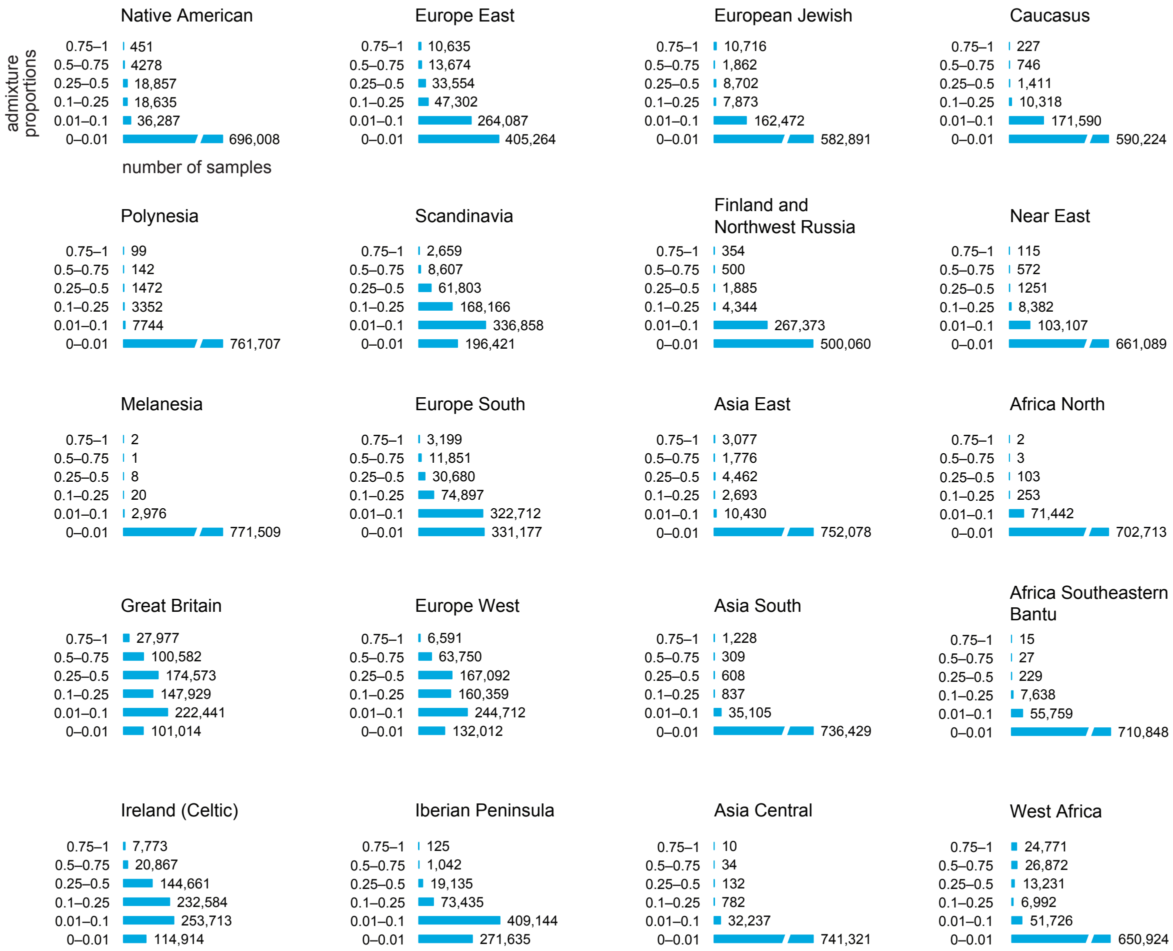

Supplementary Figure 8 | Global summary of estimated admixture proportions.

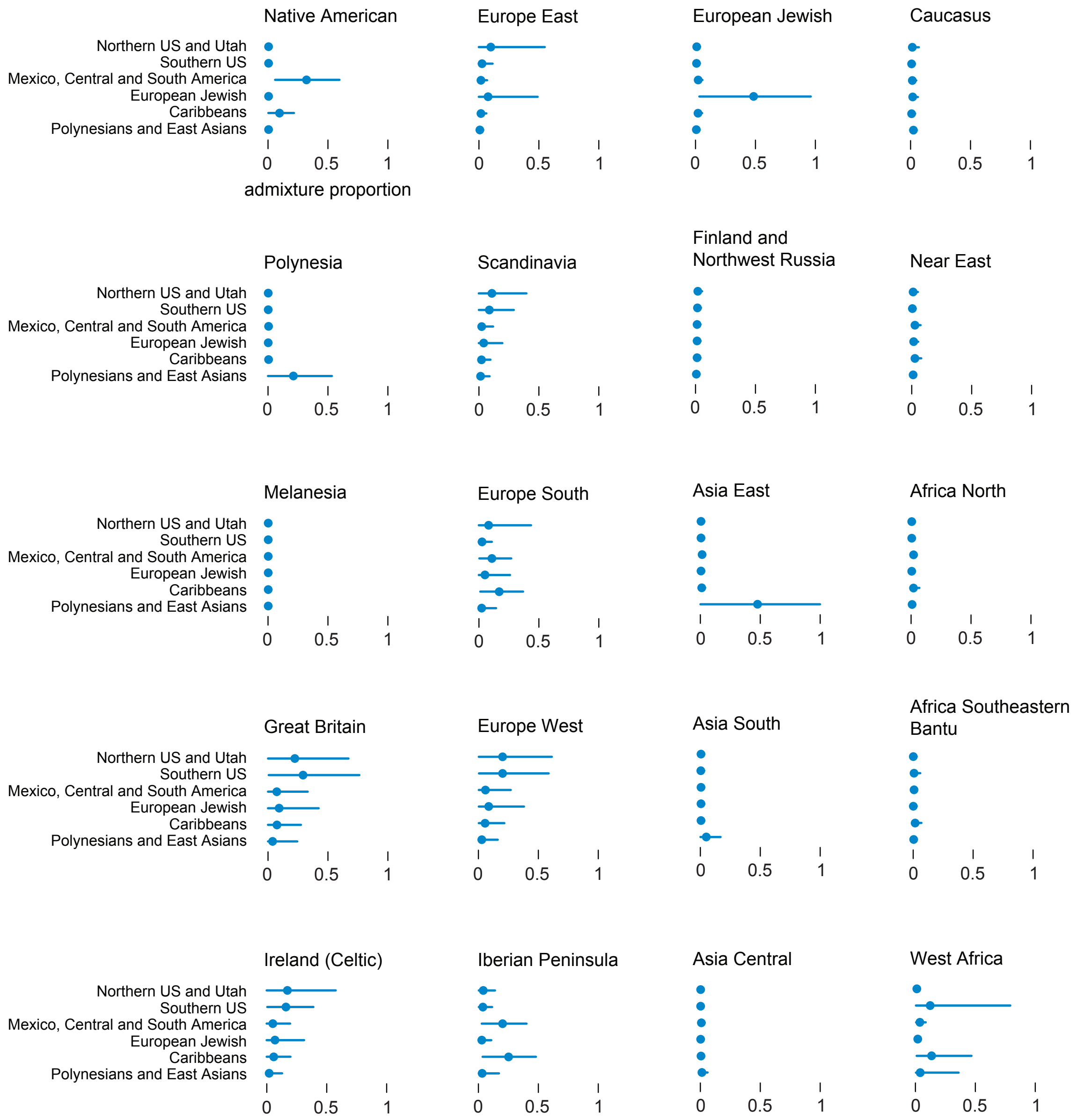

Supplementary Figure 9 | Admixture proportions in top-level IBD network clusters. Filled circles correspond to mean admixture proportions, and error bars give [0.05,0.95] credible intervals.

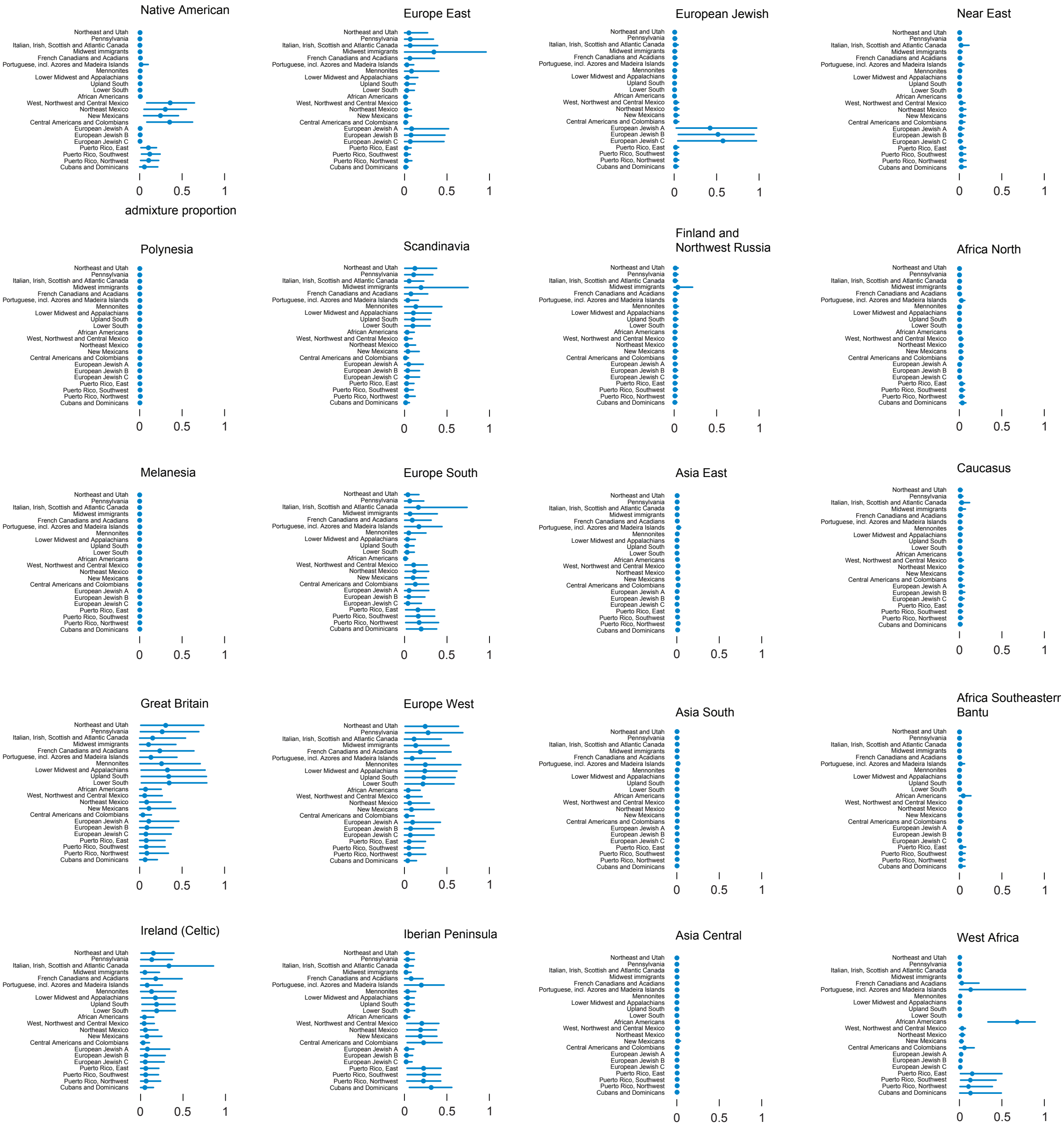

Supplementary Figure 10 | Admixture proportions in IBD sub-network clusters. Filled circles correspond to mean admixture proportions, and error bars give [0.05,0.95] credible intervals.

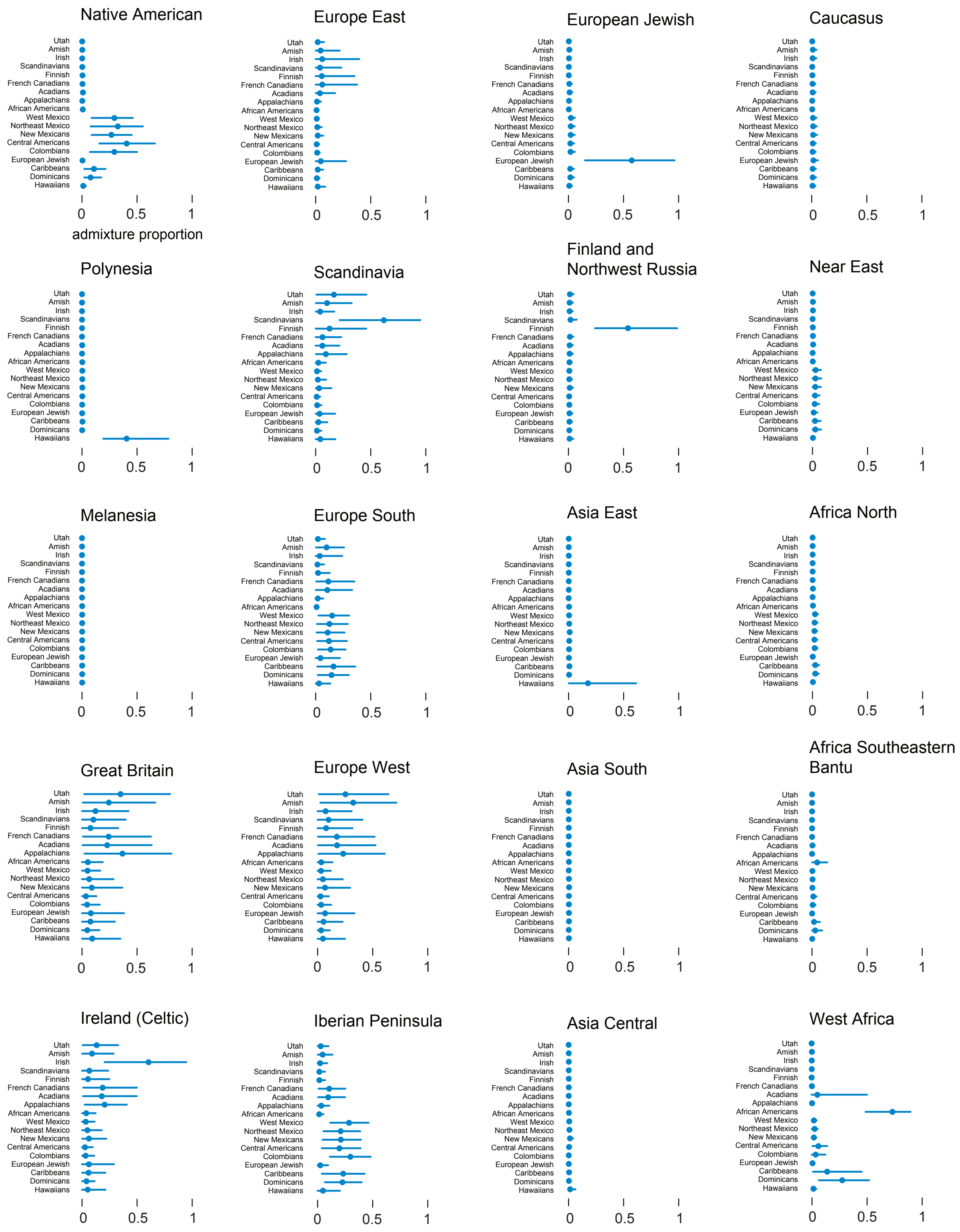

Supplementary Figure 11 | Admixture proportions in stable subsets identified from spectral analysis. Filled circles correspond to mean admixture proportions, and error bars give [0.05,0.95] credible intervals.

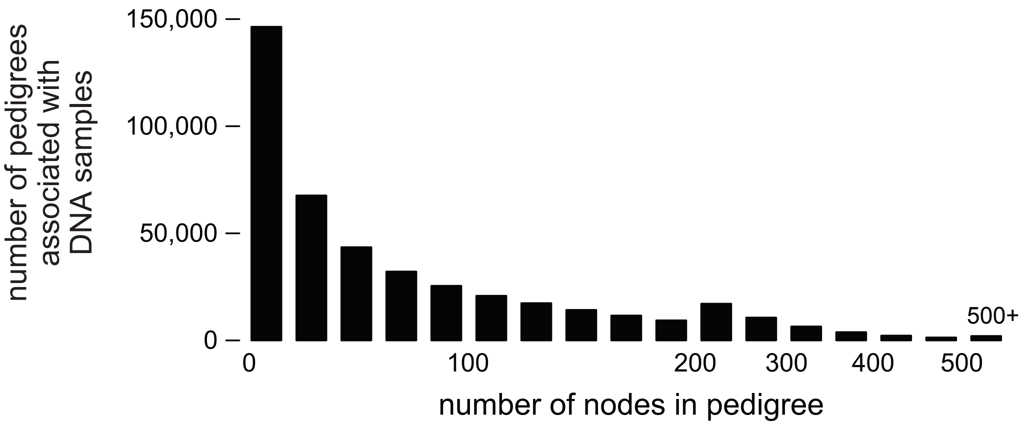

Supplementary Figure 12 | Distribution of pedigree sizes. Plot shows empirical distribution of the number of nodes in a pedigree linked to a DNA sample. Note that the histogram bins are wider for pedigrees with more than 200 nodes.

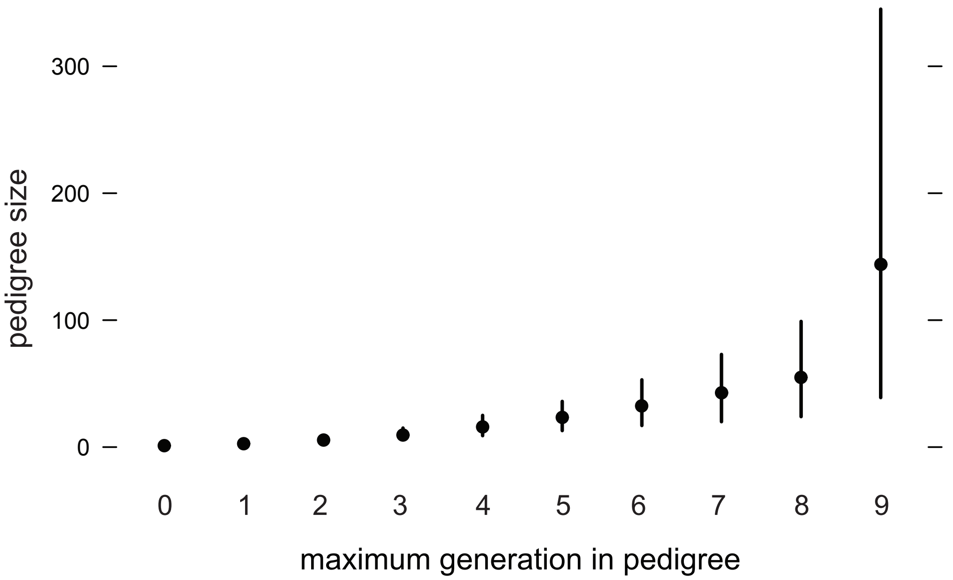

Supplementary Figure 13 | Pedigree size versus pedigree depth. Plot shows empirical distribution of pedigree size (number of nodes in pedigree) stratified by pedigree depth (maximum generation represented in pedigree) for pedigrees linked to DNA samples. Only one node in a pedigree is generation 0—this is the node corresponding to the DNA sample. Each dot at the center corresponds to the mean, and the endpoints of the vertical bars represent the 5% and 95% empirical quantiles.

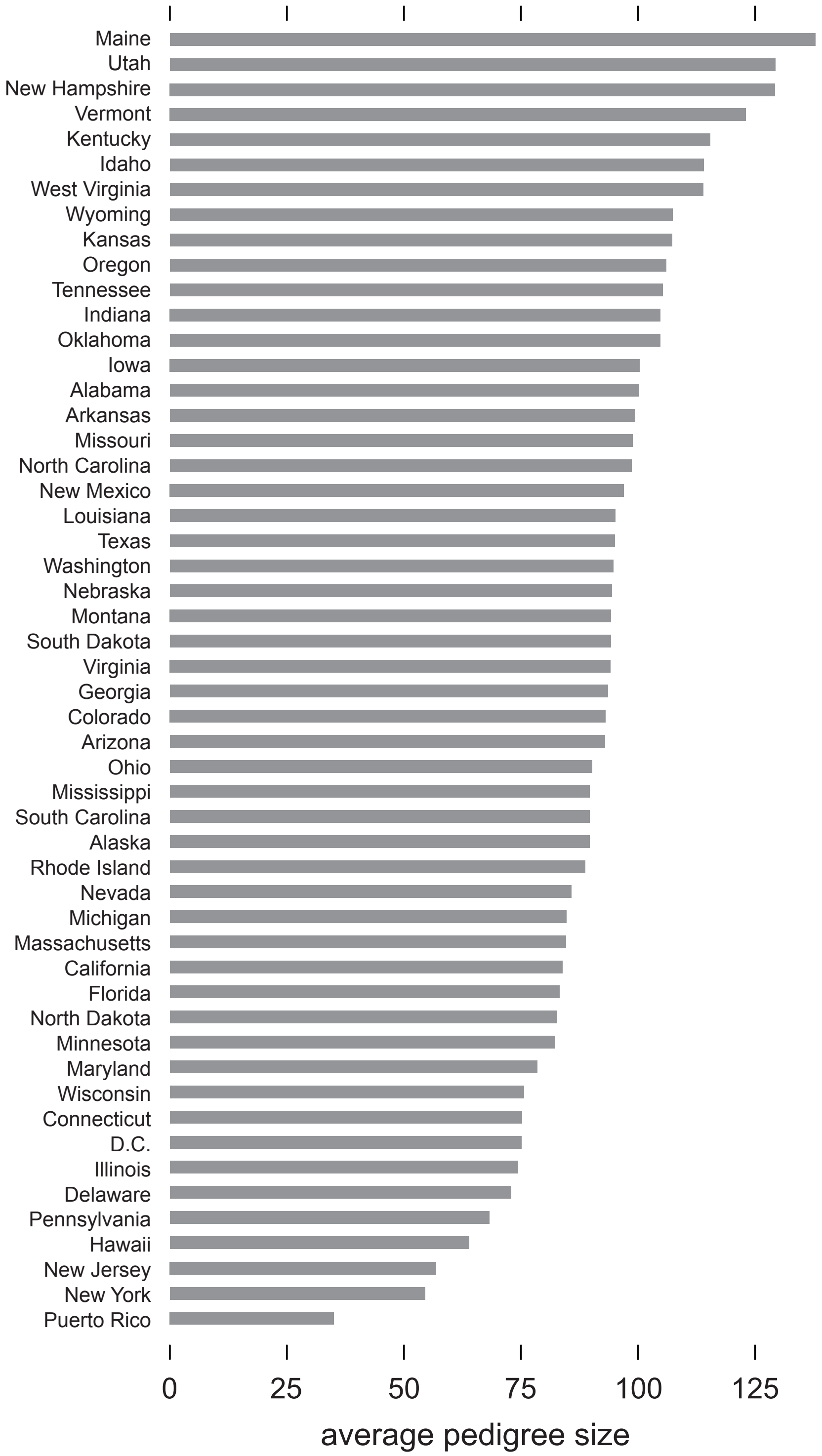

Supplementary Figure 14 | Average pedigree size by US state. These numbers are based on counts of pedigree nodes linked to DNA samples with a US birth location.

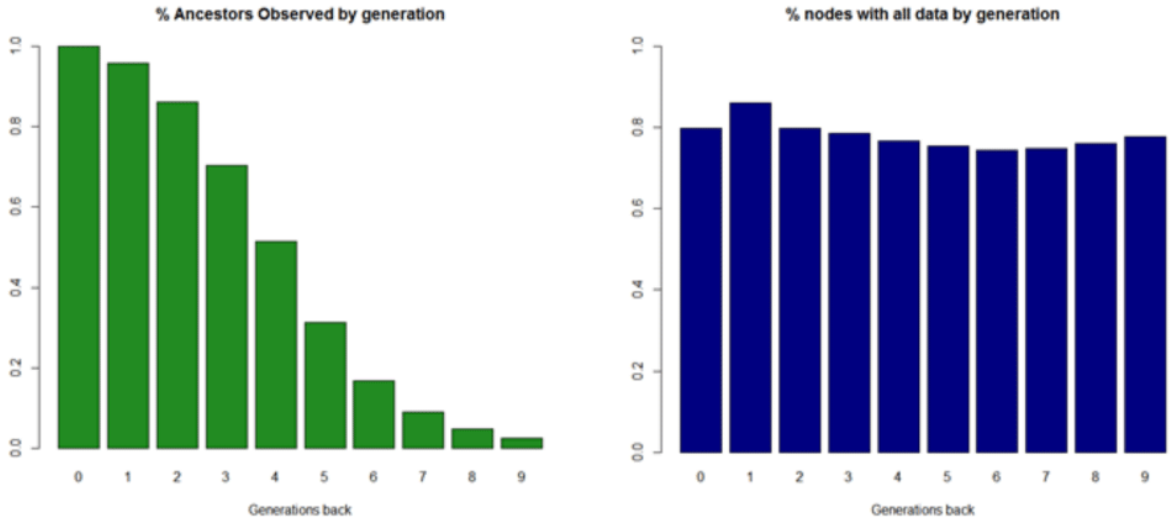

Supplementary Figure 15 | Completeness of pedigrees and pedigree annotations. *Left-hand panel:* The number of pedigree nodes per generation as a proportion of the maximum number of possible pedigree nodes in that generation. *Right-hand panel:* For each generation of the pedigree, proportion of nodes annotated with birth location, birth year and surname.

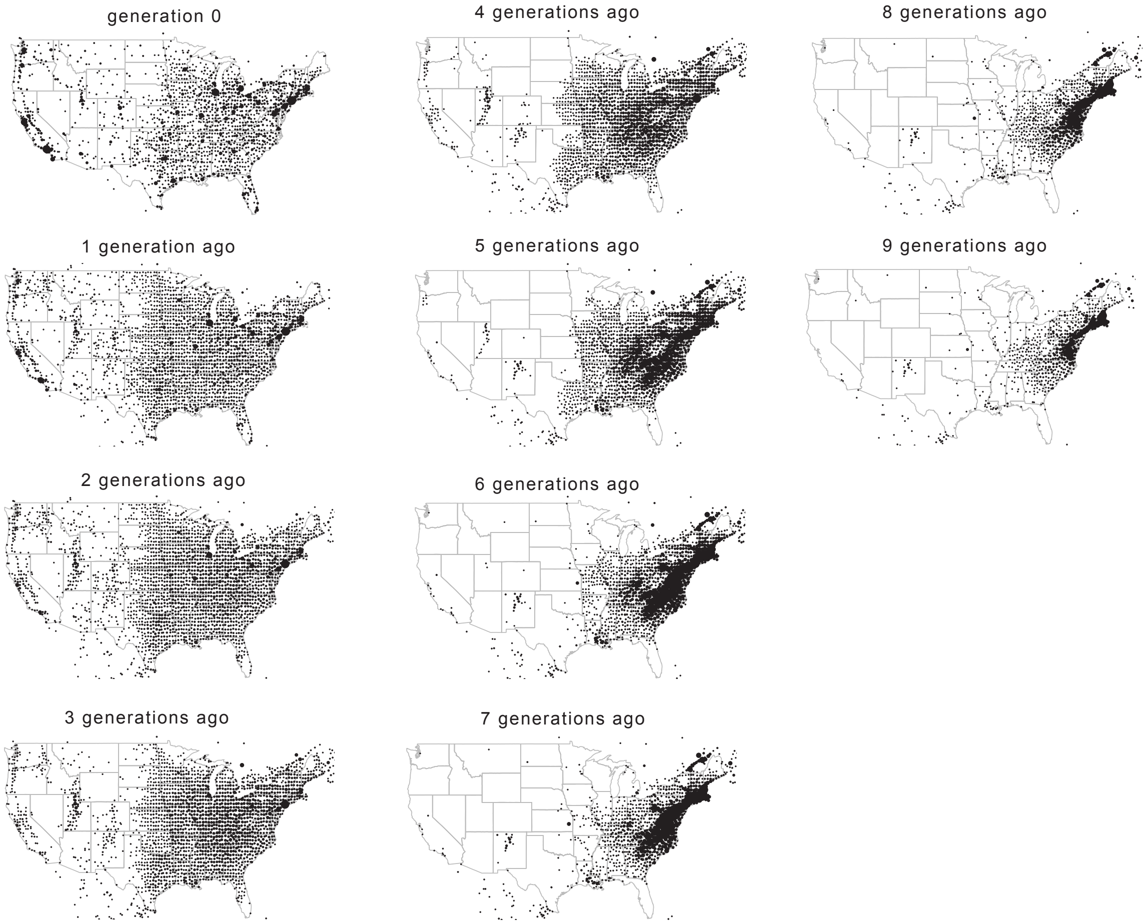

Supplementary Figure 16 | Distribution of birth locations in continental US, by generation. Generation 0 corresponds to the DNA sample, so should reflect the birth location distribution of (present-day) US-born AncestryDNA customers. Generation 1 corresponds to parents, generation 2 corresponds to grandparents, and so on. Ancestral birth locations in each pedigree generation are divided into a grid, with grid points every 0.5 degrees of latitude and longitude. Only locations with at least 20 pedigree nodes are shown on the map. The size of each point is scaled by the number of annotated pedigree nodes at that location, separately for each generation. All maps in our figures were generated with the *maps* R package using data from the Natural Earth Project (1:50m world map, version 2.0). These data are made available in the public domain (Creative Commons CC0).

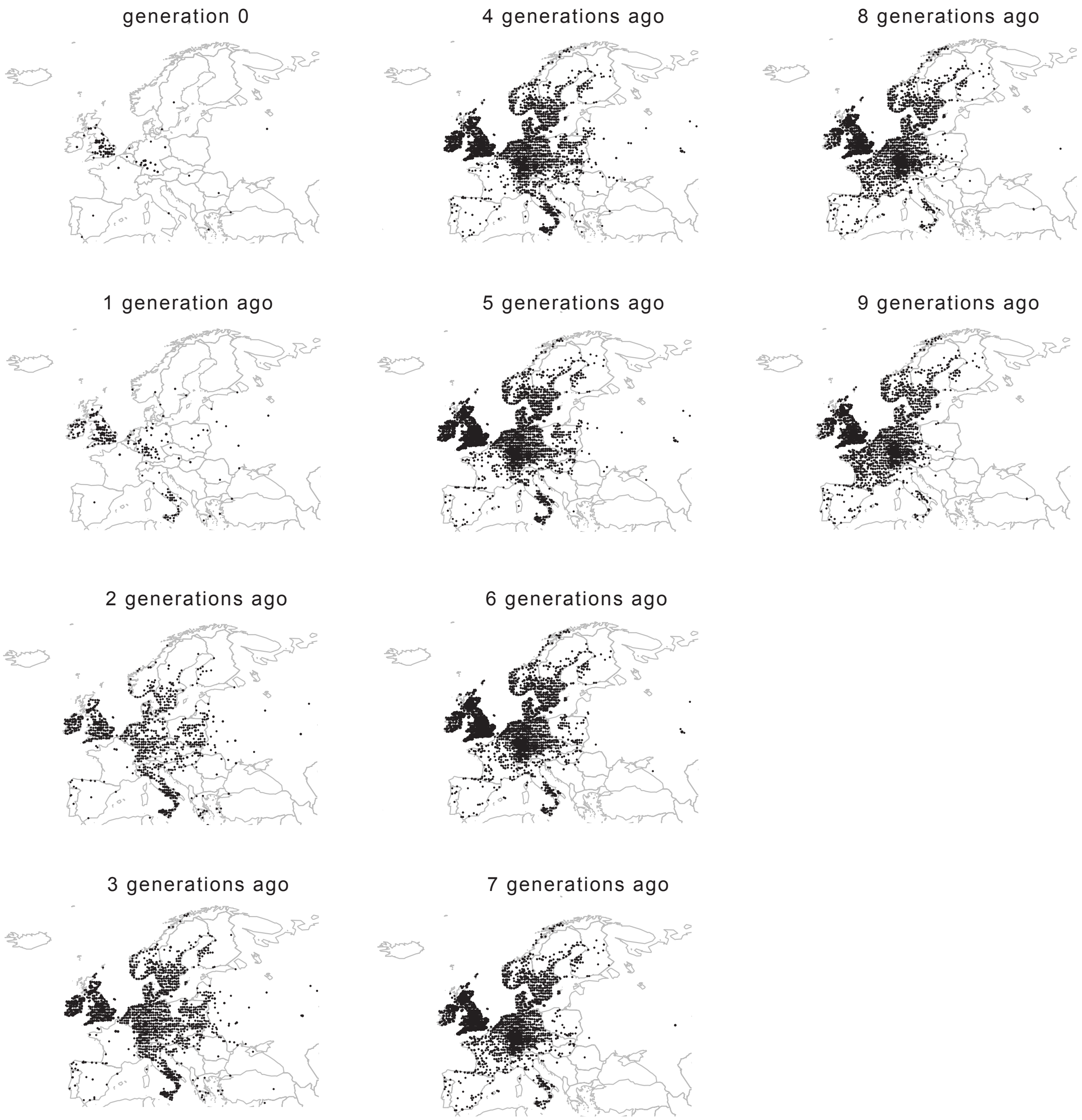

Supplementary Figure 17 | Distribution of birth locations in Europe, by generation. See description of Supplementary Fig. 16 for details. All maps in our figures were generated with the *maps* R package using data from the Natural Earth Project (1:50m world map, version 2.0). These data are made available in the public domain (Creative Commons CC0).

A

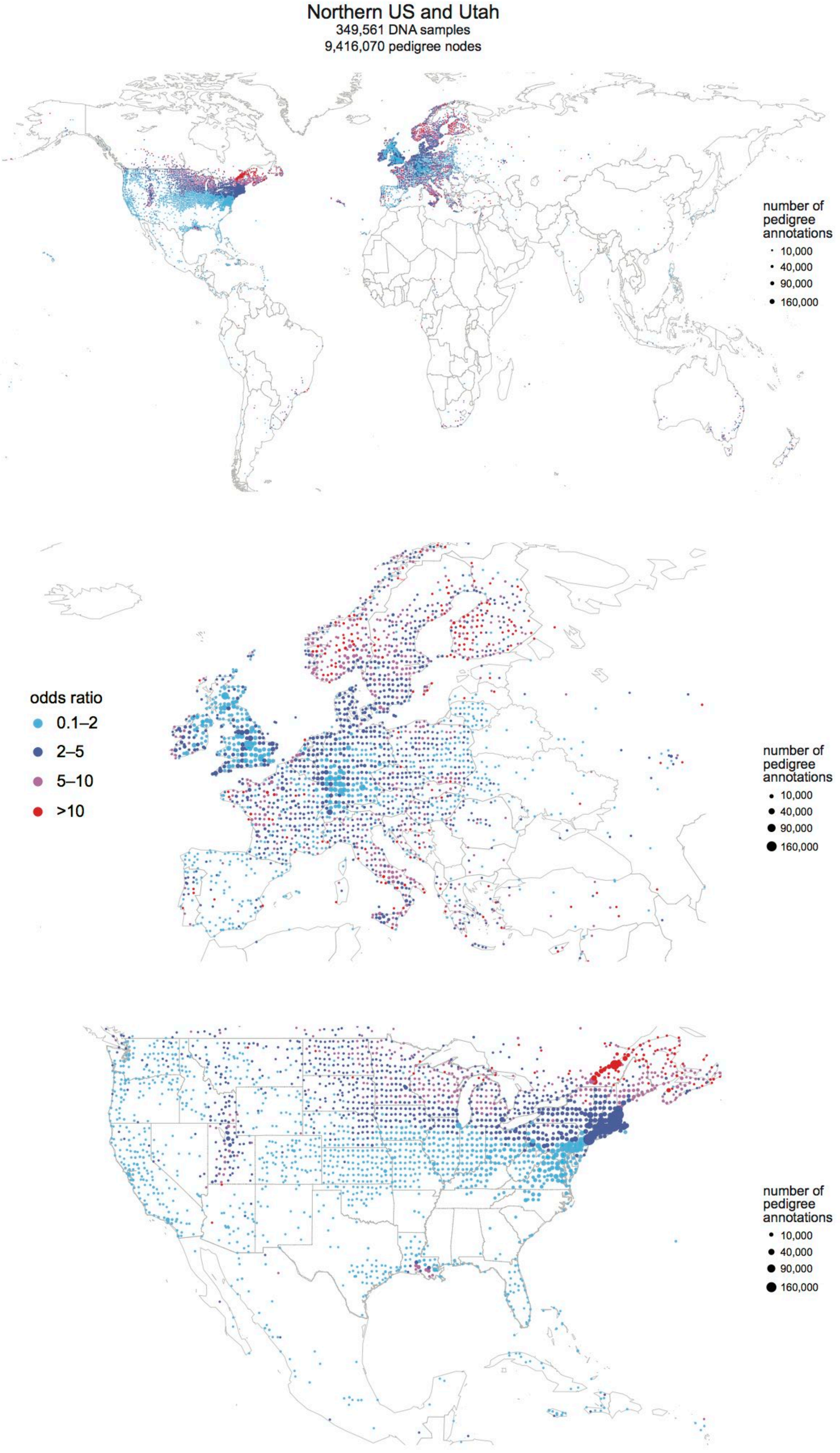
Northern US and Utah
349,561 DNA samples
9,416,070 pedigree nodes
number of pedigree annotations
10,000
40,000
90,000
160,000
odds ratio
0.1–2
2–5
5–10
>10
number of pedigree annotations
10,000
40,000
90,000
160,000
number of pedigree annotations
10,000
40,000
90,000
160,000

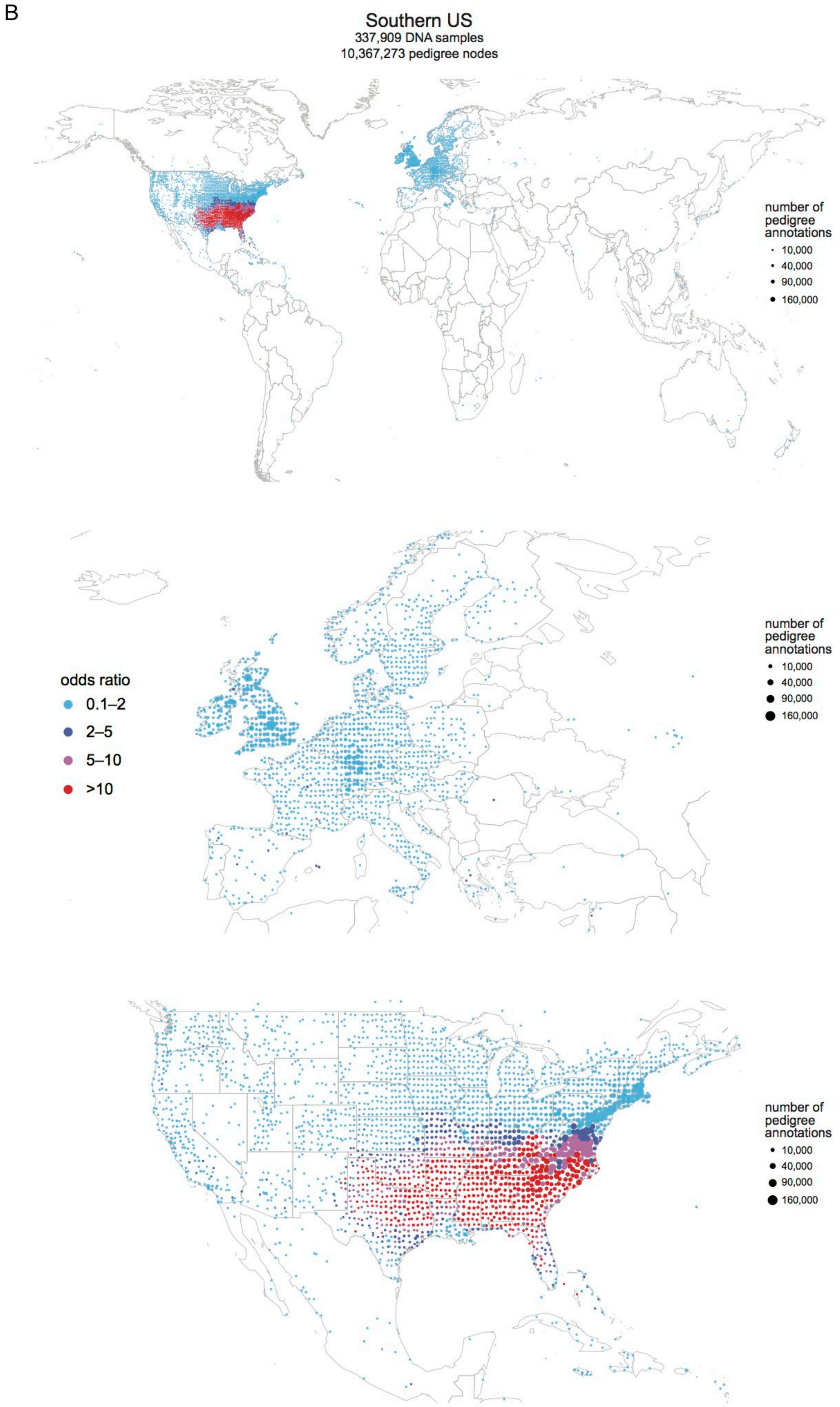

B
Southern US
337,909 DNA samples
10,367,273 pedigree nodes
number of
pedigree
annotations
10,000
40,000
90,000
160,000
odds ratio
0.1–2
2–5
5–10
>10
number of
pedigree
annotations
10,000
40,000
90,000
160,000
number of
pedigree
annotations
10,000
40,000
90,000
160,000

C

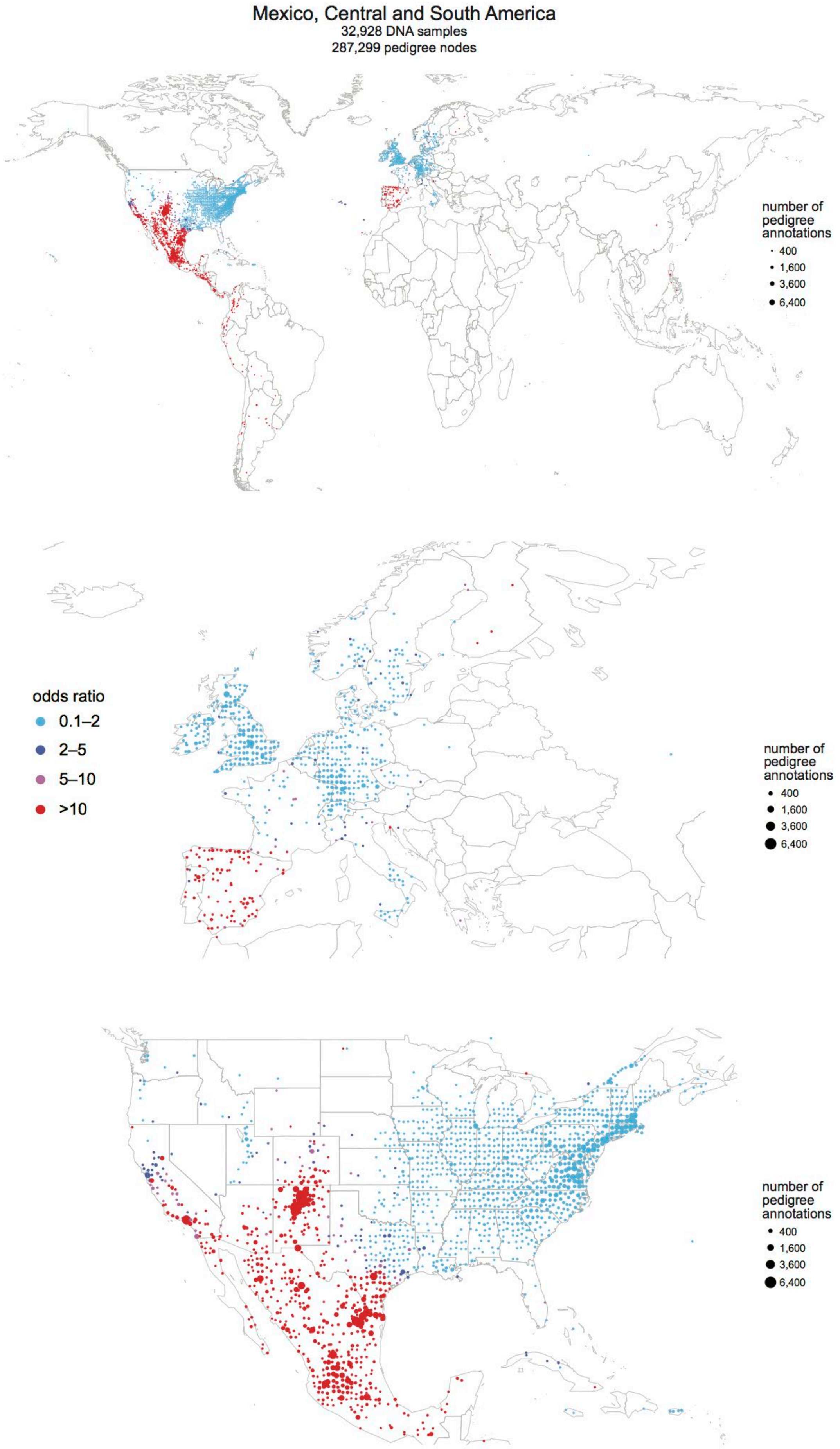
Mexico, Central and South America
32,928 DNA samples
287,299 pedigree nodes
number of pedigree annotations
400
1,600
3,600
6,400
odds ratio
0.1–2
2–5
5–10
>10
number of pedigree annotations
400
1,600
3,600
6,400
number of pedigree annotations
400
1,600
3,600
6,400

D

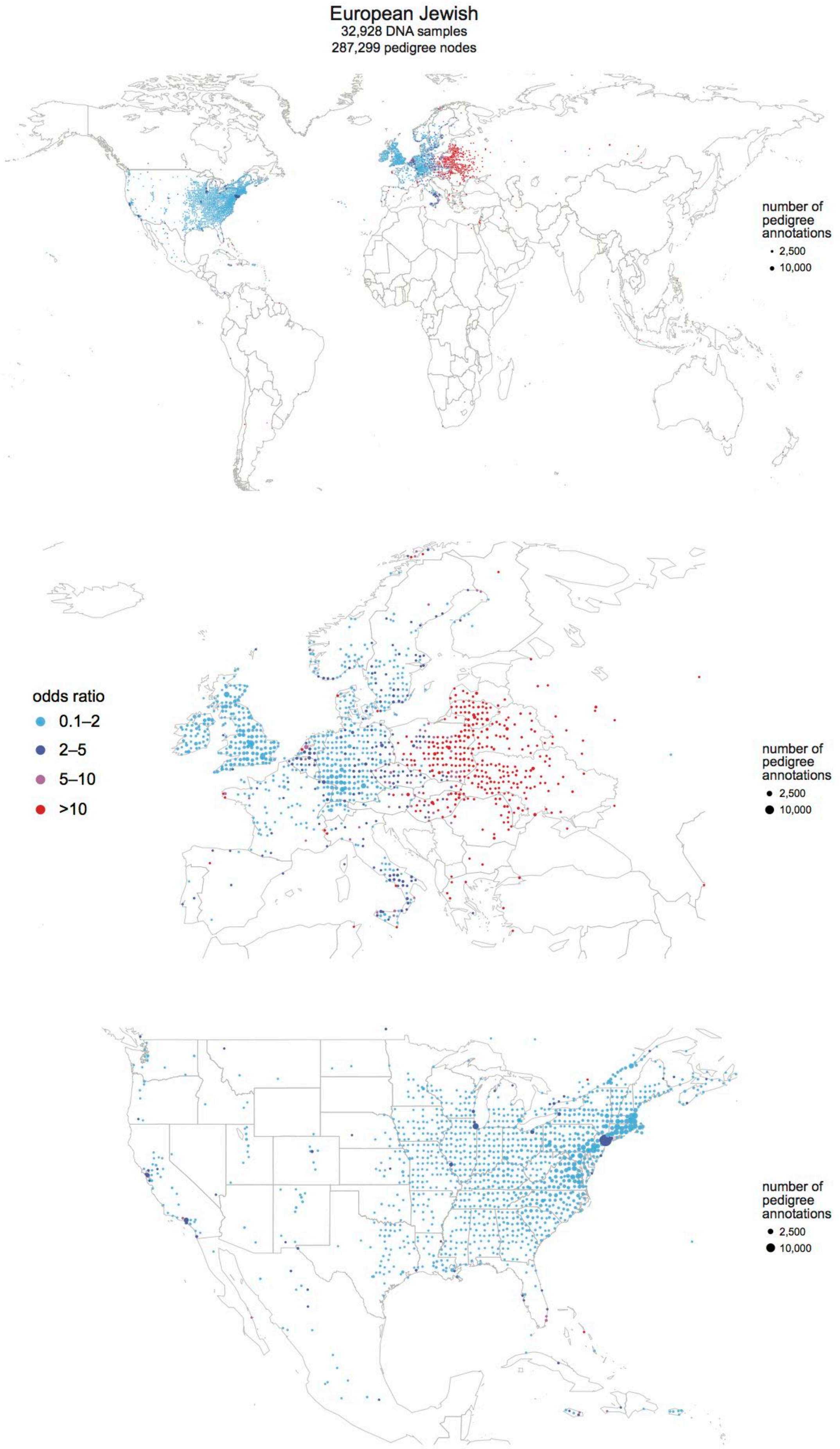
European Jewish
32,928 DNA samples
287,299 pedigree nodes
number of pedigree annotations
2,500
10,000
odds ratio
0.1–2
2–5
5–10
>10
number of pedigree annotations
2,500
10,000
number of pedigree annotations
2,500
10,000

E

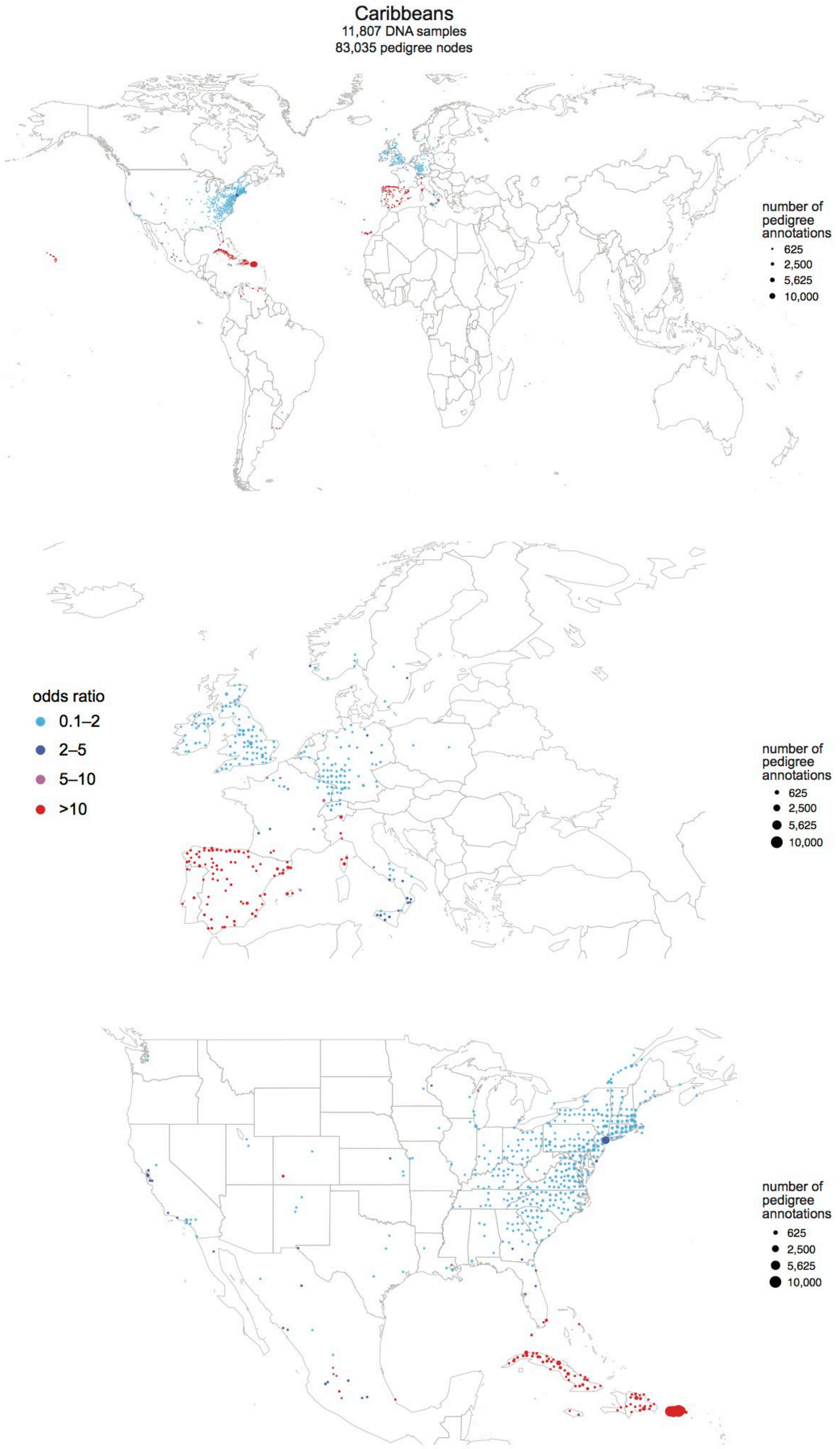
Caribbeans
11,807 DNA samples
83,035 pedigree nodes
number of pedigree annotations
625
2,500
5,625
10,000
odds ratio
0.1–2
2–5
5–10
>10
number of pedigree annotations
625
2,500
5,625
10,000
number of pedigree annotations
625
2,500
5,625
10,000

F

Polynesians and East Asians

3,845 DNA samples

16,190 pedigree nodes

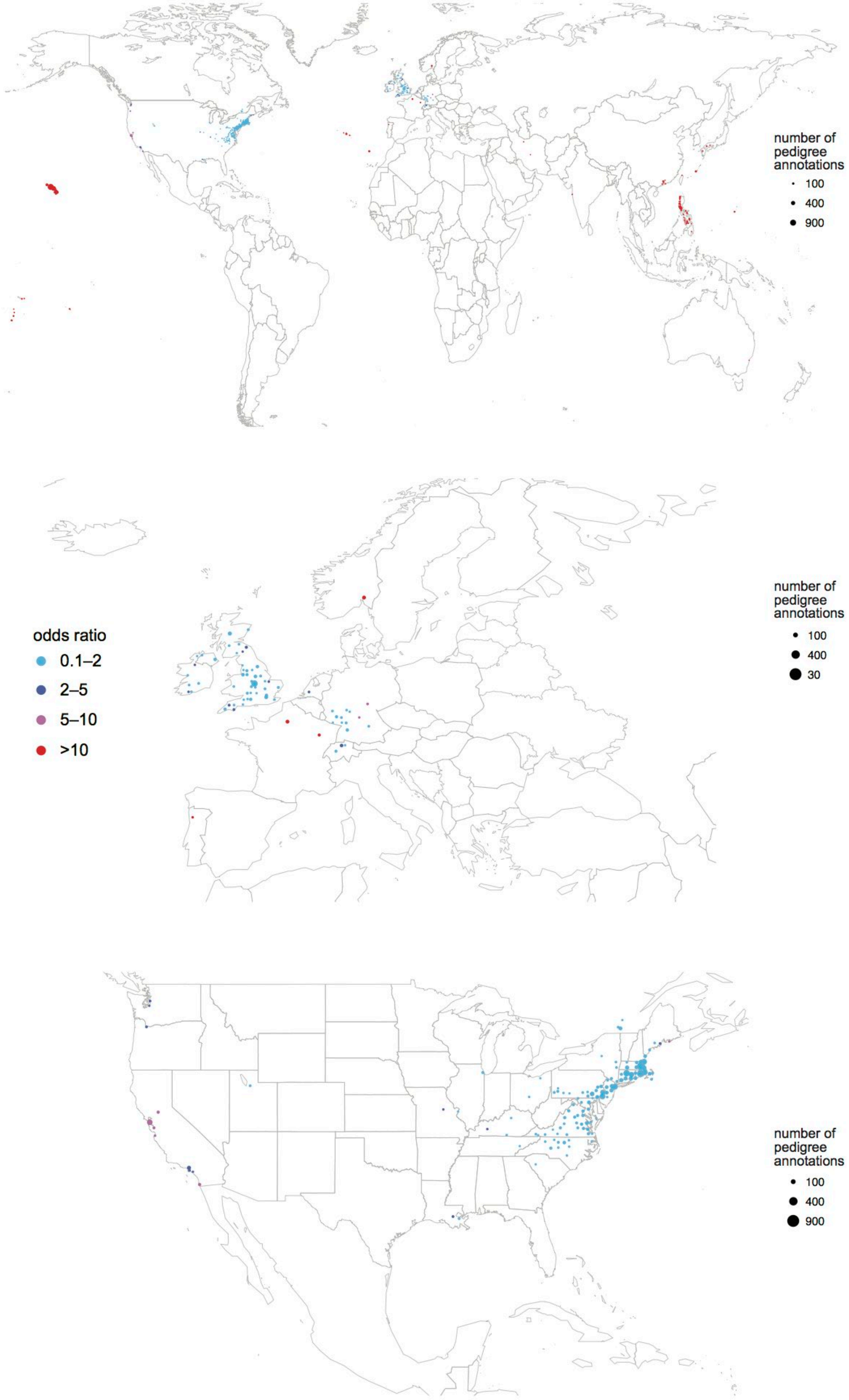

Supplementary Figure 18 | Distribution of ancestral birth locations worldwide, in North America, and in Europe, for each cluster detected in IBD network. Pedigree nodes (0–9 generations ago) annotated with birth locations are each converted to the nearest co-ordinate on a grid, with grid points every 0.5 degrees of latitude and longitude. Points are colored by *odds ratio* (*OR*)—the proportion of ancestral birth locations linked to cluster members at that map location over the proportion of ancestral birth locations linked to non-cluster members at the same location. A map location is plotted if at least 10 ancestors linked to cluster samples are born at that location, and if *OR* > 0.1. Points are scaled by the number of birth location annotations, separately in each map. Note that not all current political borders are shown in these maps. All maps in our figures were generated with the *maps* R package using data from the Natural Earth Project (1:50m world map, version 2.0). These data are made available in the public domain (Creative Commons CC0).

A

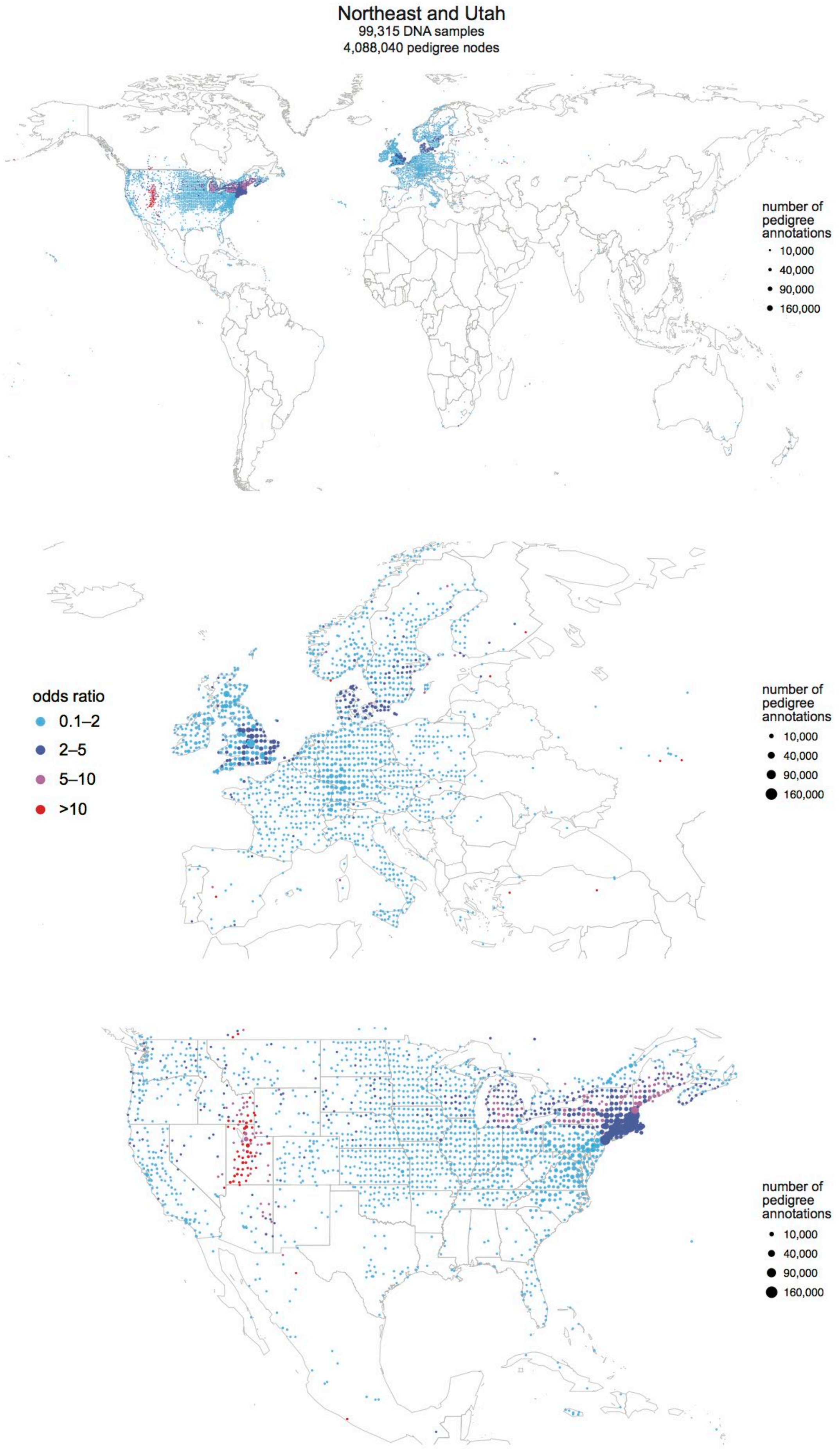
Northeast and Utah
99,315 DNA samples
4,088,040 pedigree nodes
number of pedigree annotations
10,000
40,000
90,000
160,000
odds ratio
0.1–2
2–5
5–10
>10
number of pedigree annotations
10,000
40,000
90,000
160,000
number of pedigree annotations
10,000
40,000
90,000
160,000

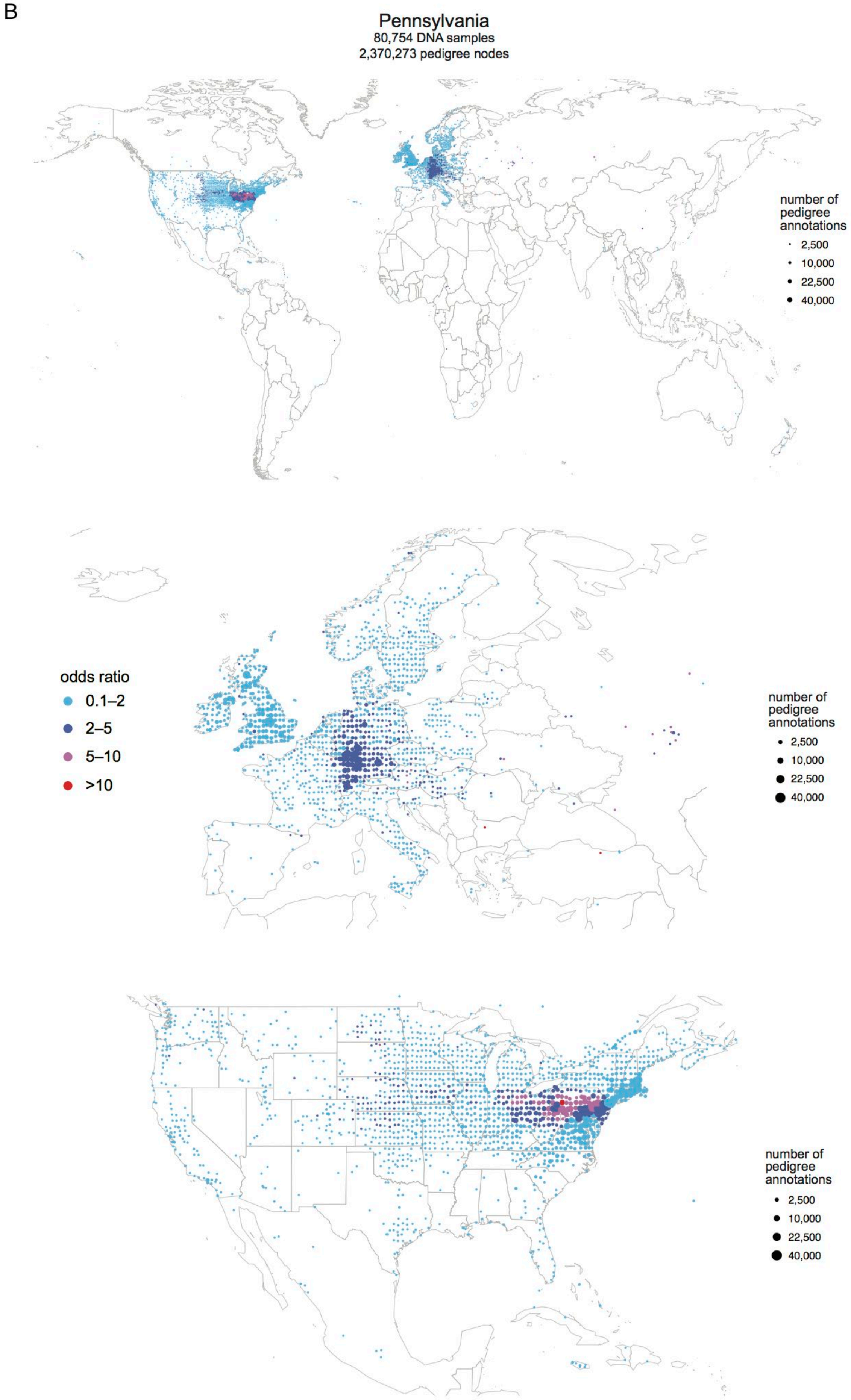
B
Pennsylvania
80,754 DNA samples
2,370,273 pedigree nodes
number of pedigree annotations
2,500
10,000
22,500
40,000
odds ratio
0.1–2
2–5
5–10
>10
number of pedigree annotations
2,500
10,000
22,500
40,000
number of pedigree annotations
2,500
10,000
22,500
40,000

C

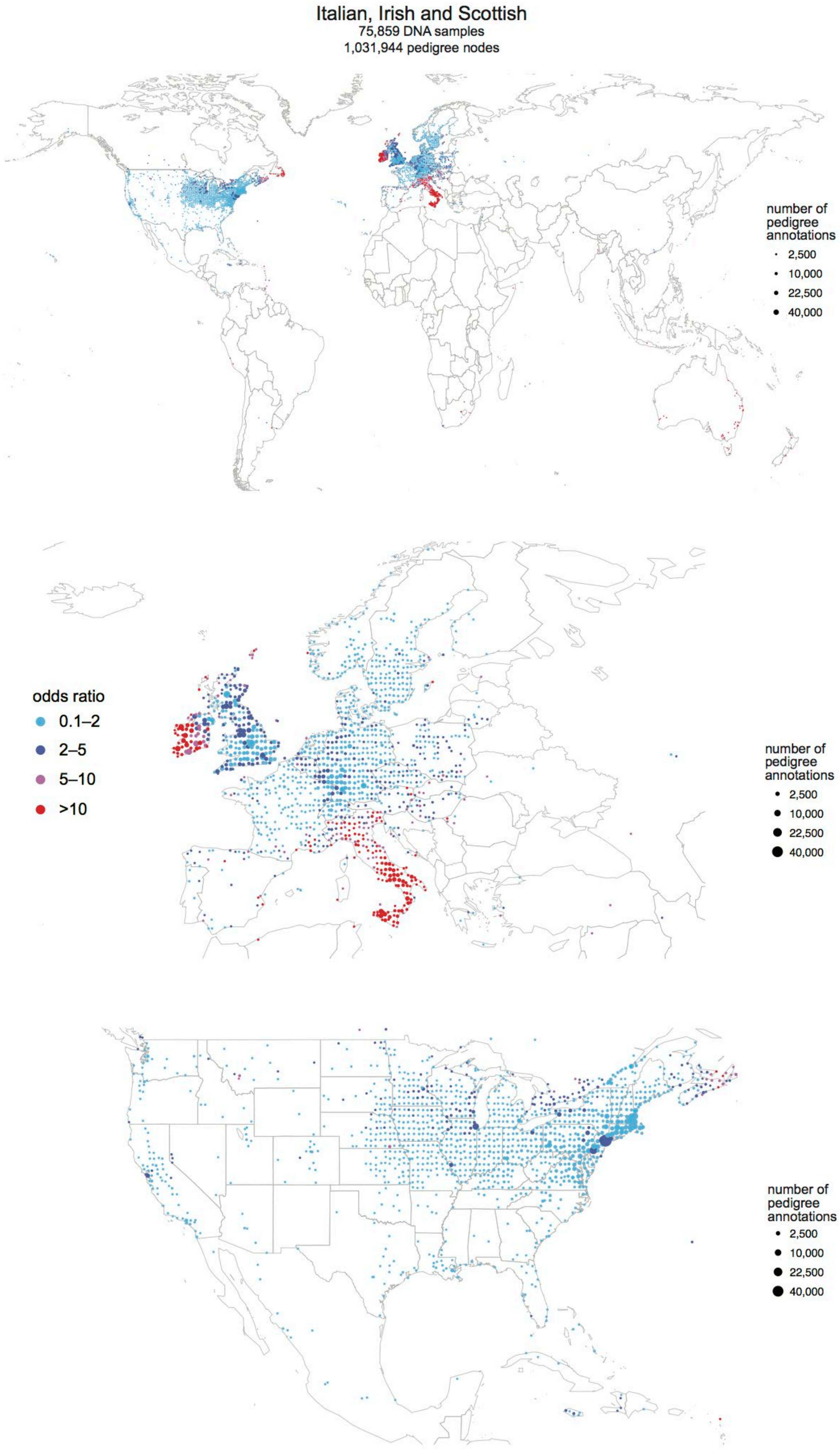
Italian, Irish and Scottish
75,859 DNA samples
1,031,944 pedigree nodes
number of pedigree annotations
2,500
10,000
22,500
40,000
odds ratio
0.1–2
2–5
5–10
>10
number of pedigree annotations
2,500
10,000
22,500
40,000
number of pedigree annotations
2,500
10,000
22,500
40,000

D

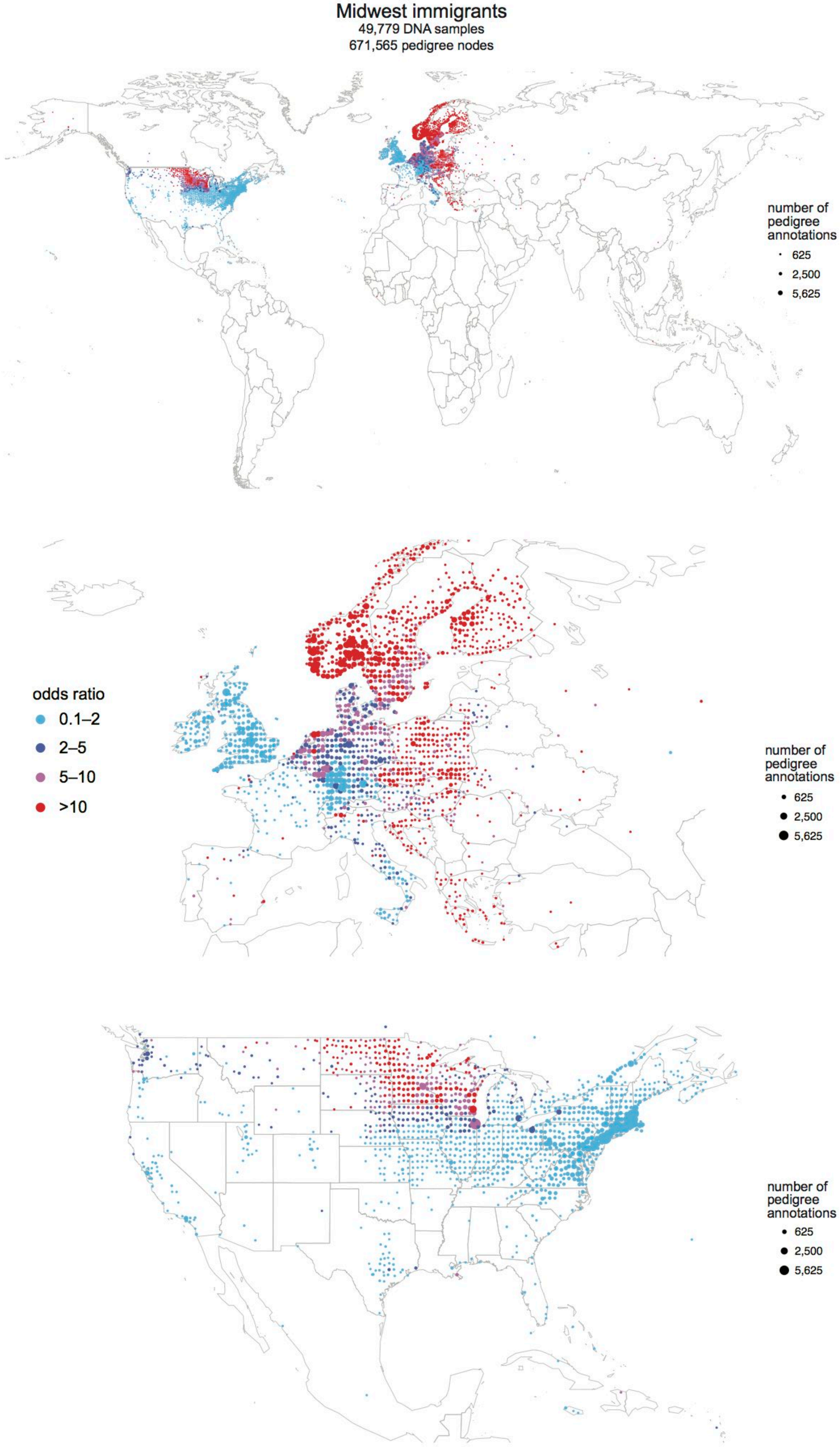
Midwest immigrants
49,779 DNA samples
671,565 pedigree nodes
number of pedigree annotations
625
2,500
5,625
odds ratio
0.1–2
2–5
5–10
>10
number of pedigree annotations
625
2,500
5,625
number of pedigree annotations
625
2,500
5,625

E

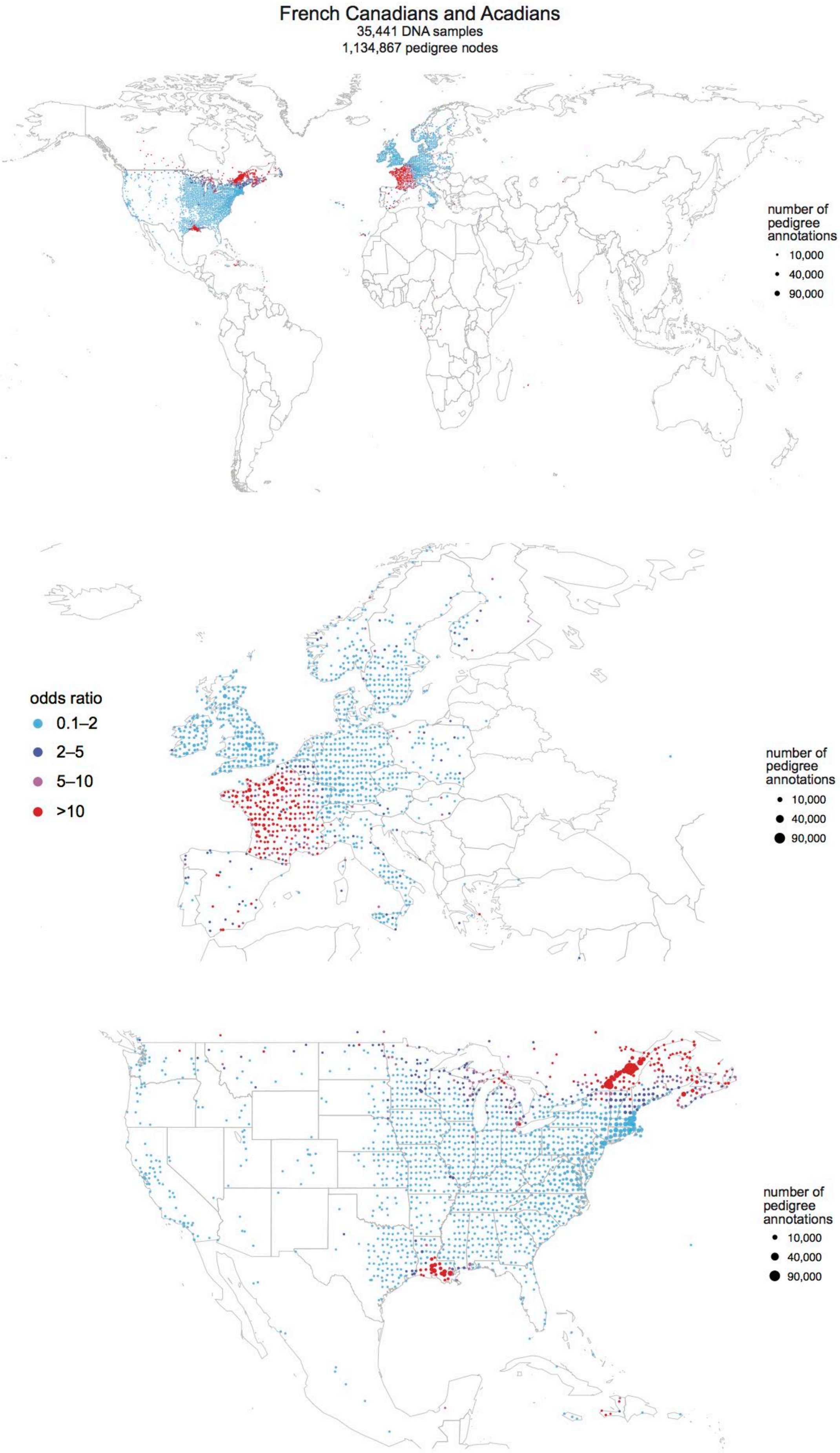
French Canadians and Acadians
35,441 DNA samples
1,134,867 pedigree nodes
number of pedigree annotations
10,000
40,000
90,000
odds ratio
0.1–2
2–5
5–10
>10
number of pedigree annotations
10,000
40,000
90,000
number of pedigree annotations
10,000
40,000
90,000

F

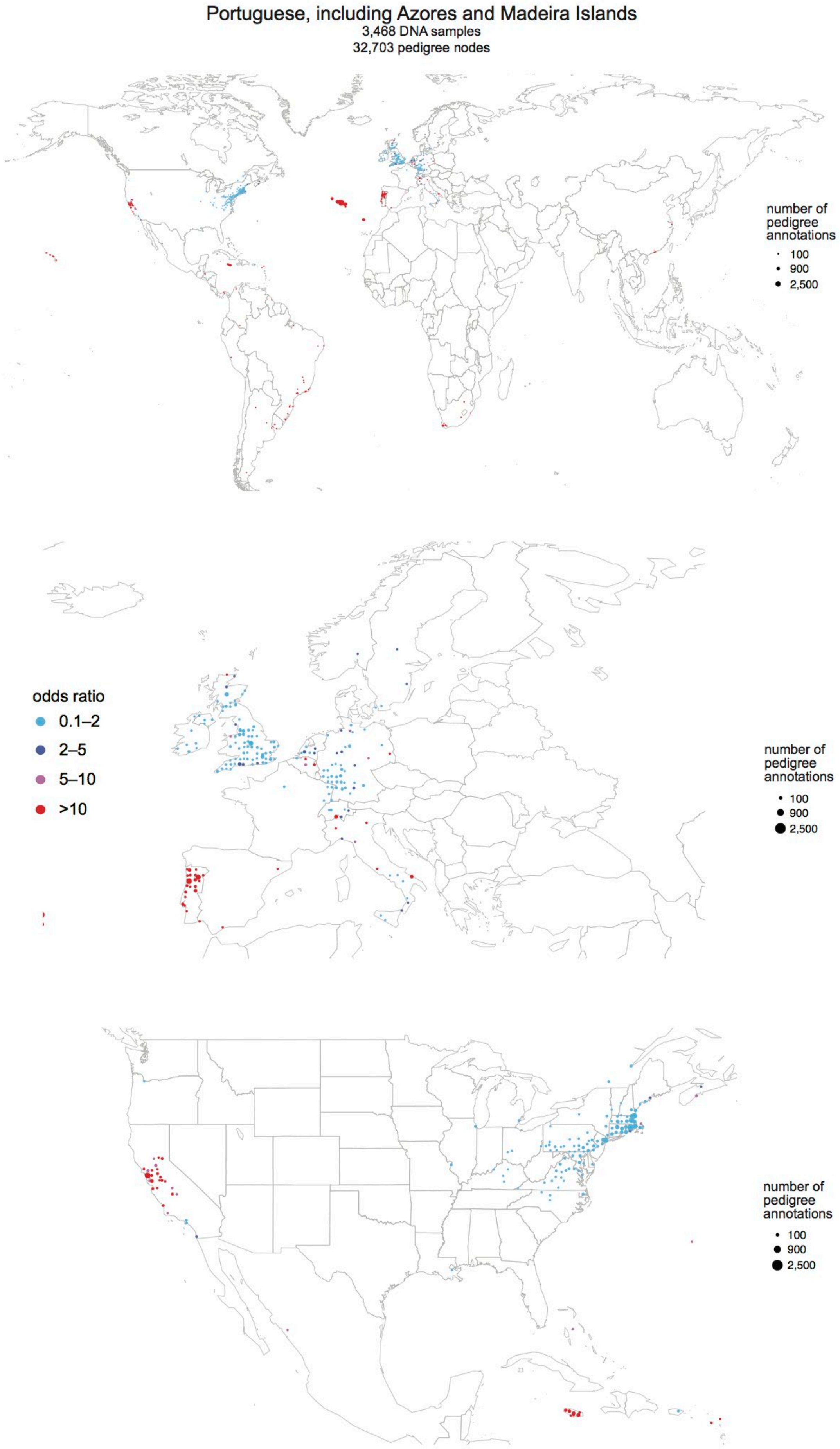
Portuguese, including Azores and Madeira Islands
3,468 DNA samples
32,703 pedigree nodes
number of pedigree annotations
100
900
2,500
odds ratio
0.1–2
2–5
5–10
>10
number of pedigree annotations
100
900
2,500
number of pedigree annotations
100
900
2,500

G

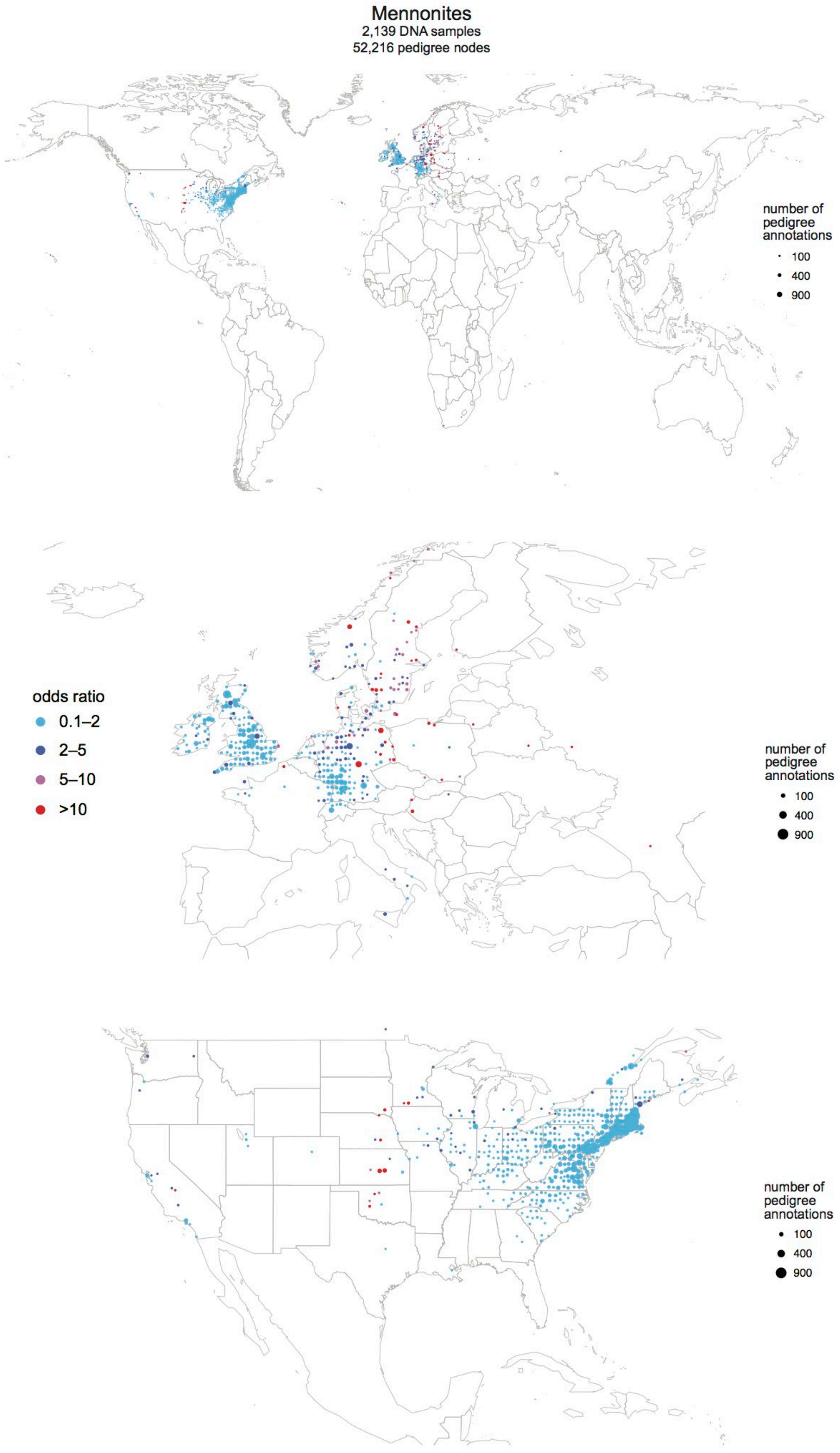
Mennonites
2,139 DNA samples
52,216 pedigree nodes
number of pedigree annotations
100
400
900
odds ratio
0.1–2
2–5
5–10
>10
number of pedigree annotations
100
400
900
number of pedigree annotations
100
400
900

H

Lower Midwest and Appalachians

108,786 DNA samples

4,131,104 pedigree nodes

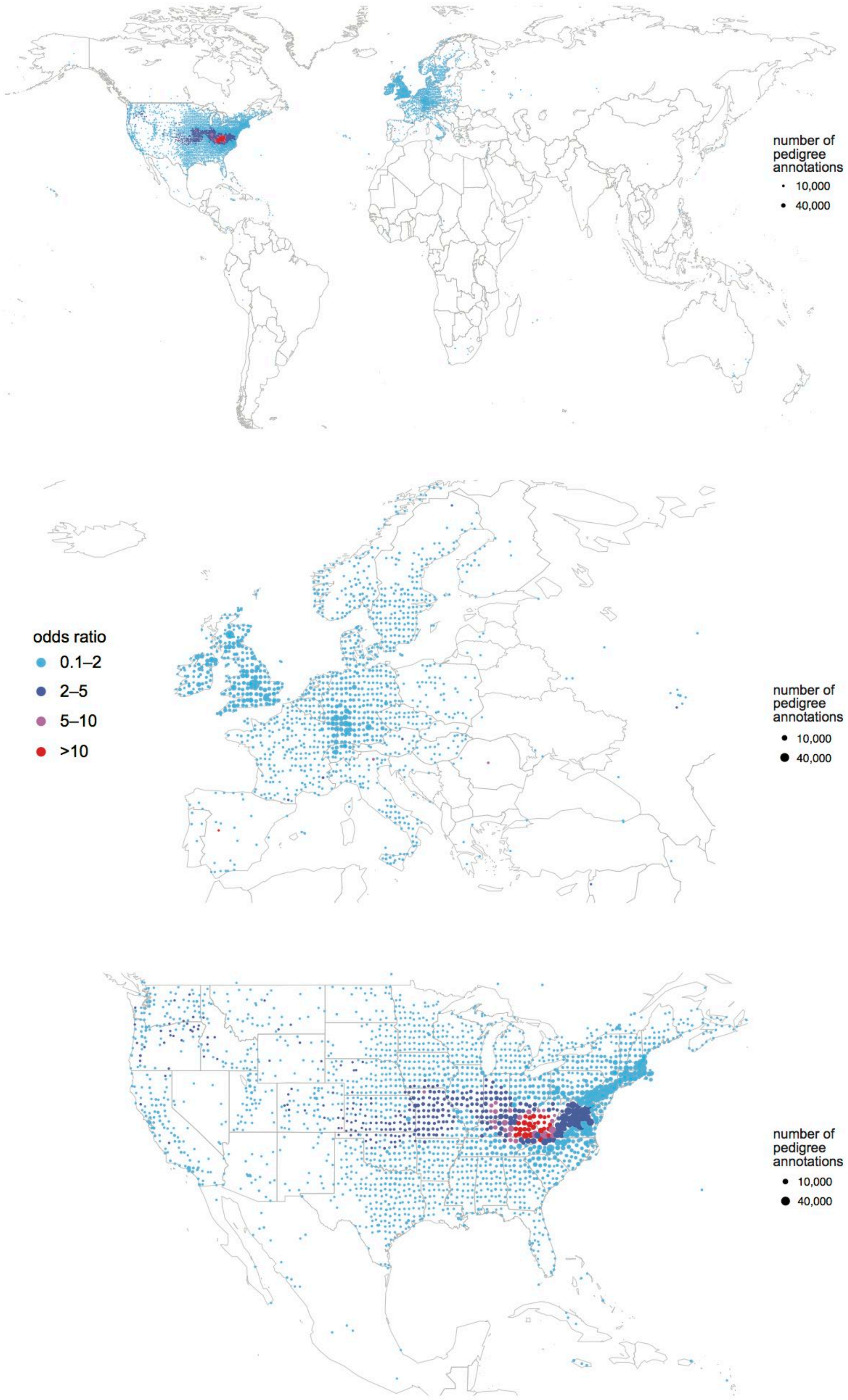

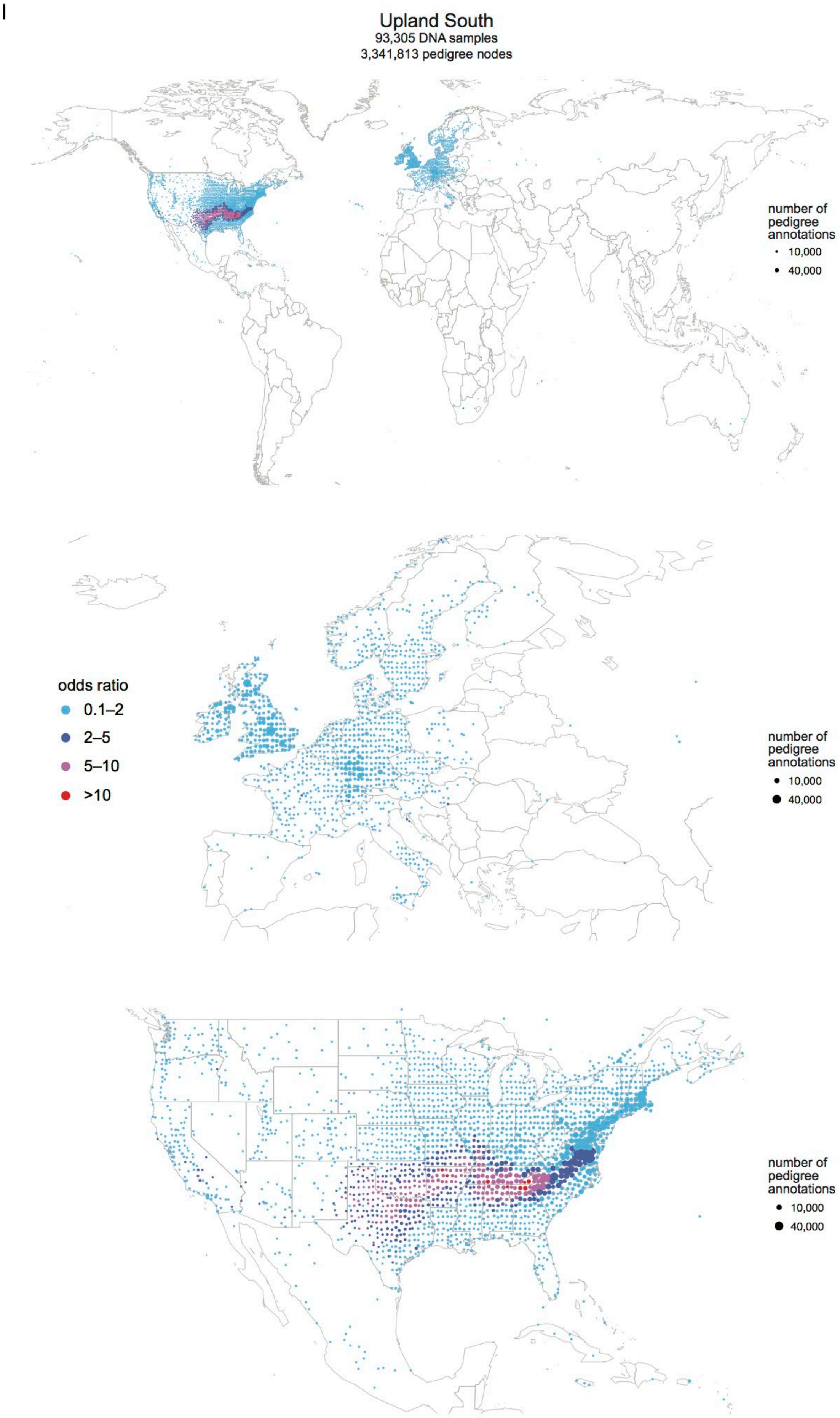
I
Upland South
93,305 DNA samples
3,341,813 pedigree nodes
number of pedigree annotations
10,000
40,000
odds ratio
0.1–2
2–5
5–10
>10
number of pedigree annotations
10,000
40,000
number of pedigree annotations
10,000
40,000

J

Lower South

77,581 DNA samples

2,608,314 pedigree nodes

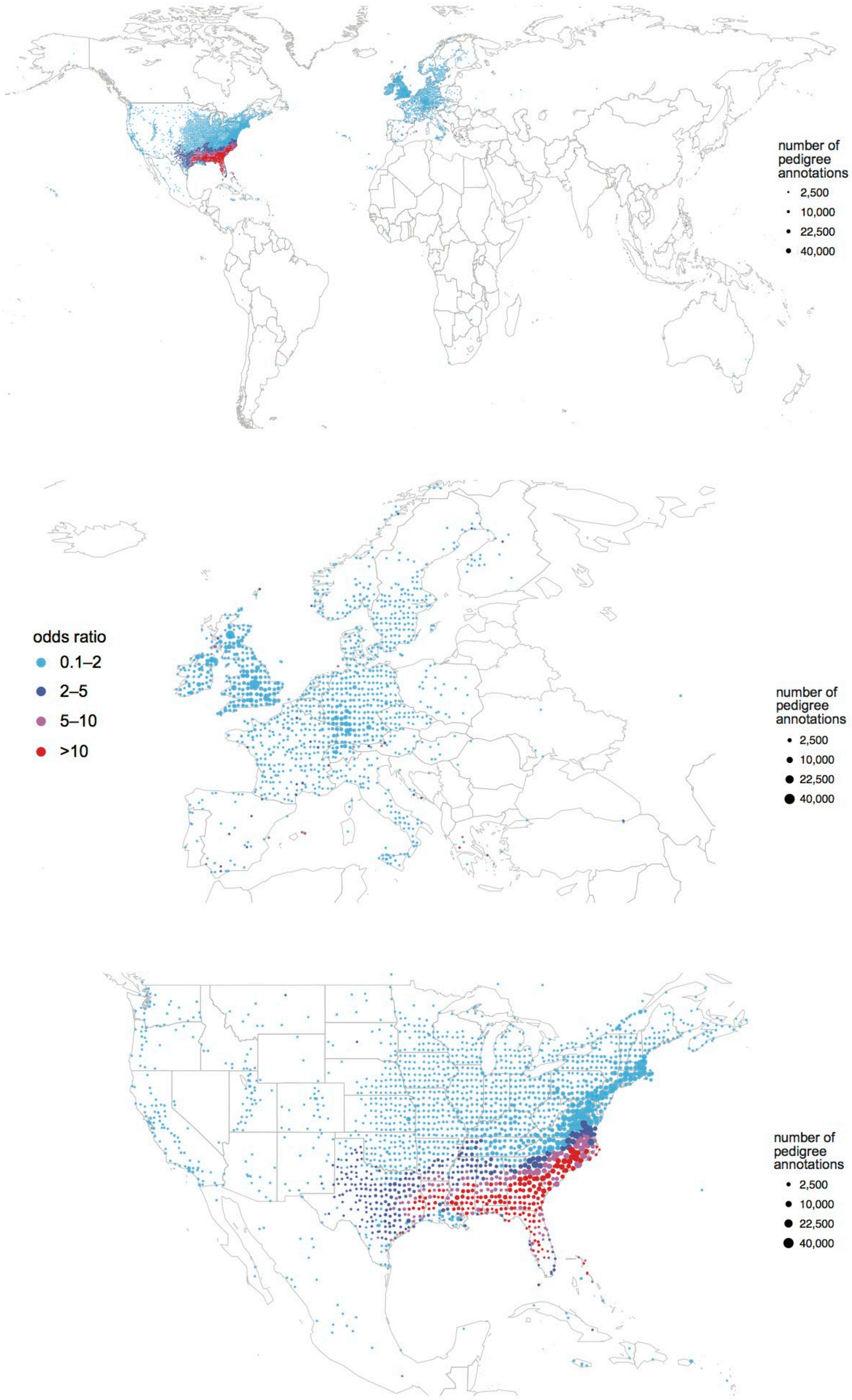

K

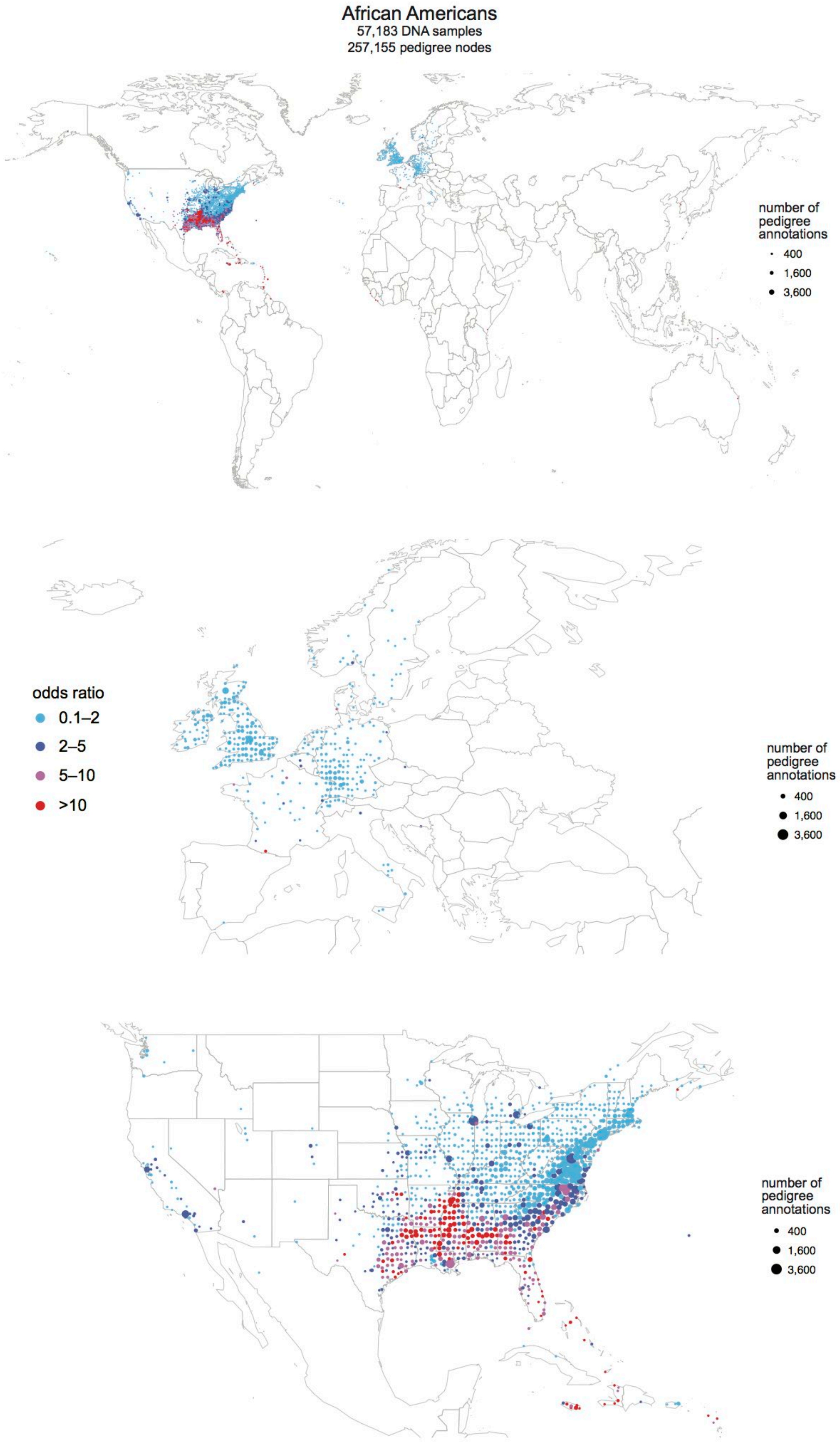

African Americans
57,183 DNA samples
257,155 pedigree nodes
number of pedigree annotations
400
1,600
3,600
odds ratio
0.1–2
2–5
5–10
>10
number of pedigree annotations
400
1,600
3,600
number of pedigree annotations
400
1,600
3,600

L

West, Northwest and Central Mexico
2,139 DNA samples
52,216 pedigree nodes

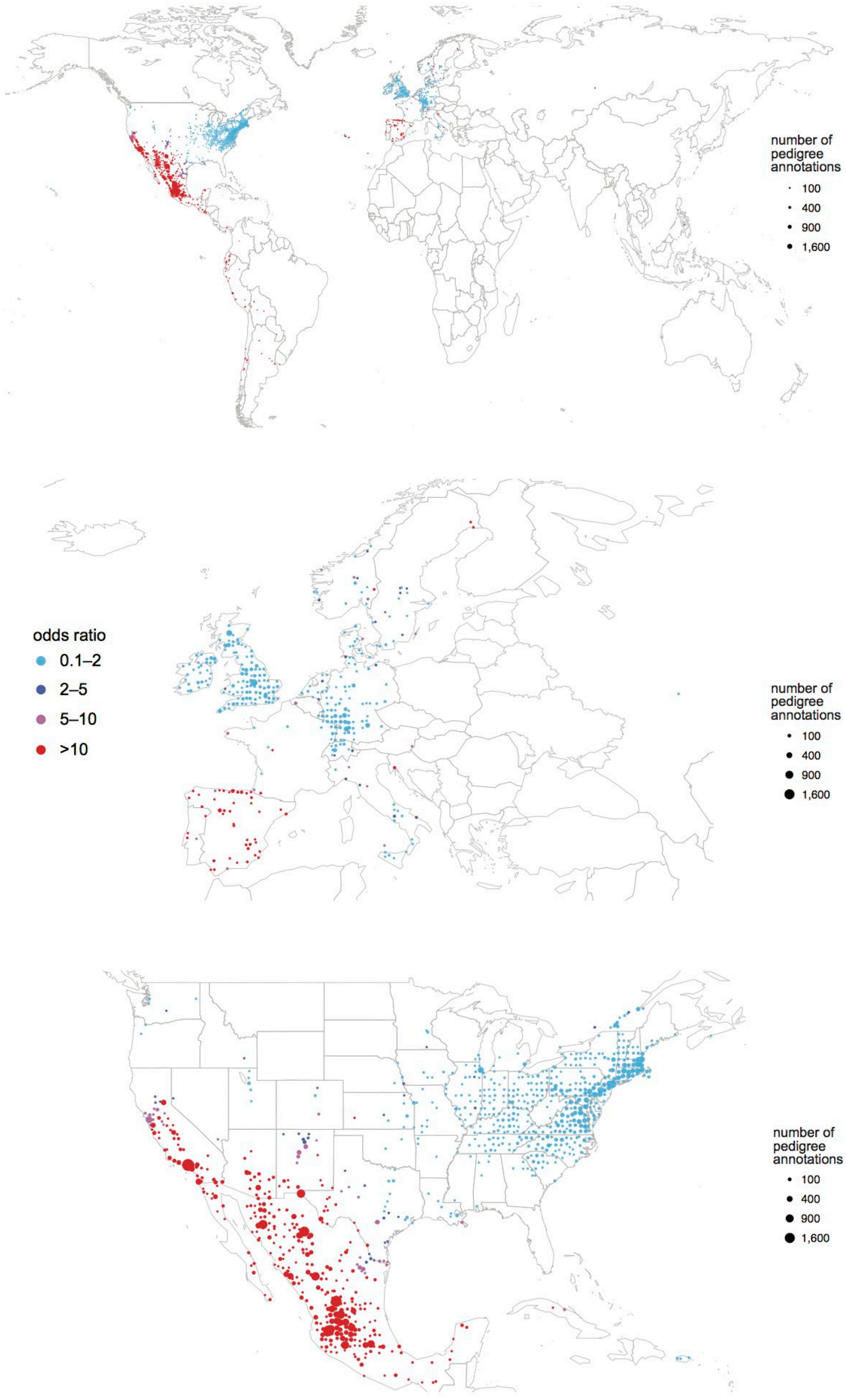

M

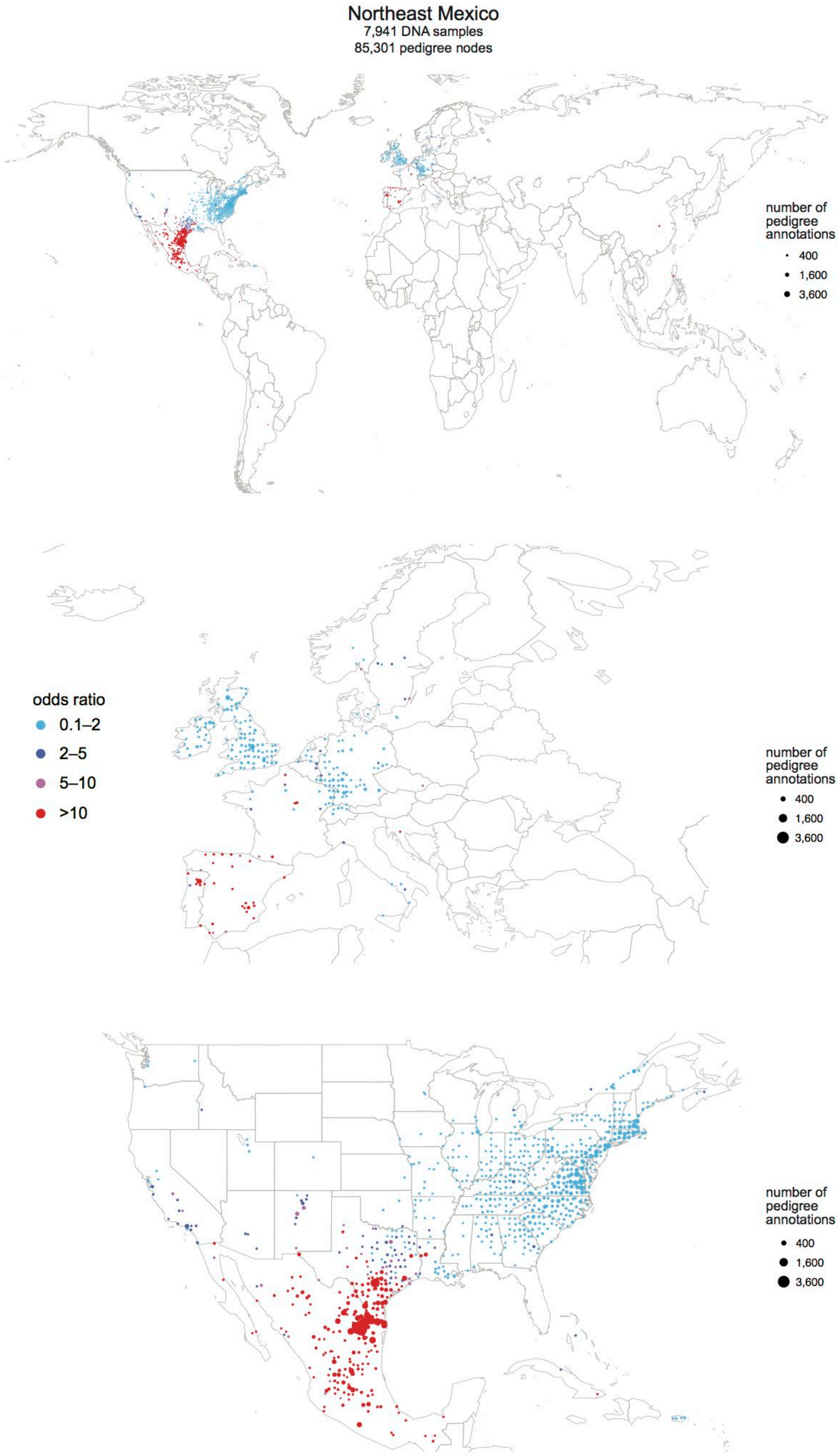
Northeast Mexico
7,941 DNA samples
85,301 pedigree nodes
number of pedigree annotations
400
1,600
3,600
odds ratio
0.1–2
2–5
5–10
>10
number of pedigree annotations
400
1,600
3,600
number of pedigree annotations
400
1,600
3,600

N

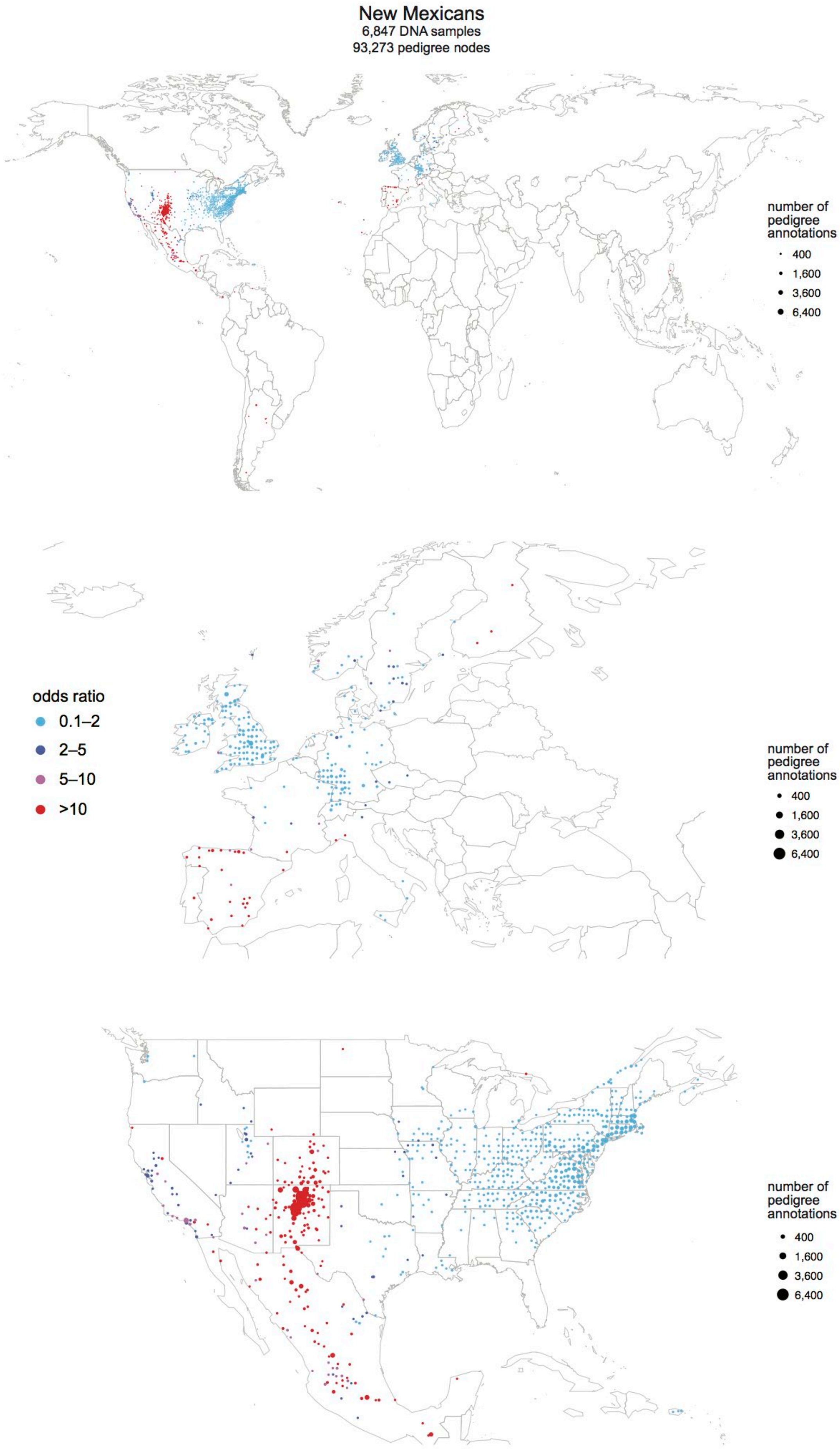
New Mexicans
6,847 DNA samples
93,273 pedigree nodes
number of pedigree annotations
400
1,600
3,600
6,400
odds ratio
0.1–2
2–5
5–10
>10
number of pedigree annotations
400
1,600
3,600
6,400
number of pedigree annotations
400
1,600
3,600
6,400

O

Central Americans and Colombians

2,675 DNA samples

11,836 pedigree nodes

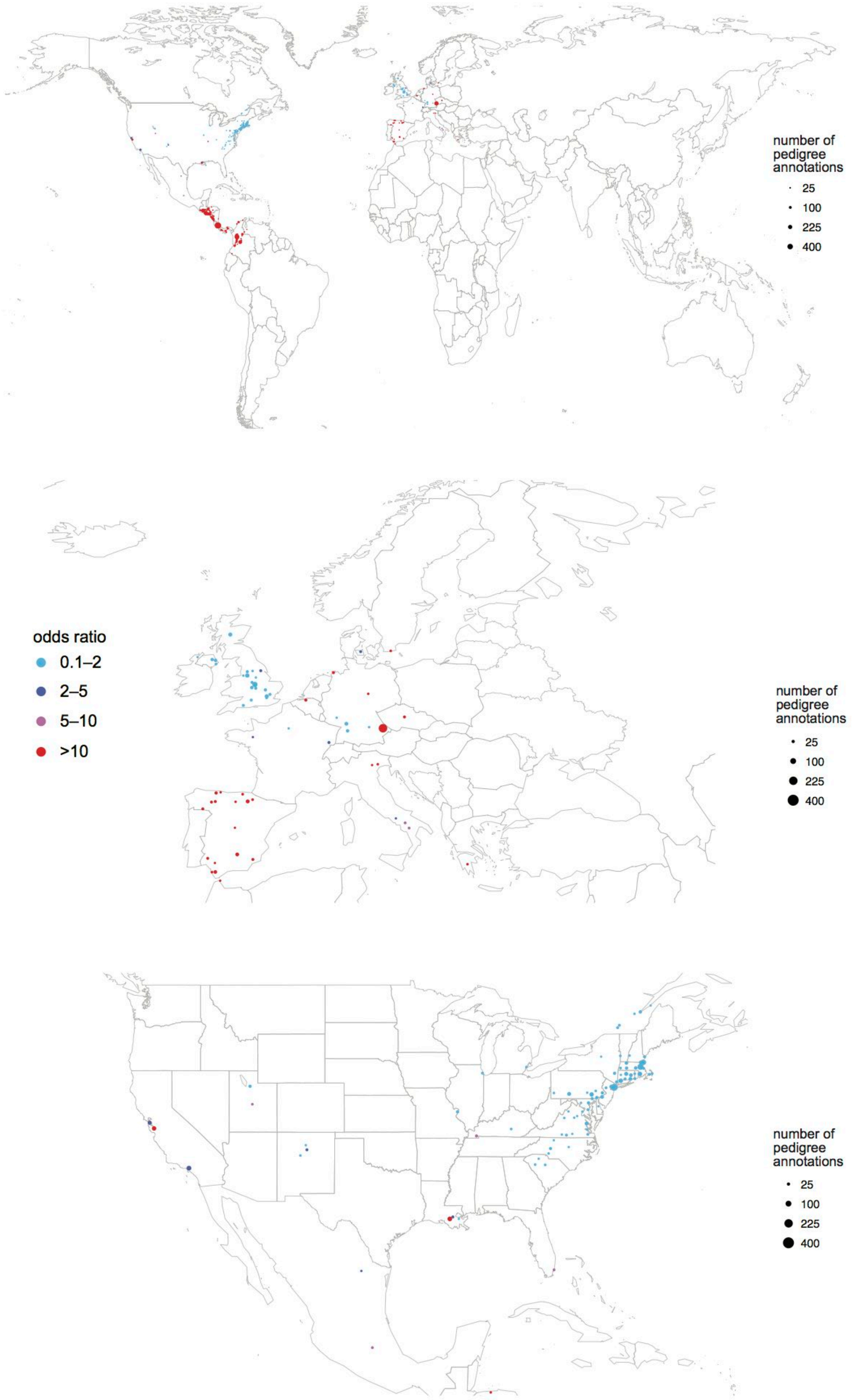

P

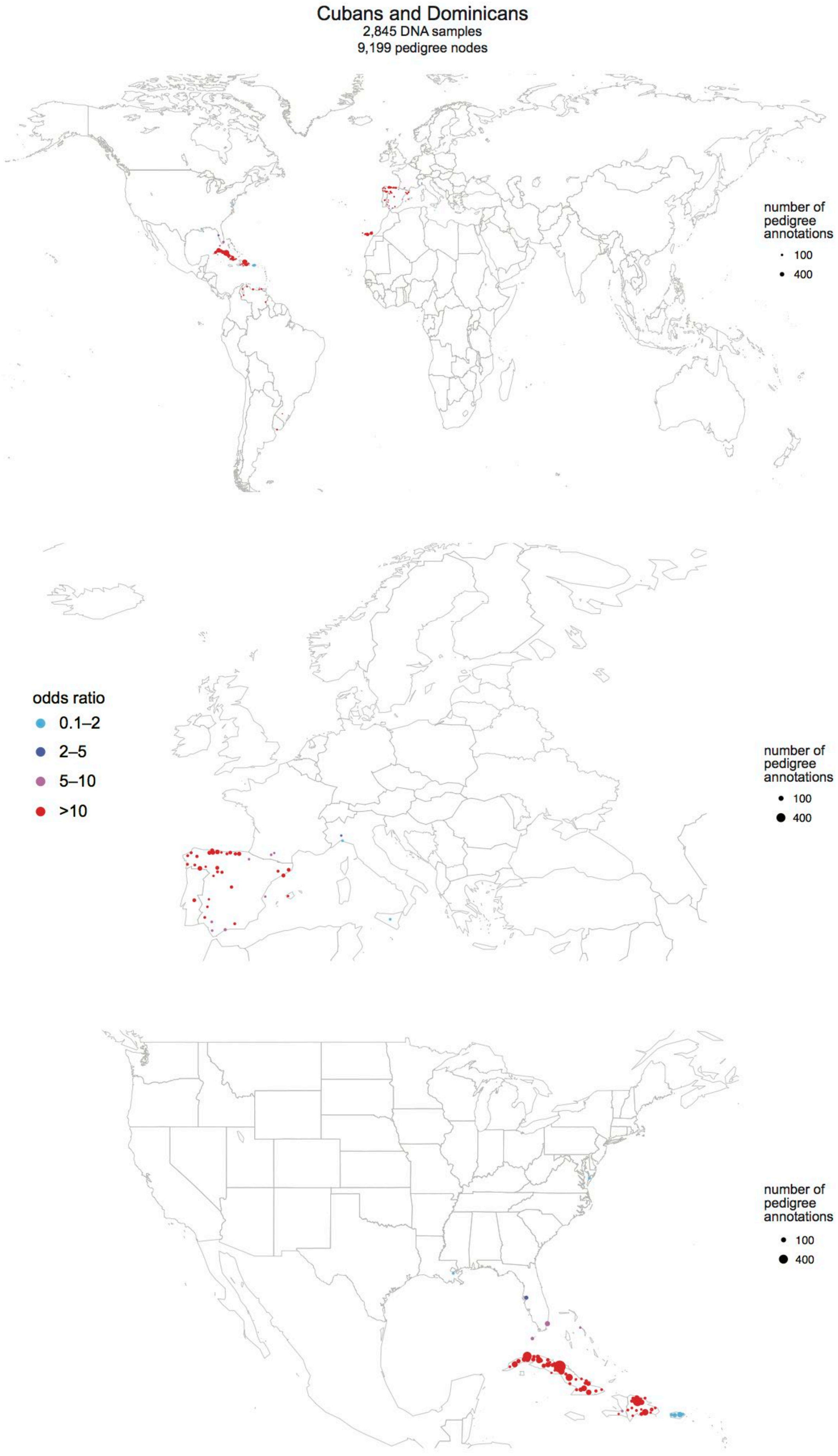
Cubans and Dominicans
2,845 DNA samples
9,199 pedigree nodes
number of pedigree annotations
100
400
odds ratio
0.1–2
2–5
5–10
>10
number of pedigree annotations
100
400
number of pedigree annotations
100
400

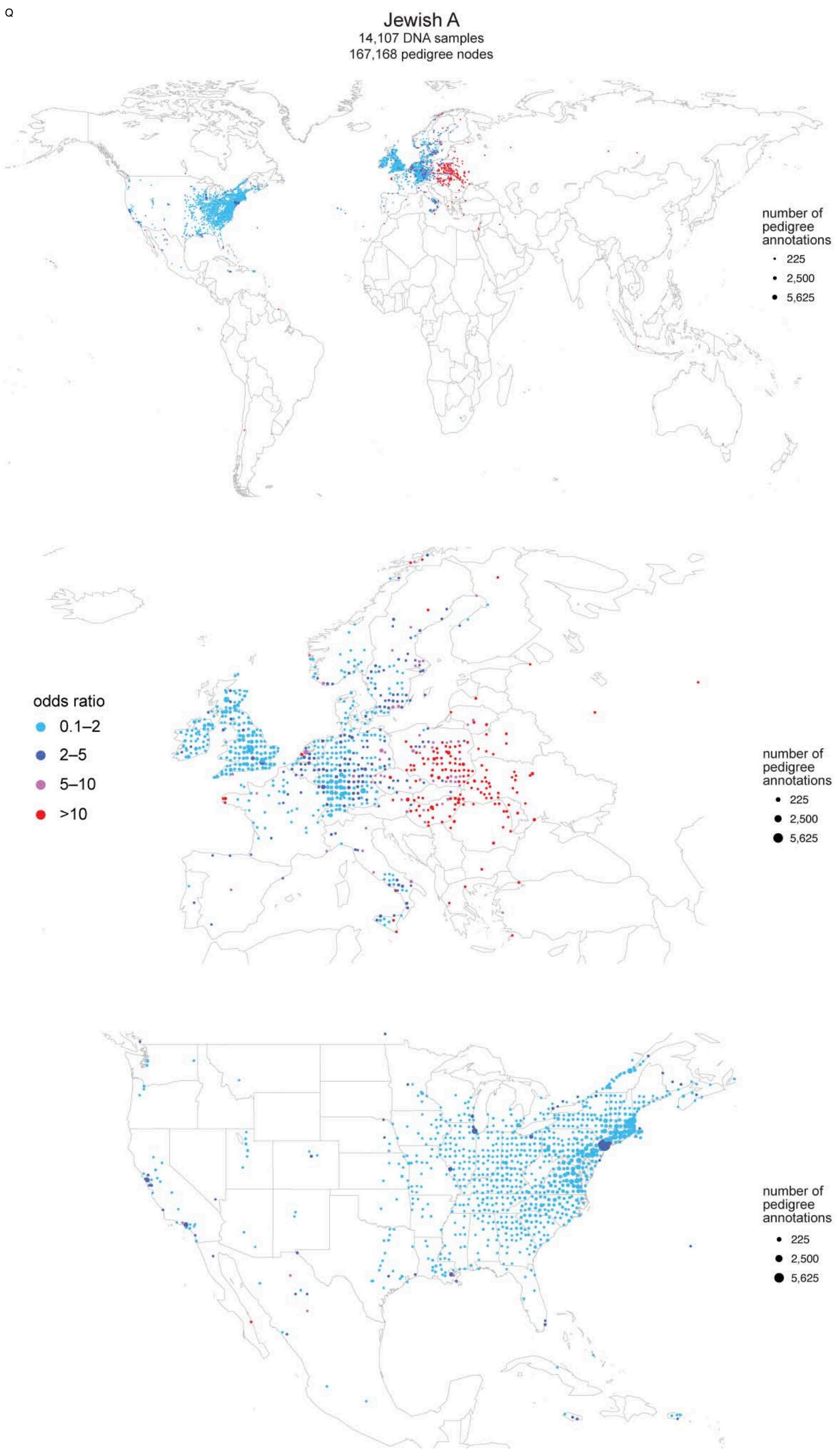
Q
Jewish A
14,107 DNA samples
167,168 pedigree nodes
number of pedigree annotations
225
2,500
5,625
odds ratio
0.1–2
2–5
5–10
>10
number of pedigree annotations
225
2,500
5,625
number of pedigree annotations
225
2,500
5,625

R

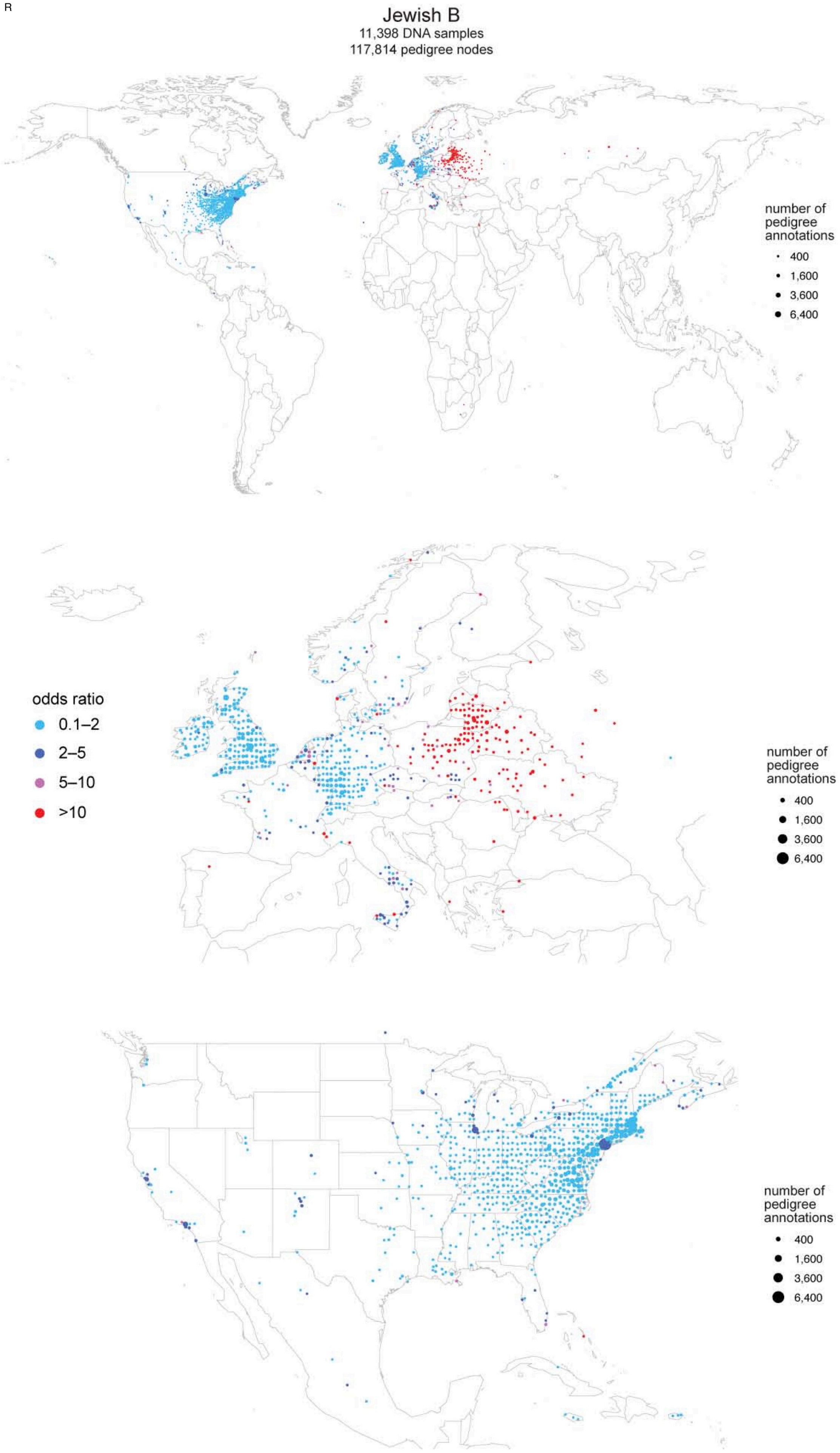
Jewish B
11,398 DNA samples
117,814 pedigree nodes
number of pedigree annotations
400
1,600
3,600
6,400
odds ratio
0.1–2
2–5
5–10
>10
number of pedigree annotations
400
1,600
3,600
6,400
number of pedigree annotations
400
1,600
3,600
6,400

S

Jewish C

2,346 DNA samples
20,721 pedigree nodes

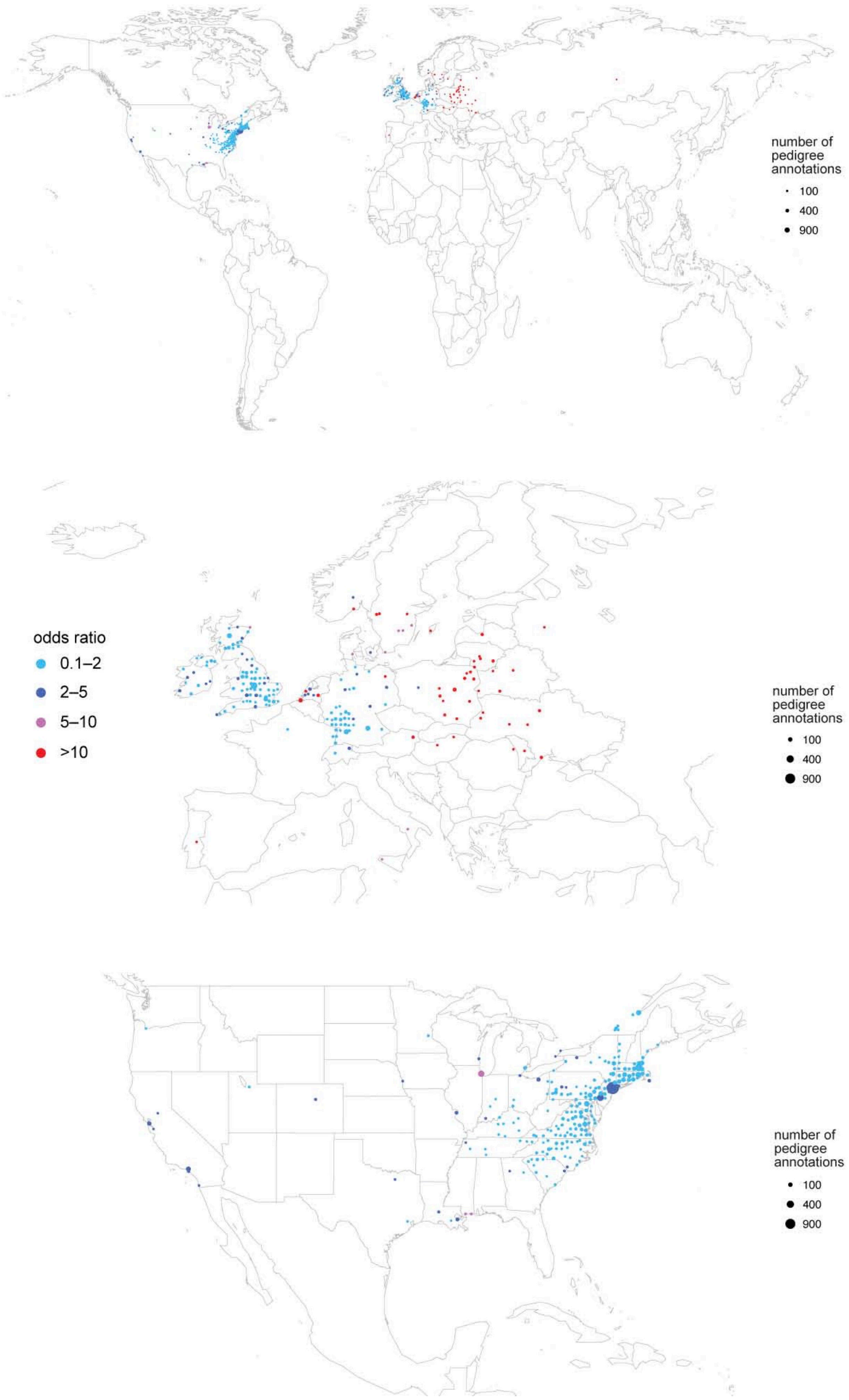

T

Puerto Rico, East

3,477 DNA samples
28,561 pedigree nodes

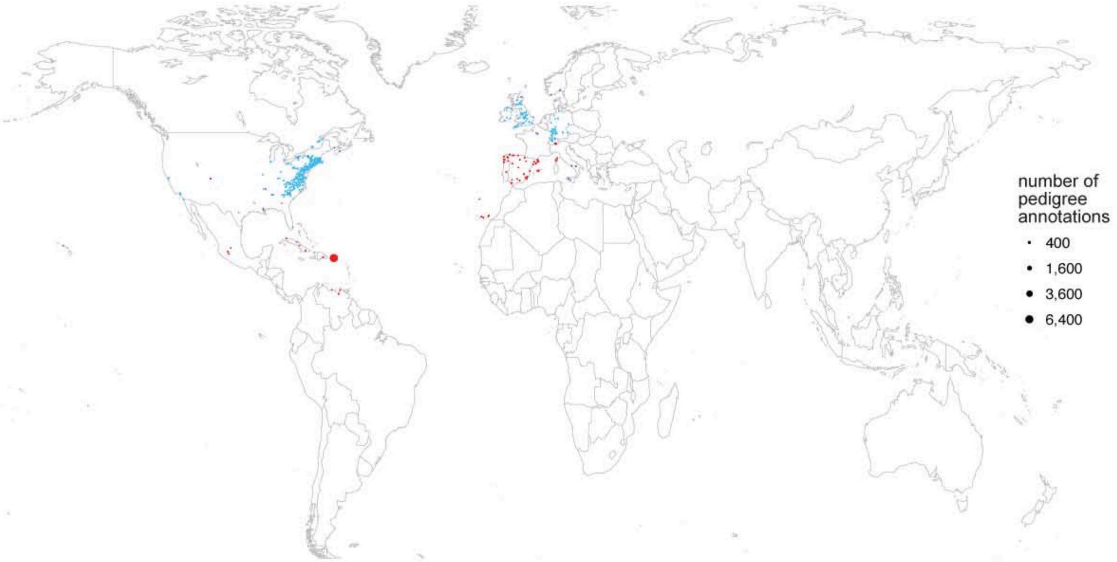

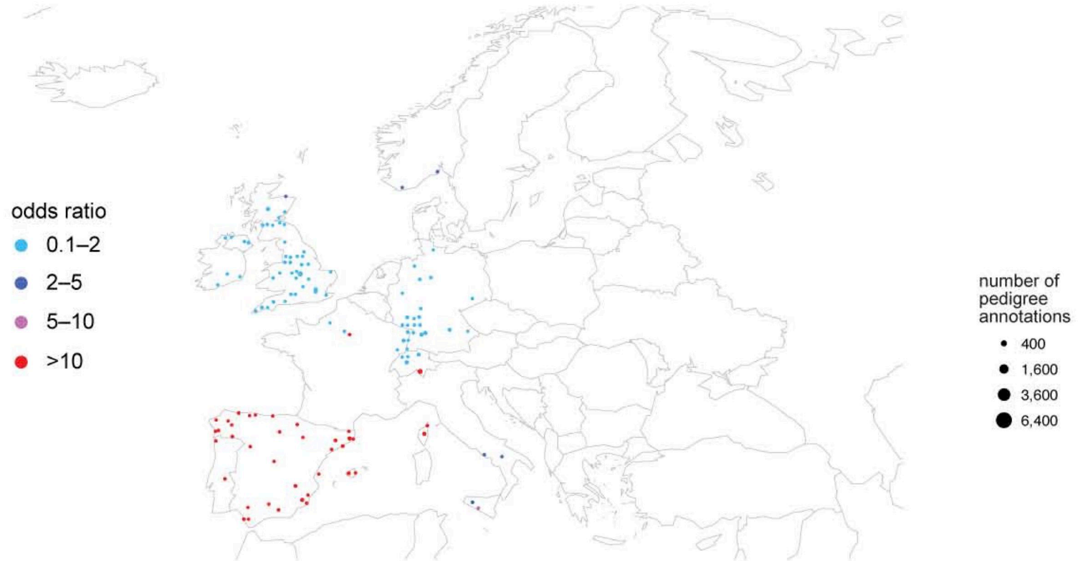

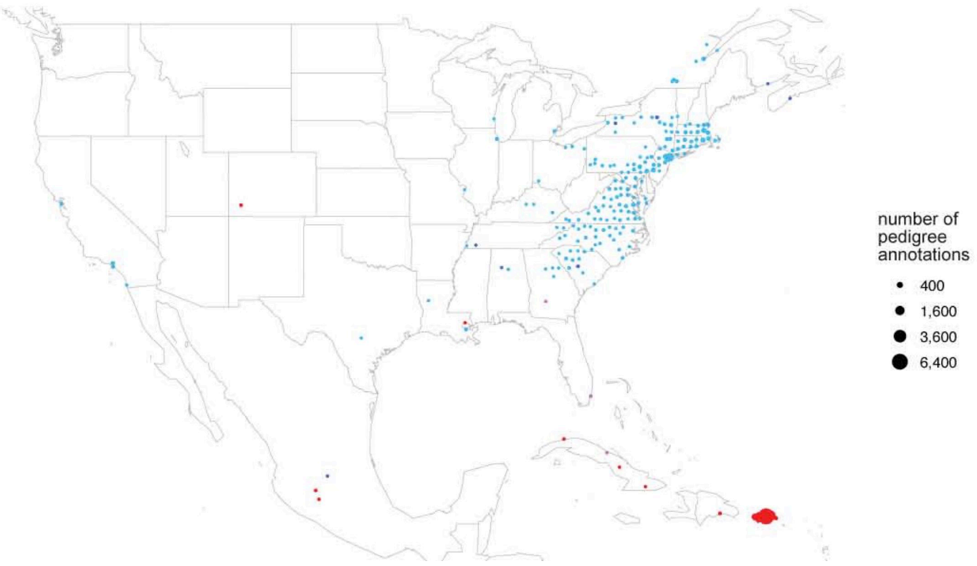

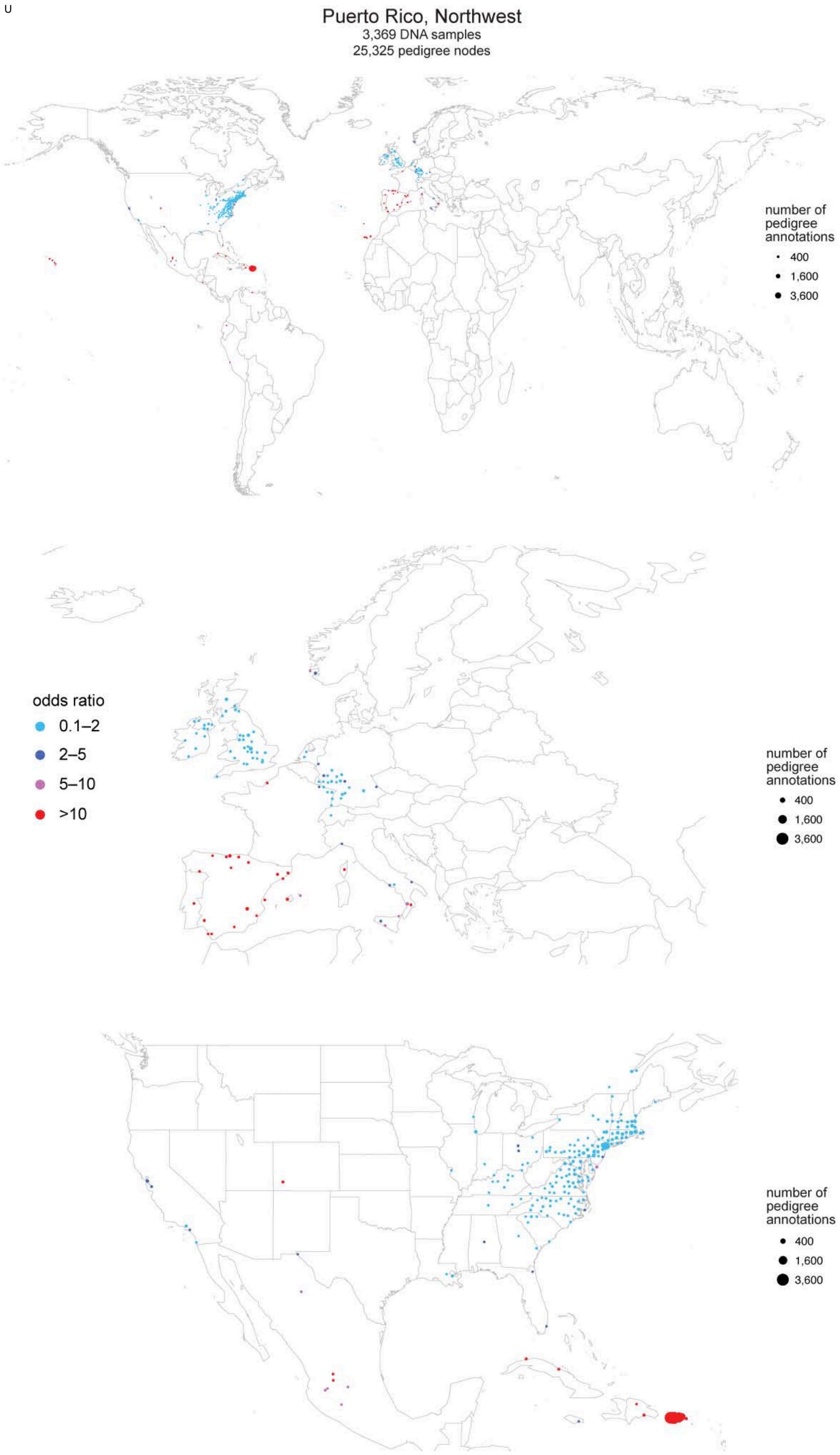
U
Puerto Rico, Northwest
3,369 DNA samples
25,325 pedigree nodes
number of
pedigree
annotations
400
1,600
3,600
odds ratio
0.1–2
2–5
5–10
>10
number of
pedigree
annotations
400
1,600
3,600
number of
pedigree
annotations
400
1,600
3,600

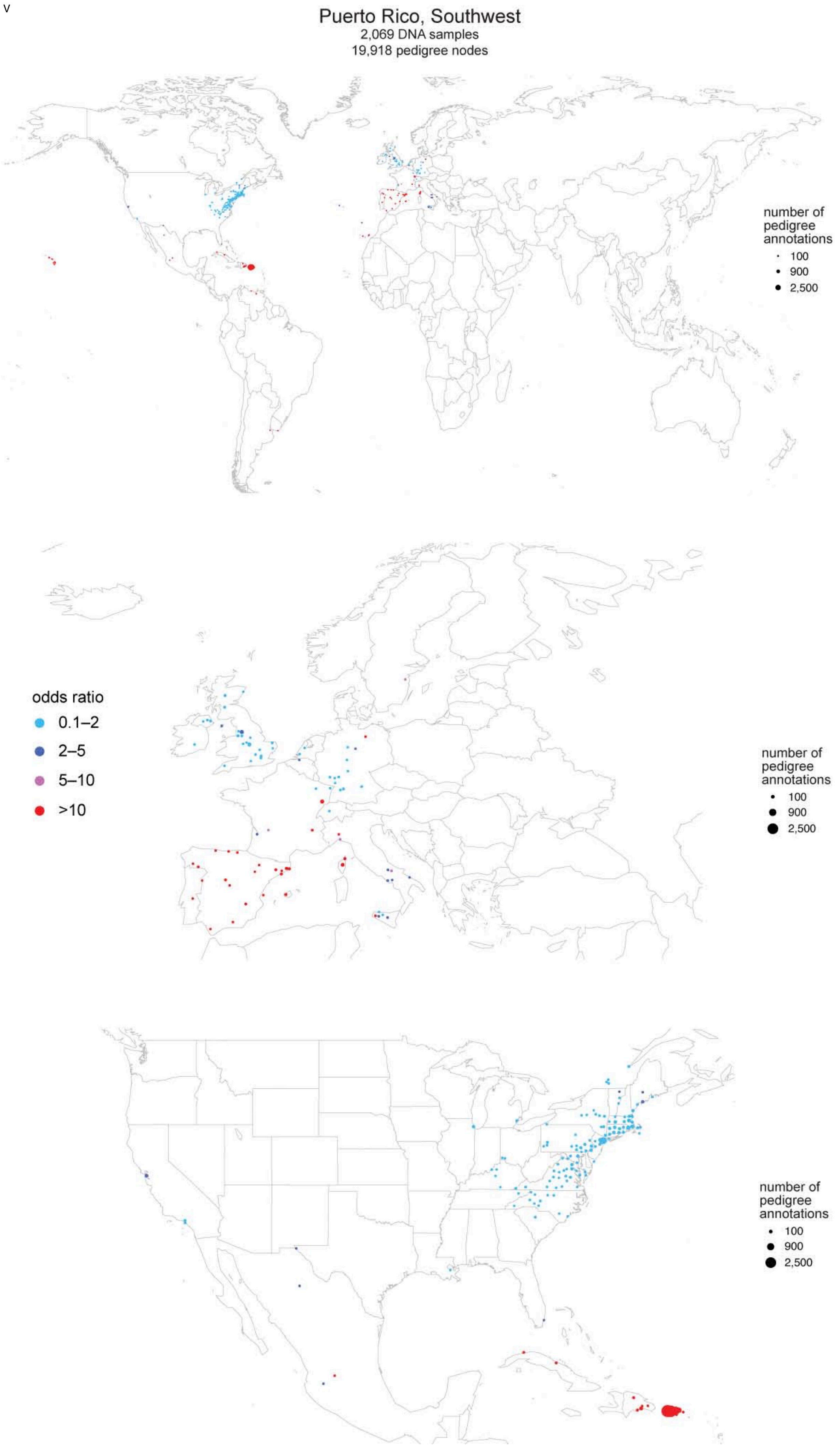

V
Puerto Rico, Southwest
2,069 DNA samples
19,918 pedigree nodes
number of pedigree annotations
100
900
2,500
odds ratio
0.1–2
2–5
5–10
>10
number of pedigree annotations
100
900
2,500
number of pedigree annotations
100
900
2,500

Supplementary Figure 19 | Distribution of ancestral birth locations worldwide, in North America, and in Europe, for each cluster detected in an IBD sub-network. See description of Supplementary Fig. 18 for details about this figure. See Supplementary Fig. 21 for a higher resolution view of the ancestral birth locations in the three Puerto Rico clusters. All maps in our figures were generated with the *maps* R package using data from the Natural Earth Project (1:50m world map, version 2.0). These data are made available in the public domain (Creative Commons CC0).

A

Utah

4,519 DNA samples

283,911 pedigree nodes

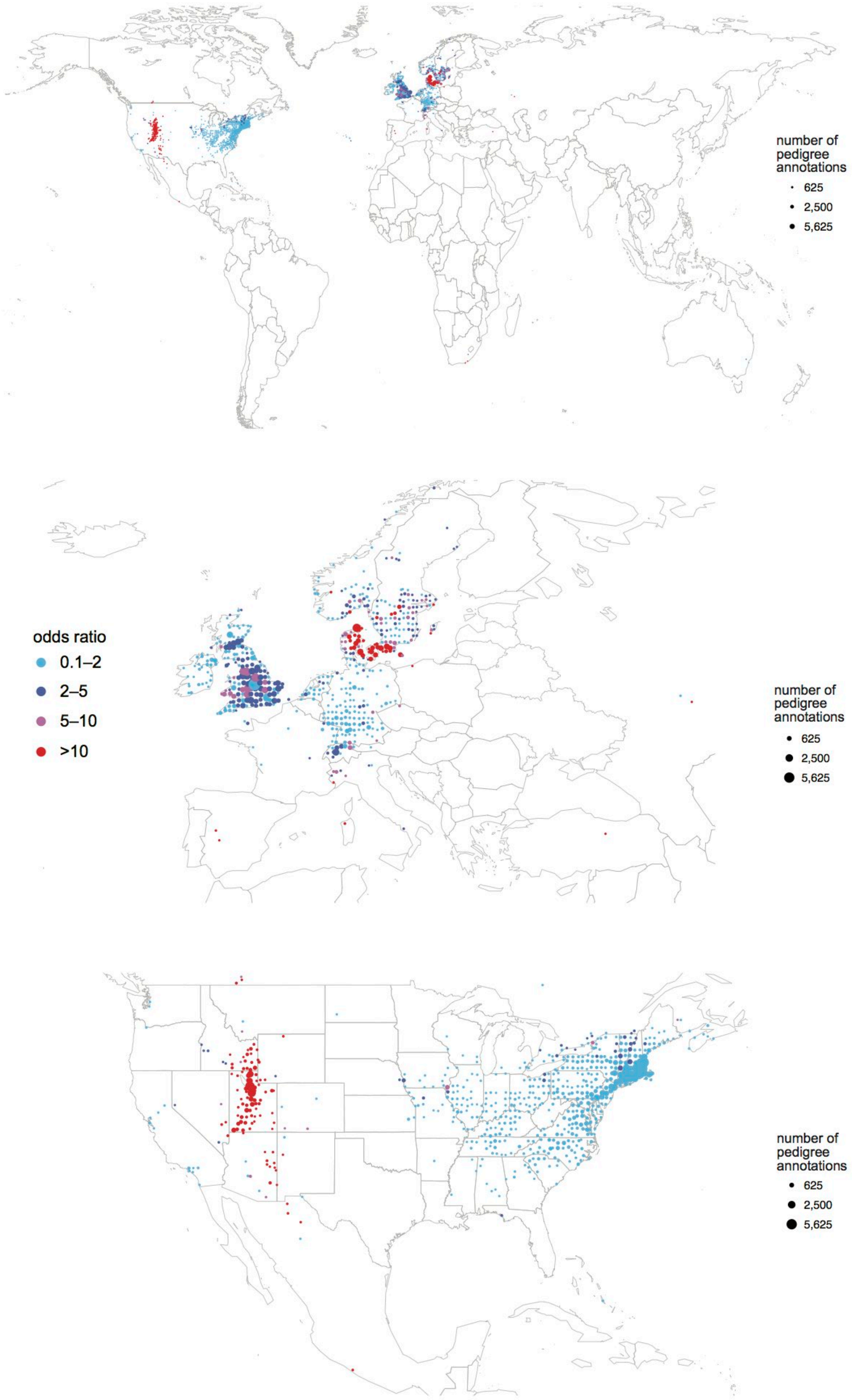

B

Amish
1,067 DNA samples
42,903 pedigree nodes

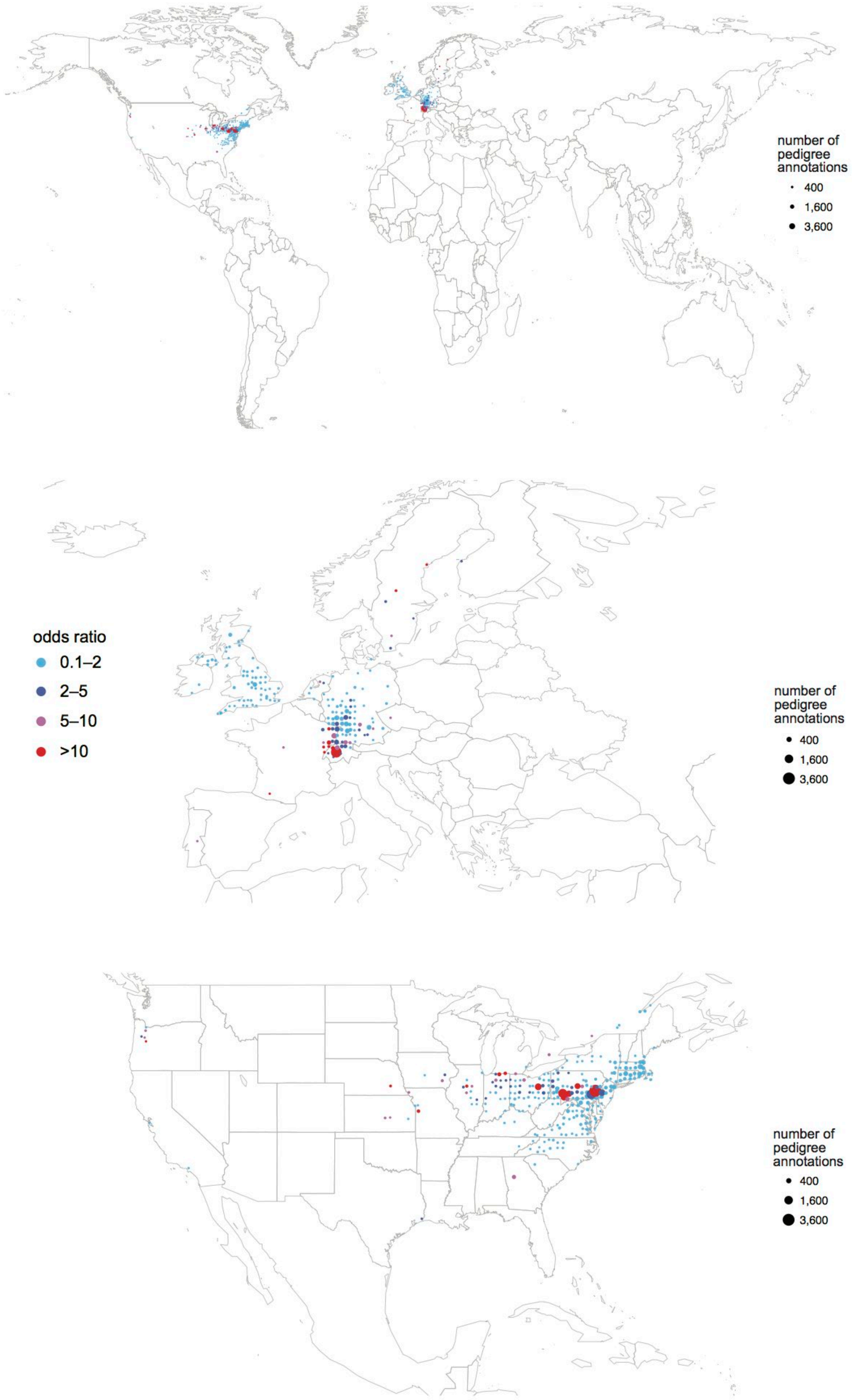

C

Irish

20,747 DNA samples
222,198 pedigree nodes

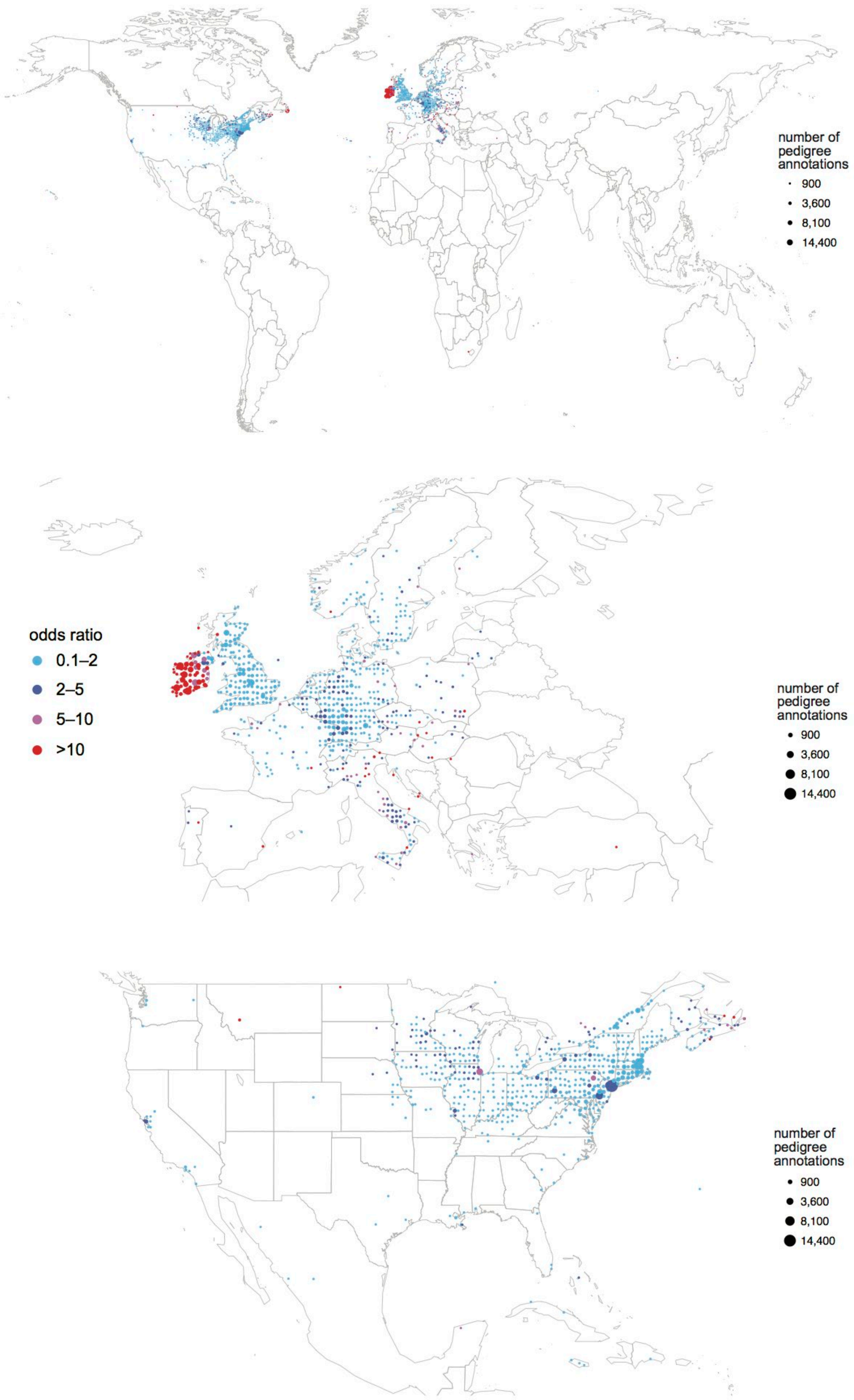

D

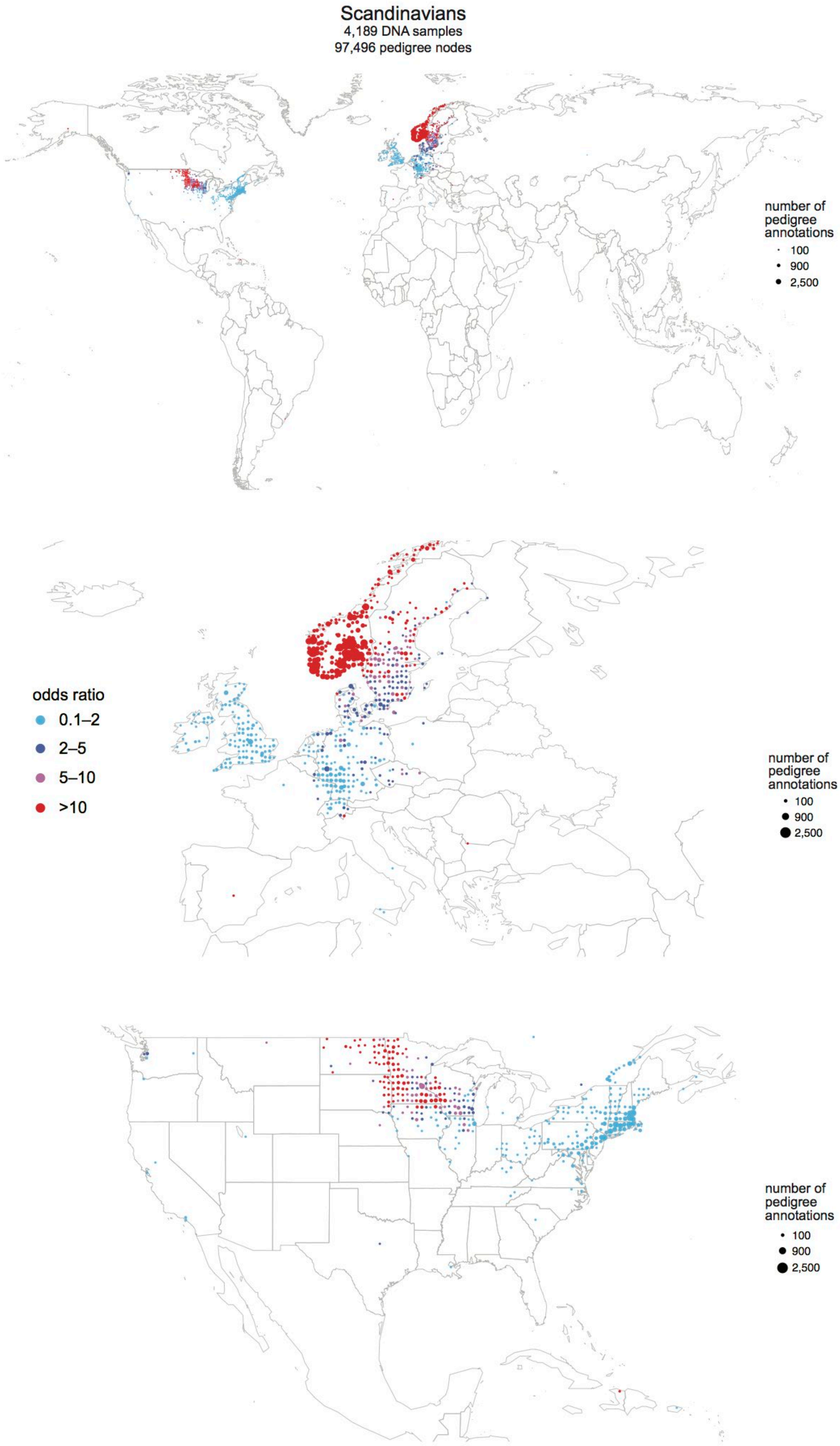
Scandinavians
4,189 DNA samples
97,496 pedigree nodes
number of pedigree annotations
100
900
2,500
odds ratio
0.1–2
2–5
5–10
>10
number of pedigree annotations
100
900
2,500
number of pedigree annotations
100
900
2,500

E

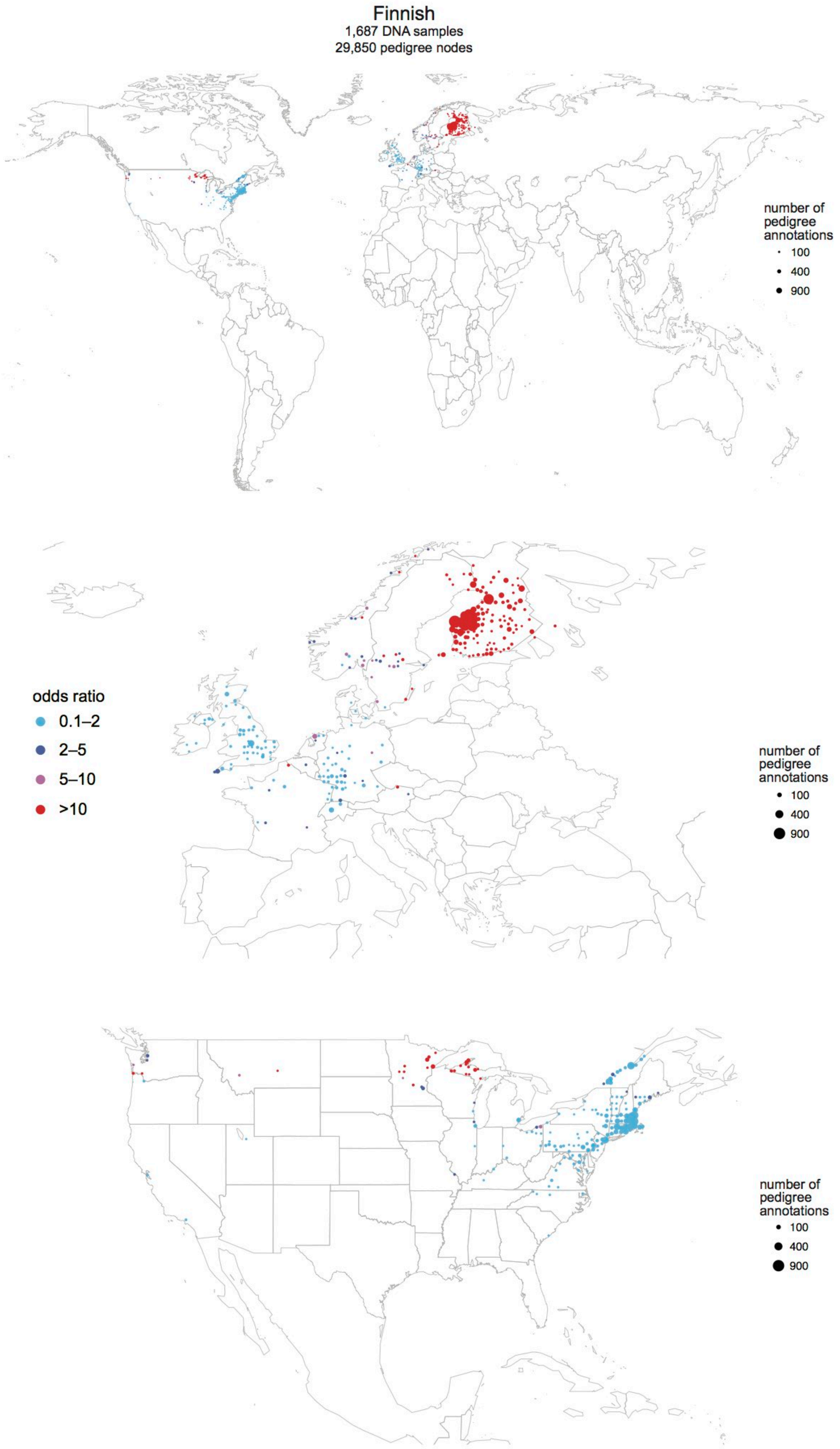
Finnish
1,687 DNA samples
29,850 pedigree nodes
number of
pedigree
annotations
100
400
900
odds ratio
0.1–2
2–5
5–10
>10
number of
pedigree
annotations
100
400
900
number of
pedigree
annotations
100
400
900

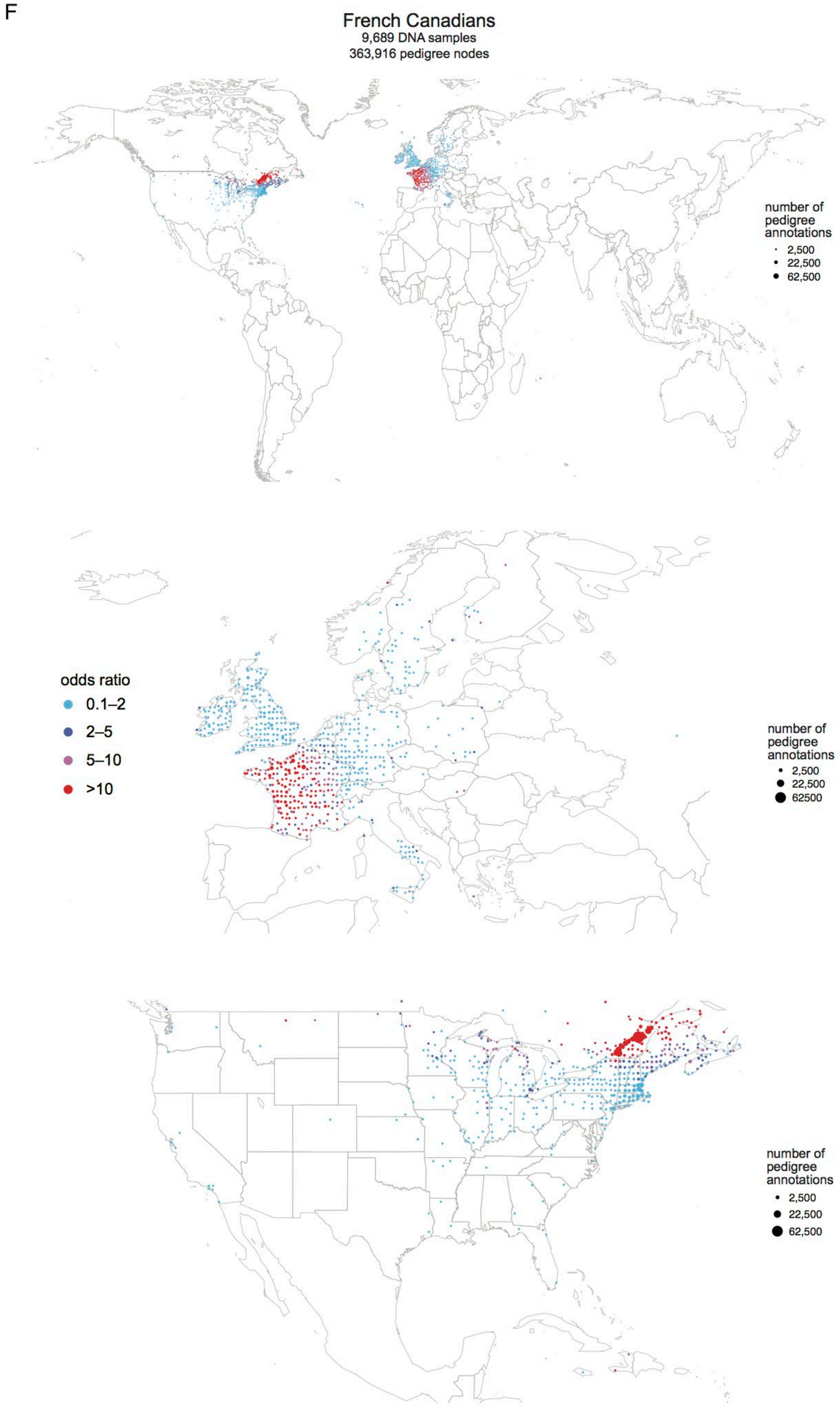
F
French Canadians
9,689 DNA samples
363,916 pedigree nodes
number of pedigree annotations
2,500
22,500
62,500
odds ratio
0.1–2
2–5
5–10
>10
number of pedigree annotations
2,500
22,500
62500
number of pedigree annotations
2,500
22,500
62,500

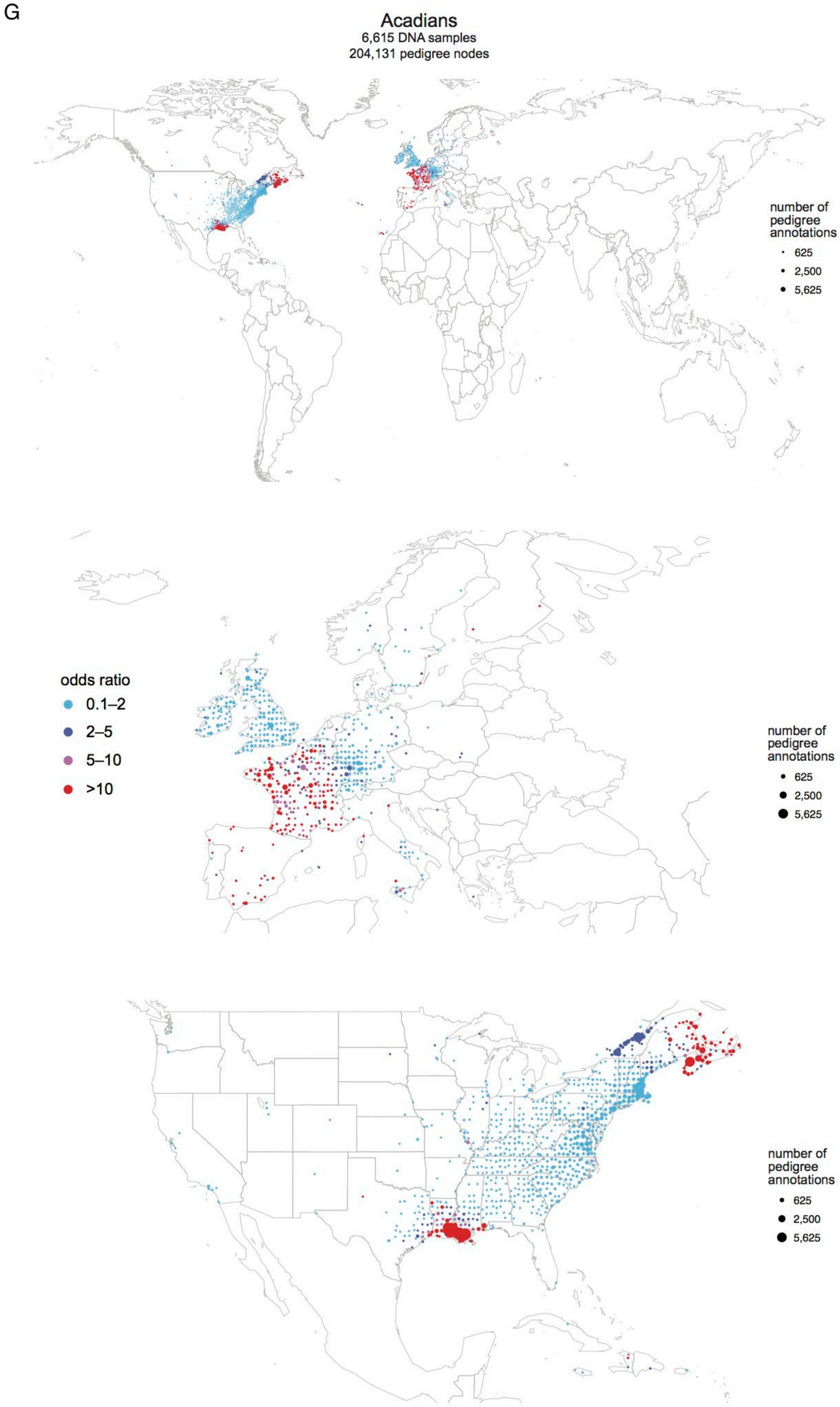
G
Acadians
6,615 DNA samples
204,131 pedigree nodes
number of pedigree annotations
625
2,500
5,625
odds ratio
0.1–2
2–5
5–10
>10
number of pedigree annotations
625
2,500
5,625
number of pedigree annotations
625
2,500
5,625

H

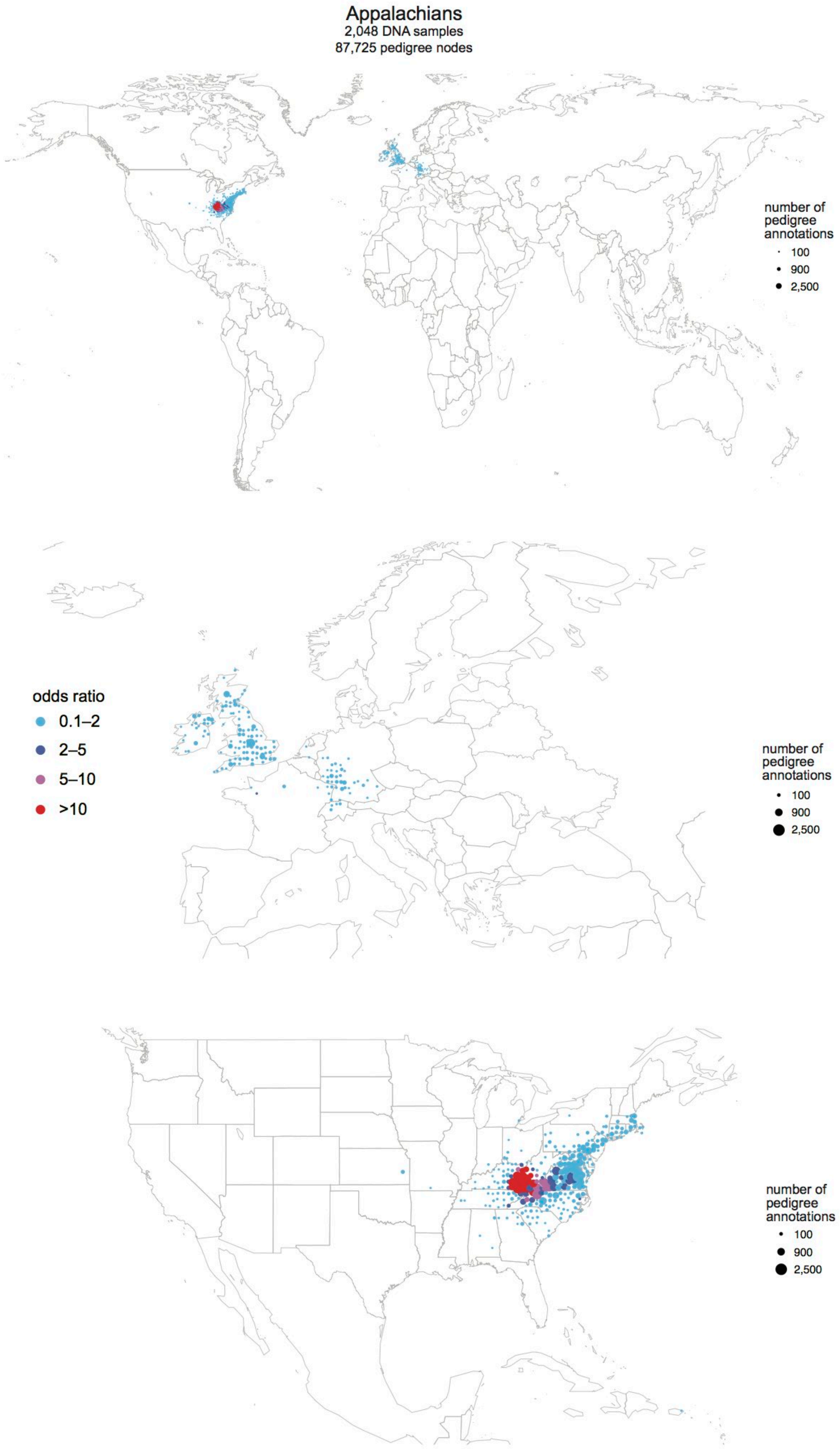
Appalachians
2,048 DNA samples
87,725 pedigree nodes
number of pedigree annotations
100
900
2,500
odds ratio
0.1–2
2–5
5–10
>10
number of pedigree annotations
100
900
2,500
number of pedigree annotations
100
900
2,500

l

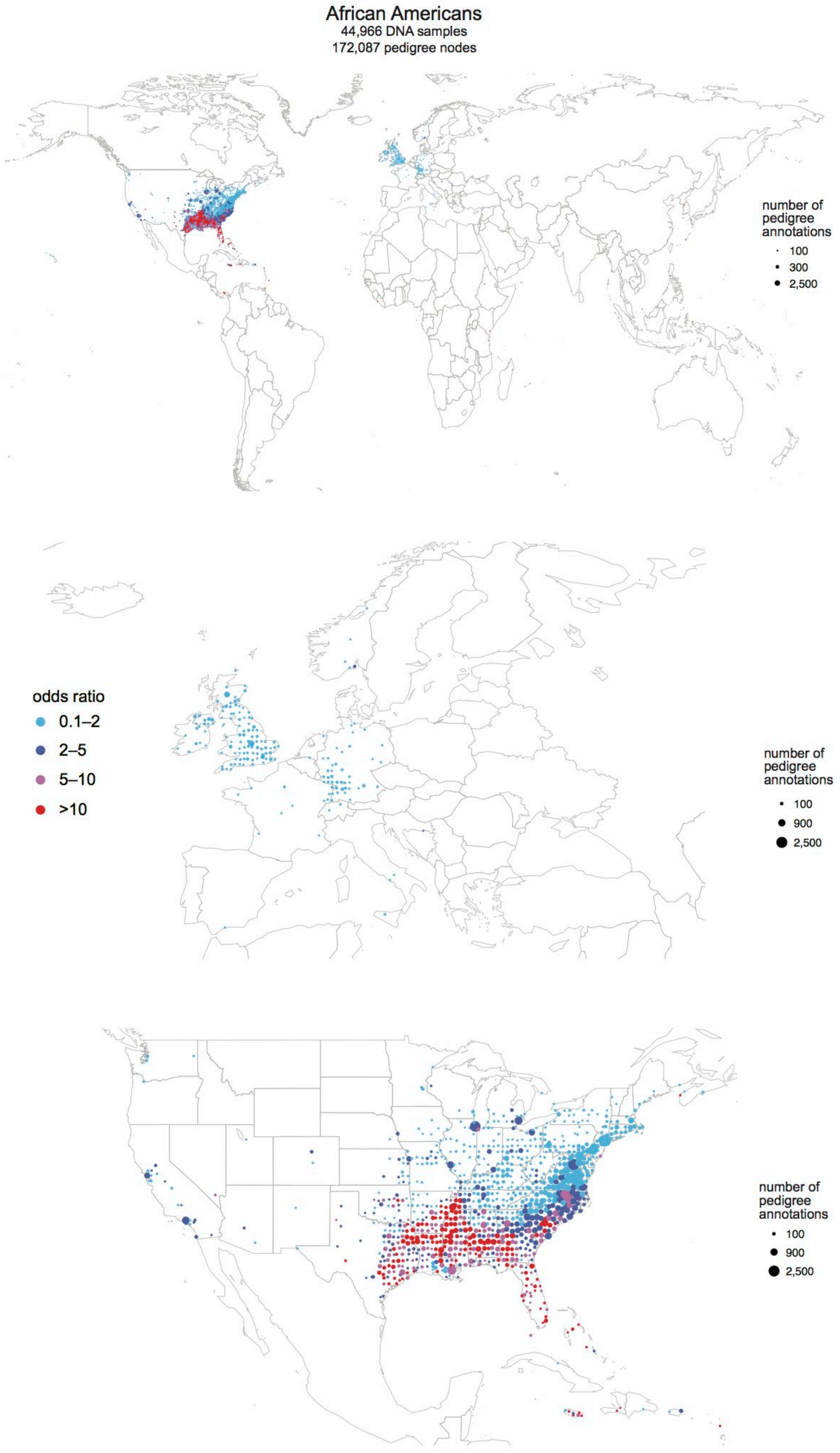
African Americans
44,966 DNA samples
172,087 pedigree nodes
number of pedigree annotations
100
300
2,500
odds ratio
0.1–2
2–5
5–10
>10
number of pedigree annotations
100
900
2,500
number of pedigree annotations
100
900
2,500

J

West Mexico
1,356 DNA samples
5,924 pedigree nodes

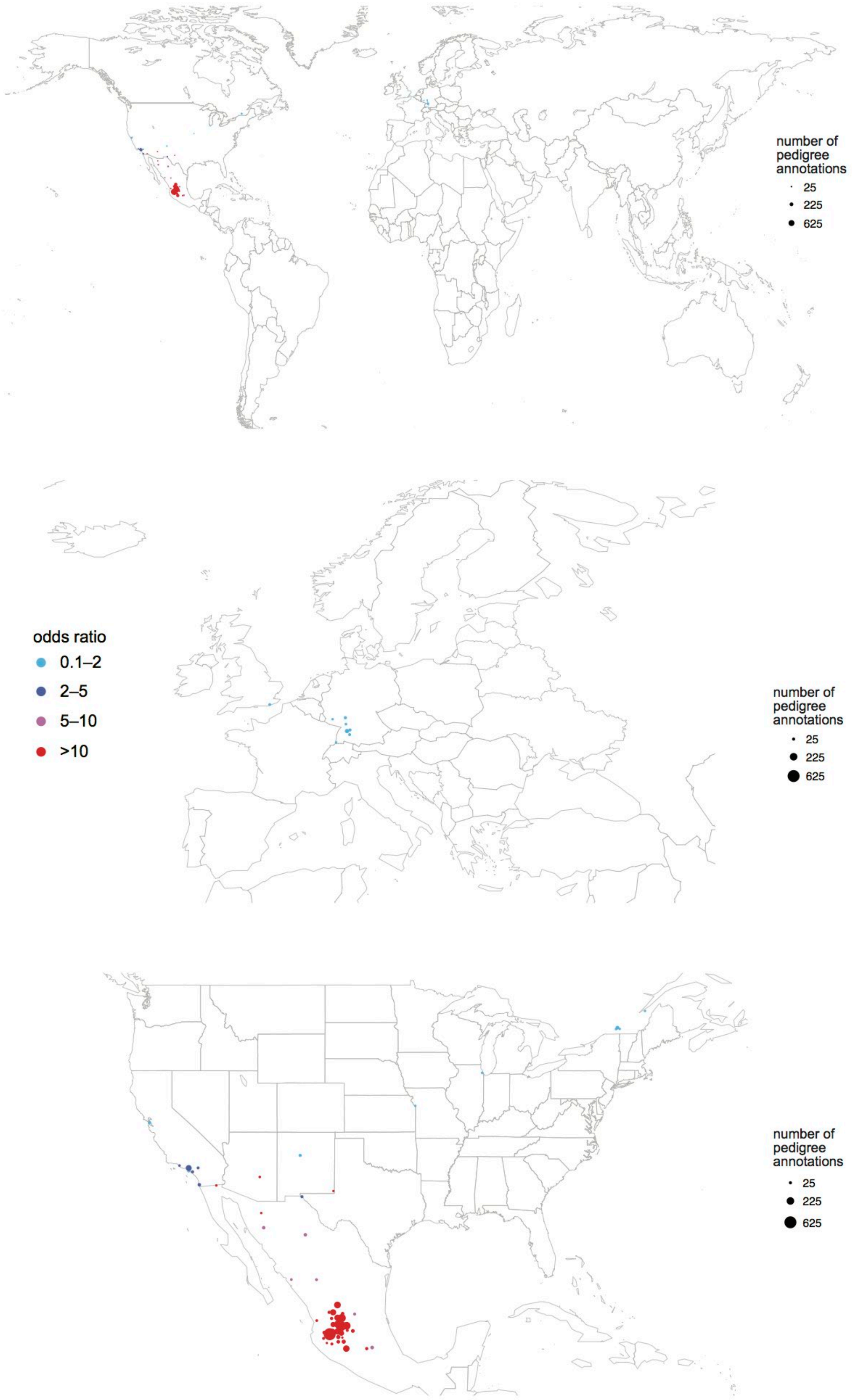

K

Northeast Mexico
6,311 DNA samples
61,391 pedigree nodes

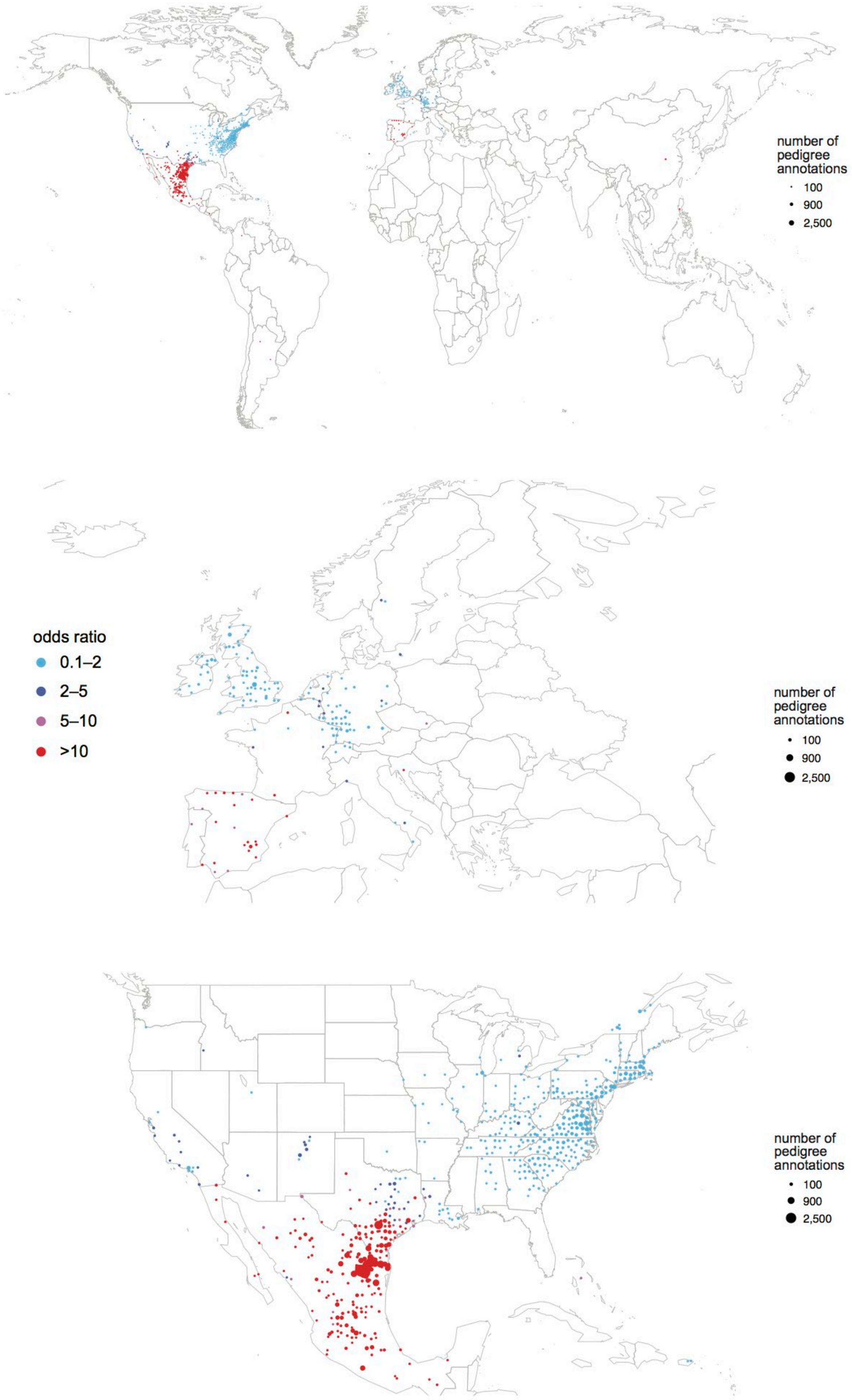

L

New Mexicans

5,291 DNA samples

65,236 pedigree nodes

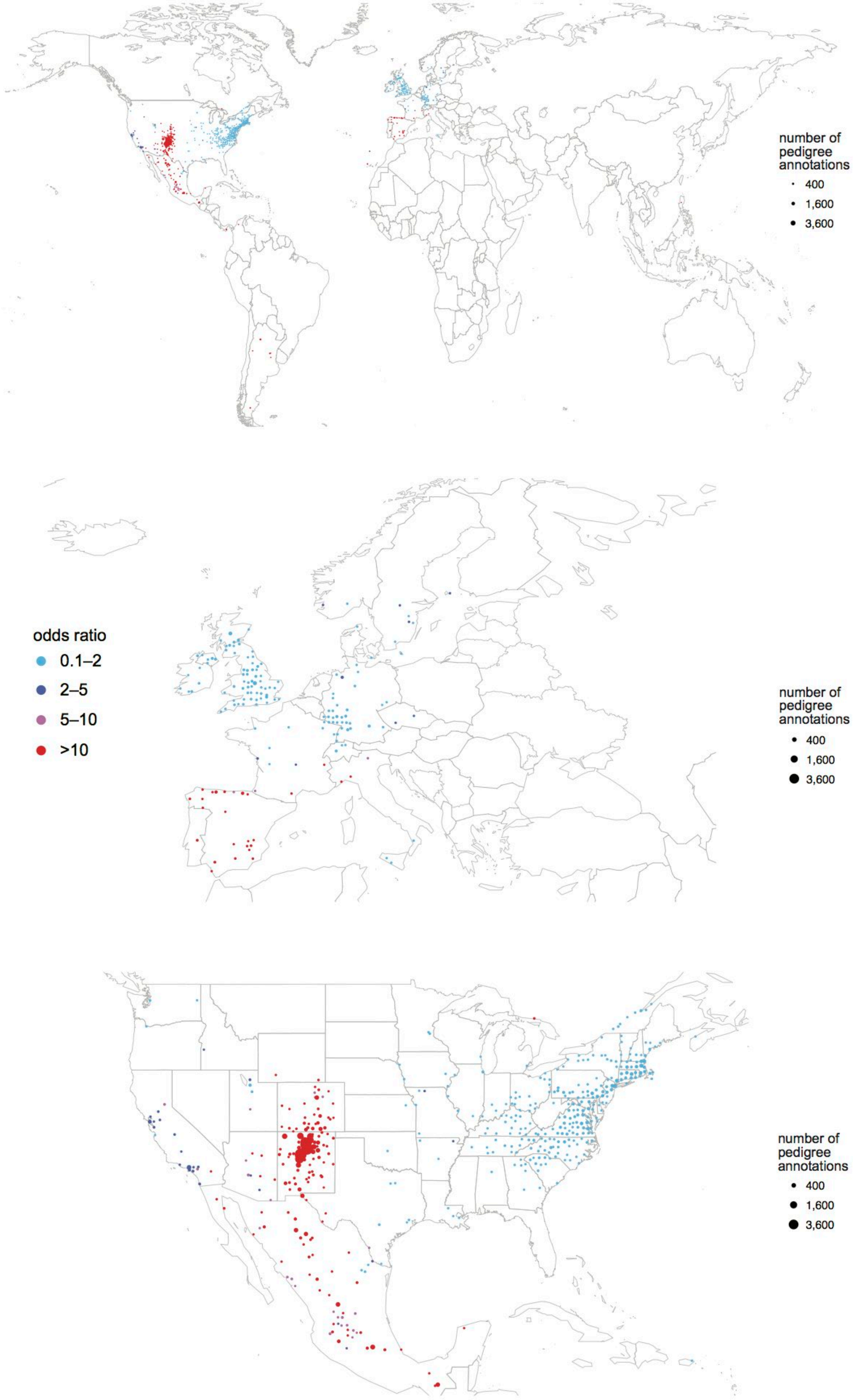

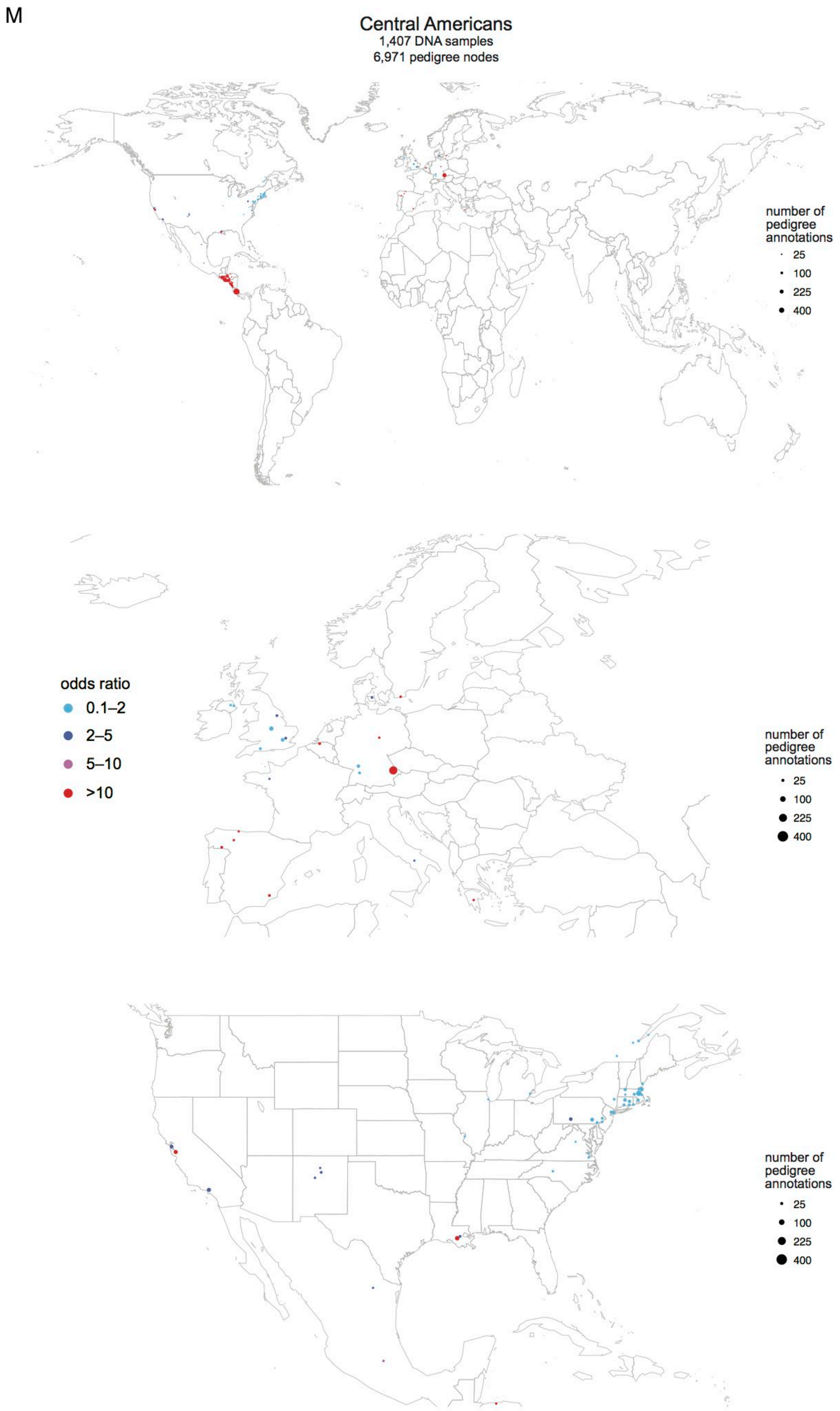
M
Central Americans
1,407 DNA samples
6,971 pedigree nodes
number of pedigree annotations
25
100
225
400
odds ratio
0.1–2
2–5
5–10
>10
number of pedigree annotations
25
100
225
400
number of pedigree annotations
25
100
225
400

N

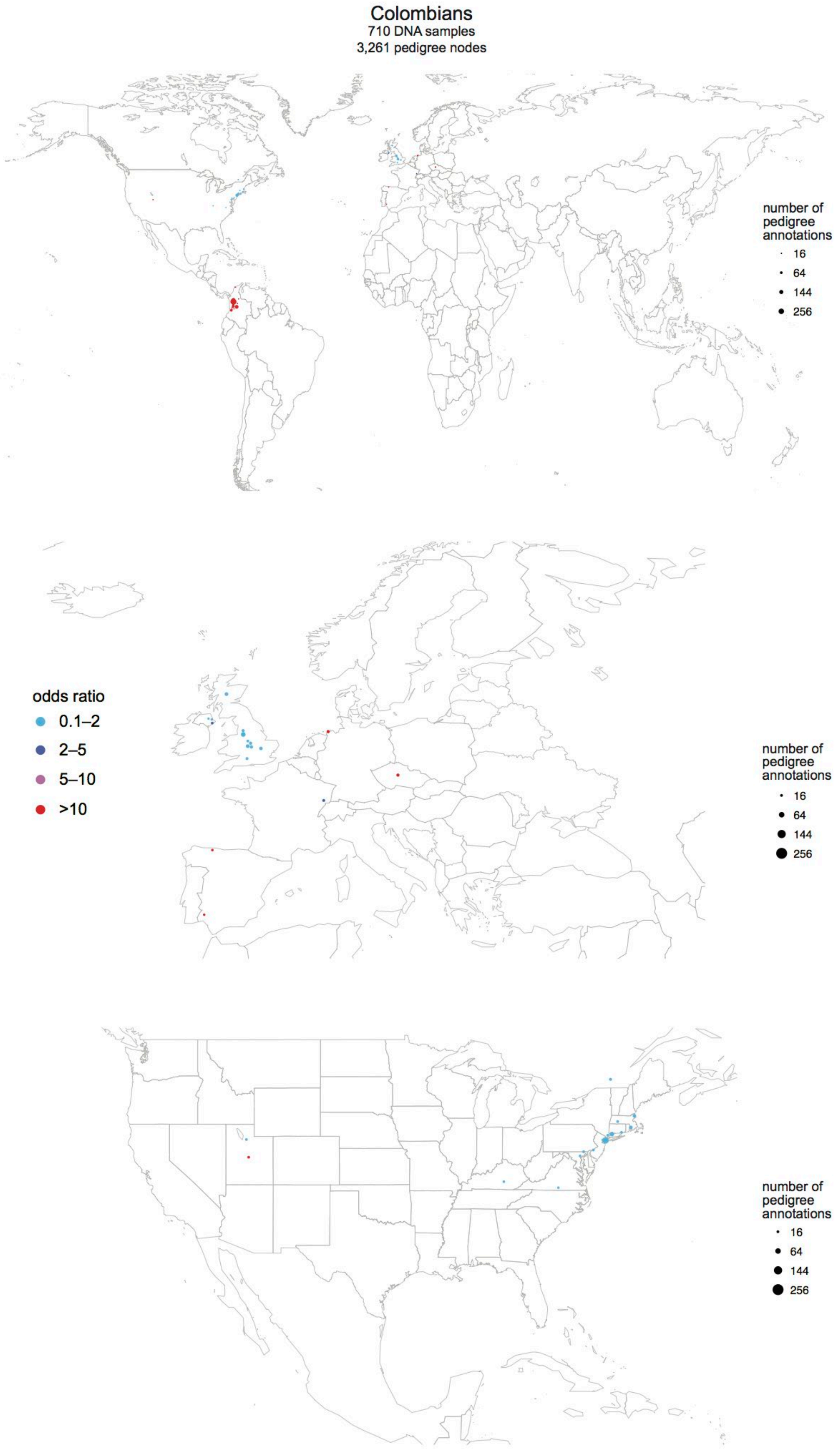
Colombians
710 DNA samples
3,261 pedigree nodes
number of pedigree annotations
16
64
144
256
odds ratio
0.1–2
2–5
5–10
>10
number of pedigree annotations
16
64
144
256
number of pedigree annotations
16
64
144
256

O

European Jewish
26,547 DNA samples
261,655 pedigree nodes

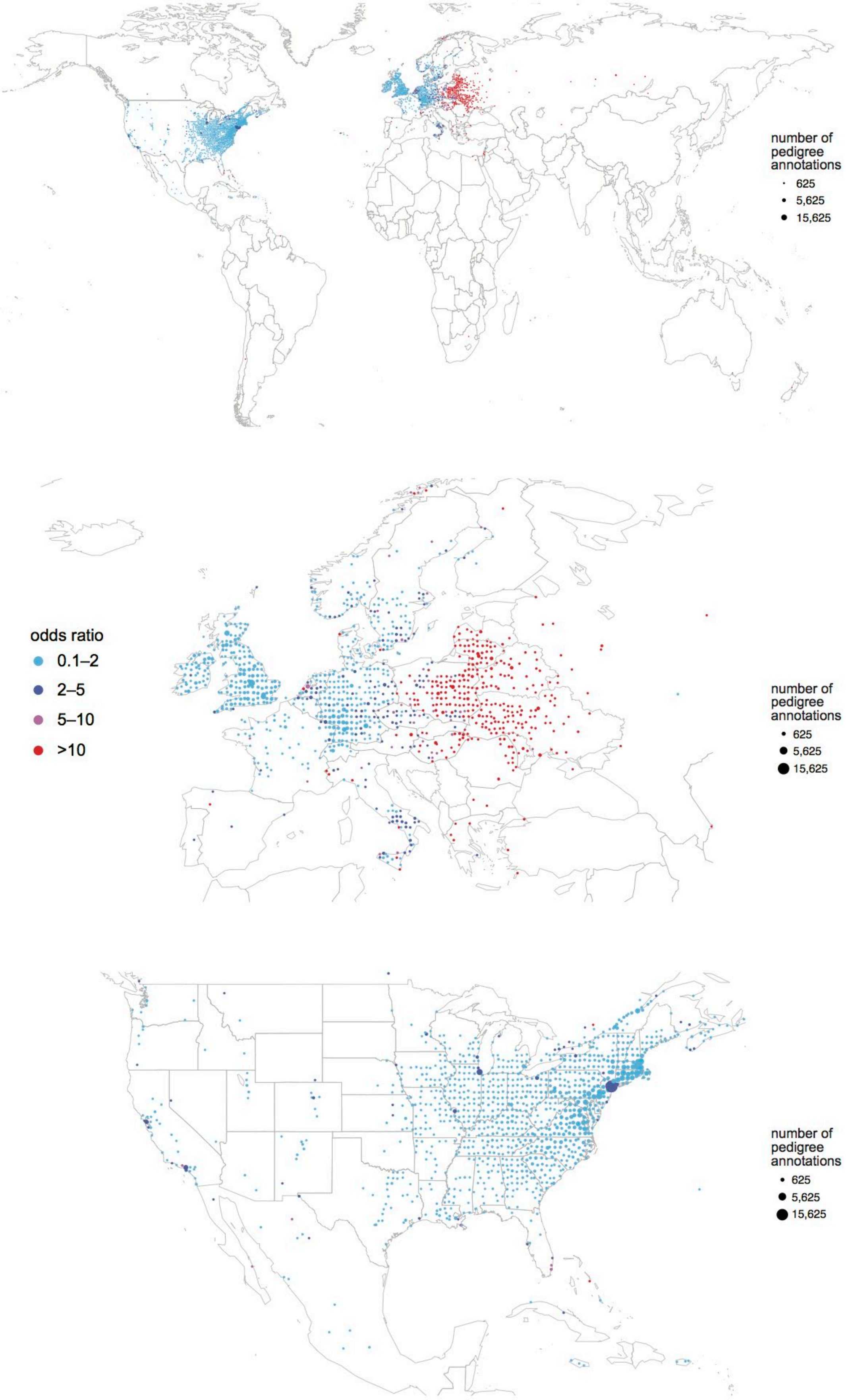

P

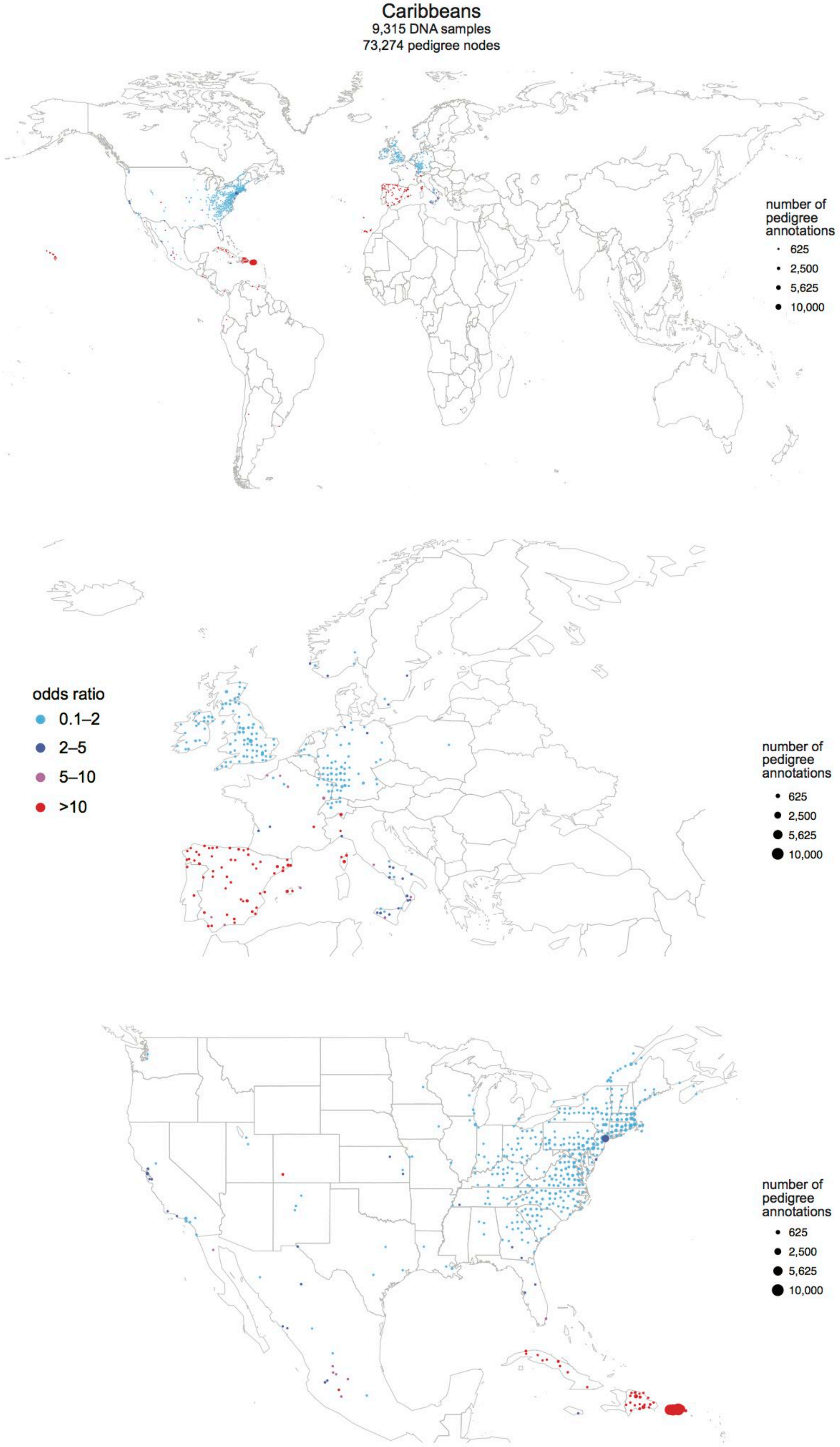
Caribbeans
9,315 DNA samples
73,274 pedigree nodes
number of pedigree annotations
625
2,500
5,625
10,000
odds ratio
0.1–2
2–5
5–10
>10
number of pedigree annotations
625
2,500
5,625
10,000
number of pedigree annotations
625
2,500
5,625
10,000

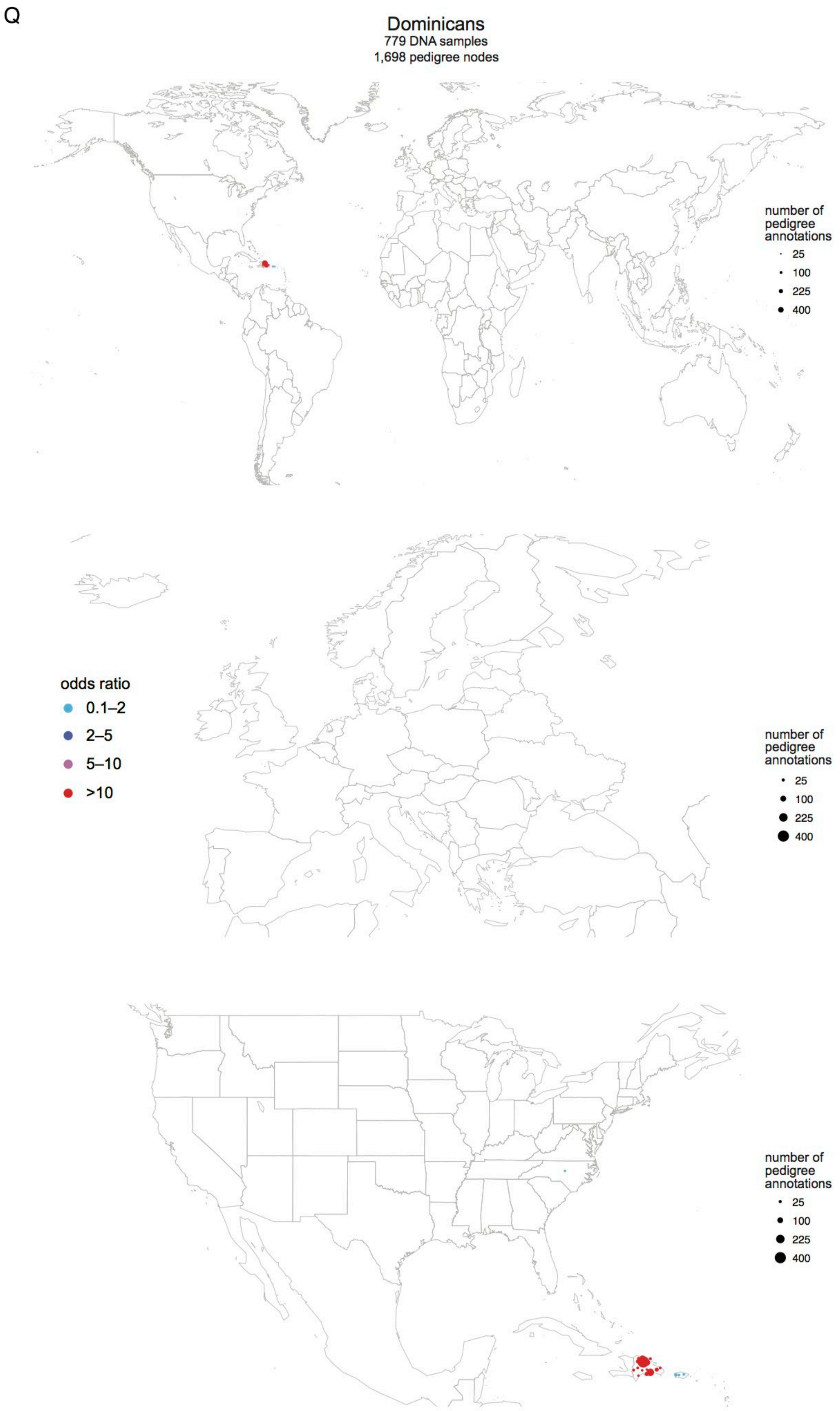
Q
Dominicans
779 DNA samples
1,698 pedigree nodes
number of pedigree annotations
25
100
225
400
odds ratio
0.1–2
2–5
5–10
>10
number of pedigree annotations
25
100
225
400
number of pedigree annotations
25
100
225
400

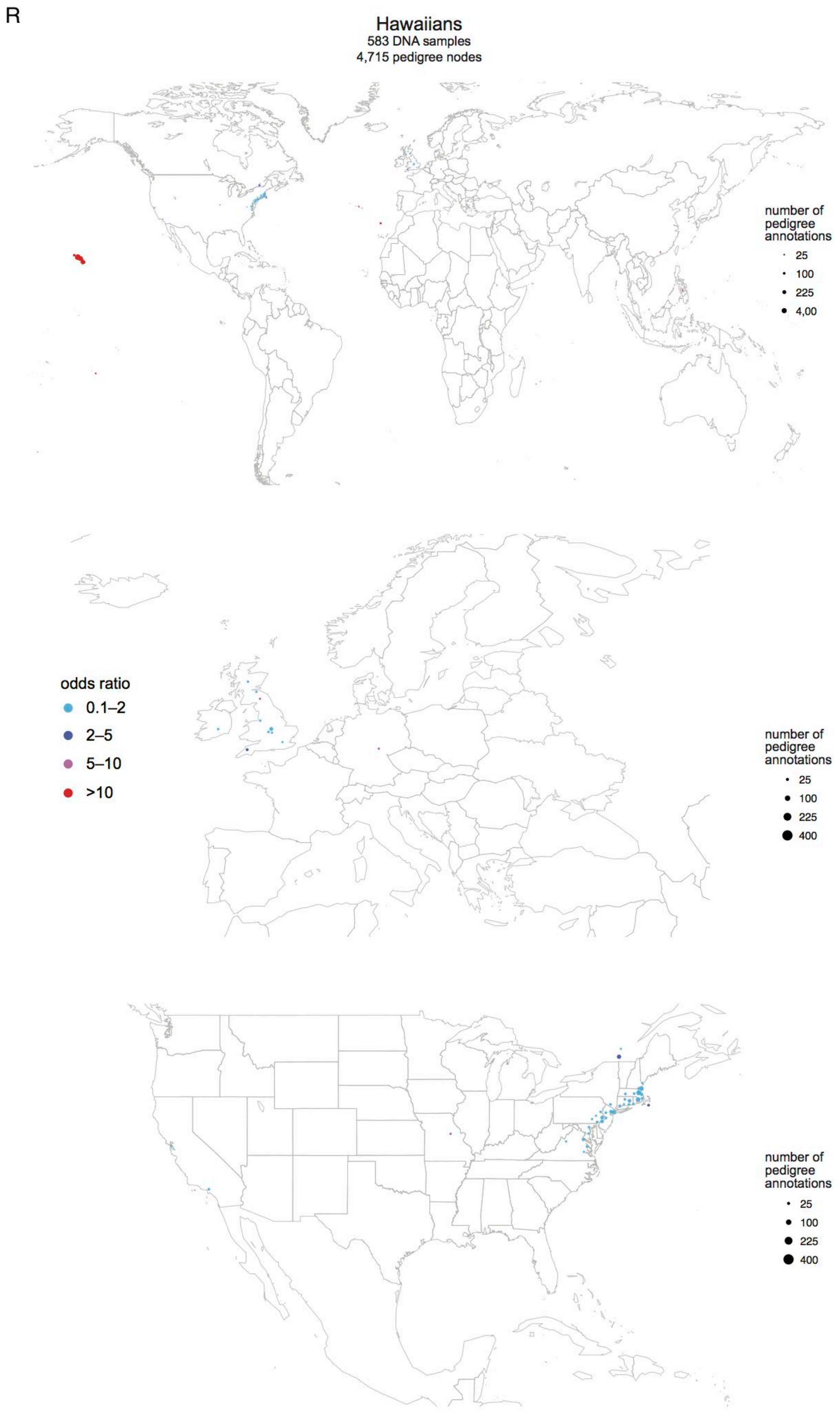
R
Hawaiians
583 DNA samples
4,715 pedigree nodes
number of pedigree annotations
25
100
225
4,00
odds ratio
0.1–2
2–5
5–10
>10
number of pedigree annotations
25
100
225
400
number of pedigree annotations
25
100
225
400

Supplementary Figure 20 | Distribution of ancestral birth locations worldwide, in North America, and in Europe, for each stable subset identified in spectral embedding. See description of Supplementary Fig. 18 for details about this figure. All maps in our figures were generated with the *maps* R package using data from the Natural Earth Project (1:50m world map, version 2.0). These data are made available in the public domain (Creative Commons CC0).

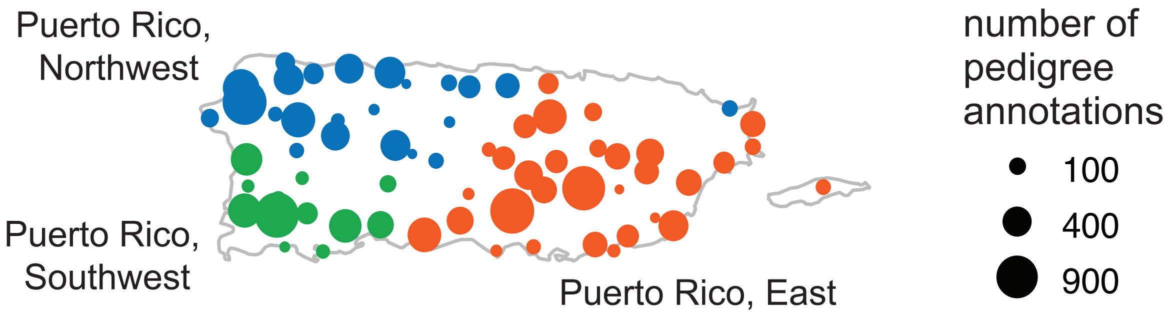

Supplementary Figure 21 | Distribution of ancestral birth locations for the 3 clusters with a high concentration of birth locations on the Island of Puerto Rico. East Puerto Rico cluster (orange), Northwest Puerto Rico cluster (blue) and Southwest Puerto Rico cluster (green) are shown. Maps are plotted as in Supplementary Figs. 16–18, except that only locations with *OR* > 2 are shown, and grid points are placed every 0.1 degrees of latitude and longitude. All maps in our figures were generated with the *maps* R package using data from the Natural Earth Project (1:50m world map, version 2.0). These data are made available in the public domain (Creative Commons CC0).

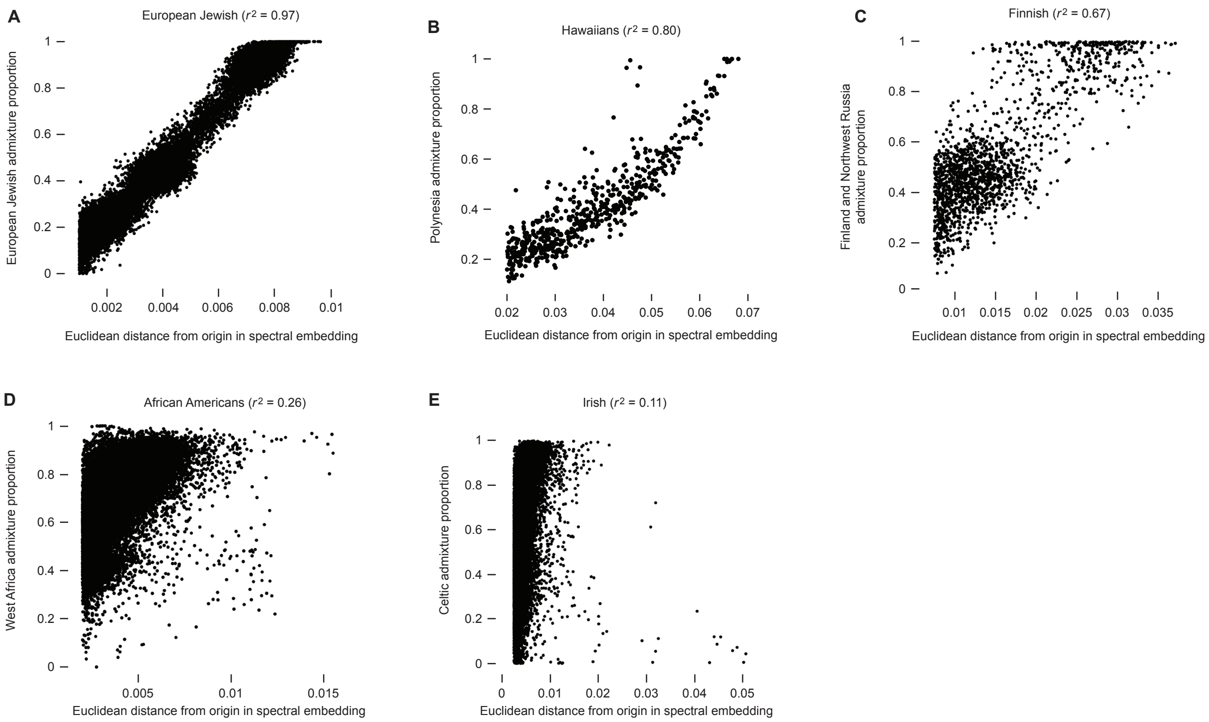

Supplementary Figure 22 | Scatterplots of distance from origin in spectral embedding versus admixture proportions demonstrate relationship between IBD and global population structure. Each panel shows admixture proportions estimated using ADMIXTURE for all genotyped individuals assigned to the stable subset against the Euclidean distance from the origin in the spectral embedding. The distance is calculated only using the dimensions (eigenvectors) given in Supplementary Data 2.

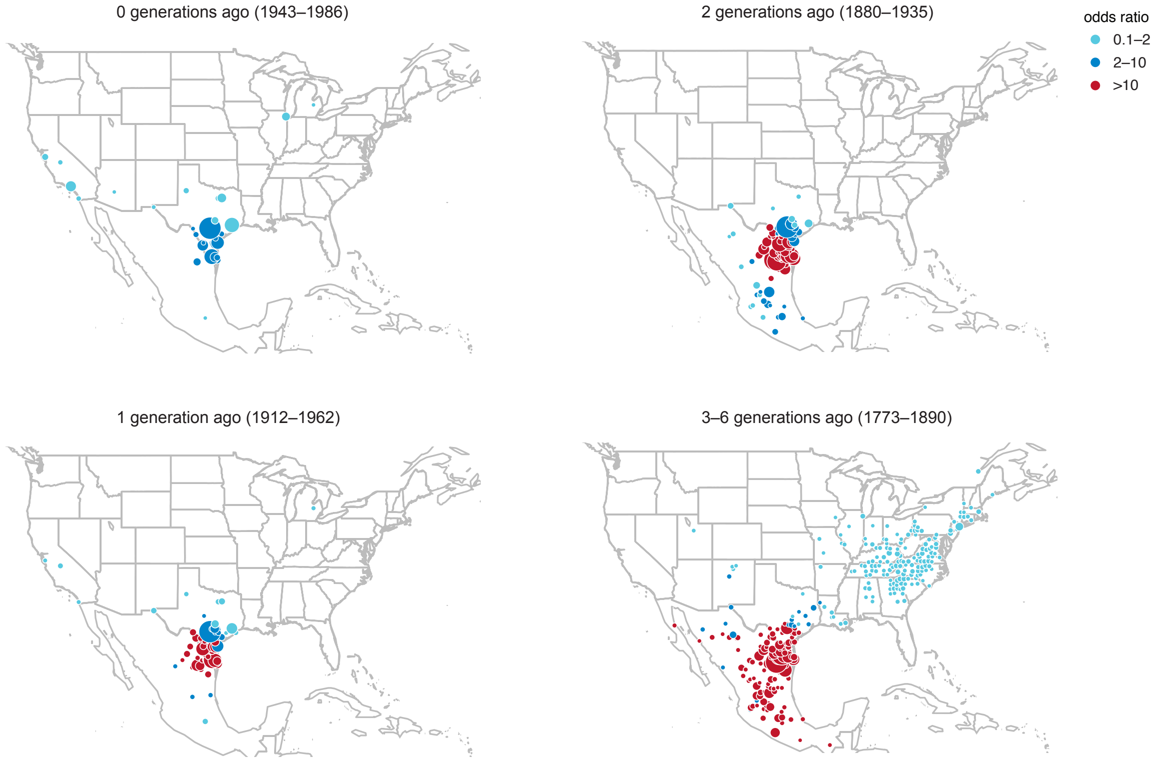

Supplementary Figure 23 | Genealogical data capture immigration patterns from Northeast Mexico to the US. In particular, birth locations in recent generations show a particularly large concentration in South Texas. The size of each point is scaled by number of pedigree birth location annotations, separately for each of the 4 maps. Date ranges are obtained from the 5th and 9th percentile of the birth year annotations. For more details, see description of Fig. 2. All maps in our figures were generated with the *maps* R package using data from the Natural Earth Project (1:50m world map, version 2.0). These data are made available in the public domain (Creative Commons CC0).

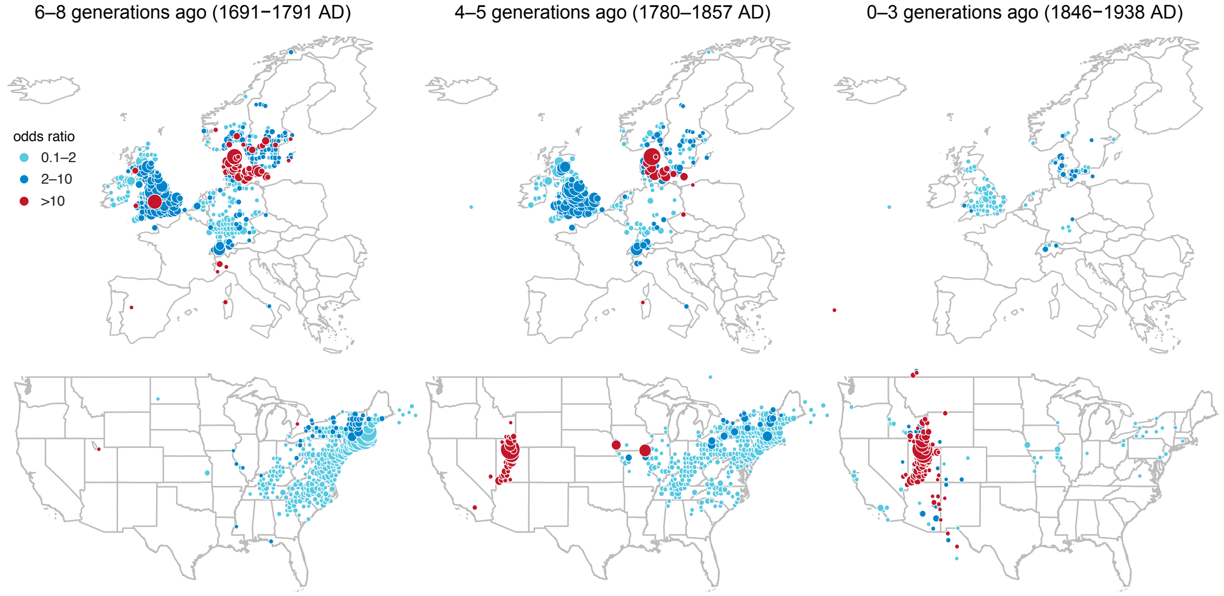

Supplementary Figure 24 | Genealogical data by generation trace US migration and European origins of Utah cluster. The size of each point is scaled by number of pedigree birth location annotations, separately for each of the 6 maps. Date ranges are the 5th and 95th percentiles of the birth year annotations. For more details, see description of Fig. 2. Note that not all current political borders are shown. All maps in our figures were generated with the *maps* R package using data from the Natural Earth Project (1:50m world map, version 2.0). These data are made available in the public domain (Creative Commons CC0).

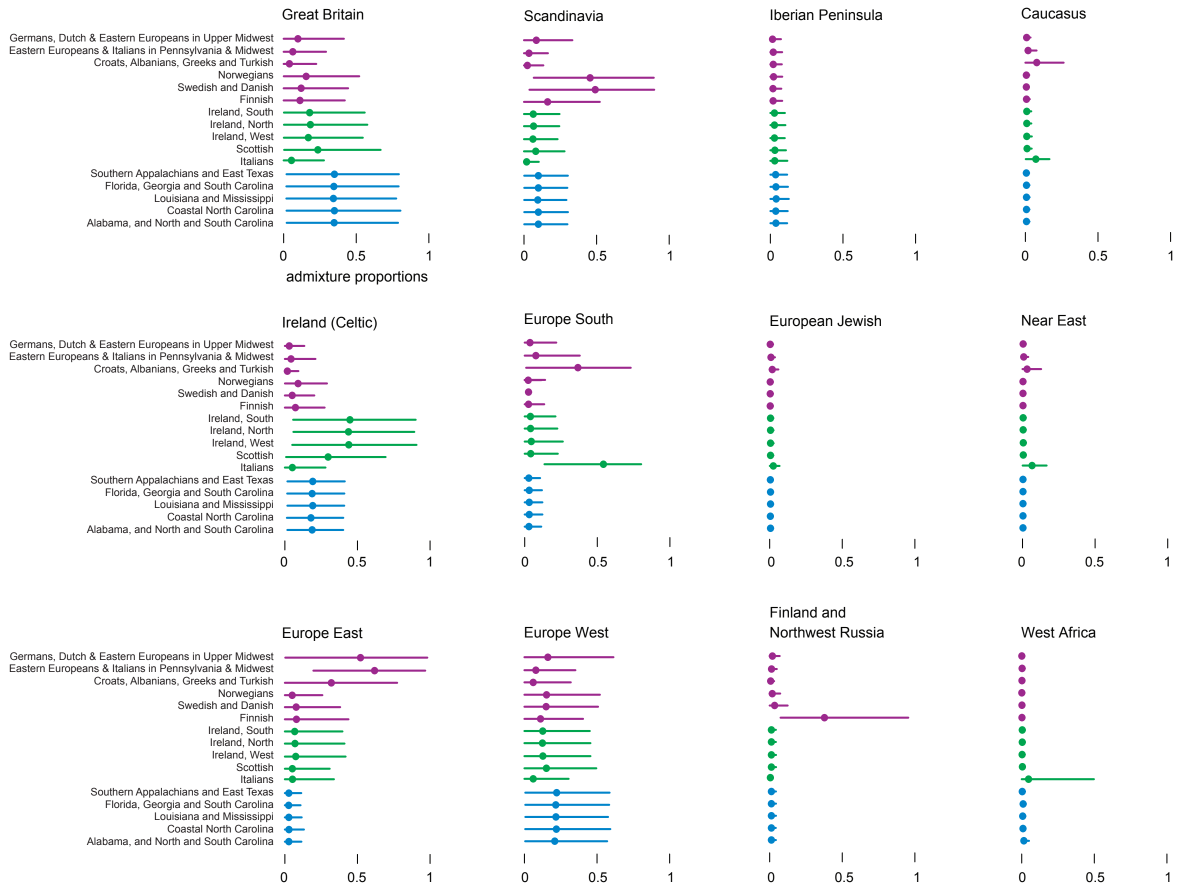

Supplementary Figure 25 | Admixture proportions in selected third-level clusters. Results are shown for the largest clusters detected in the sub-networks defined by the following second-level clusters (see Supplementary Data 2): Midwest immigrants (shown in purple); Italian, Irish, Scottish and Atlantic Canada (green); and Lower South (light blue). Filled circles correspond to mean admixture proportions, and error bars give [0.05,0.95] credible intervals. Admixture statistics are only shown for ancestral populations in which the mean admixture proportion is >1% in at least one cluster.

A

Germans, Dutch and Eastern Europeans in Upper Midwest
16,918 DNA samples
153,560 pedigree nodes

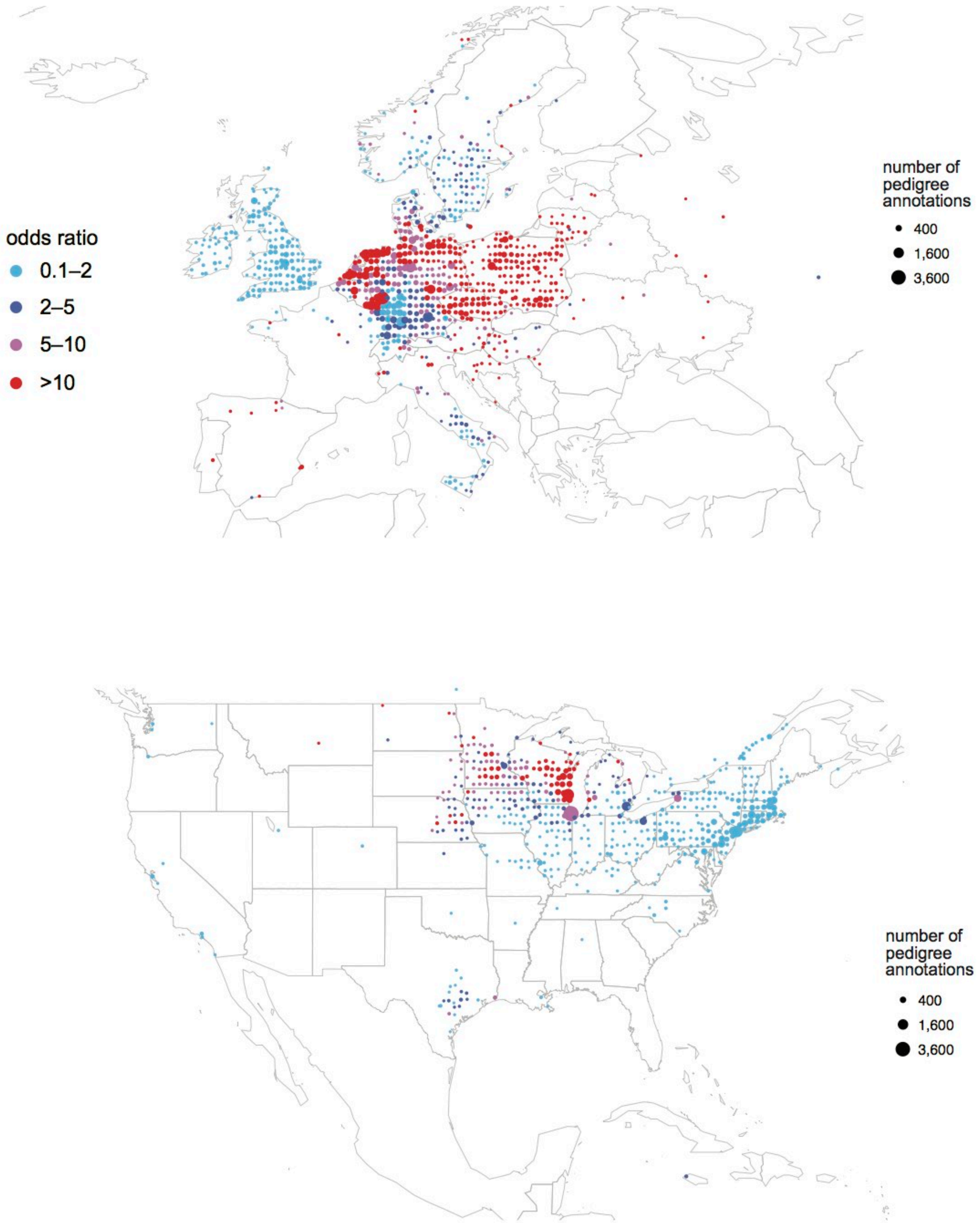

B

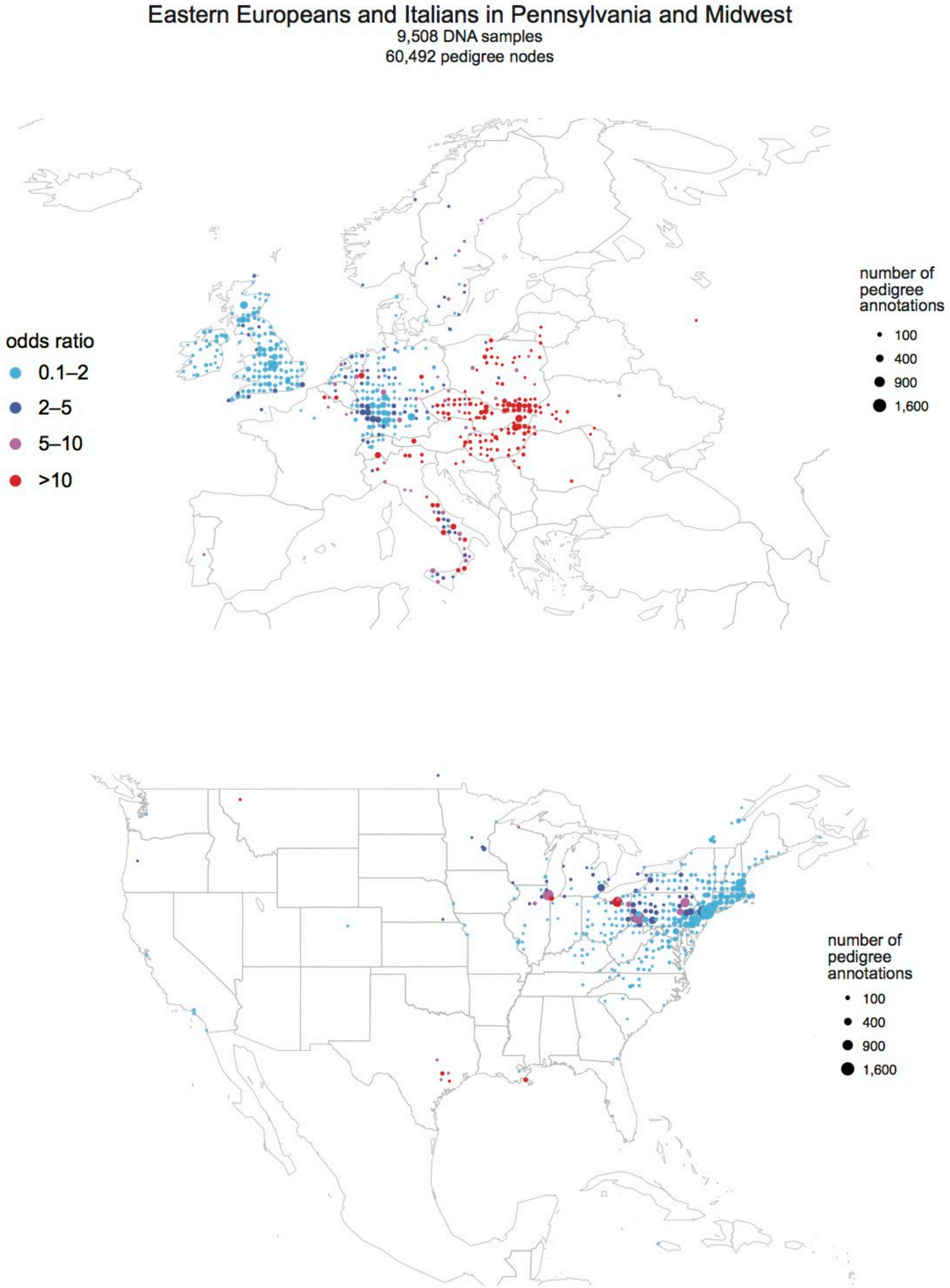

Eastern Europeans and Italians in Pennsylvania and Midwest
9,508 DNA samples
60,492 pedigree nodes
odds ratio
0.1–2
2–5
5–10
>10
number of pedigree annotations
100
400
900
1,600
number of pedigree annotations
100
400
900
1,600

C

Norwegians
11,895 DNA samples
293,878 pedigree nodes

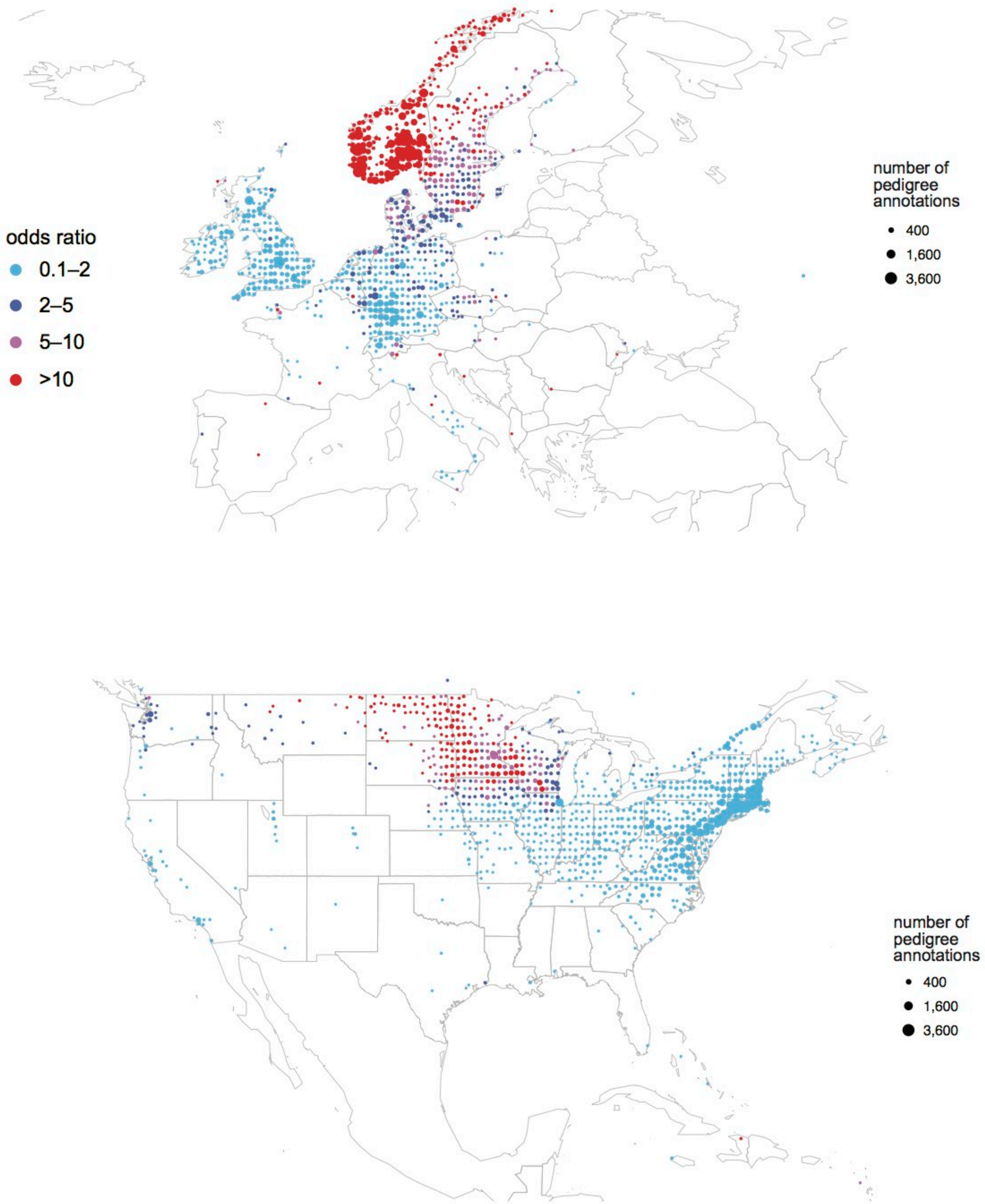

D

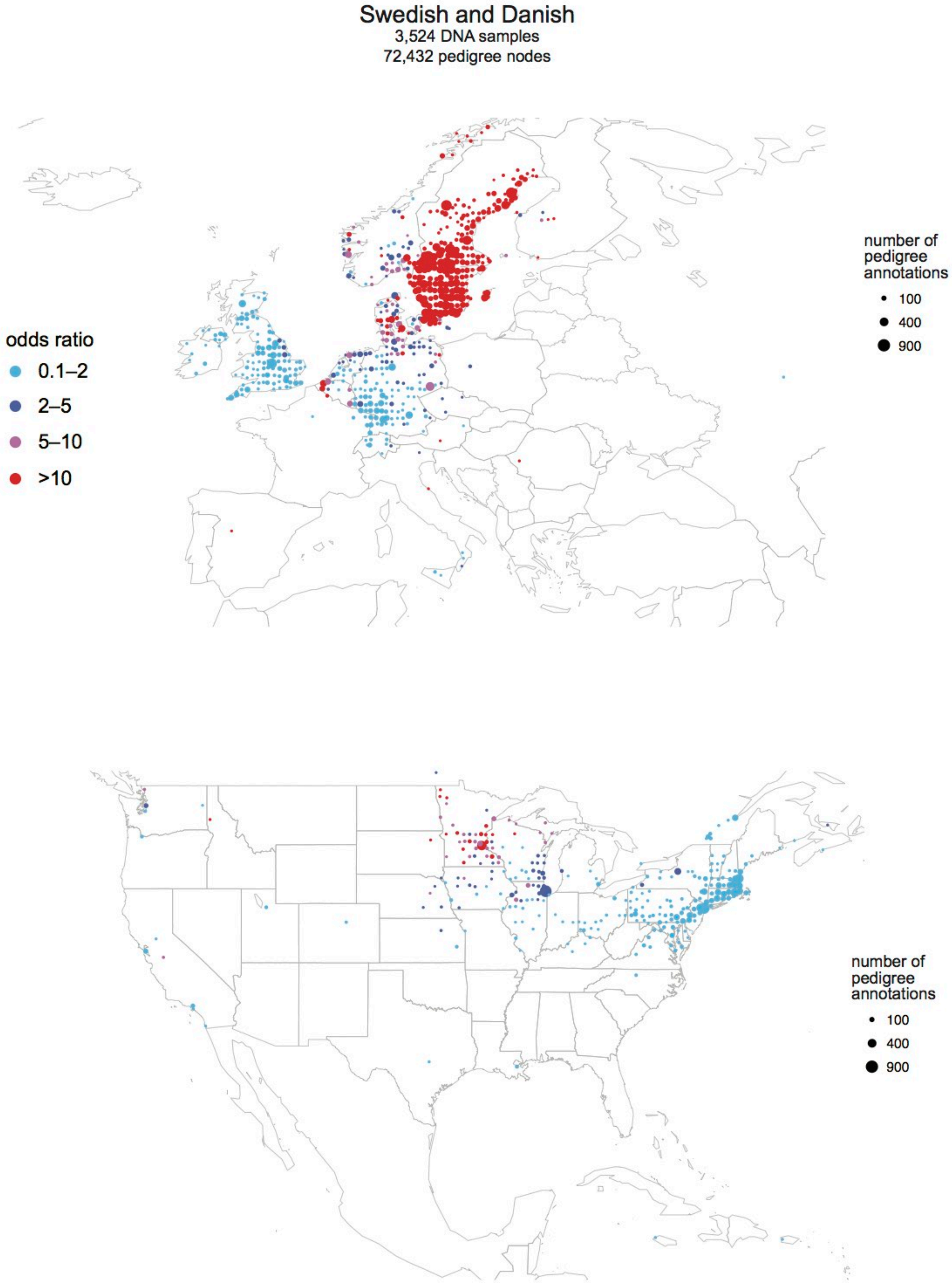
Swedish and Danish
3,524 DNA samples
72,432 pedigree nodes
odds ratio
0.1–2
2–5
5–10
>10
number of pedigree annotations
100
400
900
number of pedigree annotations
100
400
900

E

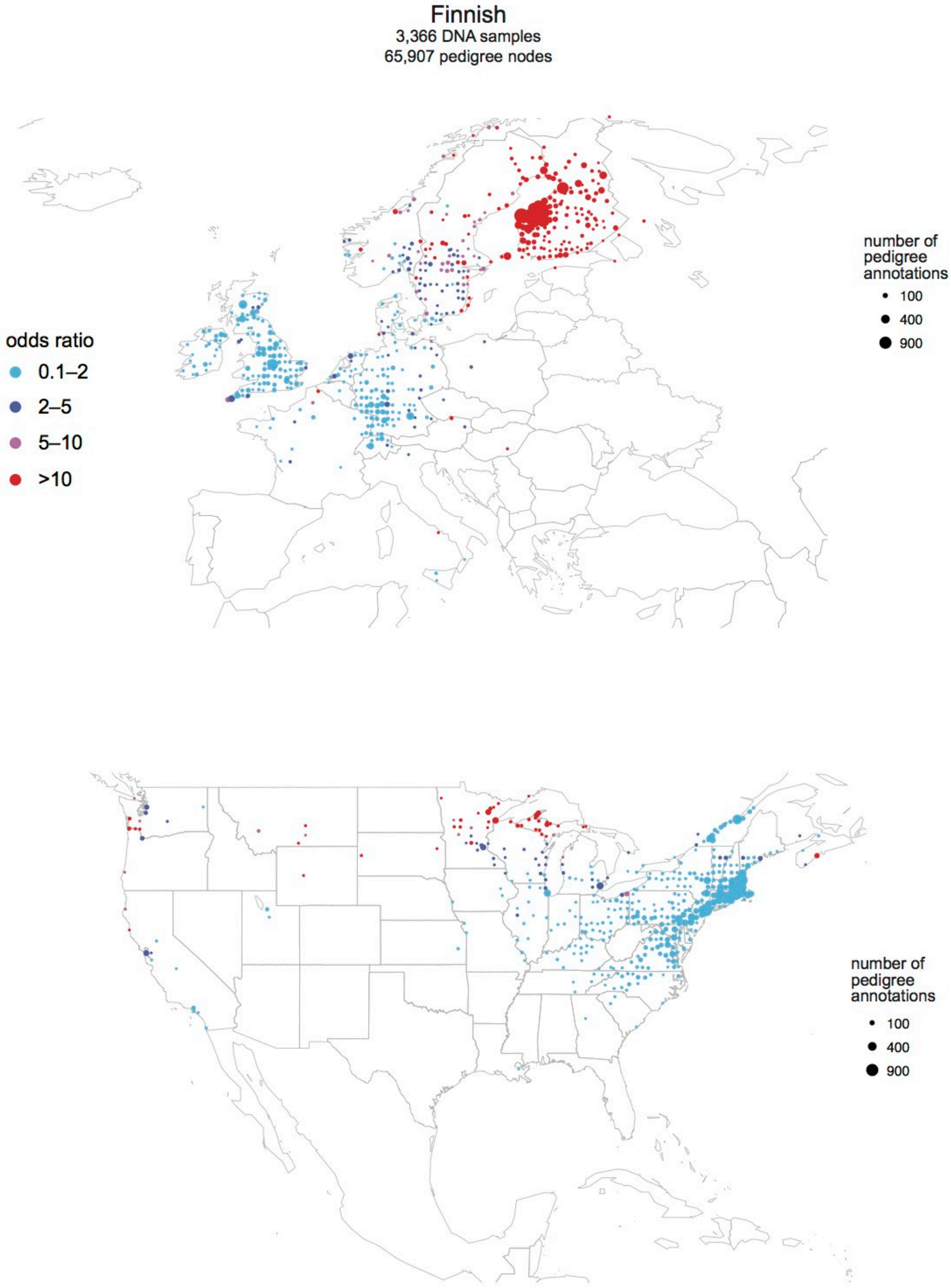
Finnish
3,366 DNA samples
65,907 pedigree nodes
odds ratio
0.1–2
2–5
5–10
>10
number of pedigree annotations
100
400
900
number of pedigree annotations
100
400
900

F

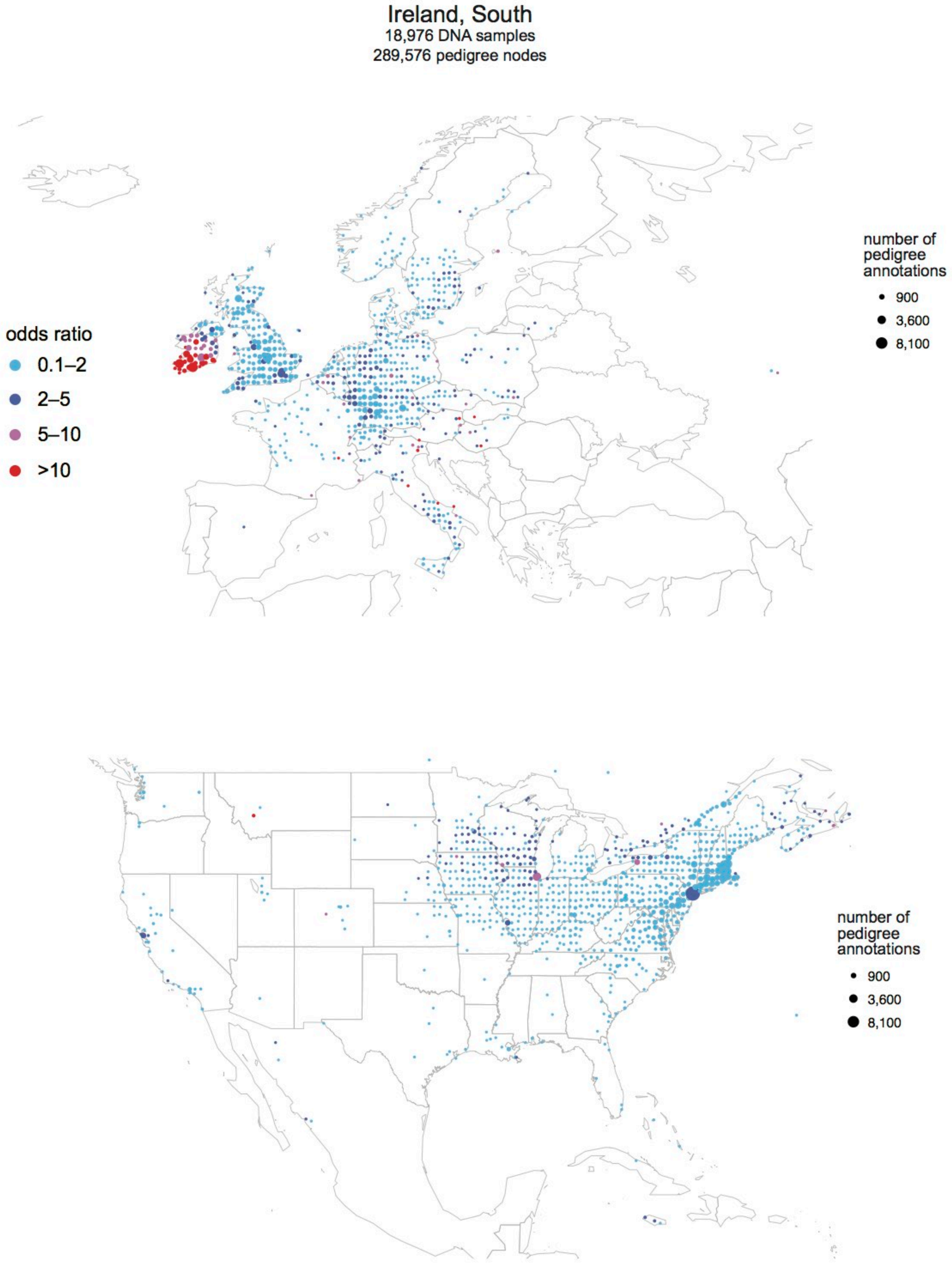
Ireland, South
18,976 DNA samples
289,576 pedigree nodes
odds ratio
0.1–2
2–5
5–10
>10
number of pedigree annotations
900
3,600
8,100
number of pedigree annotations
900
3,600
8,100

G

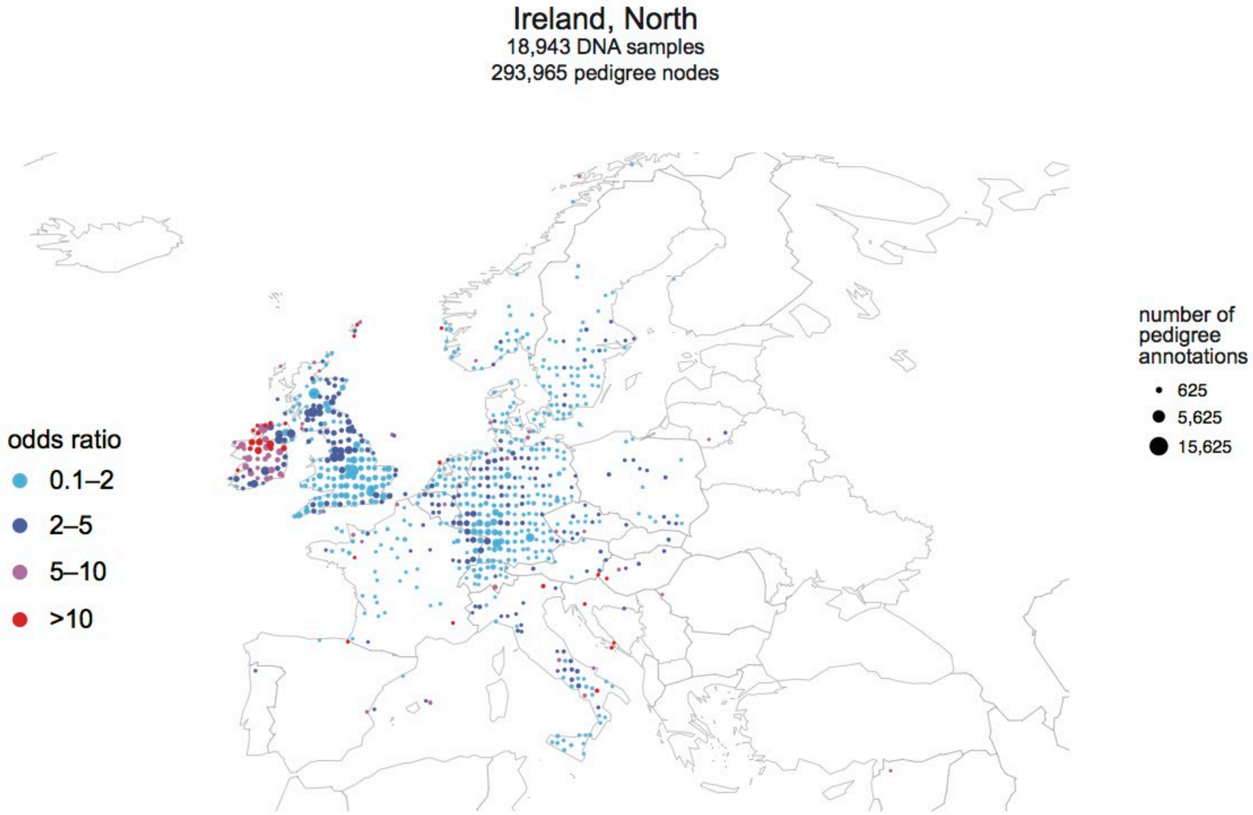
Ireland, North
18,943 DNA samples
293,965 pedigree nodes
odds ratio
0.1–2
2–5
5–10
>10
number of
pedigree
annotations
625
5,625
15,625

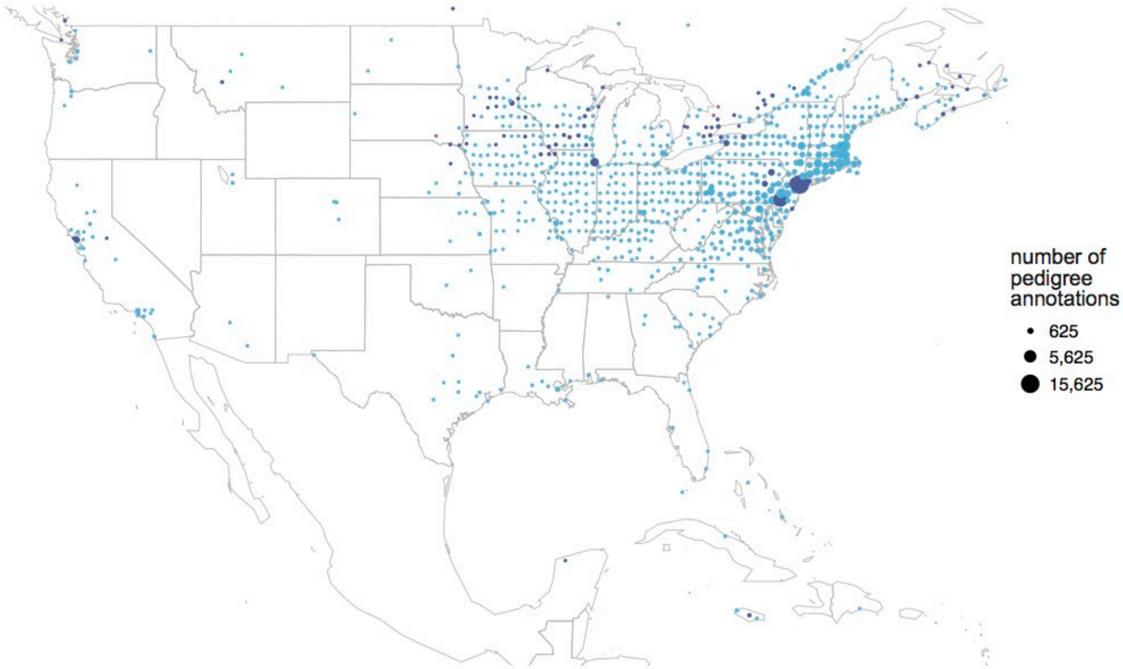
number of
pedigree
annotations
625
5,625
15,625

H

Ireland, West

10,851 DNA samples
165,833 pedigree nodes

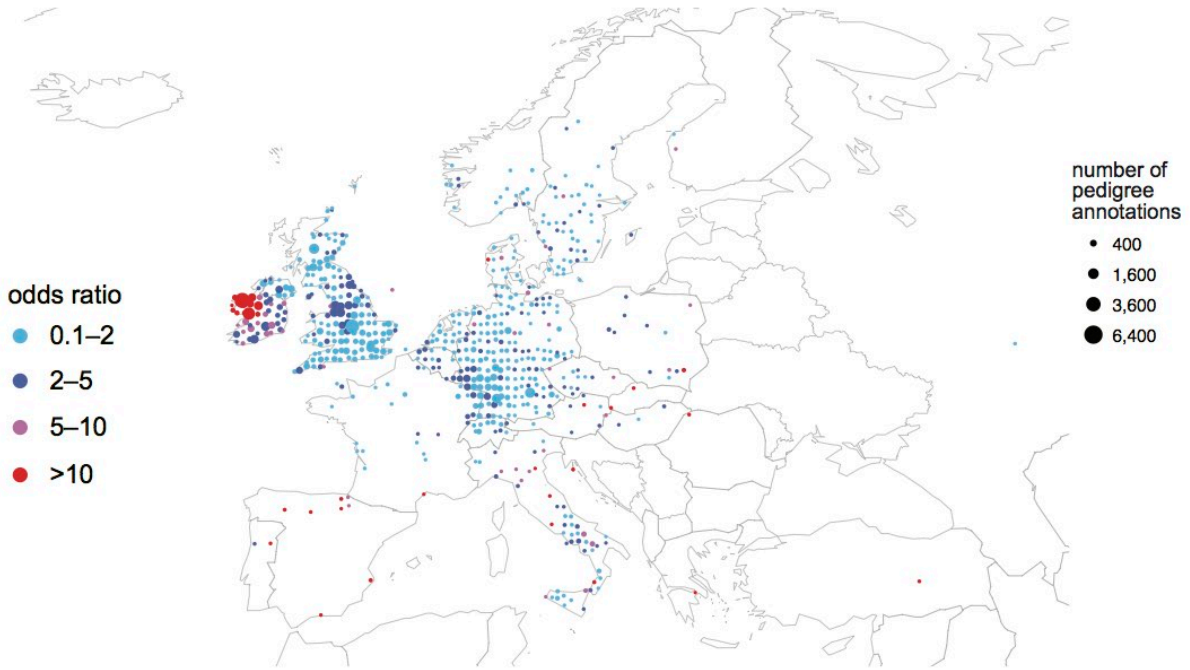

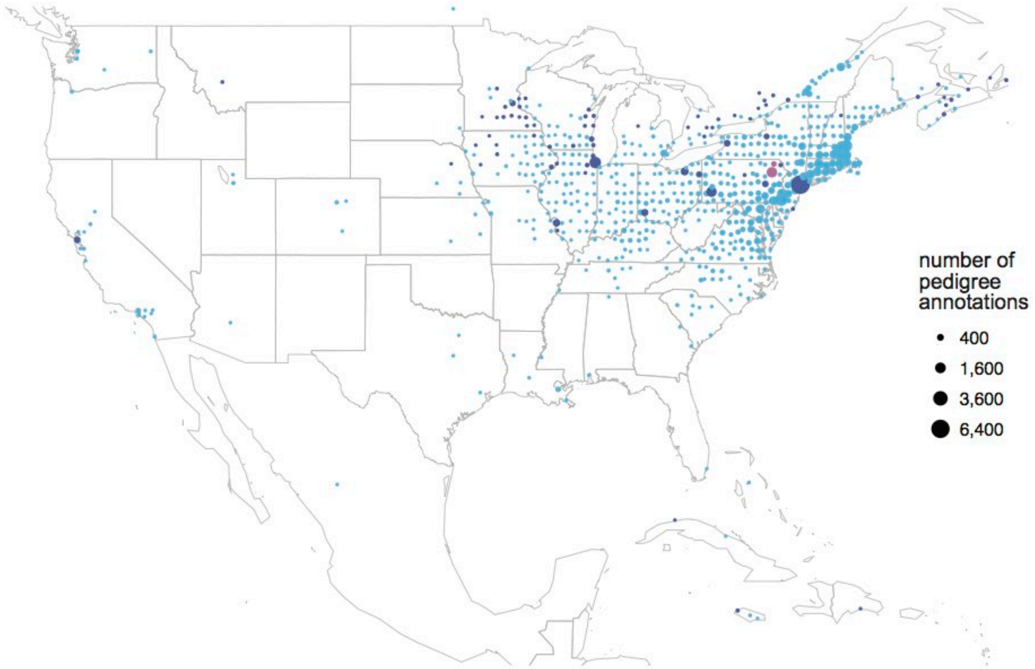

l

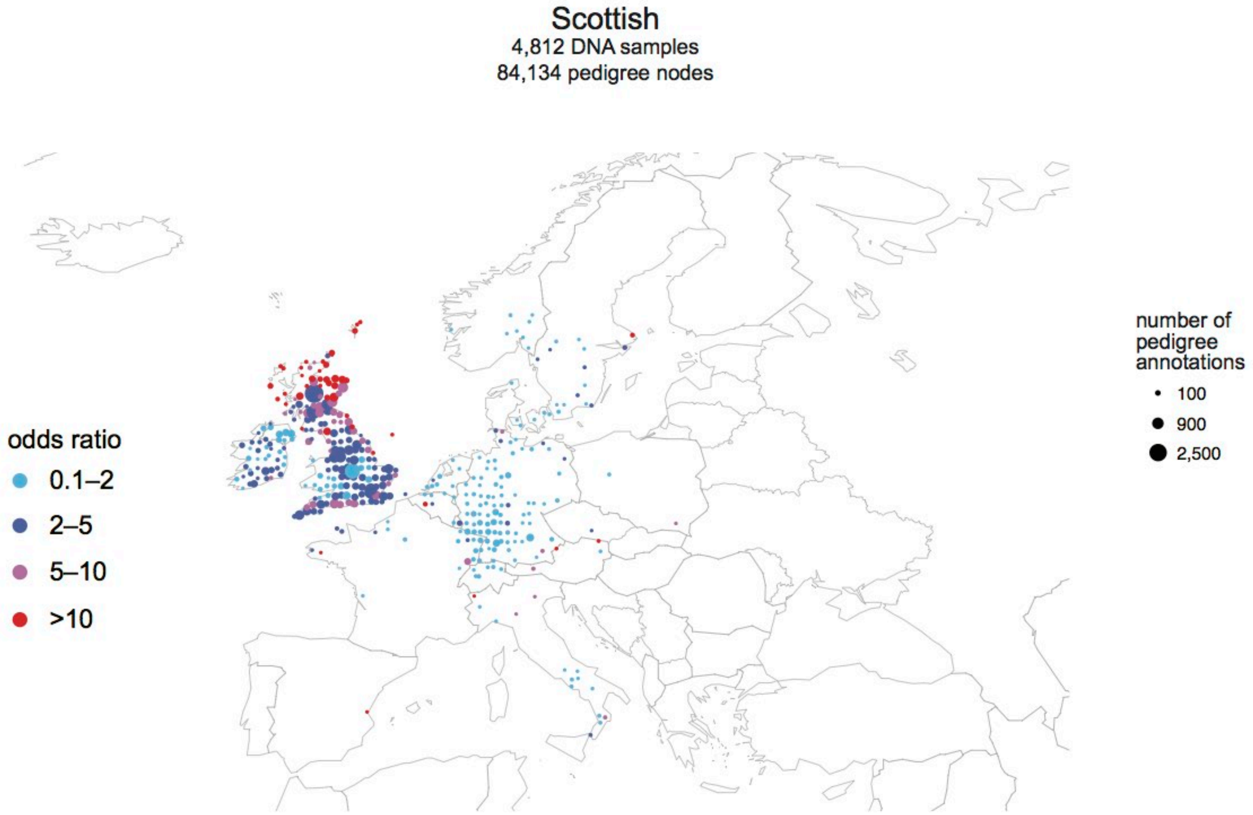
Scottish
4,812 DNA samples
84,134 pedigree nodes
odds ratio
0.1–2
2–5
5–10
>10
number of
pedigree
annotations
100
900
2,500

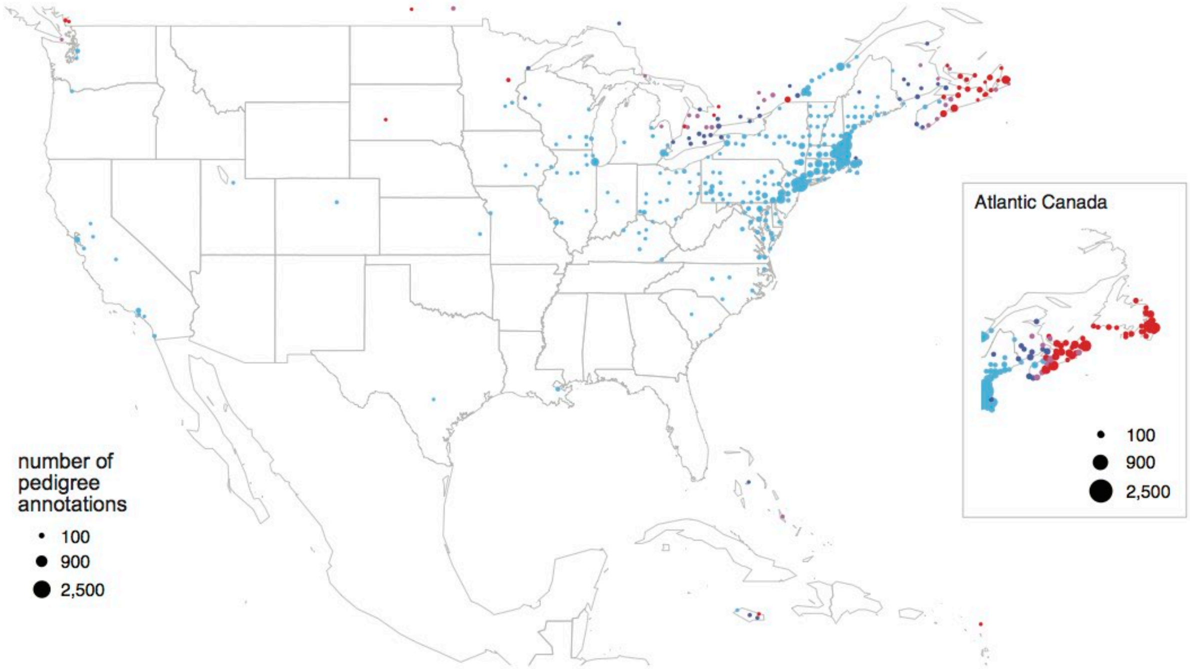
Atlantic Canada
100
900
2,500
number of
pedigree
annotations
100
900
2,500

J

Italians

18,261 DNA samples
125,807 pedigree nodes

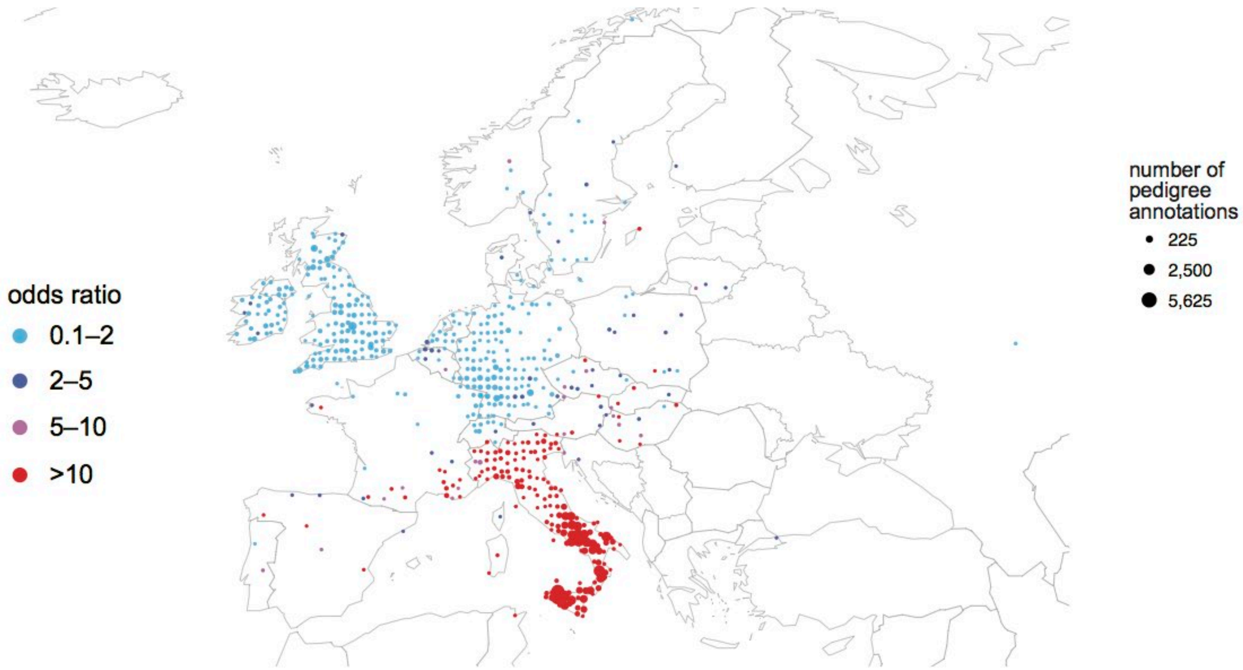

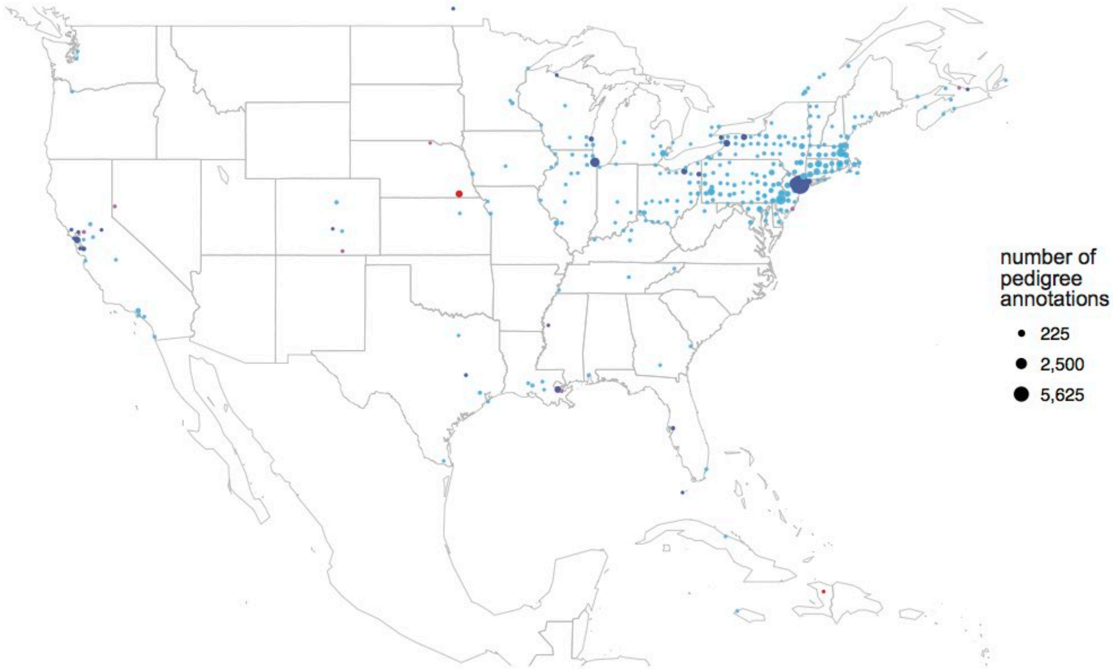

K

Croats, Albanians, Greeks and Turkish

3,532 DNA samples
12,702 pedigree nodes

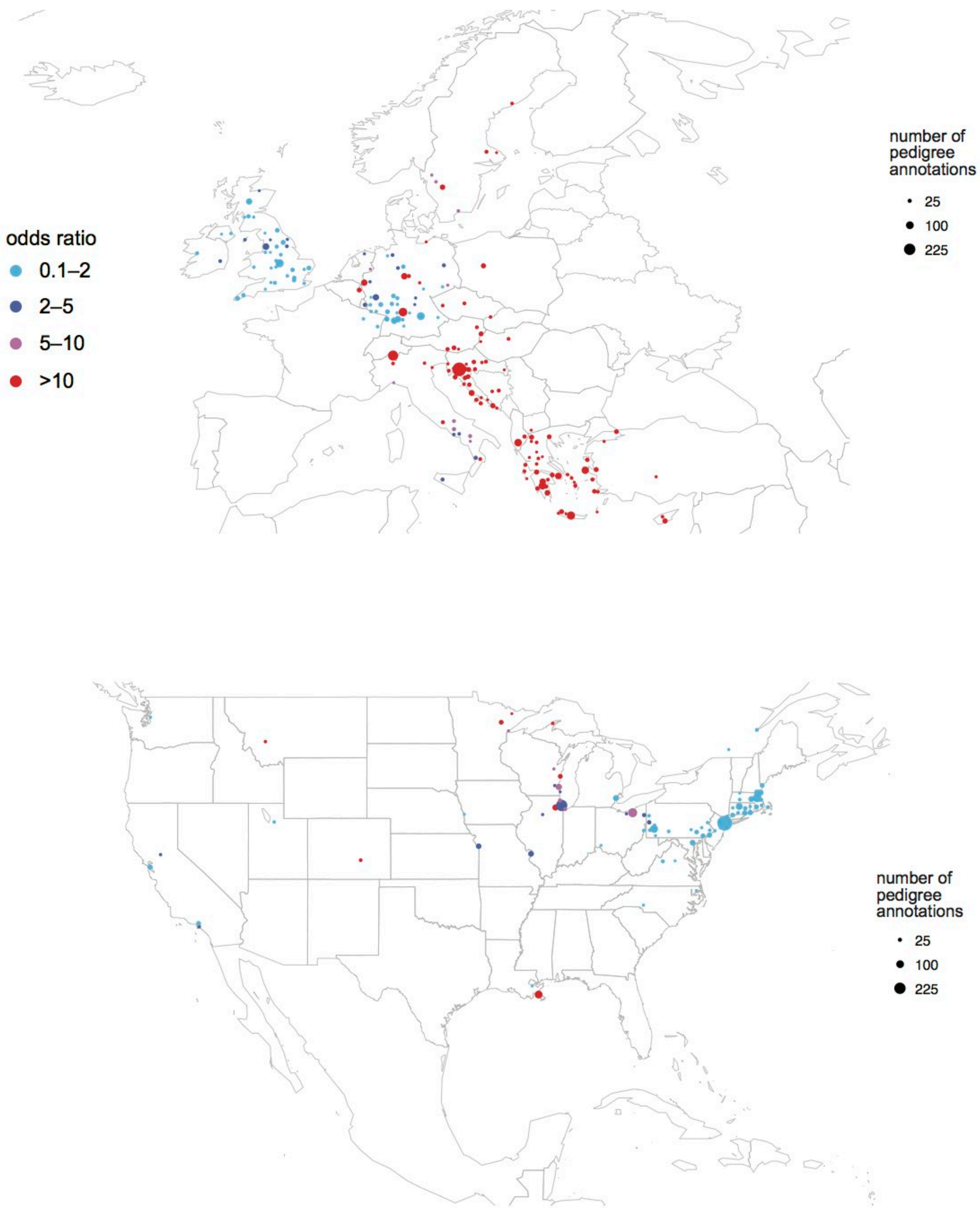

Supplementary Figure 26 | Distribution of ancestral birth locations in North America and in Europe for selected third-level clusters. Results are shown for the largest clusters detected in the sub-networks defined by these two second-level clusters: Midwest immigrants; Italian, Irish, Scottish and Atlantic Canada (see Supplementary Data 2). See description of Supplementary Fig. 18 for more details. Note that there is sometimes uncertainty about the most appropriate cluster label based on the genealogical data. When we are uncertain, we typically choose a simpler label that fits most of the genealogical data, but we point out that this label may not accurately characterize some portion of the cluster. All maps in our figures were generated with the *maps* R package using data from the Natural Earth Project (1:50m world map, version 2.0). These data are made available in the public domain (Creative Commons CC0).

A

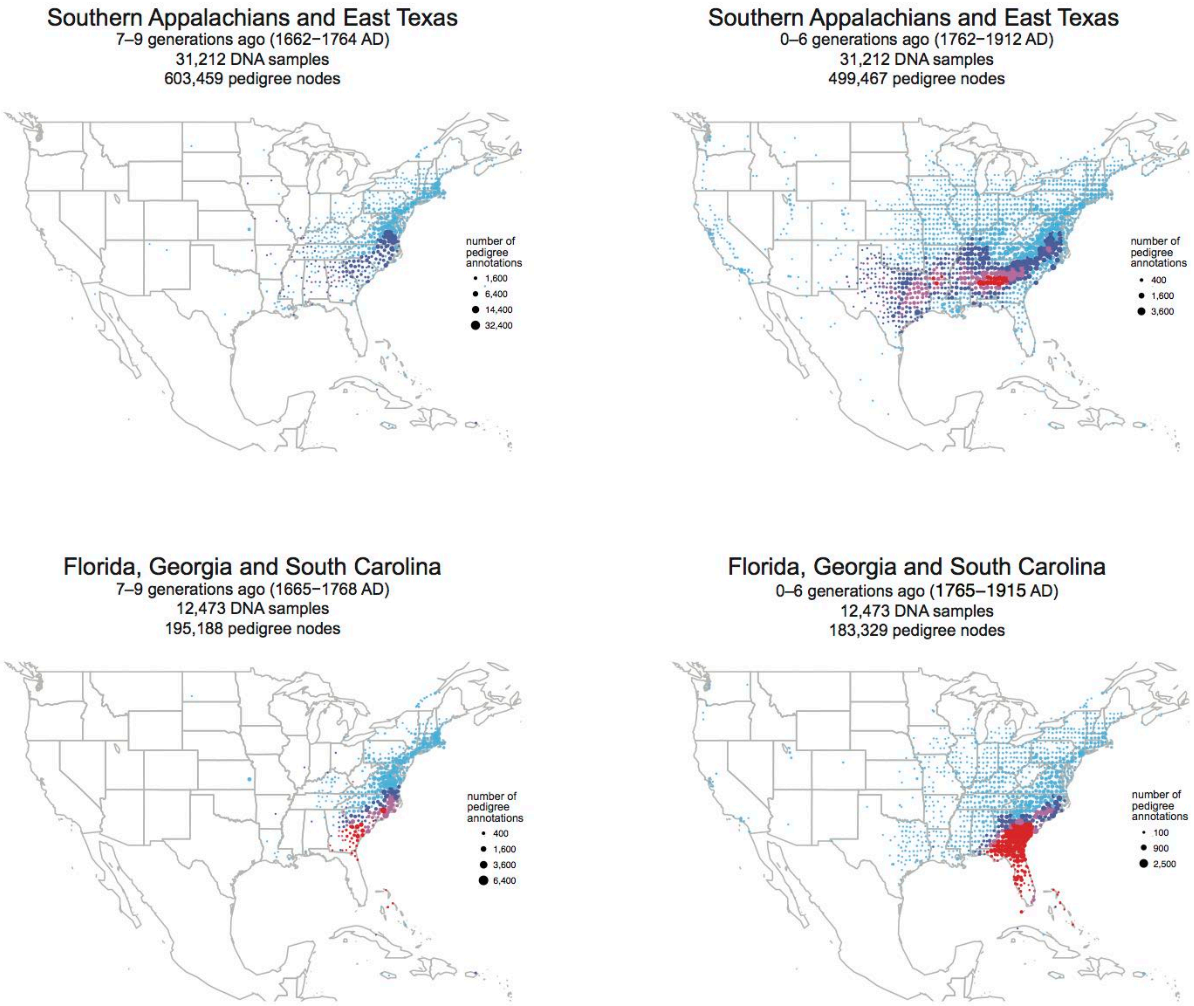
Southern Appalachians and East Texas
7–9 generations ago (1662–1764 AD)
31,212 DNA samples
603,459 pedigree nodes
number of pedigree annotations
1,600
6,400
14,400
32,400
Southern Appalachians and East Texas
0–6 generations ago (1762–1912 AD)
31,212 DNA samples
499,467 pedigree nodes
number of pedigree annotations
400
1,600
3,600
Florida, Georgia and South Carolina
7–9 generations ago (1665–1768 AD)
12,473 DNA samples
195,188 pedigree nodes
number of pedigree annotations
400
1,600
3,600
6,400
Florida, Georgia and South Carolina
0–6 generations ago (1765–1915 AD)
12,473 DNA samples
183,329 pedigree nodes
number of pedigree annotations
100
900
2,500

B

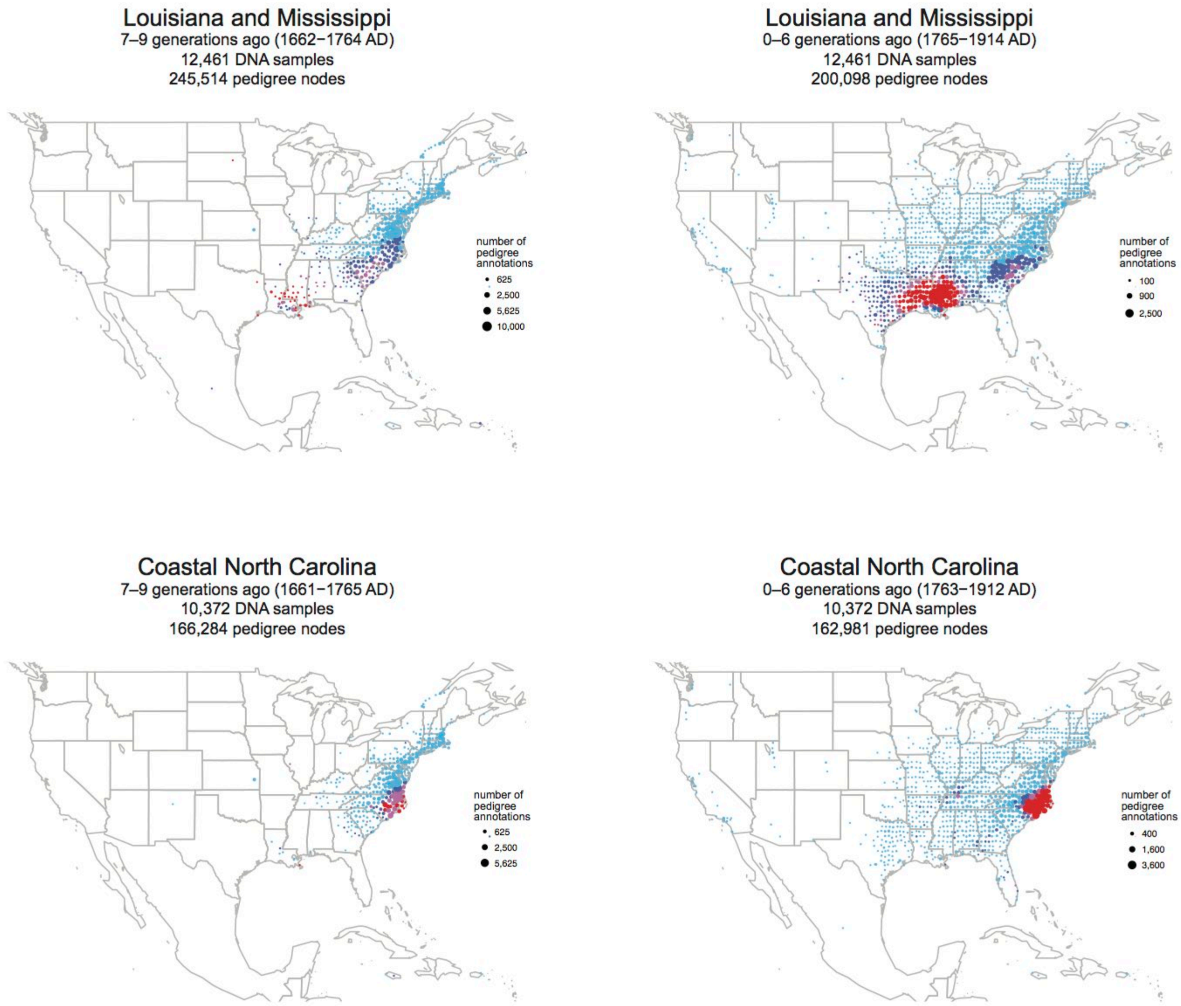

Louisiana and Mississippi
7–9 generations ago (1662–1764 AD)
12,461 DNA samples
245,514 pedigree nodes
number of pedigree annotations
625
2,500
5,625
10,000
Louisiana and Mississippi
0–6 generations ago (1765–1914 AD)
12,461 DNA samples
200,098 pedigree nodes
number of pedigree annotations
100
900
2,500
Coastal North Carolina
7–9 generations ago (1661–1765 AD)
10,372 DNA samples
166,284 pedigree nodes
number of pedigree annotations
625
2,500
5,625
Coastal North Carolina
0–6 generations ago (1763–1912 AD)
10,372 DNA samples
162,981 pedigree nodes
number of pedigree annotations
400
1,600
3,600

C

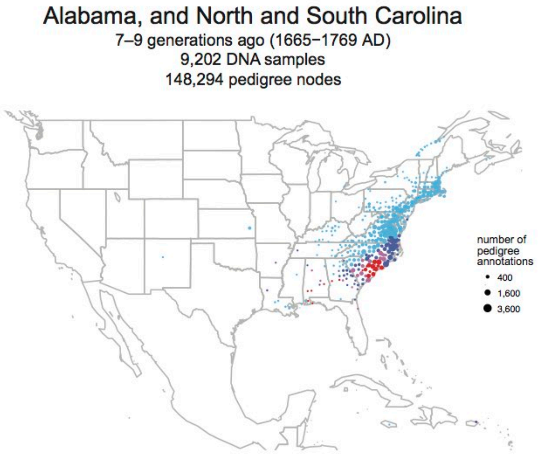
Alabama, and North and South Carolina
7–9 generations ago (1665−1769 AD)
9,202 DNA samples
148,294 pedigree nodes
number of pedigree annotations
400
1,600
3,600

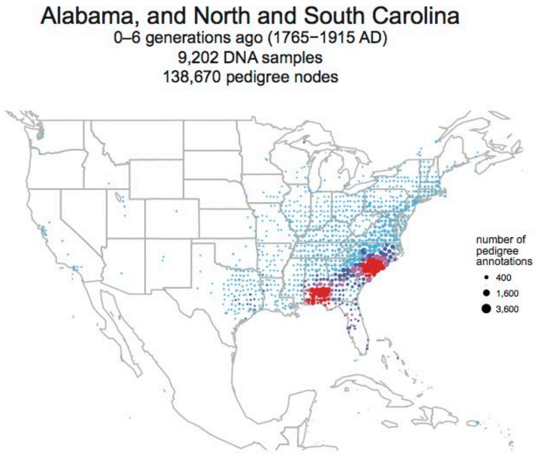
Alabama, and North and South Carolina
0–6 generations ago (1765−1915 AD)
9,202 DNA samples
138,670 pedigree nodes
number of pedigree annotations
400
1,600
3,600

Supplementary Figure 27 | Distribution of ancestral birth locations in North America for more selected third-level clusters. Results are shown for the largest clusters detected in the sub-network defined by the "Lower South" second-level cluster. Genealogical data are plotted separately for 0–6 generations ago and 7–9 generations ago to better highlight geographic concentration of ancestral birth locations. See description of Supplementary Fig. 18 for more details. Note that there is sometimes uncertainty about the most appropriate cluster label based on the genealogical data. When we are uncertain, we typically choose a simpler label that fits most of the genealogical data, but we point out that this label may not accurately characterize some portion of the cluster. All maps in our figures were generated with the *maps* R package using data from the Natural Earth Project (1:50m world map, version 2.0). These data are made available in the public domain (Creative Commons CC0).

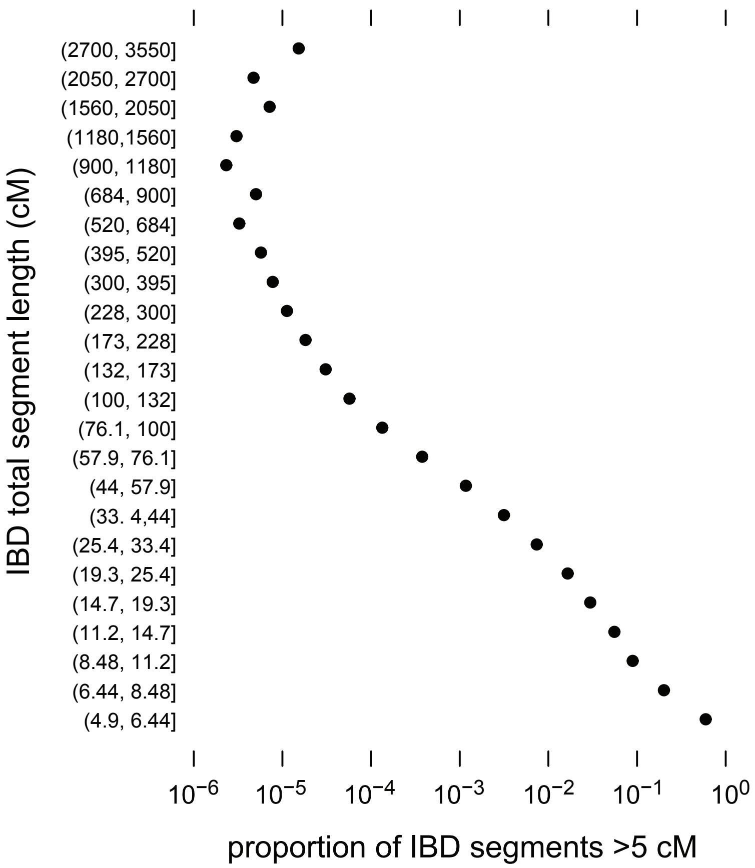

Supplementary Figure 28 | Global distribution of estimated IBD. Plot shows empirical distribution of total IBD (in cM) detected among 774,516 genotype samples, for all pairs with IBD > 5 cM. Note both axes are shown on the log-scale.

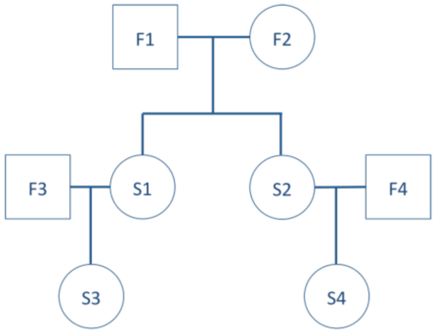

Supplementary Figure 29 | Example of pedigree used to simulate first cousins. All pedigree nodes labeled *Fn* correspond to genotyped individuals drawn at random from the set of customer genotype samples. Pedigree nodes labeled *Sn* correspond to simulated genotypes. To simulate a pair of first cousins, *S3* and *S4*, for example, we simulate a reproductive event between *F1* and *F2*, resulting in *S1*'s genotype, then we repeat the process to generate *S2*'s genotype. We continue this process to simulate the genotypes of *S3* and *S4* from the genotypes of *F3*, *F4*, *S1* and *S2*.

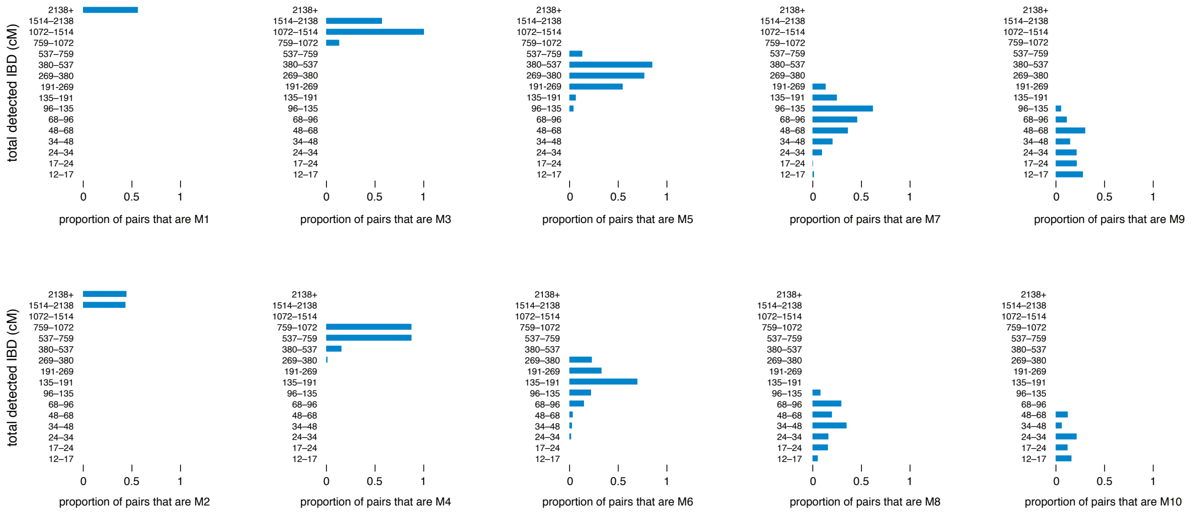

Supplementary Figure 30 | Distribution of total detected IBD (in cM) for different simulated familial relationships, grouped by number of separating meioses. One meiosis (abbreviated M1) corresponds to parent-child relationships, two meioses (abbreviated M2) corresponds to grandparent-child or (full) siblings, and so on. Each bar chart represents the conditional probability distribution Pr(number of separating meioses | total detected IBD). IBD detected in unrelated pairs, or pairs separated by more than 10 meioses, are used to calculate the conditional probability distributions, but are not shown in the figure. Note that total IBD lengths (the vertical axis) are shown on the logarithmic scale.

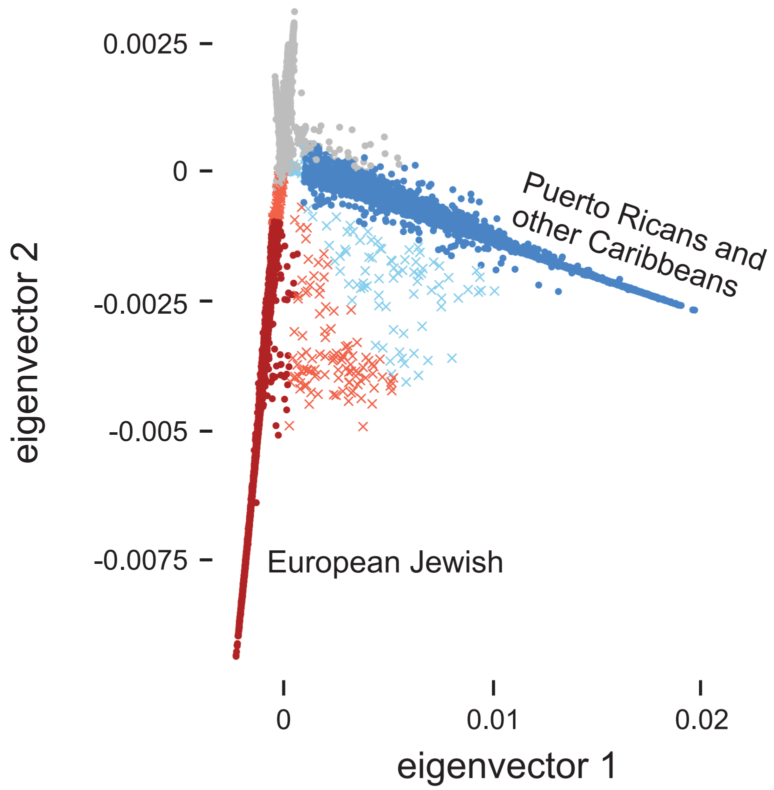

Supplementary Figure 31 | Illustration of how spectral embedding is used to delineate stable subsets. The plot shows the projection of all DNA samples onto the first two dimensions of the spectral embedding. Samples are colored by membership to clusters and corresponding stable subsets. See the text for more details about this figure. Observe that many of the lighter red and lighter blue crosses in this plot lie between the Jewish and Caribbean clusters. These are putatively "admixed" individuals that have ancestors of both Jewish and Caribbean descent.

Supplementary tables

	number of samples	avg. number of pedigree nodes	nodes with all data fields filled in
Not Reported	96,180	46.03	69.87%
Foreign Born	13,748	57.31	70.26%
US Born	322,683	86.25	77.67%
**Total**	432,611	77.78	76.59%
**Mid Atlantic**	**56,570**	**60.29**	**72.45%**
New York	23,632	53.34	72.12%
New Jersey	9,308	56.54	70.82%
Pennsylvania	15,974	67.4	72.64%
Maryland	4,981	77.6	75.19%
**West**	**54,326**	**86.6**	**78.27%**
Hawaii	1,012	62.74	75.58%
California	40,333	83.53	78.26%
Alaska	748	87.7	78.54%
Washington	7,678	94.45	77.68%
Oregon	4,555	105.53	79.55%
**Midwest**	**82,353**	**87.96**	**76.42%**
Illinois	16,597	73.98	74.84%
Wisconsin	5,226	75.16	69.93%
Minnesota	5,717	81	69.92%
North Dakota	920	81.08	66.01%
Michigan	12,186	83.83	76.34%
Ohio	14,713	89.35	77.25%
South Dakota	1,051	91.88	73.20%
Nebraska	2,487	93.58	76.21%
Iowa	4,539	99.51	76.81%
Oklahoma	7,137	102.96	81.83%
Indiana	7,566	103.58	79.18%
Kansas	4,214	105.72	79.17%
**Northeast**	**17,480**	**90.18**	**76.77%**
Delaware	735	72.19	75.03%
Connecticut	3,788	74.6	75.61%
Massachusetts	8,448	83.72	75.79%
Rhode Island	1,301	88.48	75.33%

	number of samples	avg. number of pedigree nodes	nodes with all data fields filled in
Vermont	685	121.24	79.23%
New Hampshire	1,021	129.36	79.25%
Maine	1,502	134.55	80.42%
**South**	**37,200**	**91.43**	**79.90%**
Florida	8,116	82.77	78.92%
Mississippi	2,825	87.55	81.35%
South Carolina	3,168	88.52	79.35%
Georgia	6,640	92.32	80.67%
Louisiana	4,874	93.93	78.56%
Alabama	5,151	97.46	81.39%
North Carolina	6,426	97.86	79.64%
**Southwest**	**10,133**	**92.15**	**78.84%**
Nevada	940	85.39	79.64%
Arizona	2,864	91.6	78.94%
Colorado	4,322	92.77	78.68%
New Mexico	2,007	94.66	78.72%
**South Central**	**55,885**	**98.98**	**80.24%**
Virginia	6,579	93.4	78.31%
Texas	23,017	94.52	80.26%
Arkansas	3,817	97.3	82.05%
Missouri	8,184	98.13	79.35%
Tennessee	6,243	104.44	81.35%
West Virginia	2,835	112.37	80.81%
Kentucky	5,210	113.98	80.75%
**Intermountain**	**7,678**	**116.19**	**79.93%**
Montana	1,423	93.73	77.14%
Wyoming	788	106.66	80.19%
Idaho	1,802	113.41	80.46%
Utah	3,665	128.45	80.44%

Supplementary Table 1 | Summary of genealogical data by US state, and outside the US. Each individual pedigree is assigned only a single location based on the self-reported birth location of the genetic test-taker, and contributes exclusively to statistics for this location. Columns represent the number of samples, average number of nodes per pedigree, and proportion of nodes with complete data (name, birth date, and birth location) for US states and outside the US. For clarity, US states are broken down into regional designations (e.g., "Northeast").

region	samples
Great Britain	111
Ireland (Celtic)	138
Europe East	432
Iberian Peninsula	81
European Jewish	189
Scandinavia	232
Europe South (incl. Italy, Greece)	171
Europe West (incl. France, Germany)	166
Finland and Northwest Russia	59
Africa Southeastern Bantu	18
Africa North	26
Africa South-Central Hunter-Gatherers*	35
Benin/Togo*	60
Cameroon/Congo*	115
Ivory Coast/Ghana*	99
Mali*	16
Nigeria*	67
Senegal*	28
Native American	131
Asia Central	26
Asia East	394
Asia South	161
Melanesia	28
Polynesia	18
Caucasus	58
Near East	141
total	*3,000*

Supplementary Table 2 | Composition of the global ancestry reference panel. For more details on how the ancestral populations are defined, see description of Supplementary Fig. 5, and the AncestryDNA Ethnicity Estimate White Paper[1]. *Admixture proportions for these regions are collapsed into a single admixture proportion representing West Africa.

label	number of samples	IBD (cM) mean	min.	max.
ACB	79	1261	558	2,988
ASW	66	11,034	3,370	36,697
CDX	98	0	0	59
CEU	122	22,167	987	58,161
CHB	108	0	0	629
CHD	1	0	0	0
CHS	101	0	0	53
CLM	70	7,134	202	16,062
FIN	100	9,402	1,540	44,410
GBR	101	5,339	2,325	17,975
GIH	110	103	0	2,355
IBS	100	404	47	3,595
JPT	105	0	0	89
KHV	100	29	0	149
LWK	110	17	0	249
MXL	68	11,027	553	110,296
PEL	70	844	165	3,523
PUR	70	132,038	24,336	216,855
TSI	112	228	0	2,616
YRI	125	43	0	317

Supplementary Table 3 | Summary of IBD shared between 774,516 AncestryDNA samples and 1,816 samples from 1000 Genomes Project. Columns from left to right are: provided population label; number of 1000 Genomes samples assigned that label; average total IBD; smallest total IBD; and largest total IBD. Here, "total IBD" is defined as the sum of estimated IBD segment lengths (in cM) between a given 1000 Genomes sample and all 774,516 AncestryDNA genotype samples. Estimated segment lengths less than 12 cM are treated as having a length of 0 cM.

Hawaiians	European Jewish	New Mexicans	Northeast Mexico	West Mexico	Caribbeans	Colombians	Central Americans	French Canadians	Acadians	Finnish	Scandinavians	Irish	Amish	Utah	Appalachians	
0.084	0.072	0.072	0.072	0.074	0.049	0.072	0.071	0.077	0.069	0.081	0.080	0.078	0.080	0.080	0.080	African Americans
	0.043	0.028	0.028	0.027	0.031	0.027	0.027	0.044	0.039	0.038	0.043	0.047	0.039	0.044	0.041	Hawaiians
		0.013	0.017	0.015	0.010	0.016	0.027	0.003	0.004	0.007	0.005	0.005	0.004	0.004	0.004	European Jewish
			0.002	0.001	0.007	0.003	0.005	0.012	0.010	0.012	0.012	0.014	0.011	0.012	0.012	New Mexicans
				0.001	0.008	0.002	0.003	0.016	0.013	0.015	0.016	0.018	0.015	0.016	0.015	Northeast Mexico
					0.007	0.002	0.003	0.015	0.012	0.014	0.015	0.017	0.013	0.015	0.014	West Mexico
						0.007	0.011	0.009	0.006	0.011	0.011	0.012	0.009	0.010	0.009	Caribbeans
							0.003	0.016	0.013	0.015	0.017	0.019	0.014	0.017	0.015	Colombians
								0.026	0.022	0.023	0.026	0.030	0.023	0.026	0.024	Central Americans
									0.001	0.003	0.001	0.001	0.001	0.000	0.001	French Canadians
										0.004	0.002	0.002	0.001	0.002	0.001	Acadians
											0.002	0.003	0.003	0.002	0.003	Finnish
												0.001	0.001	0.000	0.001	Scandinavians
													0.001	0.000	0.001	Irish
														0.001	0.001	Amish
															0.000	Utah

Supplementary Table 4 | Pairwise F_{ST} values between stable subsets. Note that Dominicans cluster is not included in these results since it is contained within the Caribbean cluster.

	Polynesians and East Asians	European Jewish A	European Jewish B	Portuguese	Cubans and Dominicans	Puerto Rico, East	Puerto Rico, Northwest	Puerto Rico, Southwest	Central Americans and Columbians	New Mexicans	Northeast Mexico	West, Northwest and Central Mexico	Lower South	Upland South	Lower Midwest and Appalachians	French Canadians and Acadians	Midwest immigrants	Italians, Irish and Scottish	Pennsylvania	Northeast and Utah
African Americans	0.084	0.074	0.073	0.050	0.049	0.052	0.048	0.045	0.067	0.071	0.070	0.072	0.081	0.082	0.082	0.075	0.081	0.079	0.081	0.082
Polynesians and East Asians		0.053	0.053	0.045	0.040	0.040	0.044	0.041	0.033	0.036	0.035	0.033	0.056	0.057	0.057	0.054	0.056	0.056	0.057	0.057
European Jewish A			0.000	0.005	0.008	0.007	0.006	0.008	0.020	0.011	0.015	0.020	0.004	0.004	0.004	0.003	0.004	0.003	0.003	0.004
European Jewish B				0.004	0.007	0.006	0.005	0.008	0.019	0.010	0.014	0.019	0.003	0.003	0.003	0.002	0.003	0.002	0.002	0.003
Portuguese					0.002	0.002	0.000	0.002	0.015	0.009	0.011	0.016	0.005	0.005	0.005	0.004	0.006	0.005	0.005	0.005
Cubans and Dominicans						0.001	0.002	0.001	0.009	0.006	0.007	0.010	0.009	0.010	0.009	0.007	0.010	0.009	0.009	0.009
Puerto Rico, East							0.001	0.001	0.009	0.005	0.007	0.010	0.008	0.008	0.008	0.006	0.008	0.007	0.008	0.008
Puerto Rico, Northwest								0.001	0.012	0.008	0.010	0.013	0.007	0.007	0.007	0.005	0.007	0.006	0.007	0.007
Puerto Rico, Southwest									0.010	0.007	0.008	0.011	0.010	0.010	0.010	0.007	0.010	0.009	0.010	0.010
Central Americans and Colombians										0.004	0.002	0.001	0.021	0.021	0.021	0.019	0.021	0.020	0.021	0.021
New Mexicans											0.001	0.002	0.010	0.011	0.011	0.009	0.010	0.010	0.011	0.011
Northeast Mexico												0.001	0.015	0.015	0.015	0.013	0.015	0.014	0.015	0.015
West, Northwest and Central Mexico													0.020	0.020	0.020	0.018	0.020	0.019	0.020	0.020
Lower South														0.000	0.000	0.000	0.001	0.000	0.000	0.000
Upland South															0.000	0.000	0.000	0.000	0.000	0.000
Lower Midwest and Appalachians																0.000	0.000	0.000	0.000	0.000
French Canadians and Acadians																	0.001	0.000	0.000	0.000
Midwest immigrants																		0.001	0.000	0.000
Italians, Irish and Scottish																			0.000	0.000
Pennsylvania																				0.000

Supplementary Table 5 | Pairwise F_{ST} values between clusters identified in IBD sub-networks.

chr	SNP	A	a	disease/trait	locus	OR	cluster	f_0(A)	f_1(A)	n_0(AA)	n_0(Aa)	n_0(aa)	n_1(AA)	n_1(Aa)	n_1(aa)	source
6	rs10484554	A	G	psoriasis	*HLA-C*	4.66	European Jewish	13.0%	25.1%	12,640	164,245	552,397	1,713	9,873	14,905	Strange *et al.* (2010)
6	rs2070600	T	C	pulmonary function	AGER	–	Appalachians	4.5%	7.4%	1,724	64,699	687,777	16	269	1,761	Repapi *et al.* (2010)
11	rs2237897	T	C	type 2 diabetes	*KCNQ1*	0.74	New Mexicans, Northeast and Western Mexico	5.2%	18.8%	2,724	71,126	657,090	451	3,891	8,373	Williams *et al.* (2013)
17	rs7210100	A	G	prostate cancer	*ZNF652*	1.51	African Americans	0.1%	5.6%	27	2,066	709,570	122	4,815	39,987	Haiman *et al.* (2011)
19	rs737337	C	T	high-density lipoprotein	*DOCK6*	–	African Americans	9.5%	41.8%	8,221	119,171	583,044	7,940	21,578	15,306	Willer *et al.* (2013)
20	rs1800961	T	C	high-density lipoprotein	*HNF4A*	–	Scandinavians	2.9%	5.0%	696	41,621	710,066	7	405	3,770	Willer *et al.* (2013)
22	rs17879961	G	A	squamous cell lung carcinoma	*CHEK2*	0.38	Finnish	0.2%	1.9%	15	3189	749781	1	62	1,619	Wang *et al.* (2014)

Supplementary Table 6 | Examples of disease-risk alleles at higher frequencies in specific clusters. The columns of this table from left to right are: chromosome (chr); base-pair position on the chromosome from NCBI release 37 of the human genome assembly (pos); variant entry in dbSNP database, release 142; alternative allele (A); reference allele (a); associated disease or trait; genetic locus, or top candidate gene based on prior studies; *odds ratio*, the multiplicative increase in odds of disease for each copy of the A allele, as reported by the cited publication; cluster in which we find the A allele at a higher frequency; f_0(A), the frequency of the A allele in all genotyped individuals not assigned to the cluster; f_1(A), the frequency of the A allele in genotyped individuals assigned to the cluster; n_0(AA), n_0(Aa), n_0(aa) are the genotype frequencies in samples not assigned to the cluster; n_1(AA), n_1(Aa), n_1(aa) are the genotype frequencies in samples assigned to the cluster; citation providing evidence for trait-SNP association (source). SNPs were identified by selecting SNPs with cluster minor allele frequency at least 1.5 times the frequency outside the cluster, and cross-referencing these SNPs against all entries from the NHGRI/EBI GWAS Catalog with p-value < 10^{-12}. Additional references cited in the table are listed in the supplementary text.

method	batch size	model size	CPU runtime	switch-error rate
BEAGLE	1,188	2,970,907	254 min	2.60%
BEAGLE	2,188	6,353,295	429 min	2.09%
BEAGLE	3,188	9,347,111	616 min	1.90%
BEAGLE	6,188	17,869,941	1361 min	1.63%
Our method	1,188	102,692,825	251 min	0.93%

Supplementary Table 7 | Empirical comparison of haplotype phasing methods. This experiment compares phasing accuracy using BEAGLE version 3.3.2 and different batch sizes against the phasing accuracy using our algorithm with a reference panel learned from 189,503 samples phased in large batches using HAPI-UR. We run BEAGLE using default parameters, except we set `nsamples = 20` (this is the number of haplotype pairs that are sampled for each individual). Phasing error is evaluated in a test set with 1,188 trio-phased samples. Phasing error, or "switch-error rate," is calculated as the rate of disagreement between the estimated phase and the trio-phased haplotype, only for loci in which phase can be determined unambiguously; *i.e.*, sites with at least one homozygous individual in the trio[5, 6]. "Model size" refers to the total number of haplotype Markov model states across all chromosome windows.

European Jewish	Caribbeans	Mexico, Central and South America	Southern US	Northern US and Utah	
0.053	0.040	0.034	0.049	0.056	Polynesians and East Asians
	0.007	0.016	0.004	0.003	European Jewish
		0.008	0.003	0.008	Caribbeans
			0.014	0.016	Mexico, Central and South America
				0.002	Southern US

Supplementary Table 8 | Pairwise F_{ST} estimates between the 6 largest clusters identified in IBD network.
